# Transition‐Metal‐Free Synthesis of Polyfunctional Triarylmethanes and 1,1‐Diarylalkanes by Sequential Cross‐Coupling of Benzal Diacetates with Organozinc Reagents

**DOI:** 10.1002/anie.202101682

**Published:** 2021-03-17

**Authors:** Baosheng Wei, Qianyi Ren, Thomas Bein, Paul Knochel

**Affiliations:** ^1^ Department Chemie Ludwig-Maximilians-Universität München Butenandtstrasse 5–13, Haus F 81377 München Germany

**Keywords:** cross-coupling, diacetates, organozinc reagents, transition-metal-free, triarylmethane and 1,1-diarylalkane

## Abstract

A variety of functionalized triarylmethane and 1,1‐diarylalkane derivatives were prepared via a transition‐metal‐free, one‐pot and two‐step procedure, involving the reaction of various benzal diacetates with organozinc reagents. A sequential cross‐coupling is enabled by changing the solvent from THF to toluene, and a two‐step S_N_1‐type mechanism was proposed and evidenced by experimental studies. The synthetic utility of the method is further demonstrated by the synthesis of several biologically relevant molecules, such as an anti‐tuberculosis agent, an anti‐breast cancer agent, a precursor of a sphingosine‐1‐phosphate (S1P) receptor modulator, and a FLAP inhibitor.

## Graphical abstract

A sequential cross‐coupling of benzal diacetates with organozinc reagents is achieved, delivering various functionalized triarylmethane and 1,1‐diarylalkane derivatives. This practical method features readily accessible substrates, transition‐metal‐free and one‐pot conditions, wide scope, scalability, and synthetic utility in the efficient synthesis of biologically relevant molecules.

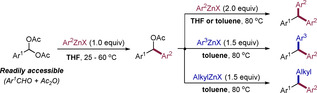

## Introduction

**Scheme 1:**
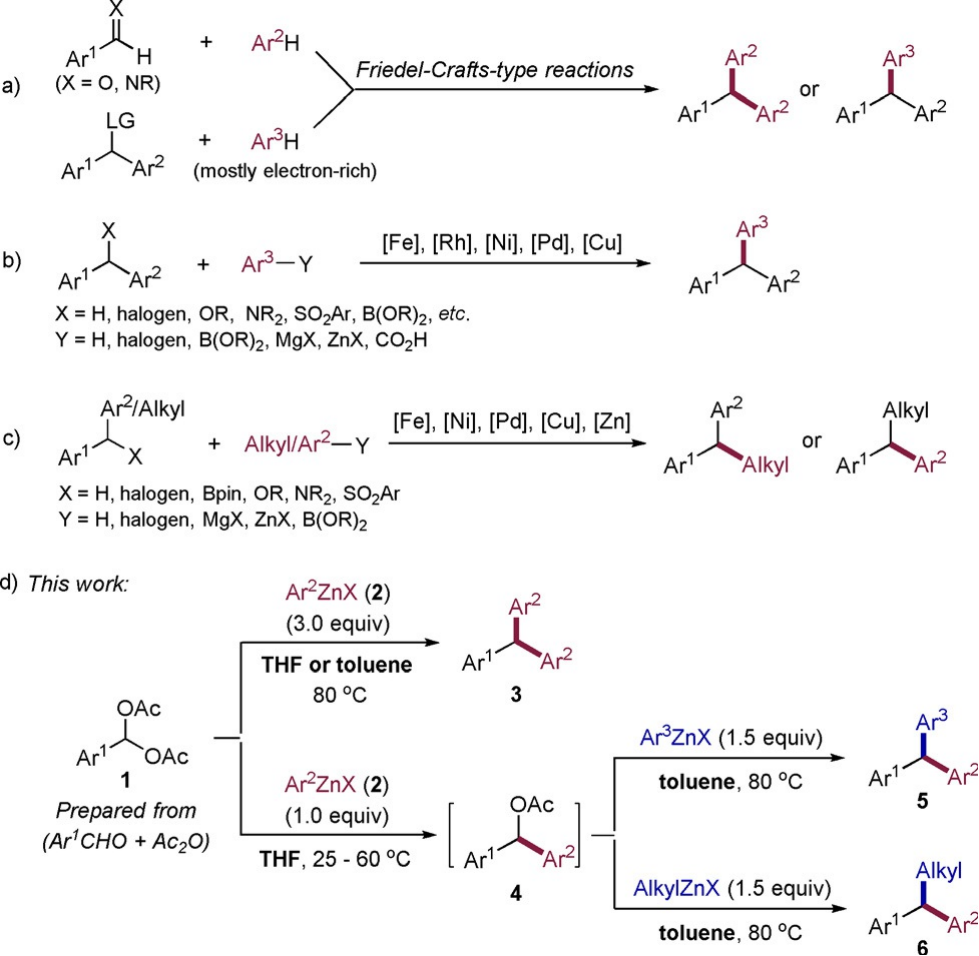
Typical methods for the synthesis of triarylmethanes and 1,1‐diarylalkanes.

Triarylmethane and 1,1‐diarylalkane scaffolds are important core structures in many pharmaceuticals and biologically active molecules,^[1]^ and are potentially valuable building blocks for the construction of covalent organic and metal–organic frameworks (COFs and MOFs) that can play a role in hydrogen storage, photocatalysis, photoelectrochemistry, and solar cells.^[2]^ Thus, their preparation has attracted much attention over the past decade.^[3]^ Typically, triarylmethanes may be prepared by Friedel–Crafts‐type reactions (Scheme 1 a),[^4^, ^5^] or by various transition‐metal‐catalyzed cross‐coupling reactions (Scheme 1 b).[^6^, ^7^, ^8^] These methods were also used for the preparation of related 1,1‐diarylalkanes (Scheme 1 c).[^9^, ^10^] Recently, some other methods have also been developed for the synthesis of triarylmethanes and 1,1‐diarylalkanes.[^11^, ^12^] Despite the popularity of these methods, there are some important drawbacks. For example, Friedel–Crafts‐type reactions are typically limited to electron‐rich and unhindered (hetero)arenes and often result in poor regioselectivity.[^4^, ^9^] Cross‐coupling methods usually require the troublesome prefunctionalization of coupling partners, and β‐hydride elimination of alkyl or benzylic reagents in transition‐metal‐involved cross‐couplings often leads to non‐productive synthesis.[^6^, ^7^, ^10^] Thus, the selective and modular synthesis of polyfunctional triarylmethanes and 1,1‐diarylalkanes from readily accessible starting materials^[13]^ under transition‐metal‐free conditions is still an important synthetic goal, and a general method that can deliver both triarylmethanes and 1,1‐diarylalkanes would be highly desirable.^[14]^

Zinc organometallics are very useful organometallic intermediates for forming new carbon−carbon bonds, and allow the synthesis of a variety of polyfunctional organic molecules.^[15]^ Usually, transition‐metal catalysts are required for achieving good yields and selectivities.[^15c^, ^15d^] Only a few reactions of organozinc reagents with electrophiles proceed in the absence of catalysts.^[16]^ We envisioned that benzal *gem*‐diacetates of type **1**, which can be easily prepared from the corresponding aldehydes,^[17]^ may be an ideal class of electrophiles for reaction with organozinc reagents allowing a modular synthesis of triarylmethanes and 1,1‐diarylalkanes (Scheme 1 d). Herein, we wish to report a convenient transition‐metal‐free, one‐pot and two‐step synthesis of triarylmethanes and 1,1‐diarylalkanes starting from **1**. Thus, the treatment of **1** with excess arylzinc halides of type **2** (Ar^2^ZnX)^[18]^ either in THF or toluene at 80 °C smoothly provides the symmetrical triarylmethanes of type **3**. However, the treatment of **1** with **2** (1.0 equiv) in THF at 25–60 °C selectively provides the diarylmethyl acetate (**4**), which reacts in situ with aryl‐ or alkylzinc halides (Ar^3^ZnX or alkylZnX) in toluene at 80 °C, producing either triarylmethanes of type **5** or 1,1‐diarylalkanes of type **6**.

## Results and Discussion

**Scheme 2:**
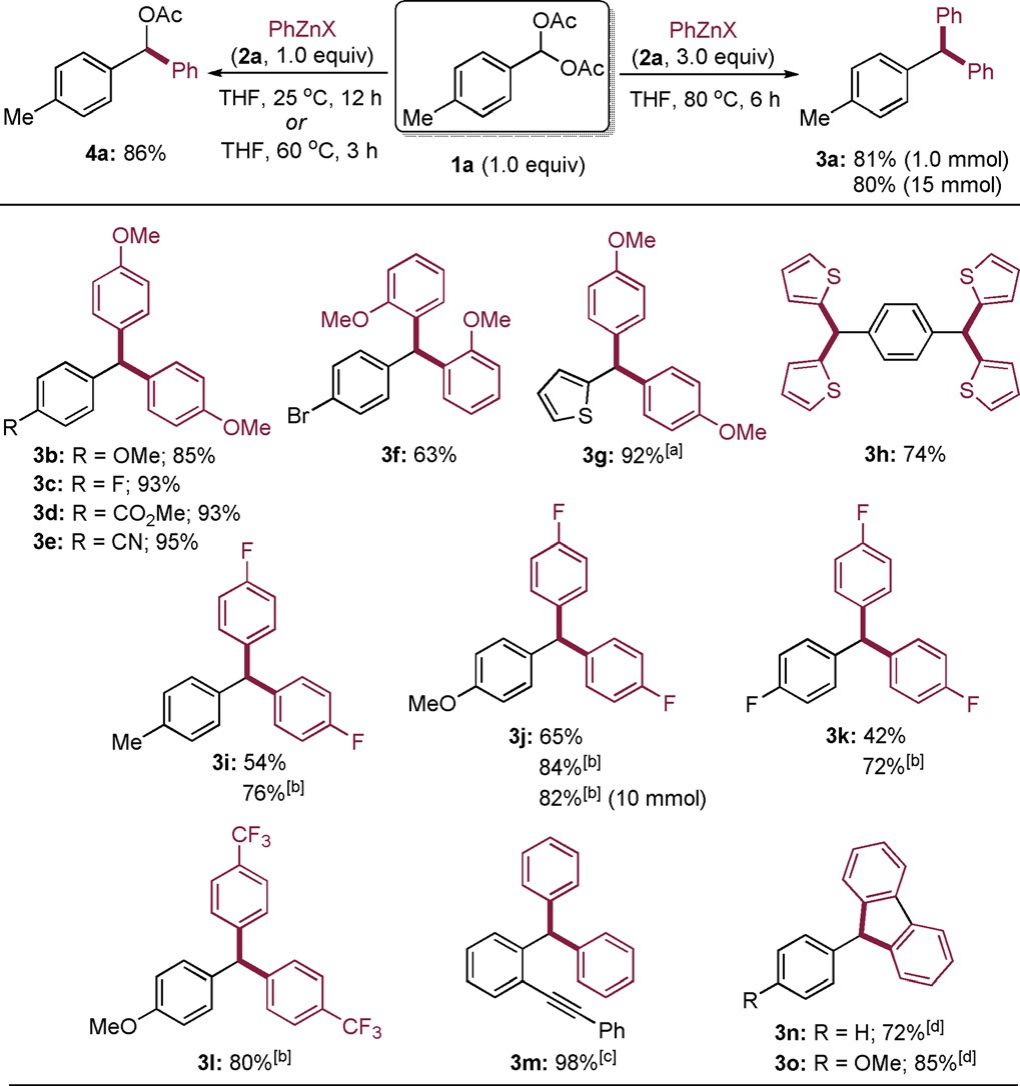
Synthesis of symmetrical triarylmethanes from benzal diacetates (**1**) and (hetero)arylzinc reagents (**2**). [a] The reaction was performed at room temperature and was completed within 3 h. [b] Toluene, 80 °C, 1 h. [c] Toluene, 120 °C, 1 h. [d] The 2,2′‐bis‐zincated biphenyl was prepared by adding ZnCl_2_ into the corresponding bis‐magnesiated or cyclometalated lanthanum reagents.^[19]^

In preliminary experiments, we have treated the benzal diacetate **1 a** (1.0 equiv) with PhZnX (**2 a**, 1.0 equiv, X=Cl⋅MgCl_2_) at 25 °C in THF for 12 h and have observed the exclusive formation of the mono‐substituted product **4 a** in 86 % isolated yield. Alternatively, heating the reaction mixture at 60 °C also led to a full conversion after 3 h.^[17]^ On the other hand, using an excess of **2 a** (3.0 equiv) and heating the reaction mixture at 80 °C for 6 h produced the double‐substituted product **3 a** in 81 % isolated yield. Notably, a scale‐up of this reaction (15 mmol) provided the similar yield of **3 a** (Scheme 2).

Also, the *p*‐methoxybenzal diacetate (**1 b**) reacted well with 4‐MeOC_6_H_4_ZnX (**2 b**) providing the triarylmethane **3 b** in 85 % yield. Similarly, various benzal diacetates (**1 c**–**1 e**) reacted with **2 b** providing the triarylmethanes **3 c**–**3 e** in 93–95 % yield. The more sterically hindered organozinc reagent 2‐MeOC_6_H_4_ZnX (**2 c**) also gave the triarylmethane **3 f** in 63 % yield. Products of type **3** bearing heterocyclic rings, such as **3 g** and **3 h**, were readily prepared by this method showing that the heterocyclic moiety can be attached either to the benzal part or to the organozinc reagent. However, by using electron‐poor arylzinc reagents such as *p*‐fluorophenylzinc halide (**2 d**), the substitution reaction with *p*‐methylbenzal diacetate (**1 a**) proceeded quite sluggishly in THF at 80 °C providing **3 i** in 54 % yield after 12 h, but much faster and high‐yielding in toluene at 80 °C (76 % yield, 1 h). A similar behavior was noticed in the reaction of *p*‐methoxy (**1 b**) and *p*‐fluoro (**1 c**) benzal diacetates with **2 d**, and yields of 65 % (**3 j**: 80 °C, 12 h) and 42 % (**3 k**: 80 °C, 12 h) were obtained in THF, whereas in toluene a clean reaction produced the triarylmethanes **3 j** and **3 k** in 84 % and 72 % yield (80 °C, 1 h), respectively. A scale‐up of these reactions was possible and **3 j** was obtained in 82 % yield at a 10 mmol scale. The preparation of triarylmethanes bearing electron‐withdrawing substituents was difficult by Friedel–Crafts reactions, however, using arylzinc halides bearing electron‐poor substituents such as 4‐CF_3_C_6_H_4_ZnX (**2 e**) allowed the preparation of the corresponding triarylmethane **3 l** in 80 % yield. Also, the presence of an ethynyl substituent in the benzal diacetate was well tolerated and a cross‐coupling with PhZnX provided the desired product **3 m** in 98 % yield. Finally, the reaction of 2,2′‐bis‐zincated biphenyl with various benzal diacetates led to 9‐aryl‐fluorene derivatives such as **3 n** and **3 o** in 72–85 % yield (Scheme 2).

**Scheme 3:**
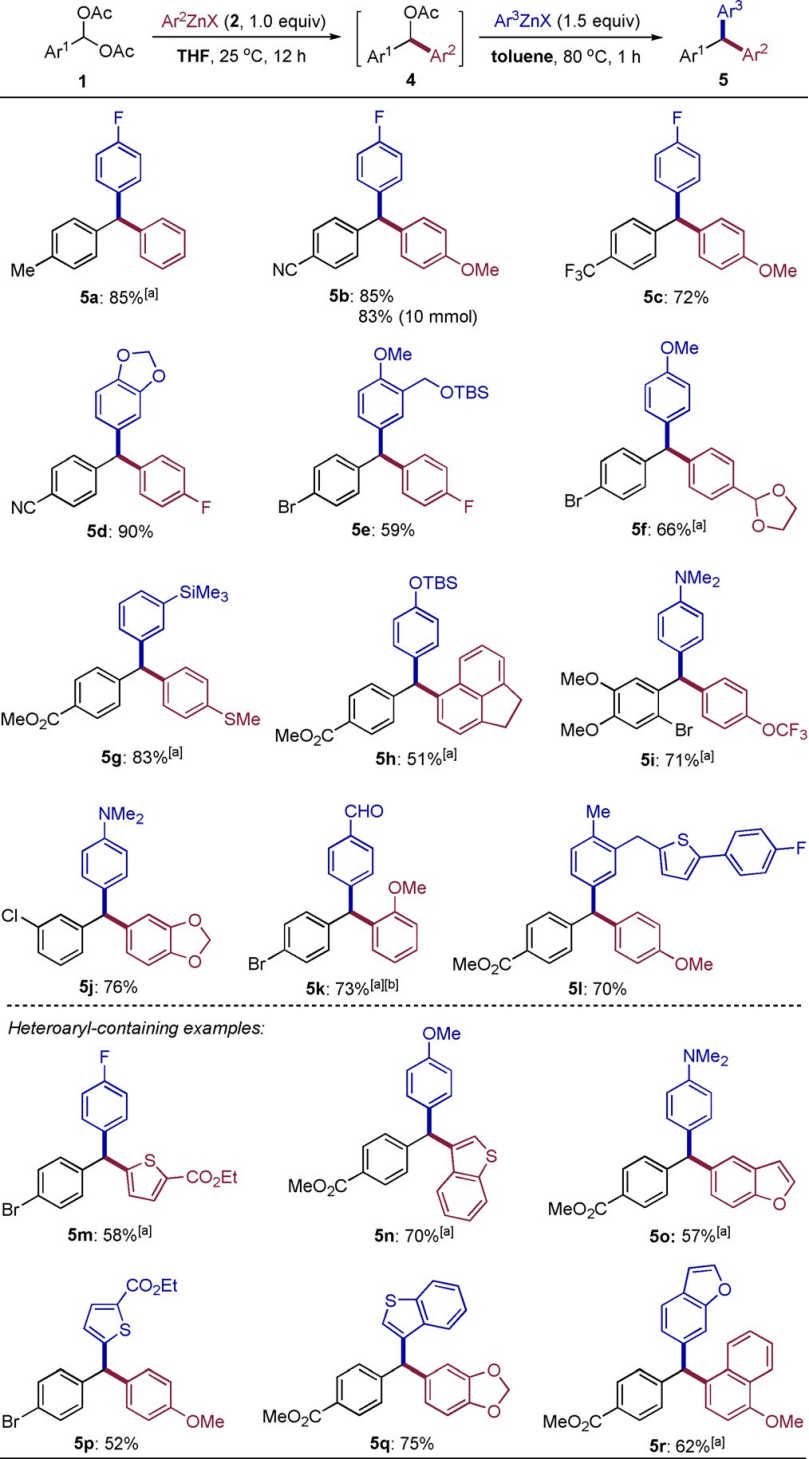
One‐pot synthesis of unsymmetrical triarylmethane derivatives. [a] In the first step, the reaction mixture was heated in THF at 60 °C for 3–12 h. [b] The aldehyde moiety was generated after work‐up from a protecting dimethyl acetal.

A one‐pot selective double arylation of benzal diacetates of type **1** can be readily achieved (Scheme 3). First, the treatment of **1** with Ar^2^ZnX (**2**, 1.0 equiv) in THF at 25 °C selectively generated the mono‐substituted product of type **4**, and heating the reaction mixture at 60 °C was necessary in some cases to achieve a full conversion in this step. Then, after addition of a second arylzinc reagent (Ar^3^ZnX) and subsequent removal of THF in vacuum, toluene was added and the reaction mixture was heated typically at 80 °C for 1 h, leading to various unsymmetrical triarylmethanes **5 a**–**5 l** in 51–90 % isolated yield. Heteroarylzinc reagents such as thienyl‐, benzothienyl‐, or benzofuranylzinc halides can be used in the first or second arylation providing triarylmethanes **5 m**–**5 r** in 52–75 % yield. A range of functional groups were well‐tolerated in the benzal diacetates of type **1** (CN, CF_3_, Br, Cl, CO_2_Me, OMe) as well as in (hetero)arylzinc reagents (F, OMe, OCF_3_, SMe, acetal, SiMe_3_, OTBS, NMe_2_, CO_2_Et). These reactions were scalable as exemplified in the case of **5 b** obtained in 83 % yield at a 10 mmol scale. Polycyclic arylzinc halides were also suited affording **5 h** and **5 r**. The aldehyde group in product **5 k** was introduced by using 4‐dimethoxymethylphenylzinc halide (**2 f**)^[17]^ in the reaction performing the deprotection during work‐up. Notably, several compouds obtained by this method were otherwise unavailable using previously reported methods,[^4^, ^8^] which shows the versatility and synthetic utility of this reaction.

**Scheme 4:**
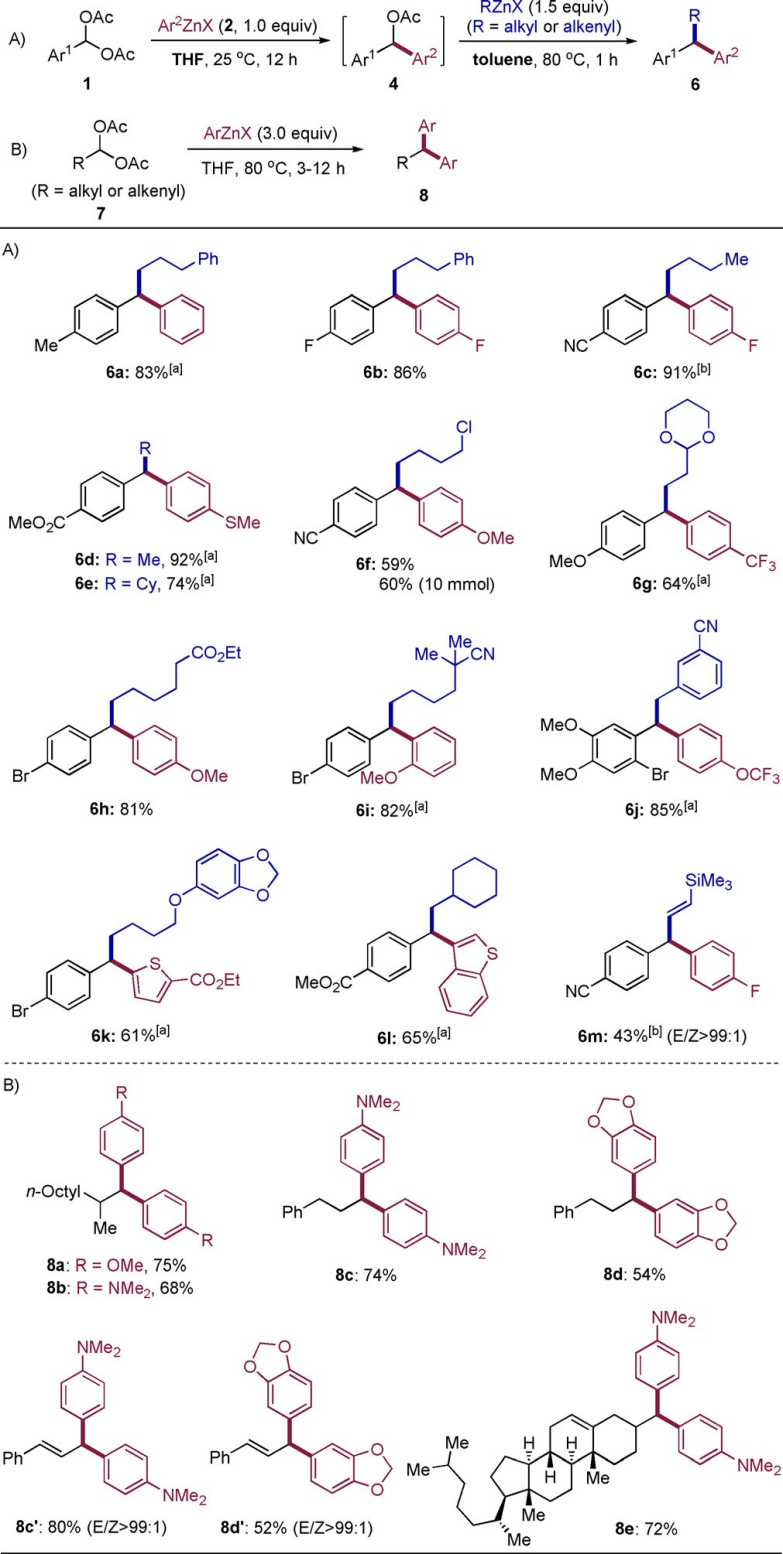
One‐pot synthesis of 1,1‐diarylalkane derivatives of type **6** and **8**. [a] In the first step, the reaction mixture was heated in THF at 60 °C for 3–12 h. [b] The second step required heating in toluene at 120 °C for 3 h.

The above‐mentioned method can be extended to the synthesis of nonsymmetrical 1,1‐diarylalkanes of type **6** by adding a second alkyl‐ or alkenylzinc reagent to the in situ formed diarylmethyl acetate **4**, followed by heating the reaction mixture in toluene typically at 80 °C for 1 h (Scheme 4 A). Thus, various 1,1‐diarylalkane derivatives **6 a**–**6 l** were obtained in 59–92 % yield. A scale‐up of these reactions was also feasible as shown for the formation of **6 f** in 60 % yield at a 10 mmol scale. Also, many functional groups were well‐tolerated in the benzal diacetates of type **1** (CN, CO_2_Me, OMe, F, Br) as well as in (hetero)arylzinc reagents (F, SMe, OMe, CF_3_, OCF_3_, CO_2_Et) and in alkylzinc reagents (Cl, acetal, CO_2_Et, CN, cyclohexyl). Besides, *E*‐2‐trimethylsilylalkenylzinc halide was used in this reaction providing **6 m** in 43 % yield with full configurational retention of the double bond (*E*/*Z*>99:1). Furthermore, symmetrical 1,1‐diarylalkanes of type **8** were also obtained starting from the corresponding alkyl or alkenyl *gem*‐diacetates of type **7** (Scheme 4 B). In comparison, the solvent effect of these reactions was unconspicuous and the scope of arylzinc reagents was narrow. Only electron‐rich arylzinc reagents with NMe_2_ or OR substituents gave good results. All efforts to prepare nonsymmetrical 1,1‐diarylalkanes starting from diacetates of type **7** failed. As shown in Scheme 4 B, 1,1‐diarylalkanes **8 a**–**8 d** were obtained in 54–75 % yield. Diacetates with an adjacent *E*‐alkenyl moiety reacted with full configurational retention (*E*/*Z*>99:1), providing **8 c′** and **8 d′** in 80 % and 52 % yield, respectively. Besides, a cholesteryl *gem*‐diacetate gave the expected product **8 e** in 72 % yield, showing that the reaction may be useful in late‐stage functionalization of steroids and other complex substrates.

**Scheme 5:**
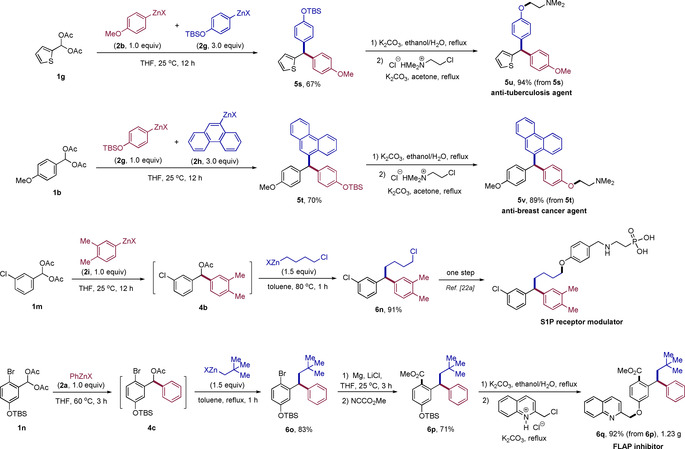
Synthesis of various biologically relevant compounds.

To further demonstrate the synthetic utility of this reaction, we targeted the synthesis of several biologically relevant compounds. However, anti‐tuberculosis agent **5 u** ^[20a]^ and anti‐breast cancer agent **5 v** ^[20b]^ were obtained in low yield by the above two‐step procedure, because the formation of quinonemethide precursors will easily lead to symmetrical triarylmethanes in the above conditions.^[21]^ Thus, as shown in Scheme 5, **5 u** and **5 v** were prepared by a modified procedure, which makes use of the reactivity difference of organozinc reagents.^[17]^ The dropwise addition of a mixture of **2 b** (1.0 equiv) and **2 g** (3.0 equiv) to **1 g** over 12 h led to **5 s** in 67 % yield. Similarly, the mixture of **2 g** (1.0 equiv) and **2 h** (3.0 equiv) was added dropwise to **1 b** leading to **5 t** in 70 % yield. Follow‐up desilylation and alkylation led to the desired anti‐tuberculosis agent **5 u** (94 % yield) and anti‐breast cancer agent **5 v** (89 % yield). Besides, a precursor (**6 n**) of a sphingosine‐1‐phosphate (S1P) receptor modulator^[22a]^ was obtained in 91 % yield by sequential cross‐coupling of **1 m** with **2 i** and 4‐chlorobutylzinc halide under indicated conditions. Also, the FLAP inhibitor **6 q** ^[22b]^ was prepared in 5 steps (1.23 g, 54 % overall yield starting from **1 n**). Specifically, the sequential cross‐coupling of **1 n** with **2 a** and neopentylzinc halide led to **6 o** in 83 % yield. **6 o** was treated with magnesium in the presence of LiCl to produce a magnesium reagent which was treated with NCCO_2_Me providing **6 p** in 71 % yield. Follow‐up desilylation and alkylation led to **6 q** in 92 % yield.

To examine the salt effect on the reaction, control experiments^[17]^ were done showing that the involved halide ions, LiCl, and MgCl_2_ have no observable effect, and ZnCl_2_ may have a limited accelerating effect on the reaction.^[23]^ To examine whether any transition metal catalysts were present, ICP‐MS analysis was performed on solvents (THF and toluene), a representative substrate (**1 a**), organozinc reagent (**2 a**), and their final reaction mixture, indicating the absence of transition‐metal catalysis.^[24]^

**Scheme 6:**
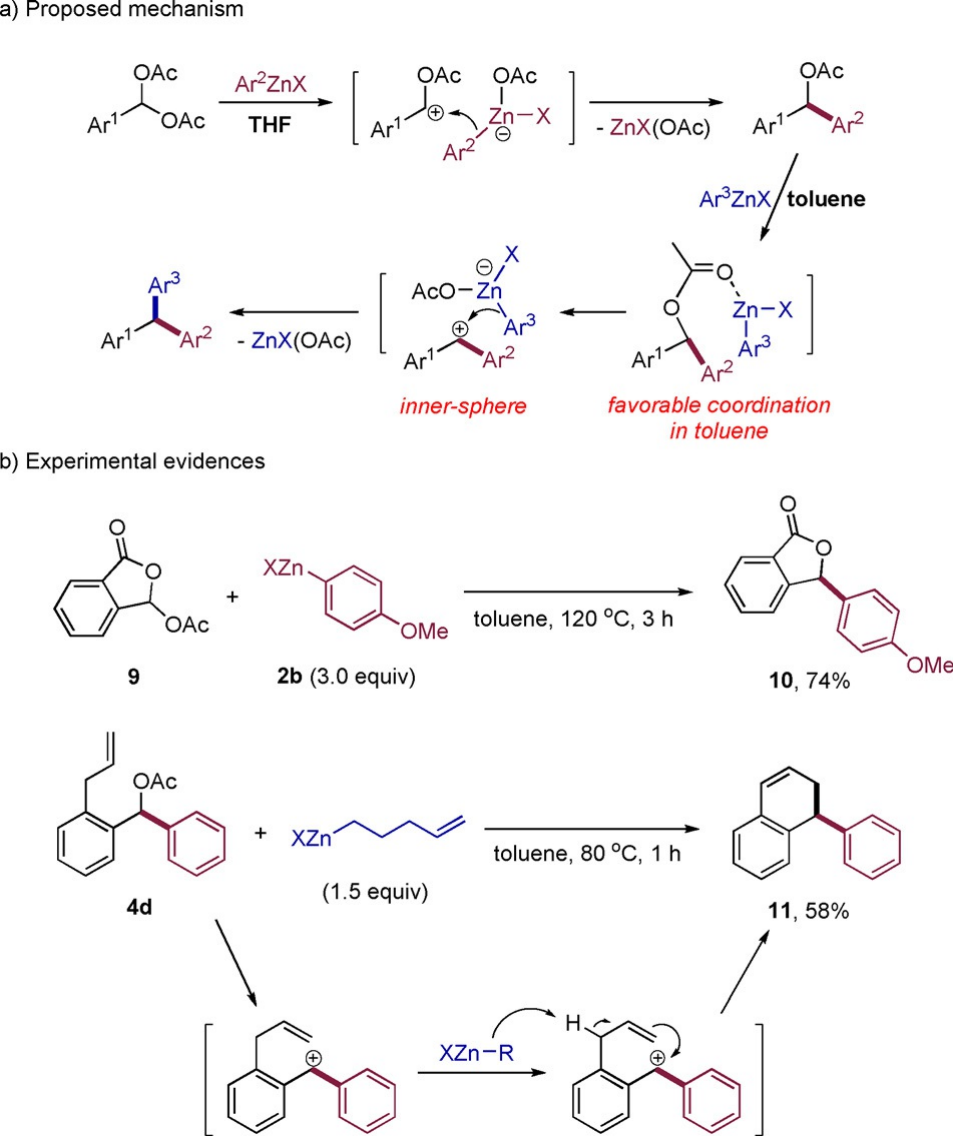
Proposed mechanism (a) and some experimental evidences (b).

To elucidate the mechanism of the reaction, we proposed that the first step was a nucleophilic addition of the arylzinc species to an in situ formed ketone oxonium. The second step may proceed by virtue of the coordination of the zinc center with the acetyl moiety, followed by a nucleophilic addition to the generated benzhydryl cation to form the final product (Scheme 6 a). Toluene favors coordination of zinc reagents, thus promoting the reaction. For experimental evidence, an intramolecular diacetate **9** was treated with excess **2 b** in toluene at 120 °C for 3 h, only generating the mono‐substituted product **10** in 74 % isolated yield. The difficulty of a second substitution on **10** may be explained by the extra stabilization of the lactone **10**. An allyl‐substituted benzhydryl acetate **4 d** was treated with excess 1‐penten‐5‐ylzinc halide providing **11** in 58 % yield, which revealed the possible formation of a benzhydryl carbocation in this reaction (Scheme 6 b).

## Conclusion

We have developed a useful method for the preparation of functionalized triarylmethane and 1,1‐diarylalkane derivatives from readily available diacetates and organozinc reagents. This one‐pot reaction is transition‐metal‐free, also featuring its versatility, scaleability, wide scope, and synthetic utility in the efficient synthesis of biologically relevant molecules. The solvent effect (toluene vs. THF) is remarkable. A two‐step S_N_1‐type mechanism was proposed and partly evidenced by experimental study. Further studies for applications in material science are underway in our laboratories.

## Conflict of interest

The authors declare no conflict of interest.

## Supporting information

As a service to our authors and readers, this journal provides supporting information supplied by the authors. Such materials are peer reviewed and may be re‐organized for online delivery, but are not copy‐edited or typeset. Technical support issues arising from supporting information (other than missing files) should be addressed to the authors.

Supplementary
