# Supplementary material for: Transition‐Metal‐Free Synthesis of Polyfunctional Triarylmethanes and 1,1‐Diarylalkanes by Sequential Cross‐Coupling of Benzal Diacetates with Organozinc Reagents
Source: Angew Chem Int Ed Engl. 2021 Mar 17;60(18):10409–14. doi: 10.1002/anie.202101682 (PMC8252654; doi:10.1002/anie.202101682)
Supplement: Supplementary file 1 — Supplementary [file ANIE-60-10409-s001.pdf]

Angewandte Chemie
Eine Zeitschrift der Gesellschaft Deutscher Chemiker

Supporting Information

Transition-Metal-Free Synthesis of Polyfunctional Triarylmethanes and 1,1-Diarylalkanes by Sequential Cross-Coupling of Benzal Diacetates with Organozinc Reagents

*Baosheng Wei, Qianyi Ren, Thomas Bein, and Paul Knochel**

anie_202101682_sm_miscellaneous_information.pdf

Supporting Information

Table of Contents

1. General Information…… S2
2. Preparation of *gem*-Diacetates…… S5
3. Reaction Optimization…… S6
4. General Procedures…… S9
5. Preparation and Characterization Data of Compounds…… S11
6. ICP-MS analysis…… S44
7. Copies of NMR Spectra
   NMR Spectra of **1i, 1n, 1o, 7a**…… S45
   NMR Spectra of **4a**…… S49
   NMR Spectra of **3a-3o**…… S50
   NMR Spectra of **5a-5v**…… S67
   NMR Spectra of **6a-6q**…… S93
   NMR Spectra of **8a-8e**……S112
   NMR Spectra of **10** and **11**……S119

1. General Information

Unless otherwise stated, all reactions were carried out with magnetic stirring and in flame-dried glassware under argon or nitrogen atmosphere. Syringes used to transfer anhydrous solvents or reagents were purged thrice with argon or nitrogen prior to use. All reactions were monitored by gas chromatography (GC and GC-MS) and thin layer chromatography (TLC). Indicated yields are isolated yields of compounds estimated to be >95% pure as determined by ^{1}H-NMR (25 °C) and capillary GC analysis.

Solvents

THF was continuously refluxed and freshly distilled from sodium benzophenone ketyl under nitrogen. **Toluene** was continuously refluxed and freshly distilled from sodium under nitrogen. Solvents for column chromatography were distilled on a rotary evaporator prior to use.

Reagents

All reagents obtained from commercial sources were used without further purification.

$ZnCl_2$: $ZnCl_2$ solution (1.0 M) was prepared by drying $ZnCl_2$ (200 mmol, 27.3 g) in a Schlenk-flask under vacuum at 160 °C for 5 h. After cooling, dry THF (200 mL) was added and stirring continued until the salt was dissolved.

ArZnX and AlkylZnX:

Method A: *Preparation of Ar- and AlkylZnX by adding $ZnCl_2$ into the corresponding magnesium reagents:*

Magnesium turnings (110 mg, 4.5 mmol) and LiCl (153 mg, 3.6 mmol) were placed in a dry Schlenk tube equipped with a magnetic stirrer and a rubber septum under argon. Under vacuum (<1 mbar), the mixture was dried by a heating gun first at 260 °C for a few seconds, and then at 500 °C for 5 min until the vacuum degree had no change. After cooling and adding THF, the mixture was activated by adding $BrCH_2CH_2Br$ (2.0 mol%) and TMSCl (1.5 mol%) followed by gently heating. The resulting mixture was stirred for 5 min to be cooled to room temperature. Aryl or alkyl bromide/iodide (3.0 mmol) was then added in one portion, and the reaction mixture was stirred under room temperature until the insertion was completed according to GC analysis.[1] The resulting Grignard reagent was transferred with a syringe into a solution of $ZnCl_2$ (3.6 mL, 1M in THF, 3.6 mmol). This mixture was stirred at room temperature for 10 min and the resulting organozinc reagent was titrated with iodine[2] prior to

---

1 F. M. Piller, P. Appukkuttan, A. Gavryushin, M. Helm, P. Knochel, *Angew. Chem. Int. Ed.* **2008**, *47*, 6802−6806; *Angew. Chem.* **2008**, *120*, 6907−6911.

2 A. Krasovskiy, P. Knochel, *Synthesis* **2006**, *5*, 890−891.

use.

Method B: *Preparation of Ar- and AlkylZnX by inserting magnesium into organic halides in the presence of $ZnCl_2$*

Magnesium turnings (110 mg, 4.5 mmol) and LiCl (153 mg, 3.6 mmol) were placed in a dry Schlenk tube equipped with a magnetic stirrer and a rubber septum under argon. Under vacuum (<1 mbar), the mixture was dried by a heating gun first at 260 °C for a few seconds, and then at 500 °C for 5 min until the vacuum degree had no change. After cooling and adding THF, the mixture was activated by adding $BrCH_2CH_2Br$ (2.0 mol%) and TMSCl (1.5 mol%) followed by gently heating. The resulting mixture was stirred for 5 min to be cooled to room temperature. Then, a solution of $ZnCl_2$ (3.6 mL, 1M in THF, 3.6 mmol) was added and the mixture was further stirred for 5 min. Aryl or alkyl bromide/iodide (3.0 mmol) was then added in one portion, and the reaction mixture was stirred under room temperature until the insertion was completed according to GC analysis.[1] The resulting organozinc reagent was titrated with iodine[2] prior to use.

(The labelled organozinc reagents in this paper are shown as follows:)

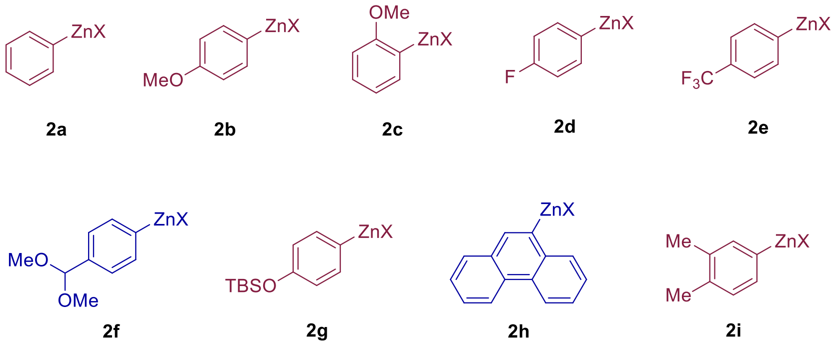

Chromatography

Flash column chromatography was performed using SiO_2 60 (0.040-0.063 mm, 230-400 mesh ASTM) from Merck. Thin layer chromatography (TLC) was performed using aluminum plates covered with SiO_2 (Merck 60, F-254). The chromatograms were examined under 254 nm UV irradiation.

Analytical Data

Gas chromatography (GC) was performed with instruments of the type Hewlett-Packard 6890 or 5890 Series II, using a column of the type HP 5 (Hewlett-Packard, 5% phenylmethylpolysiloxane; length: 10 m, diameter: 0.25 mm, film thickness: 0.25 μm). The detection was accomplished using a flame ionization detector.

High resolution mass spectroscopy (HRMS) and **low resolution mass spectra (MS)** were recorded on a FINNIGAN MAT 95Q instrument. Electron impact ionization (EI) was conducted

with an ionization energy of 70 eV. For coupled gas chromatography/mass spectrometry, a HEWLETT-PACKARD HP 6890/MSD 5973 GC/MS system was used. Molecular fragments are reported starting at a relative intensity of 10-20%.

^{1}H-NMR and **^{13}C-NMR** spectra were recorded on BRUKER ARX 400, VARIAN VXR 400 S and BRUKER AMX 600 instruments. Chemical shifts are reported as δ-values in parts per million (ppm) relative to the deuterated solvent residual peak ($CDCl_3$: δ = 7.26 ppm for ^{1}H-NMR and 77.0 ppm for ^{13}C-NMR). For the characterization of the observed signal multiplicities, the following abbreviations were used: s (singlet), d (doublet), t (triplet), q (quartet), quint (quintet), dd (doublet of doublets), m (multiplet) and br (broad).

Infrared spectra (IR) were recorded from 4500 cm^{-1} to 650 cm^{-1} on a PERKIN ELMER Spectrum BX-59343 instrument. A SMITHS DETECTION DuraSampl*IR* II Diamond ATR sensor was used for detection. Samples were measured neat and the main absorption peaks are reported in cm^{-1}.

Melting points (m.p.) were determined on a BÜCHI B-540 melting point apparatus and are uncorrected.

2. Preparation of *gem*-Diacetates

2-1 *Preparation of benzal gem-diacetates (**1**) from aromatic aldehydes*

To a solution of aromatic aldehyde (20 mmol) in acetic anhydride (40 mmol) was added *p*-TSA·H_2O (2 mmol, 10 mol%). The reaction mixture was stirred at room temperature overnight. Saturated sodium bicarbonate aqueous solution was added to neutralize the excess acetic anhydride. The mixture was washed with diethyl ether for three times to extract the product. The combined organic phase was dried over Na_2SO_4, filtered, and the solvent was evaporated to give the desired benzal *gem*-diacetate in pure form.[3] (^{1}H and ^{13}C-NMR spectra of known compounds are identical with reported data.)

(Substrates **1a**-**1o** used in this manuscript are shown as follows:)

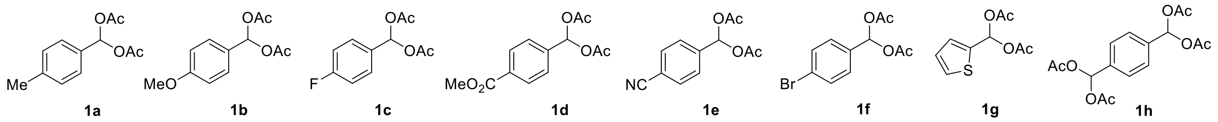

1a **1b** **1c** **1d** **1e** **1f** **1g** **1h**

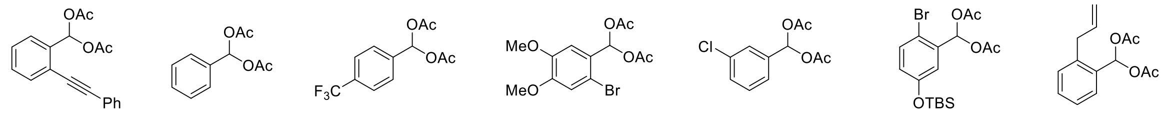

1i **1j** **1k** **1l** **1m** **1n** **1o**

2-2 *Preparation of alkyl gem-diacetates (**7**) from aliphatic aldehydes*

To a solution of aliphatic aldehyde (20 mmol) in chloroform (20 mL) was added acetic anhydride (100 mmol) and iodine (2 mmol, 10 mol%). The reaction mixture was stirred at room temperature overnight. Saturated sodium bicarbonate aqueous solution was added to neutralize the excess acetic anhydride. The reaction mixture was extracted with dichloromethane for three times. The combined organic phase was washed by saturated sodium thiosulfate solution for three times and water for one time. The organic phase was dried over Na_2SO_4, filtered and the solvent was evaporated under reduced pressure to give the desired alkyl *gem*-diacetate in pure form.[4] (^{1}H and ^{13}C-NMR NMR spectra of known compounds are identical with reported data.)

(Substrates **7a**-**7c** used in this manuscript are shown as follows:)

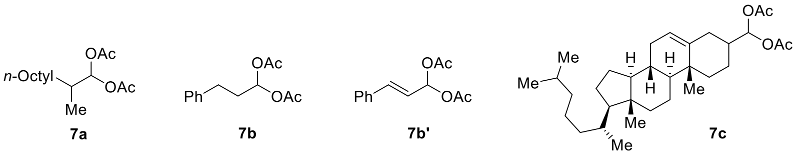

7a **7b** **7b'** **7c**

[3] R. S. L. Chapman, J. D. Tibbetts, S. D. Bull, *Tetrahedron* **2018**, *74*, 5330−5339.
[4] N. Deka, D. J. Kalita, R. Borah, J. C. Sarma, *J. Org. Chem.* **1997**, *62*, 1563−1564.

3. Reaction Optimization

In preliminary experiments, we found that under catalyst-free conditions, the benzal diacetate **1a** (1.0 mmol, 1.0 equiv) can react with phenylzinc halide (**2a**, 1.0 equiv) at 25 °C in THF, forming exclusively the mono-substituted product **4a** after 12 h (Table S1, entry 2); Heating the reaction mixture at 60 °C allowed a faster conversion to **4a** (entry 3). The reaction of **1a** with excess **2a** (3.0 equiv) at room temperature also led to **4a** exclusively (entry 4). However, when the reaction of **1a** with excess **2a** (3.0 equiv) was performed at 60 °C for 12 h, the triarylmethane product (**3a**) was formed in 51% yield (entry 5). Heating the reaction mixture at 80 °C for 6 h produced **3a** exclusively in 81% isolated yield (entry 6).

Table S1. Condition Screening for the selective synthesis of **4a** and **3a**

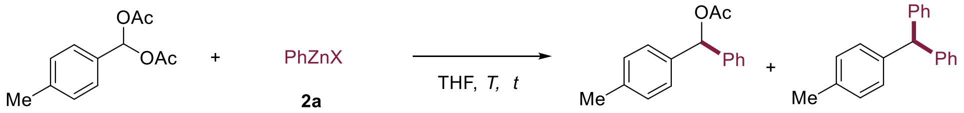

1a (1.0 mmol) **2a** (X = Cl·$MgCl_2$) **4a** **3a**

Entry	**2a**	T (°C)	t (h)	Yield of **4a** (%)[a]	Yield of **3a** (%)[a]
1	1.0 equiv	25	6	67	0
2	1.0 equiv	25	12	92 (86[b])	0
3	1.0 equiv	60	3	92	trace
4	3.0 equiv	25	12	95	trace
5	3.0 equiv	60	12	45	51
6	3.0 equiv	80	6	0	93 (81[b])
7	2.5 equiv	80	6	3	91

[a] GC yield; Hexamethylbenzene was used as an internal standard. [b] Isolated yield.

As organozinc reagents can be prepared by different methods, it's necessary to check if the involved halide ions, LiCl, $MgCl_2$, and $ZnCl_2$ have any effect on this reaction. As shown in Table S2, the reaction of *p*-fluorobenzal diacetate (**1c**, 1.0 mmol, 1.0 equiv) with 4-$MeOC_6H_4ZnX$ (**2b**, 3.0 equiv) was used as a test reaction, which was performed in THF at 80 °C for 12 h. **2b** can be prepared by transmetalation of ArMgX (entries 1-4) or ArLi (entry 5) with $ZnCl_2$ (1.2 equiv relative to ArLi or ArMgX). It needs to be mentioned that the extra pure $ZnCl_2$ (1.0 M, 99.999% purity) was used to prepare **2b** in Table S2 to rule out the possible involvement of any transition metals. From the results shown in Table S2, it can be concluded that the halide ions, LiCl and $MgCl_2$ have no remarkable effect on this reaction.

Table S2. Testing the influence of organozinc reagents prepared by various methods

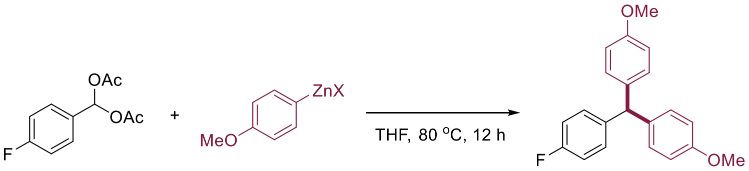

1c (1.0 mmol) + **2b** (3.0 equiv) → **3c**

Entry	**2b**[a]	Yield of **3c** (%)[b]
1	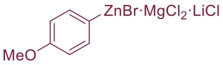	96 (93[c])
2	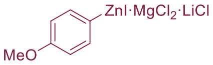	92
3	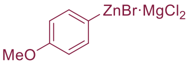	95
4	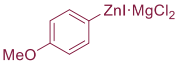	95
5	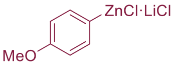	97

[a] Extra pure $ZnCl_2$ (1.0 M, 99.999% purity) was used to prepare **2b**.
[b] GC yield; Biphenyl was used as an internal standard. [c] Isolated yield.

Table S3. Testing the effect of $ZnCl_2$

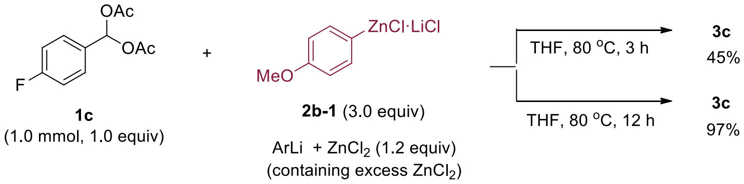

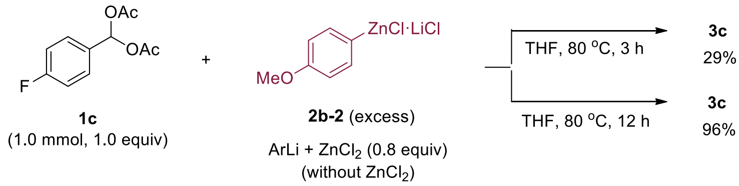

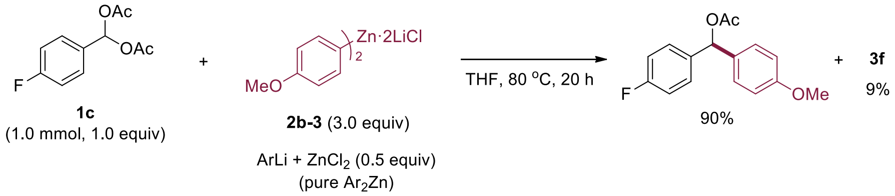

In order to study the effect of $ZnCl_2$, as shown in Table S3, we prepared three kinds of 4-$MeOC_6H_4ZnX$ (**2b-1**, **2b-2**, **2b-3**) by mixing 4-$MeOC_6H_4Li$ with different equivalents of $ZnCl_2$

(1.2 equiv, 0.8 equiv and 0.5 equiv relative to 4-MeOC_6H_4Li). By comparing the reaction results using **2b-1** and **2b-2**, excess $ZnCl_2$ should be able to accelerate the reaction to some extent, but not in a remarkable way, and have no effect on the final results. The pure Ar_2Zn (**2b-3**) is not reactive enough for double substitution.

Thus, the optimal organozinc reagents will be prepared by transmetalation of ArLi or ArMgX with $ZnCl_2$ (1.2 equiv relative to ArLi or ArMgX).

Table S4. Condition Screening for the synthesis of an unsymmetrical triarylmethane **5a**

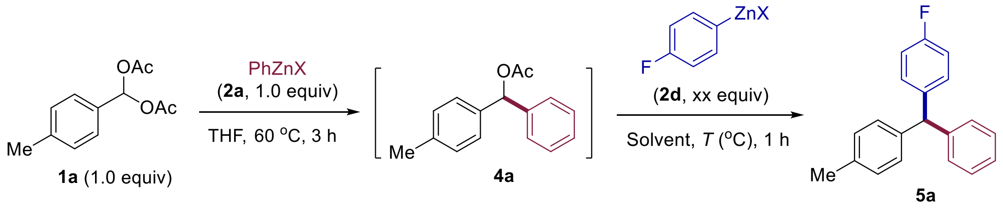

Entry	**2d**	T (°C)	Solvent	Yield of **5a** (%)[a]
1	1.5 equiv	80 °C	THF	54
2	1.5 equiv	25 °C	toluene	no reaction
3	1.5 equiv	60 °C	toluene	42
4	1.5 equiv	80 °C	toluene	94 (85[b])
5	2.0 equiv	80 °C	toluene	94

[a] GC yield; Biphenyl was used as an internal standard. [b] Isolated yield.

The optimal conditions for the synthesis of unsymmetrical triarylmethanes were also screened. As shown in Table S4, the optimal conditions for the first step have been determined in Table S1. However, when a second organozinc reagent such as *p*-fluorophenylzinc halide (**2d**) was added to the reaction mixture (**4a**) followed by heating at 80 °C for 1 h, **4a** was fully converted but the desired product **5a** was observed only in 54% yield (entry 1). To further optimize the reaction conditions for the second step, solvent and reaction temperature were mainly screened (Table S4). After addition of **2d** and subsequent removal of THF in vacuum, toluene was added and the reaction was first performed at 25 °C, but no reaction occurred (entry 2). A higher temperature at 60 °C gave **5a** in 42% yield (entry 3). Finally, heating the toluene solution at 80 °C for 1 h gave **5a** in satisfactory yield (entry 4).

4. General Procedures (GPs)

General Procedure 1 (GP1): Synthesis of symmetrical triarylmethanes (**3**)

A dry and argon-flushed 25 mL Schlenk-tube was equipped with a stirring bar and a septum. Arylzinc reagent (**2**, 3.0 mmol, 3.0 equiv) prepared by *Method A or B* was transferred to the Schlenk-tube. Then, benzal diacetate (**1**, 1.0 mmol, 1.0 equiv) was added in one portion at room temperature. The reaction mixture was stirred at 80 °C for 3-12 h and was finally quenched with NH_4Cl (aq.). The aqueous phase was extracted with ethyl acetate (3×10 mL) and the combined organic layers were dried over $MgSO_4$, filtered and concentrated under reduced pressure. Finally, the residue was purified by column chromatography using the indicated mixture of solvents as eluent to give the desired product of type **3**.

General Procedure 2 (GP2): One-pot synthesis of unsymmetrical triarylmethane derivatives (**5**)

A dry and argon-flushed 25 mL Schlenk-tube was equipped with a stirring bar and a septum. Ar^2ZnX (**2**, 1.0 mmol, 1.0 equiv) prepared by *Method A or B* was transferred to the Schlenk-tube. Benzal diacetate (**1**, 1.0 mmol, 1.0 equiv) was added in one portion at room temperature. The reaction mixture was stirred at room temperature for 12 h or at 60 °C for 3-12 h. This step was monitored by GC-MS analysis to achieve a full conversion. Then, after addition of a second different arylzinc reagent (Ar^3ZnX, 1.5 mmol, 1.5 equiv) and subsequent removal of THF in vacuum, toluene was added and the reaction mixture was heated typically at 80 °C for 1 h. The reaction mixture was finally quenched with NH_4Cl (aq.). The aqueous phase was extracted with ethyl acetate (3×10 mL) and the combined organic layers were dried over $MgSO_4$, filtered and concentrated under reduced pressure. Finally, the residue was purified by column chromatography using the indicated mixture of solvents as eluent to give the desired product of type **5**.

General Procedure 3 (GP3): One-pot synthesis of nonsymmetrical 1,1-diarylalkane derivatives (**6**)

A dry and argon-flushed 25 mL Schlenk-tube was equipped with a stirring bar and a septum. Ar^2ZnX (**2**, 1.0 mmol, 1.0 equiv) prepared by *Method A or B* was transferred to the Schlenk-tube. Benzal diacetate (**1**, 1.0 mmol, 1.0 equiv) was added in one portion at room temperature. The reaction mixture was stirred at room temperature for 12 h or at 60 °C for 3-12 h. This step

was monitored by GC-MS analysis to achieve a full conversion. Then, after addition of a second alkylzinc reagent (AlkylZnX, 1.5 mmol, 1.5 equiv) and subsequent removal of THF in vacuum, toluene was added and the reaction mixture was heated typically at 80 °C for 1 h. The reaction mixture was finally quenched with NH_4Cl (aq.). The aqueous phase was extracted with ethyl acetate (3×10 mL) and the combined organic layers were dried over $MgSO_4$, filtered and concentrated under reduced pressure. Finally, the residue was purified by column chromatography using the indicated mixture of solvents as eluent to give the desired product of type **6**.

General Procedure 4 (GP4): Synthesis of symmetrical 1,1-diarylalkane derivatives (**8**)

A dry and argon-flushed 25 mL Schlenk-tube was equipped with a stirring bar and a septum. Arylzinc reagent (**2**, 3.0 mmol, 3.0 equiv) prepared by *Method A or B* was transferred to the Schlenk-tube. Then, alkyl *gem*-diacetate (**7**, 1.0 mmol, 1.0 equiv) was added in one portion at room temperature. The reaction mixture was stirred at 80 °C for 3-12 h and was finally quenched with NH_4Cl (aq.). The aqueous phase was extracted with ethyl acetate (3×10 mL) and the combined organic layers were dried over $MgSO_4$, filtered and concentrated under reduced pressure. Finally, the residue was purified by column chromatography using the indicated mixture of solvents as eluent to give the desired product of type **8**.

5. Preparation and Characterization Data of Compounds

Preparation of 2-(phenylethynyl)benzal diacetate (1i):

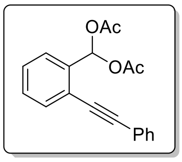

According to **2-1**, 2-(phenylethynyl)benzaldehyde (2.06 g, 10 mmol, 1.0 equiv) reacted with acetic anhydride (2.04 g, 20 mmol, 2 equiv) in the presence of *p*-TSA·H_2O (190 mg, 1 mmol, 10 mol%). The solution was stirred at room temperature for 3 h. After work-up, the product was obtained as an orange solid (93%, 2.86 g, 9.3 mmol). **M.p.:** 73-75ºC. **^{1}H-NMR (400 MHz, $CDCl_3$):** δ = 8.19 (s, 1H), 7.60−7.56 (m, 4H), 7.41−7.35 (m, 5H), 2.14 (s, 6H). **^{13}C-NMR (100 MHz, $CDCl_3$):** δ = 168.5, 136.8, 132.4, 131.7, 129.4, 128.6, 128.4, 128.4, 125.8, 122.7, 122.3, 95.2, 88.0, 85.6, 20.8. **IR (Diamond-ATR, neat):** $\tilde{\nu}$ / cm = 3060, 1741, 1592, 1492, 1368, 1237, 1198, 1055, 1006, 991, 949, 907, 758, 691. **MS (EI, 70 eV):** m/z (%) = 308 (5), 223 (16), 207 (30), 206 (100), 178 (22), 176 (12). **HRMS (EI, 70 eV)** m/z: calcd for [$C_{19}H_{16}O_4$] 308.1049, found 308.1046.

Preparation of 2-bromo-5-[(*tert*-butyldimethylsilyl)oxy]benzal diacetate (1n):

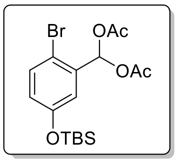

According to **2-1**, 2-bromo-5-[(tert-butyldimethylsilyl)oxy]benzaldehyde (3.15 g, 10 mmol, 1.0 equiv) reacted with acetic anhydride (2.04 g, 20 mmol, 2 equiv) in the presence of *p*-TSA·H_2O (190 mg, 1 mmol, 10 mol%). The solution was stirred at room temperature overnight. After work-up, the product was obtained as a colorless oil (95%, 3.96 g, 9.5 mmol). **^{1}H-NMR (400 MHz, $CDCl_3$):** δ = 7.80 (s, 1H), 7.38 (d, *J* = 8.8 Hz, 1H), 6.99 (d, *J* = 2.8 Hz, 1H), 6.72 (dd, *J* = 8.8, 2.8 Hz, 1H), 2.11 (s, 6H), 0.95 (s, 9H), 0.18 (s, 6H). **^{13}C-NMR (100 MHz, $CDCl_3$):** δ = 168.1, 155.1, 135.5, 133.7, 122.6, 119.4, 113.2, 88.7, 25.4, 20.5, 18.0, −4.6. **IR (Diamond-ATR, neat):** $\tilde{\nu}$ / cm = 2930, 2858, 1760, 1595, 1471, 1371, 1289, 1230, 1194, 1007, 937, 838, 780, 730. **MS (EI, 70 eV):** m/z (%) = 416 (7), 317 (14), 315 (14), 295 (29), 259 (19), 257 (17), 253 (24), 118 (22), 117 (100). **HRMS (EI, 70 eV)** m/z: calcd for [$C_{17}H_{25}BrO_5Si$] 416.0655, found 416.0646.

Preparation of 2-allylbenzal diacetate (1o):

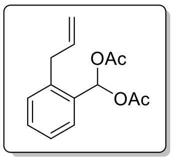

According to **2-1**, 2-allylbenzaldehyde (1.46 g, 10 mmol, 1.0 equiv) reacted with acetic anhydride (2.04 g, 20 mmol, 2 equiv) in the presence of *p*-TSA·H_2O (190 mg, 1 mmol, 10 mol%). The solution was stirred at room temperature overnight. After work-up, the product was obtained as a light yellow oil (94%, 2.33 g, 9.4 mmol). **^{1}H-NMR (400 MHz, $CDCl_3$):** δ = 7.83 (s, 1H), 7.55 (dd, *J* = 7.6, 1.6 Hz, 1H), 7.35 (td, *J* = 7.6, 1.6 Hz, 1H), 7.31−7.22 (m, 2H), 6.03−5.93 (m, 1H), 5.08−4.97 (m, 2H), 3.58 (d, *J* = 6.0 Hz, 2H), 2.10 (s, 6H). **^{13}C-NMR (100 MHz, $CDCl_3$):** δ = 168.6, 138.1, 136.8, 133.5,

130.3, 129.8, 127.3, 126.6, 116.0, 88.2, 36.6, 20.8. **IR (Diamond-ATR, neat):** $\tilde{\nu}$ / cm = 2979, 1750, 1637, 1431, 1369, 1237, 1197, 1053, 1006, 942, 911, 760, 732. **MS (EI, 70 eV):** m/z (%) = 248 (3), 189 (25), 146 (11), 145 (28), 131 (49), 128 (100), 117 (31), 115 (28). **HRMS (EI, 70 eV)** m/z: calcd for [$C_{14}H_{16}O_4$] 248.1049, found 248.1046.

Preparation of 2-methyldecane-1,1-diyl diacetate (7a):

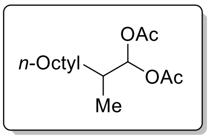

According to **2-2**, 2-methyldecanal (1.70 g, 10 mmol, 1.0 equiv) reacted with acetic anhydride (5.10 g, 50 mmol, 5 equiv) in the presence of I_2 (254 mg, 1 mmol, 10 mol%). The solution was stirred at room temperature overnight. After work-up, the product was obtained as a colorless oil (95%, 2.59 g, 9.5 mmol). **^{1}H-NMR (400 MHz, CDCl$_3$):** δ = 6.69 (d, *J* = 4.4 Hz, 1H), 2.08 (d, *J* = 1.2 Hz, 6H), 1.88−1.80 (m, 1H), 1.25 (br, 14H), 0.95 (d, *J* = 6.8 Hz, 3H), 0.88 (t, *J* = 7.2 Hz, 3H). **^{13}C-NMR (100 MHz, CDCl$_3$):** δ = 169.2, 169.1, 92.4, 36.4, 31.9, 30.8, 29.7, 29.5, 29.3, 26.8, 22.7, 20.9, 20.8, 14.1, 13.4. **IR (Diamond-ATR, neat):** $\tilde{\nu}$ / cm = 2924, 2854, 1763, 1711, 1466, 1374, 1240, 1206, 1044, 1007, 971, 722. **MS (EI, 70 eV):** m/z (%) = 272 (1), 213 (2), 170 (15), 111 (10), 97 (47), 43 (100). **HRMS (EI, 70 eV)** m/z: calcd for [$C_{15}H_{28}O_4$] 272.1988, found 272.1982.

Synthesis of phenyl(*p*-tolyl)methyl acetate (4a):

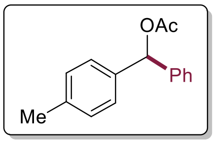

According to **GP2**, PhZnX (**2a**, 1.0 mmol, 1.0 equiv) reacted with *p*-methylbenzal diacetate (**1a**, 1.0 mmol, 1.0 equiv) at room temperature for 12 h or at 60 ºC for 3 h. After work-up, the residue was purified by column chromatography (SiO$_2$, *i*-hexane / ethyl acetate = 20 / 1) to give the desired product **4a** (86%, 206 mg, 0.86 mmol) as a colorless oil. **^{1}H-NMR (400 MHz, CDCl$_3$):** δ = 7.41−7.35 (m, 4H), 7.34−7.28 (m, 3H), 7.20 (d, *J* = 8.0 Hz, 2H), 6.92 (s, 1H), 2.38 (s, 3H), 2.20 (s, 3H). **^{13}C-NMR (100 MHz, CDCl$_3$):** δ = 170.0, 140.3, 137.6, 137.2, 129.1, 128.4, 127.7, 127.1, 126.9, 76.7, 21.2, 21.1. **IR (Diamond-ATR, neat):** $\tilde{\nu}$ / cm = 3030, 1753, 1605, 1501, 1421, 1246, 1189, 1112, 1070, 1020, 808, 754, 726, 680. **MS (EI, 70 eV):** m/z (%) = 240 (1), 197 (13), 180 (35), 179 (21), 165 (100). **HRMS (EI, 70 eV)** m/z: calcd for [$C_{16}H_{16}O_2$] 240.1150, found 240.1143.

Synthesis of (*p*-tolylmethylene)dibenzene (3a):

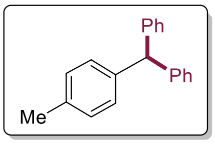

According to **GP1**, PhZnX (**2a**, 3.0 mmol, 3.0 equiv, by *Method A*) reacted with *p*-methylbenzal diacetate (**1a**, 1.0 mmol, 1.0 equiv) at 80 ºC for 6 h. After work-up, the residue was purified by column chromatography (SiO$_2$, *i*-hexane) to give the desired product **3a** (81%, 210 mg, 0.81 mmol) as a colorless oil. A scale-up of this reaction to 15 mmol gave **3a** in 80% yield (3.10 g, 12.0 mmol). **^{1}H-NMR (400 MHz, CDCl$_3$):** δ = 7.34−7.31 (m, 4H), 7.27−7.23 (m, 2H), 7.18−7.14 (m, 6H), 7.07−7.05 (m, 2H), 5.57

(s, 1H), 2.37 (s, 3H). **^{13}C-NMR (100 MHz, CDCl$_3$):** δ = 144.1, 140.9, 135.8, 129.4, 129.3, 129.0, 128.2, 126.2, 56.4, 21.0. **IR (Diamond-ATR, neat):** $\tilde{\nu}$ / cm = 3058, 3024, 2920, 1598, 1511, 1493, 1447, 1313, 1247, 1185, 1155, 1111, 1077, 1030, 1021, 1002, 916, 869, 832, 798, 749, 730, 696. **MS (EI, 70 eV):** m/z (%) = 258 (55), 244 (18), 243 (85), 207 (11), 181 (33), 179 (15), 166 (29), 165 (100). **HRMS (EI, 70 eV)** m/z: calcd for [$C_{20}H_{18}$] 258.1409, found 258.1405.

Synthesis of tris(4-methoxyphenyl)methane (3b):

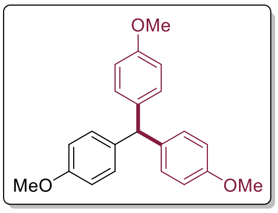

According to **GP1**, 4-MeOC$_6$H$_4$ZnX (**2b**, 3.0 mmol, 3.0 equiv, by *Method A*) reacted with *p*-anisylbenzal diacetate (**1b**, 1.0 mmol, 1.0 equiv) at 80 °C overnight. After work-up, the residue was purified by column chromatography (SiO$_2$, *i*-hexane / ethyl acetate = 20 / 1) to give the desired product **3b** (85%, 284 mg, 0.85 mmol) as an orange solid. **M.p.:** 47−48 °C. **^{1}H-NMR (400 MHz, CDCl$_3$):** δ = 7.05−7.02 (m, 6H), 6.86−6.82 (m, 6H), 5.42 (s, 1H), 3.80 (s, 9H). **^{13}C-NMR (100 MHz, CDCl$_3$):** δ = 157.8, 136.7, 130.2, 113.6, 55.2, 54.3. **IR (Diamond-ATR, neat):** $\tilde{\nu}$ / cm = 2832, 1606, 1583, 1503, 1463, 1441, 1300, 1241, 1181, 1170, 1107, 1036, 849, 827, 814, 774, 720. **MS (EI, 70 eV):** m/z (%) = 334 (61), 303 (100), 227 (68), 211 (34), 195 (22), 183 (13), 169 (15), 152 (13). **HRMS (EI, 70 eV)** m/z: calcd for [$C_{22}H_{12}O_3$] 334.1569, found 334.1562.

Synthesis of 4,4'-((4-fluorophenyl)methylene)bis(methoxybenzene) (3c):

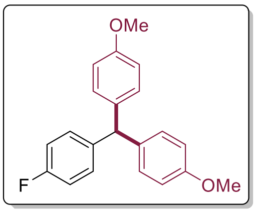

According to **GP1**, 4-MeOC$_6$H$_4$ZnX (**2b**, 3.0 mmol, 3.0 equiv, by *Method A*) reacted with *p*-fluorobenzal diacetate (**1c**, 1.0 mmol, 1.0 equiv) at 80 °C overnight. After work-up, the residue was purified by column chromatography (SiO$_2$, *i*-hexane / ethyl acetate = 20 / 1) to give the desired product **3c** (93%, 300 mg, 0.93 mmol) as a waxy oil. **^{1}H-NMR (400 MHz, CDCl$_3$):** δ = 7.08−7.03 (m, 2H), 7.02−6.93 (m, 6H), 6.85−6.81 (m, 4H), 5.44 (s, 1H), 3.79 (s, 6H). **^{13}C-NMR (100 MHz, CDCl$_3$):** δ = 161.3 (d, $^1J_{C\text{-}F}$ = 243 Hz), 158.0, 140.3 (d, $^4J_{C\text{-}F}$ = 3.2 Hz), 136.2, 130.6 (d, $^3J_{C\text{-}F}$ = 7.8 Hz), 130.2, 115.0 (d, $^2J_{C\text{-}F}$ = 21.0 Hz), 113.7, 55.2, 54.4. **^{19}F-NMR (376 MHz, CDCl$_3$):** δ = −117.2. **IR (Diamond-ATR, neat):** $\tilde{\nu}$ / cm = 3000, 2955, 2835, 1891, 1605, 1583, 1503, 1462, 1440, 1300, 1242,1220, 1174, 1156, 1109, 1032, 822, 811, 772, 721. **MS (EI, 70 eV):** m/z (%) = 322 (60), 292 (21), 291 (100), 227 (56), 215 (33), 199 (22), 183 (29), 171 (31). **HRMS (EI, 70 eV)** m/z: calcd for [$C_{21}H_{19}FO_2$] 322.1369, found 322.1361.

Synthesis of methyl 4-(bis(4-methoxyphenyl)methyl)benzoate (3d):

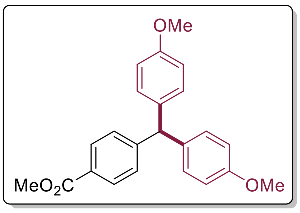

According to **GP1**, 4-$MeOC_6H_4ZnX$ (**2b**, 3.0 mmol, 3.0 equiv, by *Method A*) reacted with the corresponding benzal diacetate (**1d**, 1.0 mmol, 1.0 equiv) at 80 °C overnight. After work-up, the residue was purified by column chromatography (SiO_2, *i*-hexane / ethyl acetate = 10 / 1) to give the desired product **3d** (93%, 336 mg, 0.93 mmol) as a waxy oil. **^{1}H-NMR (400 MHz, $CDCl_3$):** δ = 7.96 (dd, *J* = 6.8, 1.6 Hz, 2H), 7.19 (d, *J* = 8.4 Hz, 2H), 7.03−6.99 (m, 4H), 6.86−6.82 (m, 4H), 5.50 (s, 1H), 3.90 (s, 3H), 3.79 (s, 6H). **^{13}C-NMR (100 MHz, $CDCl_3$):** δ = 167.0, 158.1, 150.0, 135.5, 130.2, 129.6, 129.3, 128.1, 113.7, 55.2, 55.1, 52.0. **IR (Diamond-ATR, neat):** $\tilde{\nu}$ / cm = 2999, 2951, 2835, 1717, 1608, 1582, 1507, 1461, 1434, 1410, 1276, 1242, 1174, 1108, 1031, 965, 817, 800, 784, 749, 701. **MS (EI, 70 eV):** m/z (%) = 362 (83), 347 (12), 332 (25), 331 (100), 303 (54), 255 (20), 227 (100), 195 (48), 165 (20). **HRMS (EI, 70 eV)** m/z: calcd for [$C_{23}H_{22}O_4$] 362.1518, found 362.1510.

Synthesis of 4-(bis(4-methoxyphenyl)methyl)benzonitrile (3e):

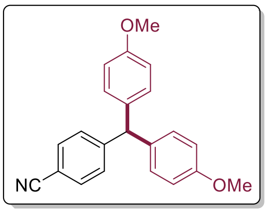

According to **GP1**, 4-$MeOC_6H_4ZnX$ (**2b**, 3.0 mmol, 3.0 equiv, by *Method A*) reacted with the 4-cyanobenzal diacetate (**1e**, 1.0 mmol, 1.0 equiv) at 80 °C overnight. After work-up, the residue was purified by column chromatography (SiO_2, *i*-hexane / ethyl acetate = 10 / 1) to give the desired product **3e** (95%, 312 mg, 0.95 mmol) as a waxy oil. **^{1}H-NMR (400 MHz, $CDCl_3$):** δ = 7.57 (d, *J* = 8.4 Hz, 2H), 7.22 (d, *J* = 8.4 Hz, 2H), 6.99−6.96 (m, 4H), 6.86−6.83 (m, 4H), 5.49 (s, 1H), 3.79 (s, 6H). **^{13}C-NMR (100 MHz, $CDCl_3$):** δ = 158.3, 150.2, 134.8, 132.0, 130.1, 130.0, 119.0, 113.9, 110.0, 55.2, 55.1. **IR (Diamond-ATR, neat):** $\tilde{\nu}$ / cm = 3000, 2932, 2835, 2226, 1606, 1582, 1507, 1461, 1408, 1301, 1242, 1174, 1111, 1030, 855, 822, 807, 772, 756, 723. **MS (EI, 70 eV):** m/z (%) = 329 (84), 298 (82), 227 (100), 190 (24), 178 (21). **HRMS (EI, 70 eV)** m/z: calcd for [$C_{22}H_{19}NO_2$] 329.1416, found 329.1408.

Synthesis of 2,2'-((4-bromophenyl)methylene)bis(methoxybenzene) (3f):

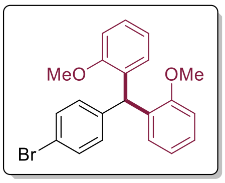

According to **GP1**, 2-$MeOC_6H_4ZnX$ (**2c**, 3.0 mmol, 3.0 equiv, by *Method A*) reacted with the 4-bromobenzal diacetate (**1f**, 1.0 mmol, 1.0 equiv) at 80 °C overnight. After work-up, the residue was purified by column chromatography (SiO_2, *i*-hexane / ethyl acetate = 15 / 1) to give the desired product **3f** (63%, 240 mg, 0.63 mmol) as a white solid. **M.p.:** 119−121 °C. **^{1}H-NMR (400 MHz, $CDCl_3$):** δ = 7.38−7.35 (m, 2H), 7.25−7.21 (m, 2H), 6.94 (d, *J* = 8.4 Hz, 2H), 6.89−6.84 (m, 4H), 6.78 (dd, *J* = 7.6, 1.6 Hz, 2H), 6.15 (s, 1H), 3.71 (s, 6H). **^{13}C-NMR (100 MHz, $CDCl_3$):** δ = 157.1, 143.1, 131.8, 131.0, 131.0, 129.9, 127.5, 120.1, 119.6, 110.7, 55.6, 42.7. **IR (Diamond-ATR, neat):** $\tilde{\nu}$ / cm = 2927, 1597, 1583, 1508, 1483, 1460, 1433, 1401, 1328, 1286, 1232, 1178,

1102, 1028, 1009, 835, 815, 803, 750, 736. **MS (EI, 70 eV):** m/z (%) = 384 (39), 382 (42), 353 (24), 351 (25), 303 (51), 195 (38), 165 (36), 152 (39), 121 (100). **HRMS (EI, 70 eV)** m/z: calcd for [$C_{21}H_{19}BrO_2$] 382.0568, found 382.0562.

Synthesis of 2-(bis(4-methoxyphenyl)methyl)thiophene (3g):

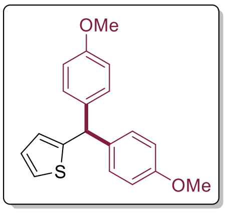

According to **GP1**, 4-MeOC$_6$H$_4$ZnX (**2b**, 3.0 mmol, 3.0 equiv, by *Method A*) reacted with thiophen-2-ylmethylene diacetate (**1g**, 1.0 mmol, 1.0 equiv) at room temperature for 3 h. After work-up, the residue was purified by column chromatography (SiO_2, *i*-hexane / ethyl acetate = 50 / 1) to give the desired product **3g** (92%, 284 mg, 0.92 mmol) as a purple solid. **M.p.:** 76−78 °C. **^{1}H-NMR (400 MHz, CDCl$_3$):** δ = 7.21 (dd, *J* = 5.0, 1.2 Hz, 1H), 7.14 (d, *J* = 8.8 Hz, 4H), 6.94 (dd, *J* = 5.2, 3.6 Hz, 1H), 6.87−6.84 (m, 4H), 6.69 (d, *J* = 3.6 Hz, 1H), 5.60 (s, 1H), 3.80 (s, 6H). **^{13}C-NMR (100 MHz, CDCl$_3$):** δ = 158.2, 148.9, 136.3, 129.7, 126.5, 126.0, 124.3, 113.6, 55.2, 50.5. **IR (Diamond-ATR, neat):** $\tilde{\nu}$ / cm = 2998, 2931, 2834, 1606, 1582, 1506, 1461, 1439, 1301, 1241, 1173, 1109, 1030, 811, 789, 760, 695. **MS (EI, 70 eV):** m/z (%) = 310 (100), 279 (51), 203 (54), 187 (49). **HRMS (EI, 70 eV)** m/z: calcd for [$C_{19}H_{18}O_2S$] 310.1028, found 310.1021.

Synthesis of 1,4-bis(di(thiophen-2-yl)methyl)benzene (3h):

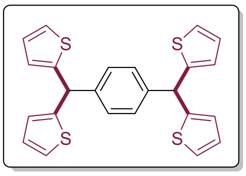

According to **GP1**, thiophen-2-ylzinc halide (3.0 mmol, 6.0 equiv, by *Method A*) reacted with 1,4-phenylenebis(methanetriyl) tetraacetate (**1h**, 0.5 mmol, 1.0 equiv) at 80 °C overnight. After work-up, the residue was purified by column chromatography (SiO_2, *i*-hexane) to give the desired product **3h** (74%, 160 mg, 0.37 mmol) as a pale solid. **M.p.:** 116−117 °C. **^{1}H-NMR (400 MHz, CDCl$_3$):** δ = 7.25−7.20 (m, 8H), 6.96−6.92 (m, 8H), 5.86 (s, 2H). **^{13}C-NMR (100 MHz, CDCl$_3$):** δ = 147.5, 142.4, 128.5, 126.5, 126.0, 124.6, 47.0. **IR (Diamond-ATR, neat):** $\tilde{\nu}$ / cm = 2992, 1505, 1413, 1227, 1104, 1074, 1034, 1017, 827, 773, 695. **MS (EI, 70 eV):** m/z (%) = 434 (100), 221 (11), 179 (54). **HRMS (EI, 70 eV)** m/z: calcd for [$C_{24}H_{18}S_4$] 434.0291, found 434.0293. The analytical data and spectra are identical with the literature.[5]

Synthesis of 4,4'-(p-tolylmethylene)bis(fluorobenzene) (3i):

[5] K. Singh, S. Sharma, A. Sharma, *J. Mol. Catal. A: Chem.* **2011**, *347*, 34−37.

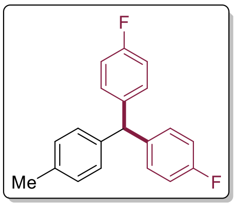

According to **GP1**, *p*-fluorophenylzinc halide (**2d**, 3.0 mmol, 3.0 equiv, by *Method A*) reacted with *p*-methylbenzal diacetate (**1a**, 1.0 mmol, 1.0 equiv) at 80 °C for 12 h. After work-up, the residue was purified by column chromatography (SiO_2, *i*-hexane) to give the desired product **3i** (54%, 159 mg, 0.54 mmol) as a colorless oil. When this reaction was performed in toluene at 80 °C for 1 h, the desired product **3i** was finally obtained in 76% (223 mg, 0.76 mmol) yield. **^{1}H-NMR (400 MHz, $CDCl_3$):** δ = 7.12 (d, *J* = 7.6 Hz, 2H), 7.08−7.03 (m, 4H), 7.01−6.95 (m, 6H), 5.48 (s, 1H), 2.34 (s, 3H). **^{13}C-NMR (100 MHz, $CDCl_3$):** δ = 161.4 (d, $^1J_{C\text{-}F}$ = 243 Hz), 140.5, 139.6 (d, $^4J_{C\text{-}F}$ = 3.2 Hz), 136.2, 130.7 (d, $^3J_{C\text{-}F}$ = 7.9 Hz), 129.1 (d, $^3J_{C\text{-}F}$ = 8.2 Hz), 115.1 (d, $^2J_{C\text{-}F}$ = 21.1 Hz), 54.8, 21.0. **^{19}F-NMR (376 MHz, $CDCl_3$):** δ = −116.7. **IR (Diamond-ATR, neat):** $\tilde{\nu}$ / cm = 3045, 2922, 1896, 1602, 1505, 1408, 1221, 1157, 1096, 1015, 855, 816, 767, 717. **MS (EI, 70 eV):** m/z (%) = 294 (31), 280 (21), 279 (100), 201 (26), 183 (84). **HRMS (EI, 70 eV)** m/z: calcd for [$C_{20}H_{16}F_2$] 294.1220, found 294.1214.

Synthesis of 4,4'-((4-methoxyphenyl)methylene)bis(fluorobenzene) (3j):

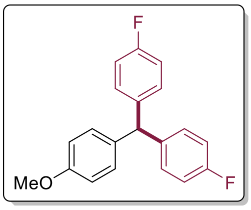

According to **GP1**, *p*-fluorophenylzinc halide (**2d**, 3.0 mmol, 3.0 equiv, by *Method A*) reacted with *p*-methoxybenzal diacetate (**1b**, 1.0 mmol, 1.0 equiv) at 80 °C for 12 h. After work-up, the residue was purified by column chromatography (SiO_2, *i*-hexane / ethyl acetate = 50 / 1) to give the desired product **3j** (65%, 202 mg, 0.65 mmol) as a waxy oil. When this reaction was performed in toluene at 80 °C for 1 h, the desired product **3j** was finally obtained in 84% (260 mg, 0.84 mmol) yield. Besides, a scale-up of this reaction (in toluene) to 10 mmol gave **3j** in 82% (2.54 g, 8.2 mmol) yield. **^{1}H-NMR (400 MHz, $CDCl_3$):** δ = 7.07−7.03 (m, 4H), 7.01−6.95 (m, 6H), 6.87−6.83 (m, 2H), 5.47 (s, 1H), 3.80 (s, 3H). **^{13}C-NMR (100 MHz, $CDCl_3$):** δ = 161.4 (d, $^1J_{C\text{-}F}$ = 244 Hz), 158.2, 139.8 (d, $^4J_{C\text{-}F}$ = 3.2 Hz), 135.6, 130.6 (d, $^3J_{C\text{-}F}$ = 7.8 Hz), 130.1, 115.1 (d, $^2J_{C\text{-}F}$ = 21.1 Hz), 113.8, 55.2, 54.4. **^{19}F-NMR (376 MHz, $CDCl_3$):** δ = −116.7. **IR (Diamond-ATR, neat):** $\tilde{\nu}$ / cm = 3001, 2931, 2836, 1893, 1601, 1583, 1503, 1462, 1300, 1246, 1220, 1177, 1156, 1096, 1034, 1014, 855, 823, 772, 716. **MS (EI, 70 eV):** m/z (%) = 310 (51), 279 (50), 215 (100), 201 (56), 183 (71), 171 (42). **HRMS (EI, 70 eV)** m/z: calcd for [$C_{20}H_{16}F_2O$] 310.1169, found 310.1162.

Synthesis of tris(4-fluorophenyl)methane (3k):

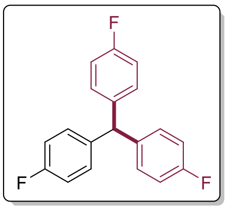

According to **GP1**, *p*-fluorophenylzinc halide (**2d**, 3.0 mmol, 3.0 equiv, by *Method A*) reacted with *p*-fluorobenzal diacetate (**1c**, 1.0 mmol, 1.0 equiv) at 80 °C for 12 h. After work-up, the residue was purified by column chromatography (SiO_2, *i*-hexane) to give the desired product **3k** (42%, 125

mg, 0.42 mmol) as a colorless oil. When this reaction was performed in toluene at 80 °C for 1 h, the desired product **3k** was finally obtained in 72% (214 mg, 0.72 mmol) yield. **^{1}H-NMR (400 MHz, $CDCl_3$):** δ = 7.07−6.97 (m, 12H), 5.51 (s, 1H). **^{13}C-NMR (100 MHz, $CDCl_3$):** δ = 161.5 (d, $^1J_{C\text{-}F}$ = 244 Hz), 139.2 (d, $^4J_{C\text{-}F}$ = 3.2 Hz), 130.6 (d, $^3J_{C\text{-}F}$ = 7.9 Hz), 115.3 (d, $^2J_{C\text{-}F}$ = 21.1 Hz), 54.4. **^{19}F-NMR (376 MHz, $CDCl_3$):** δ = −116.3. **IR (Diamond-ATR, neat):** $\tilde{\nu}$ / cm = 3042, 1895, 1601, 1504, 1416, 1301, 1220, 1157, 1098, 1015, 858, 822, 775, 717. **MS (EI, 70 eV):** m/z (%) = 298 (54), 203 (51), 201 (100), 183 (38). **HRMS (EI, 70 eV)** m/z: calcd for [$C_{19}H_{13}F_3$] 298.0969, found 298.0966.

Synthesis of 4,4'-((4-methoxyphenyl)methylene)bis((trifluoromethyl)benzene) (3l):

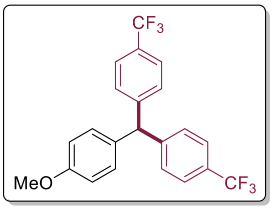

According to **GP1**, 4-$CF_3C_6H_4$ZnX (**2e**, 3.0 mmol, 3.0 equiv, by *Method A*) was mixed with *p*-methoxybenzal diacetate (**1b**, 1.0 mmol, 1.0 equiv) in THF. Then, THF was removed in vacuum and toluene was added. The toluene solution was heated at 80 °C for 1 h. After work-up, the residue was purified by column chromatography (SiO_2, *i*-hexane / ethyl acetate = 50 / 1) to give the desired product **3l** (80%, 328 mg, 0.80 mmol) as a waxy oil. **^{1}H-NMR (400 MHz, $CDCl_3$):** δ = 7.57 (d, *J* = 8.0 Hz, 4H), 7.23 (d, *J* = 8.0 Hz, 4H), 7.01 (d, *J* = 8.4 Hz, 2H), 6.90−6.87 (m, 2H), 5.62 (s, 1H), 3.81 (s, 3H). **^{13}C-NMR (100 MHz, $CDCl_3$):** δ = 158.5, 147.4, 134.0, 130.2, 129.6, 128.9 (q, $^2J_{C\text{-}F}$ = 32.0 Hz), 125.4 (q, $^3J_{C\text{-}F}$ = 4.0 Hz), 124.1 (q, $^1J_{C\text{-}F}$ = 270 Hz), 114.0, 55.6, 55.2. **^{19}F-NMR (376 MHz, $CDCl_3$):** δ = −62.5. **IR (Diamond-ATR, neat):** $\tilde{\nu}$ / cm = 3004, 2936, 2838, 1615, 1584, 1510, 1412, 1320, 1248, 1160, 1107, 1065, 1034, 1017, 860, 824, 803, 766. **MS (EI, 70 eV):** m/z (%) = 410 (88), 391 (14), 341 (45), 265 (100), 233 (41), 195 (18). **HRMS (EI, 70 eV)** m/z: calcd for [$C_{22}H_{16}F_6O$] 410.1105, found 410.1102.

Synthesis of (2-(phenylethynyl)phenyl)methylene)dibenzene (3m):

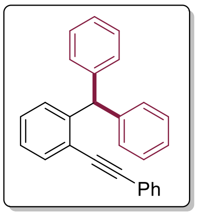

According to **GP1**, PhZnX (**2a**, 3.0 mmol, 3.0 equiv, by *Method A*) was mixed with (2-(phenylethynyl)phenyl)methylene diacetate (**1i**, 1.0 mmol, 1.0 equiv) in THF. Then, THF was removed in vacuum and toluene was added. The toluene solution was heated at 120 °C for 1 h. After work-up, the residue was purified by column chromatography (SiO_2, *i*-hexane) to give the desired product **3m** (98%, 338 mg, 0.98 mmol) as a pale solid. **M.p.:** 136−138 °C. **^{1}H-NMR (400 MHz, $CDCl_3$):** δ = 7.58 (dd, *J* = 7.0, 2.0 Hz, 1H), 7.40−7.37 (m, 2H), 7.34−7.29 (m, 7H), 7.28−7.22 (m, 4H), 7.18−7.16 (m, 4H), 7.02−7.00 (m, 1H), 6.18 (s, 1H). **^{13}C-NMR (100 MHz, $CDCl_3$):** δ = 145.9, 143.2, 132.4, 131.4, 129.6, 129.4, 128.2, 128.2, 128.2, 126.3, 123.5, 123.2, 94.1, 88.2, 54.6. **IR (Diamond-ATR, neat):** $\tilde{\nu}$ / cm = 3055, 3024, 2920, 1598, 1492, 1473, 1450, 1344,

1308, 1158, 1080, 1030, 922, 758, 729, 704, 694. **MS (EI, 70 eV):** m/z (%) = 344 (100), 267 (64), 252 (45), 165 (19). **HRMS (EI, 70 eV)** m/z: calcd for [$C_{27}H_{20}$] 344.1565, found 344.1556.

Synthesis of 9-phenyl-9*H*-fluorene (3n):

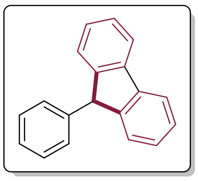

According to **GP1**, 2,2'-*bis*-zincated biphenyl (1.5 mmol, 1.5 equiv, by *Method A or by adding $ZnCl_2$* into the corresponding cyclometalated lanthanum reagents[6]) reacted with benzal diacetate (**1j**, 1.0 mmol, 1.0 equiv) at 80 °C overnight. After work-up, the residue was purified by column chromatography (SiO_2, *i*-hexane) to give the desired product **3n** (72%, 174 mg, 0.72 mmol) as a white solid. **M.p.:** 149−150 °C. **^{1}H-NMR (400 MHz, CDCl$_3$):** δ = 7.82 (d, *J* = 7.6 Hz, 2H), 7.40 (t, *J* = 7.4 Hz, 2H), 7.34−7.22 (m, 7H), 7.12−7.10 (m, 2H), 5.06 (s, 1H). **^{13}C-NMR (100 MHz, CDCl$_3$):** δ = 147.9, 141.6, 141.0, 128.7, 128.3, 127.3, 126.8, 125.3, 119.8, 54.4. **IR (Diamond-ATR, neat):** $\tilde{\nu}$ / cm = 3050, 2032, 1992, 1491, 1445, 1030, 752, 738, 697, 667, 658. **MS (EI, 70 eV):** m/z (%) = 242 (100), 239 (45), 226 (10), 165 (24), 120 (11). **HRMS (EI, 70 eV)** m/z: calcd for [$C_{19}H_{14}$] 242.1096, found 242.1087.

Synthesis of 9-(4-methoxyphenyl)-9*H*-fluorene (3o):

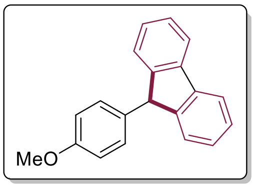

According to **GP1**, 2,2'-*bis*-zincated biphenyl (1.5 mmol, 1.5 equiv, by *Method A or by adding $ZnCl_2$* into the corresponding cyclometalated lanthanum reagents[6]) reacted with *p*-methoxybenzal diacetate (**1b**, 1.0 mmol, 1.0 equiv) at 80 °C overnight. After work-up, the residue was purified by column chromatography (SiO_2, *i*-hexane / ethyl acetate = 50 / 1) to give the desired product **3n** (85%, 231 mg, 0.85 mmol) as a white solid. **M.p.:** 121−122 °C. **^{1}H-NMR (400 MHz, CDCl$_3$):** δ = 7.85 (d, *J* = 7.6 Hz, 2H), 7.42 (t, *J* = 7.4 Hz, 2H), 7.36 (d, *J* = 7.0 Hz, 2H), 7.30 (td, *J* = 7.4, 0.8 Hz, 2H), 7.08−7.04 (m, 2H), 6.88−6.84 (m, 2H), 5.05 (s, 1H), 3.81 (s, 3H). **^{13}C-NMR (100 MHz, CDCl$_3$):** δ = 158.4, 148.1, 140.8, 133.4, 129.2, 127.2, 127.2, 125.2, 119.8, 114.0, 55.1, 53.6. **IR (Diamond-ATR, neat):** $\tilde{\nu}$ / cm = 3043, 2921, 1609, 1509, 1445, 1248, 1174, 1104, 1029, 850, 808, 789, 736. **MS (EI, 70 eV):** m/z (%) = 272 (100), 257 (34), 241 (44), 228 (25), 202 (15), 165 (10). **HRMS (EI, 70 eV)** m/z: calcd for [$C_{20}H_{16}O$] 272.1201, found 272.1195.

Synthesis of 1-fluoro-4-(phenyl(p-tolyl)methyl)benzene (5a):

[6] B. Wei, D. Zhang, Y.-H. Chen, A. Lei, P. Knochel, *Angew. Chem. Int. Ed.* **2019**, *58*, 15631−15635; *Angew. Chem.* **2019**, *131*, 15777−15782.

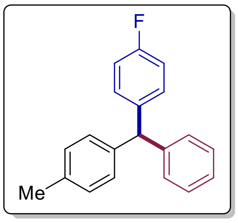

According to **GP2**, *p*-methylbenzal diacetate (**1a**, 1.0 mmol, 1.0 equiv) reacted with PhZnX (**2a**, 1.0 mmol, 1.0 equiv, by *Method A*) in THF at 60 °C for 3 h. Then, after addition of *p*-fluorophenylzinc halide (**2d**, 1.5 mmol, 1.5 equiv, by *Method A*) and subsequent removal of THF in vacuum, toluene was added and the reaction mixture was heated at 80 °C for 1 h. After work-up, the residue was purified by column chromatography (SiO_2, *i*-hexane) to give the desired product **5a** (85%, 234 mg, 0.85 mmol) as a colorless oil. **^{1}H-NMR (400 MHz, CDCl$_3$):** δ = 7.33−7.29 (m, 2H), 7.26−7.22 (m, 1H), 7.14−7.07 (m, 6H), 7.02−6.96 (m, 4H), 5.52 (s, 1H), 2.35 (s, 3H). **^{13}C-NMR (100 MHz, CDCl$_3$):** δ = 161.3 (d, $^{1}J_{C-F}$ = 243 Hz), 143.9, 140.7, 139.8 (d, $^{4}J_{C-F}$ = 3.2 Hz), 136.0, 130.8 (d, $^{3}J_{C-F}$ = 7.8 Hz), 129.3, 129.2, 129.1, 128.3, 126.3, 115.0 (d, $^{2}J_{C-F}$ = 21.1 Hz), 55.6, 21.0. **^{19}F-NMR (376 MHz, CDCl$_3$):** δ = −117.0. **IR (Diamond-ATR, neat):** $\tilde{\nu}$ / cm = 3024, 2920, 2871, 1897, 1600, 1504, 1450, 1410, 1221, 1156, 1095, 1016, 917, 872, 814, 769, 736, 697. **MS (EI, 70 eV):** m/z (%) = 276 (63), 261 (100), 199 (28), 183 (90), 179 (23), 165 (66). **HRMS (EI, 70 eV)** m/z: calcd for [$C_{20}H_{17}F$] 276.1314, found 276.1310.

Synthesis of 4-((4-fluorophenyl)(4-methoxyphenyl)methyl)benzonitrile (5b):

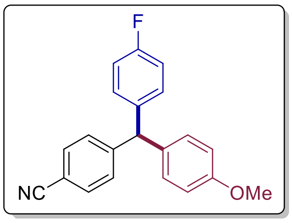

According to **GP2**, 4-cyanobenzal diacetate (**1e**, 1.0 mmol, 1.0 equiv) reacted with 4-MeOC$_6$H$_4$ZnX (**2b**, 1.0 mmol, 1.0 equiv, by *Method A*) in THF at room temperature overnight. Then, after addition of *p*-fluorophenylzinc halide (**2d**, 1.5 mmol, 1.5 equiv, by *Method A*) and subsequent removal of THF in vacuum, toluene was added and the reaction mixture was heated at 80 °C for 1 h. After work-up, the residue was purified by column chromatography (SiO_2, *i*-hexane / ethyl acetate = 10 / 1) to give the desired product **5b** (85%, 234 mg, 0.85 mmol) as a waxy oil. A scale-up of this reaction to 10 mmol gave **5b** in 83% (2.63 g, 8.3 mmol) yield. **^{1}H-NMR (400 MHz, CDCl$_3$):** δ = 7.58 (dt, *J* = 8.4, 1.6 Hz, 2H), 7.20 (d, *J* = 8.0 Hz, 2H), 7.05−6.95 (m, 6H), 6.87−6.83 (m, 2H), 5.52 (s, 1H), 3.79 (s, 3H). **^{13}C-NMR (100 MHz, CDCl$_3$):** δ = 161.6 (d, $^{1}J_{C-F}$ = 244 Hz), 158.5, 149.6, 138.5 (d, $^{4}J_{C-F}$ = 3.2 Hz), 134.3, 132.2, 130.7 (d, $^{3}J_{C-F}$ = 7.9 Hz), 130.2, 130.0, 118.8, 115.4 (d, $^{2}J_{C-F}$ = 21.2 Hz), 114.0, 110.3, 55.2, 55.2. **^{19}F-NMR (376 MHz, CDCl$_3$):** δ = −115.9. **IR (Diamond-ATR, neat):** $\tilde{\nu}$ / cm = 3001, 2933, 2836, 2227, 1606, 1582, 1504, 1462, 1407, 1302, 1246, 1222, 1177, 1157, 1111, 1032, 857, 823, 773, 723. **MS (EI, 70 eV):** m/z (%) = 317 (100), 215 (51), 141 (16), 125 (24), 99 (31), 85 (40). **HRMS (EI, 70 eV)** m/z: calcd for [$C_{21}H_{16}FNO$] 317.1216, found 317.1211.

Synthesis of 1-fluoro-4-((4-methoxyphenyl)(4-(trifluoromethyl)phenyl)methyl)benzene (5c):

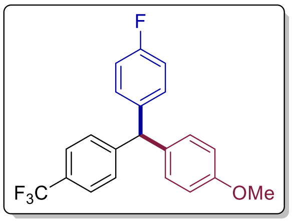

According to **GP2**, 4-trifluoromethylbenzal diacetate (**1k**, 1.0 mmol, 1.0 equiv) reacted with 4-$MeOC_6H_4ZnX$ (**2b**, 1.0 mmol, 1.0 equiv, by *Method A*) in THF at room temperature overnight. Then, after addition of *p*-fluorophenylzinc halide (**2d**, 1.5 mmol, 1.5 equiv, by *Method A*) and subsequent removal of THF in vacuum, toluene was added and the reaction mixture was heated at 80 °C for 1 h. After work-up, the residue was purified by column chromatography (SiO_2, *i*-hexane / ethyl acetate = 50 / 1) to give the desired product **5c** (72%, 260 mg, 0.72 mmol) as a waxy oil. **^{1}H-NMR (400 MHz, $CDCl_3$):** δ = 7.54 (d, *J* = 8.0 Hz, 2H), 7.21 (d, *J* = 8.4 Hz, 2H), 7.07−6.97 (m, 6H), 6.87−6.83 (m, 2H), 5.53 (s, 1H), 3.80 (s, 3H). **^{13}C-NMR (100 MHz, $CDCl_3$):** δ = 161.5 (d, $^1J_{C\text{-}F}$= 244 Hz), 158.3, 148.2, 139.0 (d, $^4J_{C\text{-}F}$= 3.2 Hz), 134.8, 130.7 (d, $^3J_{C\text{-}F}$ = 7.8 Hz), 130.2, 129.6, 128.7 (q, $^2J_{C\text{-}F}$ = 32.1 Hz), 125.3 (q, $^3J_{C\text{-}F}$ = 3.7 Hz), 124.2 (q, $^1J_{C\text{-}F}$ = 270 Hz), 115.3 (d, $^2J_{C\text{-}F}$ = 21.1 Hz), 113.9, 55.2, 55.0. **^{19}F-NMR (376 MHz, $CDCl_3$):** δ = −62.4, −116.3. **IR (Diamond-ATR, neat):** $\tilde{\nu}$ / cm = 3039, 2958, 2838, 1893, 1618, 1606, 1506, 1464, 1442, 1418, 1323, 1247, 1224, 1158, 1120, 1066, 1034, 1017, 957, 858, 824, 766, 704, 664. **MS (EI, 70 eV):** m/z (%) = 360 (72), 329 (26), 265 (24), 215 (100), 183 (74), 171 (26). **HRMS (EI, 70 eV)** m/z: calcd for [$C_{21}H_{16}F_4O$] 360.1137, found 360.1132.

Synthesis of 4-(benzo[d][1,3]dioxol-5-yl(4-fluorophenyl)methyl)benzonitrile (5d):

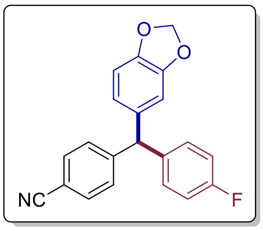

According to **GP2**, 4-cyanobenzal diacetate (**1e**, 1.0 mmol, 1.0 equiv) reacted with *p*-fluorophenylzinc halide (**2d**, 1.0 mmol, 1.0 equiv, by *Method A*) in THF at room temperature for 12 h. Then, after addition of benzo[d][1,3]dioxol-5-ylzinc halide (1.5 mmol, 1.5 equiv, by *Method A*) and subsequent removal of THF in vacuum, toluene was added and the reaction mixture was heated at 80 °C for 1 h. After work-up, the residue was purified by column chromatography (SiO_2, *i*-hexane / ethyl acetate = 20 / 1) to give the desired product **5d** (90%, 298 mg, 0.90 mmol) as a waxy oil. **^{1}H-NMR (400 MHz, $CDCl_3$):** δ = 7.58 (d, *J* = 8.4 Hz, 2H), 7.20 (d, *J* = 8.0 Hz, 2H), 7.05−6.97 (m, 4H), 6.75 (d, *J* = 8.0 Hz, 1H), 6.53−6.49 (m, 2H), 5.95 (s, 2H), 5.48 (s, 1H). **^{13}C-NMR (100 MHz, $CDCl_3$):** δ = 161.6 (d, $^1J_{C\text{-}F}$ = 244 Hz), 149.3, 147.9, 146.5, 138.2 (d, $^4J_{C\text{-}F}$ = 3.3 Hz), 136.0, 132.2, 130.6 (d, $^3J_{C\text{-}F}$ = 7.9 Hz), 129.9, 122.4, 118.8, 115.4 (d, $^2J_{C\text{-}F}$ = 21.2 Hz), 110.4, 109.5, 108.2, 101.1, 55.5. **^{19}F-NMR (376 MHz, $CDCl_3$):** δ = −115.7. **IR (Diamond-ATR, neat):** $\tilde{\nu}$ / cm = 3036, 2891, 2778, 2227, 1605, 1501, 1484, 1439, 1408, 1363, 1222, 1158, 1118, 1093, 1036, 928, 844, 824, 810, 776, 754, 727. **MS (EI, 70 eV):** m/z (%) = 331 (100), 300 (30), 236 (17), 229 (50), 199 (29), 170 (41). **HRMS (EI, 70 eV)** m/z: calcd for [$C_{21}H_{14}FNO_2$] 331.1009, found 331.0999.

Synthesis of ((5-((4-bromophenyl)(4-fluorophenyl)methyl)-2-methoxybenzyl)oxy)(tert-butyl)dimethylsilane (5e):

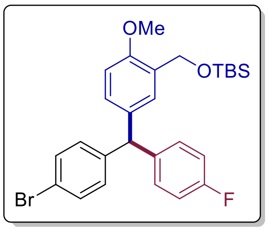

According to **GP2**, 4-bromobenzal diacetate (**1f**, 1.0 mmol, 1.0 equiv) reacted with *p*-fluorophenylzinc halide (**2d**, 1.0 mmol, 1.0 equiv, by *Method A*) in THF at room temperature for 12 h. Then, after addition of (3-(((tert-butyldimethylsilyl)oxy)methyl)-4-methoxyphenyl)zinc halide (1.5 mmol, 1.5 equiv, by *Method A*) and subsequent removal of THF in vacuum, toluene was added and the reaction mixture was heated at 80 °C for 1 h. After work-up, the residue was purified by column chromatography (SiO_2, *i*-hexane / ethyl acetate = 50 / 1) to give the desired product **5e** (59%, 303 mg, 0.59 mmol) as a waxy oil. **^{1}H-NMR (400 MHz, $CDCl_3$):** δ = 7.39 (d, *J* = 8.4 Hz, 2H), 7.14 (s, 1H), 7.06−7.03 (m, 2H), 6.98−6.91 (m, 5H), 6.74 (d, *J* = 8.4 Hz, 1H), 5.44 (s, 1H), 4.71 (s, 2H), 3.80 (s, 3H), 0.84 (s, 9H), 0.0 (s, 6H). **^{13}C-NMR (100 MHz, $CDCl_3$):** δ = 161.4 (d, $^1J_{C\text{-}F}$ = 243 Hz), 154.5, 143.3, 139.5 (d, $^4J_{C\text{-}F}$ = 3.2 Hz), 135.0, 131.4, 131.0, 130.7 (d, $^3J_{C\text{-}F}$ = 7.8 Hz), 129.8, 128.0, 127.3, 120.2, 115.1 (d, $^2J_{C\text{-}F}$ = 21.1 Hz), 109.3, 59.9, 55.2, 54.8, 25.8, 18.2, −5.4. **^{19}F-NMR (376 MHz, $CDCl_3$):** δ = −116.9. **IR (Diamond-ATR, neat):** $\tilde{\nu}$ / cm = 2953, 2926, 2854, 1603, 1506, 1497, 1462, 1376, 1291, 1249, 1223, 1181, 1158, 1132, 1080, 1032, 1010, 908, 834, 814, 774, 667. **MS (EI, 70 eV):** m/z (%) = 514 (8), 459 (100), 363 (46), 287 (18), 262 (66), 183 (80). **HRMS (EI, 70 eV)** m/z: calcd for [$C_{27}H_{32}BrFO_2Si$] 514.1339, found 514.1333.

Synthesis of 2-(4-((4-bromophenyl)(4-methoxyphenyl)methyl)phenyl)-1,3-dioxolane (5f):

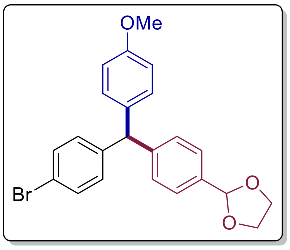

According to **GP2**, 4-bromobenzal diacetate (**1f**, 1.0 mmol, 1.0 equiv) reacted with 4-(1,3-dioxolan-2-yl)phenylzinc halide (1.0 mmol, 1.0 equiv, by *Method A*) in THF at 60 °C overnight. Then, after addition of 4-$MeOC_6H_4ZnX$ (**2b**, 1.5 mmol, 1.5 equiv, by *Method A*) and subsequent removal of THF in vacuum, toluene was added and the reaction mixture was heated at 80 °C for 1 h. After work-up, the residue was purified by column chromatography (SiO_2, *i*-hexane / ethyl acetate = 15 / 1) to give the desired product **5f** (66%, 280 mg, 0.66 mmol) as a waxy oil. **^{1}H-NMR (400 MHz, $CDCl_3$):** δ = 7.43−7.38 (m, 4H), 7.11 (d, *J* = 8.0 Hz, 2H), 7.00−6.97 (m, 4H), 6.85−6.81 (m, 2H), 5.79 (s, 1H), 5.47 (s, 1H), 4.18−4.00 (m, 4H), 3.79 (s, 3H). **^{13}C-NMR (100 MHz, $CDCl_3$):** δ = 158.1, 144.7, 143.1, 135.9, 135.1, 131.3, 131.0, 130.2, 129.3, 126.5, 120.2, 113.7, 103.5, 65.3, 55.2, 55.1. **IR (Diamond-ATR, neat):** $\tilde{\nu}$ / cm = 2951, 2881, 2833, 1608, 1581, 1508, 1484, 1462, 1393, 1300, 1245, 1176, 1072, 1031, 1009, 967, 941, 811, 768, 719. **MS (EI, 70 eV):** m/z (%) = 424 (49), 379 (20), 351 (28), 273 (57), 195 (44), 165 (100). **HRMS (EI, 70 eV)** m/z: calcd for [$C_{23}H_{21}BrO_3$] 424.0674, found 424.0669.

Synthesis of methyl 4-((4-(methylthio)phenyl)(3-(trimethylsilyl)phenyl)methyl)benzoate (5g):

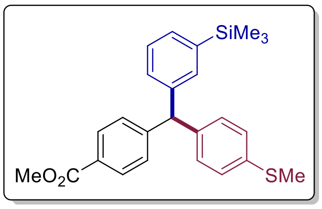

According to **GP2**, (4-(methoxycarbonyl)phenyl)methylene diacetate (**1d**, 1.0 mmol, 1.0 equiv) reacted with 4-(methylthio)phenylzinc halide (1.0 mmol, 1.0 equiv, by *Method A*) in THF at 60 °C overnight. Then, after addition of 3-(trimethylsilyl)phenylzinc halide (1.5 mmol, 1.5 equiv, by *Method A*) and subsequent removal of THF in vacuum, toluene was added and the reaction mixture was heated at 80 °C for 1 h. After work-up, the residue was purified by column chromatography (SiO_2, *i*-hexane / ethyl acetate = 30 / 1) to give the desired product **5g** (83%, 348 mg, 0.83 mmol) as a waxy oil. **^{1}H-NMR (400 MHz, $CDCl_3$):** δ = 7.97 (d, *J* = 8.4 Hz, 2H), 7.40 (d, *J* = 7.2 Hz, 1H), 7.30−7.27 (m, 2H), 7.19 (d, *J* = 8.4 Hz, 4H), 7.04−7.02 (m, 3H), 5.56 (s, 1H), 3.91 (s, 3H), 2.47 (s, 3H), 0.22 (s, 9H). **^{13}C-NMR (100 MHz, $CDCl_3$):** δ = 167.0, 149.3, 141.9, 140.8, 140.1, 136.4, 134.2, 131.6, 129.8, 129.7, 129.6, 129.4, 128.2, 127.8, 126.5, 56.3, 52.0, 15.8, -1.2. **IR (Diamond-ATR, neat):** $\tilde{\nu}$ / cm = 2950, 1719, 1609, 1573, 1492, 1434, 1399, 1310, 1276, 1247, 1179, 1104, 1018, 966, 907, 834, 751, 708. **MS (EI, 70 eV):** m/z (%) = 420 (94), 405 (100), 373 (49), 239 (35), 207 (71), 165 (100). **HRMS (EI, 70 eV)** m/z: calcd for [$C_{25}H_{28}O_2SSi$] 420.1579, found 420.1578.

Synthesis of methyl 4-((4-((tert-butyldimethylsilyl)oxy)phenyl)(1,2-dihydroacenaphthylen-5-yl)methyl)benzoate (5h):

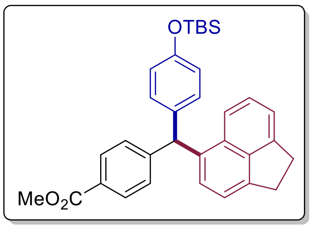

According to **GP2**, (4-(methoxycarbonyl)phenyl)methylene diacetate (**1d**, 1.0 mmol, 1.0 equiv) reacted with (1,2-dihydroacenaphthylen-5-yl)zinc halide (1.0 mmol, 1.0 equiv, by *Method A*) in THF at 60 °C overnight. Then, after addition of 4-((tert-butyldimethylsilyl)oxy)phenylzinc halide (**2g**, 1.5 mmol, 1.5 equiv, by *Method A*) and subsequent removal of THF in vacuum, toluene was added and the reaction mixture was heated at 80 °C for 1 h. After work-up, the residue was purified by column chromatography (SiO_2, *i*-hexane / ethyl acetate = 25 / 1) to give the desired product **5h** (51%, 259 mg, 0.51 mmol) as a waxy solid. **^{1}H-NMR (400 MHz, $CDCl_3$):** δ = 7.97 (d, *J* = 8.4 Hz, 2H), 7.56 (d, *J* = 8.4 Hz, 1H), 7.37 (t, *J* = 7.6 Hz, 1H), 7.29−7.23 (m, 3H), 7.18 (d, *J* = 7.2 Hz, 1H), 6.99 (d, *J* = 8.4 Hz, 2H), 6.89 (d, *J* = 7.2 Hz, 1H), 6.81−6.78 (m, 2H), 6.17 (s, 1H), 3.91 (s, 3H), 3.43−3.37 (m, 4H), 1.00 (s, 9H), 0.22 (s, 6H). **^{13}C-NMR (100 MHz, $CDCl_3$):** δ = 167.0, 154.1, 149.7, 146.4, 145.1, 139.6, 135.6, 135.5, 130.4, 130.3, 129.6, 129.5, 128.7, 128.1, 127.8, 119.9, 119.6, 119.2, 118.7, 52.0, 51.9, 30.5, 29.8, 25.6, 18.1, -4.4. **IR (Diamond-ATR, neat):**

$\tilde{\nu}$ / cm = 3029, 2950, 2927, 2856, 1720, 1607, 1574, 1505, 1471, 1434, 1362, 1251, 1170, 1104, 1019, 909, 836, 779, 730. **MS (EI, 70 eV):** m/z (%) = 508 (100), 451 (59), 301 (75), 241 (34). **HRMS (EI, 70 eV)** m/z: calcd for [$C_{33}H_{36}O_3Si$] 508.2434, found 508.2424.

Synthesis of 4-((2-bromo-4,5-dimethoxyphenyl)(4-(trifluoromethoxy)phenyl)methyl)-N,N-dimethylaniline (5i):

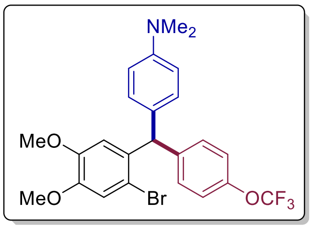

According to **GP2**, (2-bromo-4,5-dimethoxyphenyl)methylene diacetate (**1l**, 1.0 mmol, 1.0 equiv) reacted with 4-(trifluoromethoxy)phenylzinc halide (1.0 mmol, 1.0 equiv, by *Method A*) in THF at 60 °C overnight. Then, after addition of 4-(dimethylamino)phenylzinc halide (1.5 mmol, 1.5 equiv, by *Method A*) and subsequent removal of THF in vacuum, toluene was added and the reaction mixture was heated at 80 °C for 1 h. After work-up, the residue was purified by column chromatography (SiO_2, *i*-hexane / ethyl acetate = 15 / 1) to give the desired product **5i** (71%, 361 mg, 0.71 mmol) as an orange solid. **M.p.:** 134−136 °C. **^{1}H-NMR (400 MHz, $CDCl_3$):** δ = 7.15−7.08 (m, 4H), 7.05 (s, 1H), 6.92 (d, *J* = 8.8 Hz, 2H), 6.68 (d, *J* = 8.8 Hz, 2H), 6.46 (s, 1H), 5.77 (s, 1H), 3.86 (s, 3H), 3.64 (s, 3H), 2.94 (s, 6H). **^{13}C-NMR (100 MHz, $CDCl_3$):** δ = 149.2, 148.1, 148.0, 147.5, 142.4, 135.3, 130.6, 130.0, 129.9, 120.6, 120.4 (q, $^1J_{C\text{-}F}$ = 255 Hz), 115.6, 115.1, 113.7, 112.4, 56.1, 55.9, 54.0, 40.6. **^{19}F-NMR (376 MHz, $CDCl_3$):** δ = −57.8. **IR (Diamond-ATR, neat):** $\tilde{\nu}$ / cm = 2933, 2836, 1611, 1598, 1572, 1503, 1462, 1440, 1374, 1336, 1252, 1201, 1149, 1032, 970, 809, 760, 728. **MS (EI, 70 eV):** m/z (%) = 511 (55), 509 (58), 430 (29), 348 (28), 294 (100), 207 (30), 165 (22). **HRMS (EI, 70 eV)** m/z: calcd for [$C_{24}H_{23}BrF_3NO_3$] 509.0813, found 509.0814.

Synthesis of 4-(benzo[d][1,3]dioxol-5-yl(3-chlorophenyl)methyl)-N,N-dimethylaniline (5j):

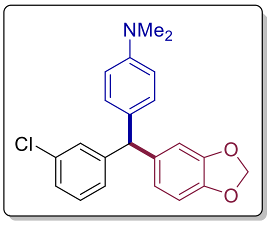

According to **GP2**, 3-chlorobenzal diacetate (**1m**, 1.0 mmol, 1.0 equiv) reacted with benzo[d][1,3]dioxol-5-ylzinc halide (1.0 mmol, 1.0 equiv, by *Method A*) in THF at room temperature overnight. Then, after addition of 4-(dimethylamino)phenylzinc halide (1.5 mmol, 1.5 equiv, by *Method A*) and subsequent removal of THF in vacuum, toluene was added and the reaction mixture was heated at 80 °C for 1 h. After work-up, the residue was purified by column chromatography (SiO_2, *i*-hexane / ethyl acetate = 15 / 1) to give the desired product **5j** (76%, 277 mg, 0.76 mmol) as a waxy oil. **^{1}H-NMR (400 MHz, $CDCl_3$):** δ = 7.24−7.19 (m, 2H), 7.14 (s, 1H), 7.05−7.03 (m, 1H), 6.99 (d, *J* = 8.8 Hz, 2H), 6.76−6.69 (m, 3H), 6.63−6.58 (m, 2H), 5.94 (s, 2H), 5.37 (s, 1H), 2.95 (s, 6H). **^{13}C-NMR (100 MHz, $CDCl_3$):** δ = 149.1, 147.6,

146.9, 145.9, 137.9, 134.0, 131.0, 129.8, 129.4, 129.3, 127.5, 126.3, 122.3, 112.4, 109.8, 107.9, 100.9, 55.2, 40.6. **IR (Diamond-ATR, neat):** $\tilde{\nu}$ / cm = 2881, 2797, 1868, 1611, 1592, 1569, 1518, 1500, 1484, 1438, 1347, 1227, 1202, 1161, 1118, 1036, 929, 807, 771, 731, 685. **MS (EI, 70 eV):** m/z (%) = 365 (48), 254 (100), 165 (11), 152 (16). **HRMS (EI, 70 eV)** m/z: calcd for [$C_{22}H_{20}ClNO_2$] 365.1183, found 365.1178.

Synthesis of 4-((4-bromophenyl)(2-methoxyphenyl)methyl)benzaldehyde (5k):

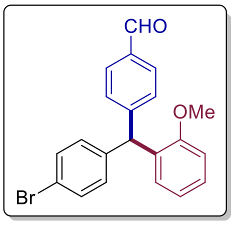

According to **GP2**, 4-bromobenzal diacetate (**1f**, 1.0 mmol, 1.0 equiv) reacted with 2-MeOC$_6$H$_4$ZnX (**2c**, 1.0 mmol, 1.0 equiv, by *Method A*) in THF at 60 °C overnight. Then, after addition of 4-(dimethoxymethyl)phenylzinc halide (1.5 mmol, 1.5 equiv, by *Method B*) and subsequent removal of THF in vacuum, toluene was added and the reaction mixture was heated at 80 °C for 1 h. After work-up, the residue was purified by column chromatography (SiO$_2$, *i*-hexane / ethyl acetate = 20 / 1) to give the desired product **5k** (73%, 277 mg, 0.73 mmol) as a waxy oil. **^{1}H-NMR (400 MHz, CDCl$_3$):** δ = 9.99 (s, 1H), 7.80 (d, J = 8.0 Hz, 2H), 7.42 (d, J = 8.4 Hz, 2H), 7.26 (d, J = 8.0 Hz, 3H), 6.97 (d, J = 8.0 Hz, 2H), 6.90 (t, J = 7.4 Hz, 2H), 6.80 (d, J = 7.2 Hz, 1H), 5.94 (s, 1H), 3.72 (s, 3H). **^{13}C-NMR (100 MHz, CDCl$_3$):** δ = 192.0, 156.8, 150.7, 141.8, 134.7, 131.4, 131.1, 130.8, 130.0, 129.9, 129.7, 128.2, 120.4, 110.7, 55.5, 49.4. **IR (Diamond-ATR, neat):** $\tilde{\nu}$ / cm = 3027, 2934, 2834, 2733, 1904, 1697, 1603, 1485, 1462, 1305, 1241, 1210, 1167, 1106, 1072, 1009, 813, 778, 752. **MS (EI, 70 eV):** m/z (%) = 382 (36), 380 (39), 301 (27), 239 (38), 207 (35), 165 (100). **HRMS (EI, 70 eV)** m/z: calcd for [$C_{21}H_{17}BrO_2$] 380.0412, found 380.0408.

Synthesis of methyl 4-((3-((5-(4-fluorophenyl)thiophen-2-yl)methyl)-4-methylphenyl)(4-methoxyphenyl)methyl)benzoate (5l):

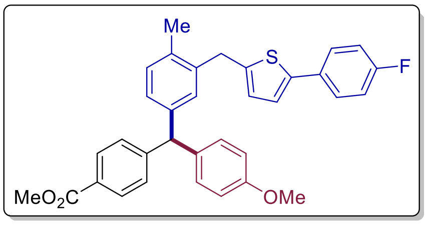

According to **GP2**, (4-(methoxycarbonyl)phenyl)methylene diacetate (**1d**, 1.0 mmol, 1.0 equiv) reacted with 4-MeOC$_6$H$_4$ZnX (**2b**, 1.0 mmol, 1.0 equiv, by *Method A*) in THF at room temperature overnight. Then, after addition of (3-((5-(4-fluorophenyl)thiophen-2-yl)methyl)-4-methylphenyl)zinc halide (1.5 mmol, 1.5 equiv, by *Method B*) and subsequent removal of THF in vacuum, toluene was added and the reaction mixture was heated at 80 °C for 1 h. After work-up, the residue was purified by column chromatography (SiO$_2$, *i*-hexane / ethyl acetate = 25 / 1) to give the desired product **5l** (70%, 375 mg, 0.70 mmol) as a waxy oil. **^{1}H-NMR (400 MHz, CDCl$_3$):** δ = 7.95 (d, J = 8.4 Hz, 2H), 7.49−7.44 (m, 2H), 7.20 (d, J = 8.0 Hz, 2H), 7.10 (d, J = 7.6 Hz, 1H), 7.07−6.97 (m, 6H), 6.90 (dd, J = 8.0, 1.8 Hz, 1H), 6.83 (d, J = 8.8 Hz, 2H),

6.61 (d, J = 3.6 Hz, 1H), 5.52 (s, 1H), 4.05 (s, 2H), 3.90 (s, 3H), 3.78 (s, 3H), 2.30 (s, 3H). **^{13}C-NMR (100 MHz, CDCl$_3$):** δ = 167.0, 162.0 (d, $^1J_{C\text{-}F}$ = 245 Hz), 158.1, 149.8, 143.4, 141.4, 141.3, 138.2, 135.3, 134.6, 130.8 (d, $^4J_{C\text{-}F}$ = 3.2 Hz), 130.5, 130.5, 130.3, 129.6, 129.4, 128.1, 127.8, 127.0 (d, $^3J_{C\text{-}F}$ = 8.0 Hz), 125.8, 122.6, 115.7 (d, $^2J_{C\text{-}F}$ = 21.6 Hz), 113.7, 55.5, 55.2, 52.0, 34.1, 19.0. **^{19}F-NMR (376 MHz, CDCl$_3$):** δ = −115.2. **IR (Diamond-ATR, neat):** $\tilde{\nu}$ / cm = 2997, 2948, 2834, 1716, 1607, 1507, 1434, 1276, 1246, 1176, 1109, 1033, 956, 906, 800, 752. **MS (EI, 70 eV):** m/z (%) = 536 (100), 521 (12), 401 (13), 358 (13), 345 (28), 255 (17), 191 (24). **HRMS (EI, 70 eV)** m/z: calcd for [$C_{34}H_{29}FO_3S$] 536.1821, found 536.1816.

Synthesis of ethyl 5-((4-bromophenyl)(4-fluorophenyl)methyl)thiophene-2-carboxylate (5m):

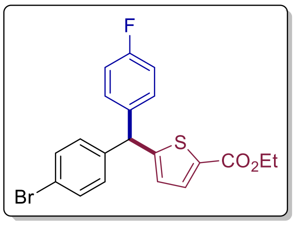

According to **GP2**, 4-bromobenzal diacetate (**1f**, 1.0 mmol, 1.0 equiv) reacted with (5-(ethoxycarbonyl)thiophen-2-yl)zinc halide (1.0 mmol, 1.0 equiv, by *Method B*) in THF at 60 °C for 12 h. Then, after addition of *p*-fluorophenylzinc halide (**2d**, 1.5 mmol, 1.5 equiv, by *Method A*) and subsequent removal of THF in vacuum, toluene was added and the reaction mixture was heated at 80 °C for 1 h. After work-up, the residue was purified by column chromatography (SiO$_2$, *i*-hexane / ethyl acetate = 30 / 1) to give the desired product **5m** (58%, 242 mg, 0.58 mmol) as a waxy oil. **^{1}H-NMR (400 MHz, CDCl$_3$):** δ = 7.66 (d, J = 3.6 Hz, 1H), 7.44 (dd, J = 6.8, 1.6 Hz, 2H), 7.16−7.12 (m, 2H), 7.07−6.99 (m, 4H), 6.70 (dd, J = 3.6, 0.6 Hz, 1H), 5.61 (s, 1H), 4.31 (q, J = 7.2 Hz, 2H), 1.34 (t, J = 7.2 Hz, 3H). **^{13}C-NMR (100 MHz, CDCl$_3$):** δ = 162.0, 161.8 (d, $^1J_{C\text{-}F}$ = 245 Hz), 154.5, 141.6, 138.0 (d, $^4J_{C\text{-}F}$ = 3.3 Hz), 133.2, 133.0, 131.7, 130.3, 130.2 (d, $^3J_{C\text{-}F}$ = 8.0 Hz), 127.2, 121.2, 115.5 (d, $^2J_{C\text{-}F}$ = 21.4 Hz), 61.1, 51.0, 14.3. **^{19}F-NMR (376 MHz, CDCl$_3$):** δ = −115.0. **IR (Diamond-ATR, neat):** $\tilde{\nu}$ / cm = 2980, 1897, 1703, 1603, 1536, 1506, 1485, 1452, 1402, 1367, 1252, 1222, 1158, 1088, 1009, 816, 748. **MS (EI, 70 eV):** m/z (%) = 420 (12), 418 (13), 388 (12), 346 (100), 265 (39), 233 (24), 183 (36). **HRMS (EI, 70 eV)** m/z: calcd for [$C_{20}H_{16}BrFO_2S$] 418.0038, found 418.0035.

Synthesis of methyl 4-(benzo[b]thiophen-3-yl(4-methoxyphenyl)methyl)benzoate (5n):

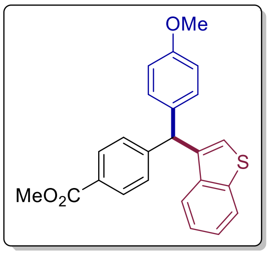

According to **GP2**, (4-(methoxycarbonyl)phenyl)methylene diacetate (**1d**, 1.0 mmol, 1.0 equiv) reacted with benzo[b]thiophen-3-ylzinc halide (1.0 mmol, 1.0 equiv, by *Method B*) in THF at 60 °C for 12 h. Then, after addition of *p*-methoxyphenylzinc halide (**2b**, 1.5 mmol, 1.5 equiv, by *Method A*) and subsequent removal of THF in vacuum, toluene was added and the reaction mixture was heated at 80 °C for 1 h. After work-up, the residue was purified by column chromatography (SiO$_2$, *i*-hexane / ethyl acetate = 20 /

1) to give the desired product **5n** (70%, 270 mg, 0.70 mmol) as an orange waxy oil. **^{1}H-NMR (400 MHz, CDCl$_3$):** δ = 7.98 (d, *J* = 8.4 Hz, 2H), 7.86 (d, *J* = 8.0 Hz, 1H), 7.44 (d, *J* = 8.0 Hz, 1H), 7.32 (t, *J* = 7.2 Hz, 1H), 7.27−7.22 (m, 3H), 7.08 (d, *J* = 8.4 Hz, 2H), 6.86 (d, *J* = 8.8 Hz, 2H), 6.72 (d, *J* = 0.8 Hz, 1H), 5.75 (s, 1H), 3.91 (s, 3H), 3.79 (s, 3H). **^{13}C-NMR (100 MHz, CDCl$_3$):** δ = 166.9, 158.4, 148.3, 140.6, 138.6, 138.2, 133.9, 130.0, 129.8, 129.0, 128.5, 125.2, 124.3, 124.0, 122.8, 122.6, 113.9, 55.2, 52.0, 50.5. **IR (Diamond-ATR, neat):** $\tilde{\nu}$ / cm = 2996, 2948, 2834, 1716, 1608, 1509, 1432, 1276, 1246, 1176, 1102, 1018, 965, 893, 829, 794, 730. **MS (EI, 70 eV):** m/z (%) = 388 (100), 357 (16), 329 (18), 253 (38), 221 (29). **HRMS (EI, 70 eV)** m/z: calcd for [$C_{24}H_{20}O_3S$] 388.1133, found 388.1133.

Synthesis of methyl 4-(benzofuran-5-yl(4-(dimethylamino)phenyl)methyl)benzoate (5o):

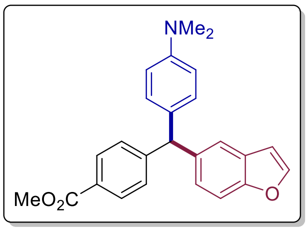

According to **GP2**, (4-(methoxycarbonyl)phenyl)methylene diacetate (**1d**, 1.0 mmol, 1.0 equiv) reacted with benzofuran-5-ylzinc halide (1.0 mmol, 1.0 equiv, by *Method B*) in THF at 60 °C for 12 h. Then, after addition of 4-(dimethylamino)phenylzinc halide (1.5 mmol, 1.5 equiv, by *Method A*) and subsequent removal of THF in vacuum, toluene was added and the reaction mixture was heated at 80 °C for 1 h. After work-up, the residue was purified by column chromatography (SiO$_2$, *i*-hexane / ethyl acetate = 10 / 1) to give the desired product **5o** (57%, 220 mg, 0.57 mmol) as a waxy oil. **^{1}H-NMR (400 MHz, CDCl$_3$):** δ = 7.98 (d, *J* = 8.4 Hz, 2H), 7.61 (d, *J* = 2.4 Hz, 1H), 7.43 (d, *J* = 8.4 Hz, 1H), 7.30−7.24 (m, 3H), 7.10 (dd, *J* = 8.8, 1.6 Hz, 1H), 7.00 (d, *J* = 8.8 Hz, 2H), 6.72−6.68 (m, 3H), 5.64 (s, 1H), 3.92 (s, 3H), 2.95 (s, 6H). **^{13}C-NMR (100 MHz, CDCl$_3$):** δ = 167.0, 153.6, 150.5, 149.1, 145.2, 138.5, 131.2, 129.9, 129.5, 129.4, 127.9, 127.4, 125.9, 121.6, 112.4, 111.0, 106.6, 55.7, 52.0, 40.5. **IR (Diamond-ATR, neat):** $\tilde{\nu}$ / cm = 2948, 2883, 2799, 1716, 1608, 1518, 1465, 1434, 1347, 1275, 1189, 1102, 1018, 946, 908, 804, 732. **MS (EI, 70 eV):** m/z (%) = 385 (100), 268 (25), 250 (55). **HRMS (EI, 70 eV)** m/z: calcd for [$C_{25}H_{23}NO_3$] 385.1678, found 385.1684.

Synthesis of ethyl 5-((4-bromophenyl)(4-methoxyphenyl)methyl)thiophene-2-carboxylate (5p):

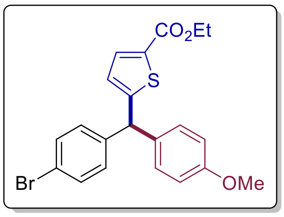

According to **GP2**, 4-bromobenzal diacetate (**1f**, 1.0 mmol, 1.0 equiv) reacted with *p*-methoxyphenylzinc halide (**2b**, 1.0 mmol, 1.0 equiv, by *Method A*) in THF at room temperature for 12 h. Then, after addition of (5-(ethoxycarbonyl)thiophen-2-yl)zinc halide (1.5 mmol, 1.5 equiv, by *Method B*) and subsequent removal of THF in vacuum, toluene was added and the reaction mixture was heated at 80 °C for 1 h. After work-up, the residue was purified by column chromatography (SiO$_2$, *i*-hexane / ethyl acetate = 20 / 1) to give the desired

product **5p** (52%, 223 mg, 0.52 mmol) as a waxy oil. **^{1}H-NMR (400 MHz, CDCl$_3$):** δ = 7.66 (d, *J* = 3.6 Hz, 1H), 7.43 (dd, *J* = 6.6, 1.8 Hz, 2H), 7.08 (d, *J* = 8.4 Hz, 4H), 6.87−6.84 (m, 2H), 6.71 (dd, *J* = 4.0, 0.8 Hz, 1H), 5.58 (s, 1H), 4.31 (t, *J* = 7.2 Hz, 2H), 3.79 (s, 3H), 1.34 (t, *J* = 7.2 Hz, 3H). **^{13}C-NMR (100 MHz, CDCl$_3$):** δ = 162.1, 158.6, 155.4, 142.1, 134.4, 133.2, 132.7, 131.6, 130.4, 129.6, 127.0, 120.9, 114.0, 61.0, 55.2, 51.1, 14.3. **IR (Diamond-ATR, neat):** $\tilde{\nu}$ / cm = 2978, 2834, 1702, 1608, 1583, 1536, 1509, 1451, 1366, 1246, 1175, 1087, 1032, 1009, 803, 748. **MS (EI, 70 eV):** m/z (%) = 432 (24), 430 (25), 401 (22), 359 (100), 275 (29), 247 (33), 207 (22), 171 (26). **HRMS (EI, 70 eV)** m/z: calcd for [$C_{21}H_{19}BrO_3S$] 430.0238, found 430.0229.

Synthesis of methyl 4-(benzo[b]thiophen-3-yl(benzo[d][1,3]dioxol-5-yl)methyl)benzoate (5q):

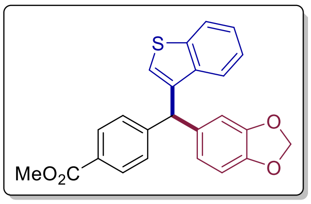

According to **GP2**, (4-(methoxycarbonyl)phenyl)methylene diacetate (**1d**, 1.0 mmol, 1.0 equiv) reacted with benzo[d][1,3]dioxol-5-ylzinc halide (1.0 mmol, 1.0 equiv, by *Method A*) in THF at room temperature for 12 h. Then, after addition of benzo[b]thiophen-3-ylzinc halide (1.5 mmol, 1.5 equiv, by *Method B*) and subsequent removal of THF in vacuum, toluene was added and the reaction mixture was heated at 80 °C for 1 h. After work-up, the residue was purified by column chromatography (SiO_2, *i*-hexane / ethyl acetate = 15 / 1) to give the desired product **5q** (75%, 303 mg, 0.75 mmol) as a waxy oil. **^{1}H-NMR (400 MHz, CDCl$_3$):** δ = 7.98 (d, *J* = 8.4 Hz, 2H), 7.86 (d, *J* = 8.0 Hz, 1H), 7.45 (d, *J* = 8.0 Hz, 1H), 7.34−7.30 (m, 1H), 7.27−7.23 (m, 3H), 6.76−6.74 (m, 2H), 6.66−6.62 (m, 2H), 5.94 (dd, *J* = 4.0, 1.2 Hz, 2H), 5.71 (s, 1H), 3.91 (s, 3H). **^{13}C-NMR (100 MHz, CDCl$_3$):** δ = 166.9, 148.0, 147.8, 146.4, 140.6, 138.2, 138.1, 135.6, 129.8, 129.0, 128.6, 125.2, 124.4, 124.0, 122.8, 122.5, 122.1, 109.4, 108.3, 101.0, 52.1, 50.9. **IR (Diamond-ATR, neat):** $\tilde{\nu}$ / cm = 3423, 2953, 2875, 1718, 1608, 1501, 1486, 1435, 1363, 1276, 1246, 1177, 1103, 1037, 929, 885, 791, 761, 716. **MS (EI, 70 eV):** m/z (%) = 402 (100), 371 (12), 343 (23), 280 (22), 267 (62), 237 (29), 221 (63), 208 (36), 165 (14). **HRMS (EI, 70 eV)** m/z: calcd for [$C_{24}H_{18}O_4S$] 402.0926, found 402.0919.

Synthesis of methyl 4-(benzofuran-6-yl(4-methoxynaphthalen-1-yl)methyl)benzoate (5r):

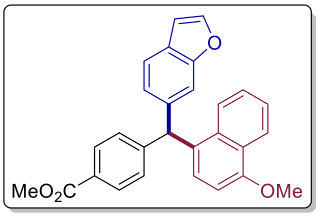

According to **GP2**, (4-(methoxycarbonyl)phenyl)methylene diacetate (**1d**, 1.0 mmol, 1.0 equiv) reacted with (4-methoxynaphthalen-1-yl)zinc halide (1.0 mmol, 1.0 equiv, by *Method A*) in THF at 60 °C for 12 h. Then, after addition of benzofuran-6-ylzinc halide (1.5 mmol, 1.5 equiv, by *Method B*) and

subsequent removal of THF in vacuum, toluene was added and the reaction mixture was heated at 80 °C for 1 h. After work-up, the residue was purified by column chromatography (SiO_2, *i*-hexane / ethyl acetate = 15 / 1) to give the desired product **5r** (62%, 262 mg, 0.62 mmol) as a waxy oil. **^{1}H-NMR (400 MHz, $CDCl_3$):** δ = 8.34 (dd, *J* = 8.0, 1.0 Hz, 1H), 7.98 (d, *J* = 8.4 Hz, 2H), 7.89 (d, *J* = 8.0 Hz, 1H), 7.60 (d, *J* = 2.0 Hz, 1H), 7.48−7.39 (m, 3H), 7.26−7.22 (m, 3H), 7.11 (dd, *J* = 8.6, 1.8 Hz, 1H), 6.80 (d, *J* = 8.0 Hz, 1H), 6.70−6.65 (m, 2H), 6.34 (s, 1H), 3.98 (s, 3H), 3.91 (s, 3H). **^{13}C-NMR (100 MHz, $CDCl_3$):** δ = 167.0, 154.6, 153.7, 150.0, 145.3, 137.8, 132.5, 131.4, 129.6, 129.6, 128.2, 127.7, 127.5, 126.7, 126.1, 125.9, 124.9, 123.9, 122.5, 121.9, 111.3, 106.6, 102.8, 55.4, 52.6, 52.0. **IR (Diamond-ATR, neat):** $\tilde{\nu}$ / cm = 3419, 2951, 2839, 2358, 1716, 1608, 1586, 1513, 1461, 1434, 1381, 1275, 1191, 1091, 1019, 967, 907, 807, 760, 729. **MS (EI, 70 eV):** m/z (%) = 422 (100), 391 (16), 287 (20), 205 (13). **HRMS (EI, 70 eV)** m/z: calcd for [$C_{28}H_{22}O_4$] 422.1518, found 422.1511.

Synthesis of *tert*-butyl(4-((4-methoxyphenyl)(thiophen-2-yl)methyl)phenoxy)dimethylsilane (5s) by a Modified Procedure:

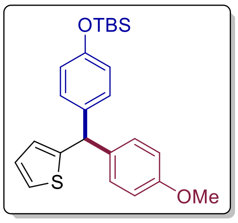

To a dry and argon-flushed *Schlenk*-tube charged with thiophen-2-ylmethylene diacetate (**1g**, 2.0 mmol, 1.0 equiv) in THF (2.0 mL), was added dropwise the mixture of 4-methoxyphenylzinc halide (**2b**, 2.0 mmol, 1.0 equiv) and 4-((*tert*-butyldimethylsilyl)oxy)phenylzinc halide (**2g**, 6.0 mmol, 3.0 equiv) over 6 h. The resulting solution was stirred at room temperature overnight and was then quenched with NH_4Cl (aq.). The aqueous phase was extracted with ethyl acetate (3×10 mL) and the combined organic layers were dried over $MgSO_4$, filtered and concentrated under reduced pressure. Finally, the residue was purified by column chromatography (SiO_2, *i*-hexane / ethyl acetate = 50 / 1) to give the desired product **5s** (67%, 550 mg, 1.34 mmol) as a waxy oil. **^{1}H-NMR (400 MHz, $CDCl_3$):** δ = 7.20 (dd, *J* = 5.2, 1.2 Hz, 1H), 7.15−7.12 (m, 2H), 7.08−7.05 (m, 2H), 6.95−6.92 (m, 1H), 6.87−6.83 (m, 2H), 6.80−6.76 (m, 2H), 6.67 (d, *J* = 3.2 Hz, 1H), 5.59 (s, 1H), 3.80 (s, 3H), 0.99 (s, 9H), 0.20 (s, 6H). **^{13}C-NMR (100 MHz, $CDCl_3$):** δ = 158.2, 154.2, 149.0, 136.8, 136.4, 129.7, 129.6, 126.5, 126.0, 124.3, 119.7, 113.6, 55.2, 50.5, 25.6, 18.2, -4.4. **IR (Diamond-ATR, neat):** $\tilde{\nu}$ / cm = 2953, 2928, 2856, 1606, 1504, 1462, 1361, 1247, 1169, 1107, 1035, 912, 837, 779, 693. **MS (EI, 70 eV):** m/z (%) = 410 (24), 379 (2), 203 (100). **HRMS (EI, 70 eV)** m/z: calcd for [$C_{24}H_{30}O_2SSi$] 410.1736, found 410.1733.

Synthesis of *tert*-butyl(4-((4-methoxyphenyl)(phenanthren-9-yl)methyl)phenoxy)dimethylsilane (5t) by a Modified Procedure:

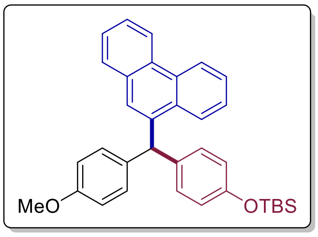

To a dry and argon-flushed *Schlenk*-tube charged with 4-methoxybenzal diacetate (**1b**, 2.0 mmol, 1.0 equiv) in THF (2.0 mL), was added dropwise the mixture of 4-((*tert*-butyldimethylsilyl)oxy)phenylzinc halide (**2g**, 2.0 mmol, 1.0 equiv) and phenanthren-9-ylzinc halide (**2h**, 6.0 mmol, 3.0 equiv) over 6 h. The resulting solution was stirred at room temperature overnight and was then quenched with NH_4Cl (aq.). The aqueous phase was extracted with ethyl acetate (3×10 mL) and the combined organic layers were dried over $MgSO_4$, filtered and concentrated under reduced pressure. Finally, the residue was purified by column chromatography (SiO_2, *i*-hexane / ethyl acetate = 50 / 1) to give the desired product **5t** (70%, 706 mg, 1.40 mmol) as a waxy oil. **^{1}H-NMR (400 MHz, $CDCl_3$):** δ = 8.74 (d, *J* = 8.0 Hz, 1H), 8.68 (d, *J* = 8.0 Hz, 1H), 8.07 (d, *J* = 8.4 Hz, 1H), 7.69 (d, *J* = 8.0 Hz, 1H), 7.64−7.60 (m, 2H), 7.56−7.49 (m, 2H), 7.17 (s, 1H), 7.10 (d, *J* = 8.8 Hz, 2H), 7.03 (d, *J* = 8.4 Hz, 2H), 6.85 (d, *J* = 8.8 Hz, 2H), 6.80 (d, *J* = 8.4 Hz, 2H), 6.17 (s, 1H), 3.80 (s, 3H), 1.01 (s, 9H), 0.22 (s, 6H). **^{13}C-NMR (100 MHz, $CDCl_3$):** δ = 158.0, 154.0, 138.9, 136.5, 135.8, 131.4, 131.2, 130.7, 130.6, 130.5, 129.7, 128.7, 128.4, 126.6, 126.5, 126.3, 126.0, 125.3, 123.0, 122.3, 119.9, 113.7, 55.2, 51.8, 25.7, 18.2, −4.4. **IR (Diamond-ATR, neat):** $\tilde{\nu}$ / cm = 2954, 2926, 2854, 1733, 1606, 1504, 1462, 1361, 1248, 1169, 1099, 1036, 914, 836, 799, 778, 745, 722. **MS (EI, 70 eV):** m/z (%) = 504 (100), 327 (13), 297 (76), 266 (24), 223 (16). **HRMS (EI, 70 eV)** m/z: calcd for [$C_{34}H_{36}O_2Si$] 504.2485, found 504.2479.

Synthesis of an anti-tuberculosis agent (5u):

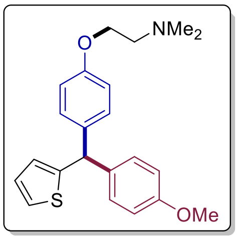

The mixture of **5s** (410 mg, 1.0 mmol, 1.0 equiv), water (90 mg, 5.0 mmol, 5.0 equiv), K_2CO_3 (166 mg, 1.2 mmol, 1.2 equiv) and ethanol (5 mL) was refluxed overnight. Then, the solvent was concentrated under reduced pressure. Water (10 mL) was added and the aqueous phase was extracted with ethyl acetate (3×10 mL). The combined organic layers were dried over $MgSO_4$, filtered and concentrated under reduced pressure to give the desilylation product in pure form. **^{1}H-NMR (400 MHz, $CDCl_3$):** δ = 7.20 (dd, *J* = 4.8, 1.2 Hz, 1H), 7.15−7.11 (m, 2H), 7.09−7.05 (m, 2H), 6.93 (dd, *J* = 5.0, 3.4 Hz, 1H), 6.87−6.83 (m, 2H), 6.79−6.74 (m, 2H), 6.68−6.67 (m, 1H), 5.58 (s, 1H), 4.95 (s, OH), 3.80 (s, 3H). **^{13}C-NMR (100 MHz, $CDCl_3$):** δ = 158.1, 154.1, 148.8, 136.5, 136.3, 129.9, 129.7, 126.5, 126.0, 124.3, 115.1, 113.7, 55.2, 50.4.

The above-obtained product was mixed with 2-chloro-N,N-dimethylethan-1-amine hydrochloride (288 mg, 2.0 mmol, 2.0 equiv), K_2CO_3 (553 mg, 4.0 mmol, 4.0 equiv) and acetone (10 mL). The mixture was refluxed for 6 h and then acetone was removed under reduced pressure. Water (10 mL) was added and the aqueous phase was extracted with ethyl

acetate (3×10 mL). The combined organic layers were dried over $MgSO_4$, filtered and concentrated under reduced pressure to give the desired product **5u** (94%, 345 mg, 0.94 mmol) as a waxy oil. **^{1}H-NMR (400 MHz, $CDCl_3$):** δ = 7.19 (dd, J = 5.2, 1.2 Hz, 1H), 7.14−7.09 (m, 4H), 6.92 (dd, J = 5.0, 3.4 Hz, 1H), 6.87−6.82 (m, 4H), 6.67−6.66 (m, 1H), 5.58 (s, 1H), 4.05 (t, J = 5.8 Hz, 2H), 3.79 (s, 3H), 2.72 (t, J = 5.8 Hz, 2H), 2.34 (s, 6H). **^{13}C-NMR (100 MHz, $CDCl_3$):** δ = 158.2, 157.5, 148.9, 136.4, 136.3, 129.7, 129.6, 126.5, 126.0, 124.3, 114.3, 113.6, 65.8, 58.2, 55.2, 50.4, 45.9. **IR (Diamond-ATR, neat):** $\tilde{\nu}$ / cm = 2924, 2853, 2771, 1608, 1582, 1507, 1462, 1300, 1241, 1174, 1109, 1033, 956, 826, 793, 763, 745, 693. **MS (EI, 70 eV):** m/z (%) = 367 (2), 203 (85), 189 (14), 171 (42), 152 (22), 115 (27), 58 (100). **HRMS (EI, 70 eV)** m/z: calcd for [$C_{22}H_{25}NO_2S$] 367.1606, found 367.1601.

Synthesis of an anti-breast cancer agent (5v):

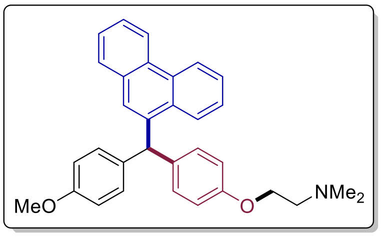

The mixture of **5t** (504 mg, 1.0 mmol, 1.0 equiv), water (90 mg, 5.0 mmol, 5.0 equiv), K_2CO_3 (166 mg, 1.2 mmol, 1.2 equiv) and ethanol (5 mL) was refluxed overnight. Then, the solvent was concentrated under reduced pressure. Water (10 mL) was added and the aqueous phase was extracted with ethyl acetate (3×10 mL). The combined organic layers were dried over $MgSO_4$, filtered and concentrated under reduced pressure to give the desilylation product in pure form. **^{1}H-NMR (400 MHz, $CDCl_3$):** δ = 8.73 (d, J = 8.0 Hz, 1H), 8.66 (d, J = 8.0 Hz, 1H), 8.04 (d, J = 8.4 Hz, 1H), 7.69 (d, J = 8.0 Hz, 1H), 7.62−7.59 (m, 2H), 7.55−7.48 (m, 2H), 7.16 (s, 1H), 7.09−7.01 (m, 4H), 6.86−6.82 (m, 2H), 6.78−6.74 (m, 2H), 6.15 (s, 1H), 4.93 (s, OH), 3.80 (s, 3H). **^{13}C-NMR (100 MHz, $CDCl_3$):** δ = 158.0, 154.0, 138.8, 136.0, 135.8, 131.4, 131.1, 130.7, 130.6, 129.7, 128.7, 128.3, 126.6, 126.5, 126.4, 126.1, 125.2, 123.0, 122.3, 115.2, 113.8, 55.2, 51.8.

The above-obtained product was mixed with 2-chloro-N,N-dimethylethan-1-amine hydrochloride (288 mg, 2.0 mmol, 2.0 equiv), K_2CO_3 (553 mg, 4.0 mmol, 4.0 equiv) and acetone (10 mL). The mixture was refluxed for 6 h and then acetone was removed under reduced pressure. Water (10 mL) was added and the aqueous phase was extracted with ethyl acetate (3×10 mL). The combined organic layers were dried over $MgSO_4$, filtered and concentrated under reduced pressure to give the desired product **5v** (89%, 411 mg, 0.89 mmol) as a waxy oil. **^{1}H-NMR (400 MHz, $CDCl_3$):** δ = 8.72 (d, J = 8.0 Hz, 1H), 8.66 (d, J = 8.0 Hz, 1H), 8.04 (d, J = 8.4 Hz, 1H), 7.68 (d, J = 8.0 Hz, 1H), 7.62−7.59 (m, 2H), 7.54−7.47 (m, 2H), 7.15 (s, 1H), 7.08−7.04 (m, 4H), 6.86−6.82 (m, 4H), 6.15 (s, 1H), 4.06 (t, J = 5.8 Hz, 2H), 3.79 (s, 3H), 2.76 (t, J = 5.6 Hz, 2H), 2.36 (s, 6H). **^{13}C-NMR (100 MHz, $CDCl_3$):** δ = 158.0, 157.2, 138.8, 136.0, 135.8, 131.4, 131.2, 130.8, 130.5, 130.5, 129.7, 128.7, 128.3, 126.6, 126.5, 126.4, 126.0, 125.2, 123.0, 122.3, 114.4, 113.7, 65.6, 58.2, 55.2, 51.8, 45.8. **IR (Diamond-**

ATR, neat): ṽ / cm = 2924, 2854, 2771, 1712, 1608, 1582, 1507, 1462, 1361, 1299, 1242, 1175, 1149, 1034, 906, 836, 800, 747, 723. **MS (EI, 70 eV):** m/z (%) = 461 (5), 389 (27), 297 (13), 58 (100). **HRMS (EI, 70 eV)** m/z: calcd for [$C_{32}H_{31}NO_2$] 461.2355, found 461.2346.

Synthesis of (1-(p-tolyl)butane-1,4-diyl)dibenzene (6a):

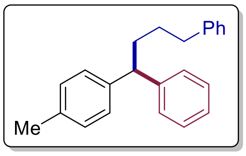

According to **GP3**, *p*-methylbenzal diacetate (**1a**, 1.0 mmol, 1.0 equiv) reacted with PhZnX (**2a**, 1.0 mmol, 1.0 equiv, by *Method A*) in THF at 60 °C for 3 h. Then, after addition of 3-phenylpropylzinc halide (1.5 mmol, 1.5 equiv, by *Method A*) and subsequent removal of THF in vacuum, toluene was added and the reaction mixture was heated at 80 °C for 1 h. After work-up, the residue was purified by column chromatography (SiO_2, *i*-hexane) to give the desired product **6a** (83%, 249 mg, 0.83 mmol) as a colorless oil. **^{1}H-NMR (400 MHz, CDCl$_3$):** δ = 7.31−7.22 (m, 6H), 7.20−7.09 (m, 8H), 3.90 (t, *J* = 7.8 Hz, 1H), 2.66 (t, *J* = 7.6 Hz, 2H), 2.32 (s, 3H), 2.12−2.06 (m, 2H), 1.66−1.59 (m, 2H). **^{13}C-NMR (100 MHz, CDCl$_3$):** δ = 145.3, 142.3, 142.0, 135.5, 129.1, 128.4, 128.3, 128.2, 127.7, 127.6, 125.9, 125.6, 50.8, 35.8, 35.2, 29.8, 21.0. **IR (Diamond-ATR, neat):** ṽ / cm = 3023, 2925, 2857, 1658, 1600, 1511, 1493, 1451, 1276, 1178, 1112, 1073, 1030, 909, 808, 747, 696. **MS (EI, 70 eV):** m/z (%) = 300 (2), 182 (15), 181 (100), 166 (24), 165 (24). **HRMS (EI, 70 eV)** m/z: calcd for [$C_{23}H_{24}$] 300.1878, found 300.1872.

Synthesis of 4,4'-(4-phenylbutane-1,1-diyl)bis(fluorobenzene) (6b):

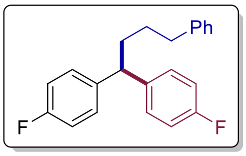

According to **GP3**, *p*-fluorobenzal diacetate (**1c**, 1.0 mmol, 1.0 equiv) reacted with *p*-fluorophenylzinc halide (**2d**, 1.0 mmol, 1.0 equiv, by *Method A*) in THF at room temperature overnight. Then, after addition of 3-phenylpropylzinc halide (1.5 mmol, 1.5 equiv, by *Method A*) and subsequent removal of THF in vacuum, toluene was added and the reaction mixture was heated at 80 °C for 1 h. After work-up, the residue was purified by column chromatography (SiO_2, *i*-hexane) to give the desired product **6b** (86%, 277 mg, 0.86 mmol) as a colorless oil. **^{1}H-NMR (400 MHz, CDCl$_3$):** δ = 7.29−7.25 (m, 2H), 7.20−7.12 (m, 7H), 6.99−6.93 (m, 4H), 3.88 (t, *J* = 7.8 Hz, 1H), 2.64 (t, *J* = 7.6 Hz, 2H), 2.06−2.00 (m, 2H), 1.62−1.55 (m, 2H). **^{13}C-NMR (100 MHz, CDCl$_3$):** δ = 161.3 (d, $^1J_{C\text{-}F}$ = 243 Hz), 142.1, 140.5 (d, $^4J_{C\text{-}F}$ = 3.0 Hz), 129.0 (d, $^3J_{C\text{-}F}$ = 7.8 Hz), 128.3, 128.3, 125.8, 115.2 (d, $^2J_{C\text{-}F}$ = 20.9 Hz), 49.6, 35.7, 35.4, 29.6. **^{19}F-NMR (376 MHz, CDCl$_3$):** δ = −117.0. **IR (Diamond-ATR, neat):** ṽ / cm = 3026, 2932, 2858, 1887, 1601, 1504, 1452, 1413, 1299, 1219, 1156, 1100, 1014, 822, 746, 698. **MS (EI, 70 eV):** m/z (%) = 322 (1), 204 (15), 203 (100), 201 (15), 183 (29). **HRMS (EI, 70 eV)** m/z: calcd for [$C_{22}H_{20}F_2$] 322.1533, found 322.1526.

Synthesis of 4-(1-(4-fluorophenyl)pentyl)benzonitrile (6c):

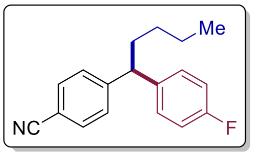

According to **GP3**, *p*-cyanobenzal diacetate (**1e**, 1.0 mmol, 1.0 equiv) reacted with *p*-fluorophenylzinc halide (**2d**, 1.0 mmol, 1.0 equiv, by *Method A*) in THF at room temperature for 12 h. Then, after addition of butylzinc halide (1.5 mmol, 1.5 equiv, by *Method A*) and subsequent removal of THF in vacuum, toluene was added and the reaction mixture was heated at 120 °C for 1 h. After work-up, the residue was purified by column chromatography (SiO_2, *i*-hexane / ethyl acetate = 25 / 1) to give the desired product **6c** (91%, 243 mg, 0.91 mmol) as a colorless oil. **^{1}H-NMR (400 MHz, $CDCl_3$):** δ = 7.57 (d, *J* = 8.4 Hz, 2H), 7.31 (d, *J* = 8.4 Hz, 2H), 7.18−7.13 (m, 2H), 7.01−6.95 (m, 2H), 3.92 (t, *J* = 7.8 Hz, 1H), 2.05−1.97 (m, 2H), 1.39−1.28 (m, 2H), 1.27−1.15 (m, 2H), 0.87 (t, *J* = 7.2 Hz, 3H). **^{13}C-NMR (100 MHz, $CDCl_3$):** δ = 161.4 (d, $^1J_{C\text{-}F}$ = 244 Hz), 150.7, 139.3 (d, $^4J_{C\text{-}F}$ = 3.3 Hz), 132.3, 129.1 (d, $^3J_{C\text{-}F}$ = 7.9 Hz), 128.5, 118.9, 115.4 (d, $^2J_{C\text{-}F}$ = 21.1 Hz), 110.0, 50.5, 35.1, 29.9, 22.5, 13.9. **^{19}F-NMR (376 MHz, $CDCl_3$):** δ = −116.3. **IR (Diamond-ATR, neat):** $\tilde{\nu}$ / cm = 2956, 2929, 2859, 2226, 1607, 1507, 1466, 1414, 1379, 1302, 1221, 1158, 1015, 942, 818, 755. **MS (EI, 70 eV):** m/z (%) = 267 (2), 210 (100), 208 (20), 190 (20), 183 (18). **HRMS (EI, 70 eV)** m/z: calcd for [$C_{18}H_{18}FN$] 267.1423, found 267.1418.

Synthesis of methyl 4-(1-(4-(methylthio)phenyl)ethyl)benzoate (6d):

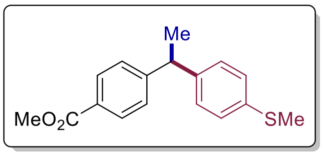

According to **GP3**, (4-(methoxycarbonyl)phenyl)methylene diacetate (**1d**, 1.0 mmol, 1.0 equiv) reacted with 4-(methylthio)phenylzinc halide (1.0 mmol, 1.0 equiv, by *Method A*) in THF at 60 °C for 12 h. Then, after addition of methylzinc halide (1.5 mmol, 1.5 equiv, by *Method A*) and subsequent removal of THF in vacuum, toluene was added and the reaction mixture was heated at 80 °C for 1 h. After work-up, the residue was purified by column chromatography (SiO_2, *i*-hexane / ethyl acetate = 25 / 1) to give the desired product **6d** (92%, 263 mg, 0.92 mmol) as a waxy oil. **^{1}H-NMR (400 MHz, $CDCl_3$):** δ = 7.95 (d, *J* = 8.4 Hz, 2H), 7.27 (d, *J* = 8.0 Hz, 2H), 7.20 (dd, *J* = 6.6, 1.8 Hz, 2H), 7.12 (d, *J* = 8.0 Hz, 2H), 4.16 (q, *J* = 7.2 Hz, 1H), 3.89 (s, 3H), 2.46 (s, 3H), 1.63 (d, *J* = 7.2 Hz, 3H). **^{13}C-NMR (100 MHz, $CDCl_3$):** δ = 167.0, 151.5, 142.4, 136.0, 129.7, 128.0, 128.0, 127.6, 126.8, 52.0, 44.2, 21.5, 16.0. **IR (Diamond-ATR, neat):** $\tilde{\nu}$ / cm = 2966, 2920, 1715, 1608, 1573, 1494, 1433, 1274, 1178, 1104, 1014, 965, 857, 820, 774, 754, 704. **MS (EI, 70 eV):** m/z (%) = 286 (46), 271 (100), 225 (16), 207 (36), 178 (13), 165 (58). **HRMS (EI, 70 eV)** m/z: calcd for [$C_{17}H_{18}O_2S$] 286.1028, found 286.1021.

Synthesis of methyl 4-(cyclohexyl(4-(methylthio)phenyl)methyl)benzoate (6e):

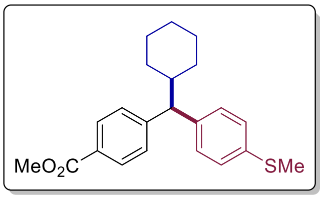

According to **GP3**, (4-(methoxycarbonyl)phenyl)methylene diacetate (**1d**, 1.0 mmol, 1.0 equiv) reacted with 4-(methylthio)phenylzinc halide (1.0 mmol, 1.0 equiv, by *Method A*) in THF at 60 °C for 12 h. Then, after addition of cyclohexylzinc halide (1.5 mmol, 1.5 equiv, by *Method A*) and subsequent removal of THF in vacuum, toluene was added and the reaction mixture was heated at 80 °C for 1 h. After work-up, the residue was purified by column chromatography (SiO_2, *i*-hexane / ethyl acetate = 30 / 1) to give the desired product **6e** (74%, 262 mg, 0.74 mmol) as a waxy oil. **^{1}H-NMR (400 MHz, $CDCl_3$):** δ = 7.93 (d, *J* = 8.4 Hz, 2H), 7.32 (d, *J* = 8.4 Hz, 2H), 7.20−7.15 (m, 4H), 3.87 (s, 3H), 3.50 (d, *J* = 11.2 Hz, 1H), 2.43 (s, 3H), 2.15−2.05 (m, 1H), 1.68−1.64 (m, 4H), 1.55−1.52 (m, 1H), 1.28−1.08 (m, 3H), 0.90−0.80 (m, 2H). **^{13}C-NMR (100 MHz, $CDCl_3$):** δ = 167.0, 149.8, 140.5, 135.9, 129.8, 128.6, 128.0, 127.9, 126.9, 58.9, 52.0, 41.0, 31.9, 31.9, 26.4, 26.2, 16.0. **IR (Diamond-ATR, neat):** $\tilde{\nu}$ / cm = 2919, 2848, 1716, 1608, 1573, 1492, 1433, 1275, 1176, 1102, 1018, 966, 869, 823, 790, 766, 728, 705. **MS (EI, 70 eV):** m/z (%) = 354 (4), 281 (25), 271 (100), 225 (44), 209 (21), 207 (66), 191 (15), 165 (42). **HRMS (EI, 70 eV)** m/z: calcd for [$C_{22}H_{26}O_2S$] 354.1654, found 354.1648.

Synthesis of 4-(5-chloro-1-(4-methoxyphenyl)pentyl)benzonitrile (6f):

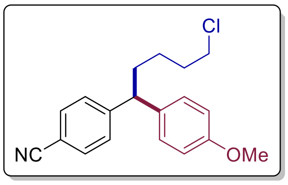

According to **GP3**, 4-cyanobenzal diacetate (**1e**, 1.0 mmol, 1.0 equiv) reacted with 4-methoxyphenylzinc halide (**2b**, 1.0 mmol, 1.0 equiv, by *Method A*) in THF at room temperature overnight. Then, after addition of (4-chlorobutyl)zinc halide (1.5 mmol, 1.5 equiv, by *Method B*) and subsequent removal of THF in vacuum, toluene was added and the reaction mixture was heated at 80 °C for 1 h. After work-up, the residue was purified by column chromatography (SiO_2, *i*-hexane / ethyl acetate = 20 / 1) to give the desired product **6f** (59%, 186 mg, 0.59 mmol) as a colorless oil. A scale-up of this reaction to 10 mmol gave **6f** in 60% (1.88 g, 6.0 mmol) yield. **^{1}H-NMR (400 MHz, $CDCl_3$):** δ = 7.56 (d, *J* = 8.0 Hz, 2H), 7.32 (d, *J* = 8.4 Hz, 2H), 7.11 (d, *J* = 8.8 Hz, 2H), 6.86−6.82 (m, 2H), 3.90 (t, *J* = 7.8 Hz, 1H), 3.78 (s, 3H), 3.49 (t, *J* = 6.6 Hz, 2H), 2.10−1.96 (m, 2H), 1.80 (quint, *J* = 7.2 Hz, 2H), 1.45−1.34 (m, 2H). **^{13}C-NMR (100 MHz, $CDCl_3$):** δ = 158.2, 150.9, 135.2, 132.3, 128.6, 128.4, 118.9, 114.1, 109.9, 55.2, 50.4, 44.7, 34.6, 32.4, 25.2. **IR (Diamond-ATR, neat):** $\tilde{\nu}$ / cm = 2998, 2933, 2835, 2225, 1607, 1582, 1510, 1461, 1302, 1246, 1177, 1110, 1032, 823, 755, 719. **MS (EI, 70 eV):** m/z (%) = 313 (2), 223 (16), 222 (100), 207 (10). **HRMS (EI, 70 eV)** m/z: calcd for [$C_{19}H_{20}ClNO$] 313.1233, found 313.1227.

Synthesis of 2-(3-(4-methoxyphenyl)-3-(4-(trifluoromethyl)phenyl)propyl)-1,3-dioxane (6g):

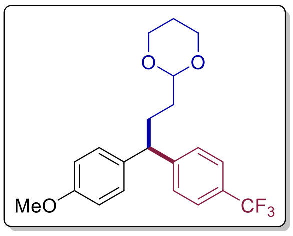

According to **GP3**, 4-methoxybenzal diacetate (**1b**, 1.5 mmol, 1.0 equiv) reacted with 4-(trifluoromethyl)phenylzinc halide (**2e**, 1.5 mmol, 1.0 equiv, by *Method A*) in THF at 60 °C overnight. Then, after addition of 2-(1,3-dioxan-2-yl)ethylzinc halide (2.25 mmol, 1.5 equiv, by *Method A*) and subsequent removal of THF in vacuum, toluene was added and the reaction mixture was heated at 80 °C for 1 h. After work-up, the residue was purified by column chromatography (SiO_2, *i*-hexane / ethyl acetate = 10 / 1) to give the desired product **6g** (64%, 365 mg, 0.64 mmol) as a waxy oil. **^{1}H-NMR (400 MHz, $CDCl_3$):** δ = 7.51 (d, *J* = 8.0 Hz, 2H), 7.33 (d, *J* = 8.0 Hz, 2H), 7.15−7.12 (m, 2H), 6.84−6.81 (m, 2H), 4.52 (t, *J* = 5.2 Hz, 1H), 4.10−4.06 (m, 2H), 3.89 (t, *J* = 8.0 Hz, 1H), 3.77−3.70 (m, 5H), 2.18−2.10 (m, 2H), 2.09−1.99 (m, 1H), 1.61−1.48 (m, 2H), 1.34−1.30 (m, 1H). **^{13}C-NMR (100 MHz, $CDCl_3$):** δ = 158.1, 149.4, 135.8, 129.8 (q, $^2J_{C\text{-}F}$ = 32.2 Hz), 128.7, 128.0, 125.3 (q, $^3J_{C\text{-}F}$ = 3.7 Hz), 124.2 (q, $^1J_{C\text{-}F}$ = 270 Hz), 113.9, 101.9, 66.8, 55.2, 50.1, 33.6, 29.6, 25.7. **^{19}F-NMR (376 MHz, $CDCl_3$):** δ = −62.3. **IR (Diamond-ATR, neat):** $\tilde{\nu}$ / cm = 2957, 2849, 1679, 1609, 1583, 1510, 1462, 1414, 1322, 1247, 1113, 1067, 1017, 990, 894, 825, 766. **MS (EI, 70 eV):** m/z (%) = 313 (2), 223 (16), 222 (100), 207 (10). **HRMS (EI, 70 eV)** m/z: calcd for [$C_{21}H_{23}F_3O_3$] 380.1599, found [M-H]$^+$ 379.1517.

Synthesis of ethyl 7-(4-bromophenyl)-7-(4-methoxyphenyl)heptanoate (6h):

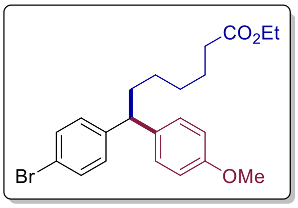

According to **GP3**, 4-bromobenzal diacetate (**1f**, 1.0 mmol, 1.0 equiv) reacted with 4-methoxyphenylzinc halide (**2b**, 1.0 mmol, 1.0 equiv, by *Method A*) in THF at room temperature for 12 h. Then, after addition of (6-ethoxy-6-oxohexyl)zinc halide (1.5 mmol, 1.5 equiv, by *Method B*) and subsequent removal of THF in vacuum, toluene was added and the reaction mixture was heated at 80 °C for 1 h. After work-up, the residue was purified by column chromatography (SiO_2, *i*-hexane / ethyl acetate = 15 / 1) to give the desired product **6h** (81%, 338 mg, 0.81 mmol) as a colorless oil. **^{1}H-NMR (400 MHz, $CDCl_3$):** δ = 7.38 (d, *J* = 8.4 Hz, 2H), 7.12−7.07 (m, 4H), 6.84−6.80 (m, 2H), 4.11 (q, *J* = 7.2 Hz, 2H), 3.79 (t, *J* = 7.4 Hz, 1H), 3.77 (s, 3H), 2.25 (t, *J* = 7.4 Hz, 2H), 2.00−1.94 (m, 2H), 1.63−1.55 (m, 2H), 1.38−1.31 (m, 2H), 1.24 (t, *J* = 7.2 Hz, 5H). **^{13}C-NMR (100 MHz, $CDCl_3$):** δ = 173.7, 157.9, 144.6, 136.6, 131.4, 129.4, 128.6, 119.6, 113.8, 60.2, 55.2, 49.8, 35.4, 34.2, 29.0, 27.6, 24.8, 14.2. **IR (Diamond-ATR, neat):** $\tilde{\nu}$ / cm = 2930, 2857, 1729, 1609, 1583, 1510, 1485, 1463, 1371, 1301, 1245, 1175, 1073, 1033, 1008, 816. **MS (EI, 70 eV):** m/z (%) = 420 (1), 418 (1),

277 (96), 275 (100), 196 (20), 181 (12), 165 (12). **HRMS (EI, 70 eV)** m/z: calcd for [$C_{22}H_{27}BrO_3$] 418.1144, found 418.1136.

Synthesis of 7-(4-bromophenyl)-7-(2-methoxyphenyl)-2,2-dimethylheptanenitrile (6i):

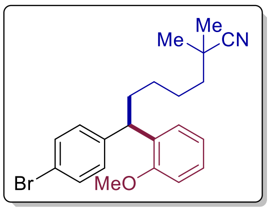

According to **GP3**, 4-bromobenzal diacetate (**1f**, 1.0 mmol, 1.0 equiv) reacted with 2-methoxyphenylzinc halide (**2c**, 1.0 mmol, 1.0 equiv, by *Method A*) in THF at 60 °C for 12 h. Then, after addition of (5-cyano-5-methylhexyl)zinc halide (1.5 mmol, 1.5 equiv, by *Method B*) and subsequent removal of THF in vacuum, toluene was added and the reaction mixture was heated at 80 °C for 1 h. After work-up, the residue was purified by column chromatography (SiO_2, *i*-hexane / ethyl acetate = 25 / 1) to give the desired product **6i** (82%, 327 mg, 0.82 mmol) as a colorless oil. **^{1}H-NMR (400 MHz, CDCl$_3$):** δ = 7.38 (d, *J* = 8.4 Hz, 2H), 7.21−7.13 (m, 4H), 6.94 (t, *J* = 7.2 Hz, 1H), 6.84 (d, *J* = 8.0 Hz, 1H), 4.34 (t, *J* = 7.8 Hz, 1H), 3.78 (s, 3H), 2.08−1.94 (m, 2H), 1.56−1.44 (m, 4H), 1.37−1.27 (m, 8H). **^{13}C-NMR (100 MHz, CDCl$_3$):** δ = 156.8, 144.0, 132.7, 131.1, 129.8, 127.2, 127.2, 125.1, 120.5, 119.4, 110.6, 55.3, 42.5, 40.8, 34.4, 32.3, 27.8, 26.6, 26.6, 25.2. **IR (Diamond-ATR, neat):** $\tilde{\nu}$ / cm = 2973, 2935, 2860, 2232, 1899, 1598, 1585, 1485, 1462, 1405, 1289, 1240, 1072, 1028, 1009, 816, 751. **MS (EI, 70 eV):** m/z (%) = 399 (2), 320 (12), 277 (60), 275 (61), 196 (26), 171 (98), 169 (100). **HRMS (EI, 70 eV)** m/z: calcd for [$C_{22}H_{26}BrNO$] 399.1198, found 399.1191.

Synthesis of 3-(2-(2-bromo-4,5-dimethoxyphenyl)-2-(4-(trifluoromethoxy)phenyl)ethyl)benzonitrile (6j):

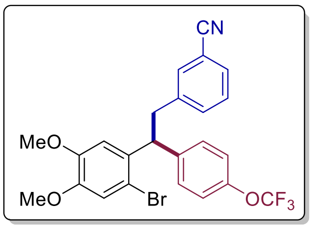

According to **GP3**, (2-bromo-4,5-dimethoxyphenyl)methylene diacetate (**1l**, 1.0 mmol, 1.0 equiv) reacted with 4-(trifluoromethoxy)phenylzinc halide (1.0 mmol, 1.0 equiv, by *Method A*) in THF at 60 °C overnight. Then, after addition of 3-cyanobenzylzinc halide (1.5 mmol, 1.5 equiv, by *Method B*) and subsequent removal of THF in vacuum, toluene was added and the reaction mixture was heated at 80 °C for 1 h. After work-up, the residue was purified by column chromatography (SiO_2, *i*-hexane / ethyl acetate = 10 / 1) to give the desired product **6j** (85%, 429 mg, 0.85 mmol) as a waxy oil. **^{1}H-NMR (400 MHz, CDCl$_3$):** δ = 7.46−7.44 (m, 1H), 7.36 (s, 1H), 7.31−7.22 (m, 4H), 7.12 (d, *J* = 8.4 Hz, 2H), 6.96 (s, 1H), 6.72 (s, 1H), 4.74 (t, *J* = 8.0 Hz, 1H), 3.83 (s, 3H), 3.82 (s, 3H), 3.38−3.28 (m, 2H). **^{13}C-NMR (100 MHz, CDCl$_3$):** δ = 148.6, 148.3, 147.8, 140.9, 140.5, 133.7, 133.4, 132.4, 130.1, 129.2, 129.0, 120.9, 120.4 (q, $^1J_{C\text{-}F}$ = 256 Hz), 118.8, 115.5, 114.9, 112.2, 111.2, 56.1, 56.0, 49.4, 41.0. **^{19}F-NMR (376 MHz, CDCl$_3$):** δ = −57.9. **IR (Diamond-ATR, neat):** $\tilde{\nu}$ / cm = 2935, 2841, 2228, 1600, 1501, 1439, 1378, 1252,

1206, 1154, 1018, 920, 850, 794, 688. **MS (EI, 70 eV):** m/z (%) = 505 (1), 388 (100), 310 (52), 295 (32), 279 (21), 267 (25), 225 (12), 181 (22). **HRMS (EI, 70 eV)** m/z: calcd for [$C_{24}H_{19}BrF_3NO_3$] 505.0500, found 505.0498.

Synthesis of ethyl 5-(5-(benzo[d][1,3]dioxol-5-yloxy)-1-(4-bromophenyl)pentyl)thiophene-2-carboxylate (6k):

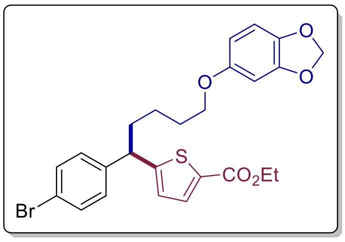

According to **GP3**, 4-bromobenzal diacetate (**1f**, 1.0 mmol, 1.0 equiv) reacted with (5-(ethoxycarbonyl)thiophen-2-yl)zinc halide (1.0 mmol, 1.0 equiv, by *Method B*) in THF at 60 °C for 12 h. Then, after addition of (4-(benzo[d][1,3]dioxol-5-yloxy)butyl)zinc halide (1.5 mmol, 1.5 equiv, by *Method A*) and subsequent removal of THF in vacuum, toluene was added and the reaction mixture was heated at 80 °C for 1 h. After work-up, the residue was purified by column chromatography (SiO_2, *i*-hexane / ethyl acetate = 20 / 1) to give the desired product **6k** (61%, 315 mg, 0.61 mmol) as a waxy oil. **^{1}H-NMR (400 MHz, $CDCl_3$):** δ = 7.64 (d, *J* = 3.6 Hz, 1H), 7.41 (d, *J* = 8.4 Hz, 2H), 7.05 (d, *J* = 8.4 Hz, 2H), 6.69 (d, *J* = 4.0 Hz, 1H), 6.51 (s, 1H), 6.44 (s, 1H), 5.94 (s, 1H), 5.89 (d, *J* = 2.4 Hz, 2H), 4.30 (q, *J* = 7.2 Hz, 2H), 3.81 (t, *J* = 6.4 Hz, 2H), 1.63−1.56 (m, 2H), 1.35−1.21 (m, 5H), 0.87 (t, *J* = 7.2 Hz, 3H). **^{13}C-NMR (100 MHz, $CDCl_3$):** δ = 162.2, 155.5, 151.2, 147.1, 142.0, 140.8, 133.2, 132.2, 131.4, 130.4, 126.9, 123.3, 120.6, 109.0, 101.2, 95.5, 68.8, 61.0, 44.8, 31.2, 19.1, 14.3, 13.7. **IR (Diamond-ATR, neat):** $\tilde{\nu}$ / cm = 2957, 2871, 1703, 1625, 1536, 1502, 1483, 1399, 1366, 1257, 1171, 1088, 1036, 1009, 934, 821, 748. **MS (EI, 70 eV):** m/z (%) = 518 (58), 516 (54), 461 (32), 433 (19), 386 (24), 303 (19), 225 (70), 138 (100). **HRMS (EI, 70 eV)** m/z: calcd for [$C_{25}H_{25}BrO_5S$] 516.0606, found 516.0605.

Synthesis of methyl 4-(1-(benzo[b]thiophen-3-yl)-2-cyclohexylethyl)benzoate (6l):

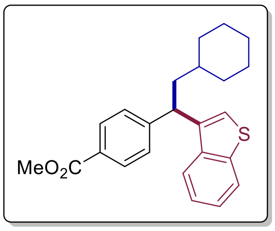

According to **GP3**, (4-(methoxycarbonyl)phenyl)methylene diacetate (**1d**, 1.0 mmol, 1.0 equiv) reacted with benzo[b]thiophen-3-ylzinc halide (1.0 mmol, 1.0 equiv, by *Method B*) in THF at 60 °C for 12 h. Then, after addition of cyclohexylmethylzinc halide (1.5 mmol, 1.5 equiv, by *Method A*) and subsequent removal of THF in vacuum, toluene was added and the reaction mixture was heated at 80 °C for 1 h. After work-up, the residue was purified by column chromatography (SiO_2, *i*-hexane / ethyl acetate = 20 / 1) to give the desired product **6l** (65%, 246 mg, 0.65 mmol) as a white solid. **M.p.:** 94−95 °C. **^{1}H-NMR (400 MHz, $CDCl_3$):** δ = 7.94 (d, *J* = 8.4 Hz, 2H), 7.85−7.81 (m, 1H), 7.62−7.58 (m, 1H), 7.35−7.31 (m, 2H), 7.30−7.25 (m, 2H), 7.23 (s, 1H), 4.48 (t, *J* = 7.6 Hz, 1H), 3.88 (s, 3H), 2.11−2.04 (m, 1H), 1.97−1.90 (m, 2H), 1.72−1.62 (m, 4H), 1.27−1.09 (m, 4H), 1.06−0.94 (m, 2H). **^{13}C-NMR (100

MHz, CDCl$_3$): δ = 167.0, 149.6, 140.5, 138.8, 138.5, 129.8, 128.2, 127.9, 124.3, 123.9, 122.8, 122.0, 121.6, 52.0, 43.8, 42.0, 34.9, 33.8, 33.1, 26.5, 26.1. **IR (Diamond-ATR, neat):** $\tilde{\nu}$ / cm = 2919, 2846, 1720, 1608, 1432, 1281, 1175, 1102, 1018, 964, 862, 829, 764, 737, 707. **MS (EI, 70 eV):** m/z (%) = 378 (22), 281 (100), 221 (42). **HRMS (EI, 70 eV)** m/z: calcd for [$C_{24}H_{26}O_2S$] 378.1654, found 378.1647.

Synthesis of (*E*)-4-(1-(4-fluorophenyl)-3-(trimethylsilyl)allyl)benzonitrile (6m):

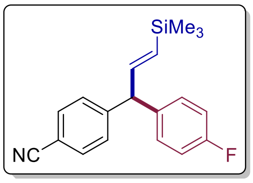

According to **GP3**, *p*-cyanobenzal diacetate (**1e**, 1.0 mmol, 1.0 equiv) reacted with *p*-fluorophenylzinc halide (**2d**, 1.0 mmol, 1.0 equiv, by *Method A*) in THF at room temperature for 12 h. Then, after addition of *E*-2-trimethylsilylalkenylzinc halide (1.5 mmol, 1.5 equiv, by *Method A*) and subsequent removal of THF in vacuum, toluene was added and the reaction mixture was heated at 120 °C for 3 h. After work-up, the residue was purified by column chromatography (SiO_2, *i*-hexane / ethyl acetate = 30 / 1) to give the desired product **6m** (43%, 133 mg, 0.43 mmol) as a waxy oil. **^{1}H-NMR (400 MHz, CDCl$_3$):** δ = 7.60 (d, *J* = 8.4 Hz, 2H), 7.24 (d, *J* = 8.4 Hz, 2H), 7.12−7.06 (m, 2H), 7.04−6.99 (m, 2H), 6.35 (dd, *J* = 18.4, 6.4 Hz, 1H), 5.62 (dd, *J* = 18.4, 1.4 Hz, 1H), 4.78 (d, *J* = 6.4 Hz, 1H), 0.08 (s, 9H). **^{13}C-NMR (100 MHz, CDCl$_3$):** δ = 161.6 (d, $^1J_{C\text{-}F}$ = 244 Hz), 148.6, 146.0, 137.5 (d, $^4J_{C\text{-}F}$ = 3.2 Hz), 133.7, 132.3, 130.0 (d, $^3J_{C\text{-}F}$ = 8.0 Hz), 129.4, 118.9, 115.5 (d, $^2J_{C\text{-}F}$ = 21.2 Hz), 110.3, 56.4, −1.3. **^{19}F-NMR (376 MHz, CDCl$_3$):** δ = −115.9. **IR (Diamond-ATR, neat):** $\tilde{\nu}$ / cm = 2954, 2896, 2227, 1604, 1505, 1411, 1246, 1222, 1158, 1096, 992, 831, 753, 714. **MS (EI, 70 eV):** m/z (%) = 309 (15), 217 (54), 208 (22), 190 (19), 153 (28), 73 (100). **HRMS (EI, 70 eV)** m/z: calcd for [$C_{19}H_{20}FNSi$] 309.1349, found 309.1352.

Synthesis of 4-(5-chloro-1-(3-chlorophenyl)pentyl)-1,2-dimethylbenzene (6n): (the precursor of an S1P receptor modulator)

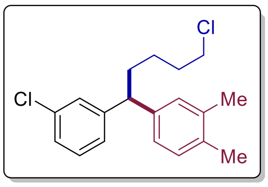

According to **GP3**, 3-chlorobenzal diacetate (**1m**, 3.0 mmol, 1.0 equiv) reacted with 3,4-dimethylphenylzinc halide (**2i**, 3.0 mmol, 1.0 equiv, by *Method A*) in THF at room temperature overnight. Then, after addition of (4-chlorobutyl)zinc halide (1.5 mmol, 1.5 equiv, by *Method B*) and subsequent removal of THF in vacuum, toluene was added and the reaction mixture was heated at 80 °C for 1 h. After work-up, the residue was purified by column chromatography (SiO_2, *i*-hexane) to give the desired product **6n** (91%, 873 mg, 2.73 mmol) as a colorless oil. **^{1}H-NMR (400 MHz, CDCl$_3$):** δ = 7.20 (t, *J* = 7.4 Hz, 2H), 7.16−7.11 (m, 2H), 7.06 (d, *J* = 8.0 Hz, 1H), 6.96 (d, *J* = 8.0 Hz, 2H), 3.80 (t, *J* = 7.8 Hz, 1H), 3.50 (t, *J* = 6.6 Hz, 2H), 2.23 (d, *J* = 5.6 Hz, 6H), 2.06−1.99 (m, 2H), 1.84−1.77 (m, 2H), 1.44−1.36 (m, 2H). **^{13}C-NMR (100 MHz,**

$CDCl_3$): δ = 147.4, 141.4, 136.7, 134.7, 134.2, 129.8, 129.7, 129.1, 127.8, 126.2, 125.9, 124.9, 50.6, 44.8, 34.8, 32.5, 25.3, 19.9, 19.3. **IR (Diamond-ATR, neat):** $\tilde{\nu}$ / cm = 2936, 2861, 1593, 1570, 1503, 1452, 1428, 1298, 1190, 1080, 998, 880, 819, 783, 759, 729, 693. **MS (EI, 70 eV):** m/z (%) = 320 (2), 229 (100), 194 (45), 193 (11), 179 (33), 178 (18). **HRMS (EI, 70 eV)** m/z: calcd for [$C_{19}H_{22}Cl_2$] 320.1099, found 320.1091.

Synthesis of (4-bromo-3-(3,3-dimethyl-1-phenylbutyl)phenoxy)(*tert*-butyl)dimethylsilane (6o):

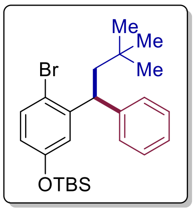

According to **GP3**, (2-bromo-5-((*tert*-butyldimethylsilyl)oxy)phenyl)methylene diacetate (**1n**, 5.0 mmol, 1.0 equiv) reacted with PhZnX (**2a**, 5.0 mmol, 1.0 equiv, by *Method A*) in THF at 60 °C for 3 h. Then, after addition of neopentylzinc halide (7.5 mmol, 1.5 equiv, by *Method A*) and subsequent removal of THF in vacuum, toluene was added and the reaction mixture was heated to reflux for 1 h. After work-up, the residue was purified by column chromatography (SiO_2, *i*-hexane / ethyl acetate = 50 / 1) to give the desired product **6o** (83%, 1.85 g, 4.15 mmol) as a waxy oil. **^{1}H-NMR (400 MHz, $CDCl_3$):** δ = 7.38−7.33 (m, 3H), 7.28−7.24 (m, 2H), 7.17−7.13 (m, 1H), 6.89 (d, *J* = 2.8 Hz, 1H), 6.52 (dd, *J* = 8.8, 2.8 Hz, 1H), 4.61 (t, *J* = 6.6 Hz, 1H), 2.08−1.98 (m, 2H), 0.95 (s, 9H), 0.87 (s, 9H), 0.15 (d, 6H). **^{13}C-NMR (100 MHz, $CDCl_3$):** δ = 155.0, 146.3, 145.5, 133.4, 128.3, 127.8, 126.0, 120.9, 119.5, 115.9, 49.5, 45.8, 31.7, 30.2, 25.7, 18.2, -4.4. **IR (Diamond-ATR, neat):** $\tilde{\nu}$ / cm = 2952, 2928, 2857, 1590, 1566, 1466, 1364, 1284, 1251, 1164, 1114, 982, 834, 779, 695. **MS (EI, 70 eV):** m/z (%) = 448 (51), 446 (47), 391 (78), 375 (48), 239 (57), 165 (40). **HRMS (EI, 70 eV)** m/z: calcd for [$C_{24}H_{35}BrOSi$] 446.1641, found 446.1635.

Synthesis of methyl 4-((*tert*-butyldimethylsilyl)oxy)-2-(3,3-dimethyl-1-phenylbutyl)benzoate (6p):

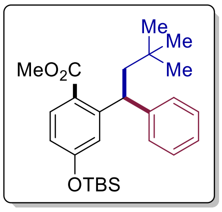

Magnesium turnings (152 mg, 6.25 mmol) and LiCl (212 mg, 5.0 mmol) were placed in a dry Schlenk tube equipped with a magnetic stirrer and a rubber septum under argon. Under vacuum (<1 mbar), the mixture was dried by a heating gun first at 260 °C for a few seconds, and then at 500 °C for 5 min until the vacuum degree had no change. After cooling and adding THF (10 mL), the mixture was activated by adding $BrCH_2CH_2Br$ (2.0 mol%) and TMSCl (1.5 mol%) followed by gently heating. The resulting mixture was stirred for 5 min to be cooled to room temperature. **6o** (1.85g, 4.15 mmol) was then added in one portion, and the reaction mixture was stirred under room temperature until the insertion was completed according to GC analysis. The resulting Grignard reagent was transferred with a syringe into a solution of methyl

cyanoformate (4.98 mmol, 1.2 equiv) in THF at −40 °C, and the reaction mixture was stirred at room temperature overnight. The reaction was finally quenched with $NaHCO_3$ (aq.). The aqueous phase was extracted with ethyl acetate (3×10 mL) and the combined organic layers were dried over $MgSO_4$, filtered and concentrated under reduced pressure. The residue was purified by column chromatography (SiO_2, *i*-hexane / ethyl acetate = 20 / 1) to give the desired product **6p** (71%, 1.26 g, 2.95 mmol) as a waxy oil. **^{1}H-NMR (400 MHz, $CDCl_3$):** δ = 7.72 (d, *J* = 8.8 Hz, 1H), 7.38−7.35 (m, 2H), 7.25−7.22 (m, 2H), 7.14−7.09 (m, 1H), 6.97 (d, *J* = 2.4 Hz, 1H), 6.63 (dd, *J* = 8.6, 2.6 Hz, 1H), 5.44 (t, *J* = 6.6 Hz, 1H), 3.89 (s, 3H), 2.06−2.04 (m, 2H), 0.96 (s, 9H), 0.84 (s, 9H), 0.18 (d, 6H). **^{13}C-NMR (100 MHz, $CDCl_3$):** δ = 168.3, 158.7, 150.6, 146.7, 132.5, 128.2, 127.9, 125.8, 122.3, 120.2, 117.3, 51.8, 50.0, 40.9, 31.7, 30.2, 25.6, 18.3, -4.3. **IR (Diamond-ATR, neat):** $\tilde{\nu}$ / cm = 3027, 2950, 2858, 1715, 1598, 1563, 1491, 1433, 1363, 1238, 1188, 1122, 1085, 987, 837, 780, 697. **MS (EI, 70 eV):** m/z (%) = 426 (12), 369 (52), 355 (25), 337 (77), 313 (22), 281 (17), 207 (15), 71 (59), 57 (100). **HRMS (EI, 70 eV)** m/z: calcd for [$C_{26}H_{38}O_3Si$] 426.2590, found 426.2589.

Synthesis of a FLAP inhibitor (6q):

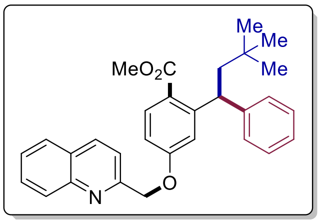

The mixture of **6p** (1.26 g, 2.95 mmol, 1.0 equiv), water (266 mg, 14.8 mmol, 5.0 equiv), K_2CO_3 (490 mg, 3.54 mmol, 1.2 equiv) and ethanol (15 mL) was refluxed overnight. Then, the solvent was concentrated under reduced pressure. Water (10 mL) was added and the aqueous phase was extracted with ethyl acetate (3×10 mL). The combined organic layers were dried over $MgSO_4$, filtered and concentrated under reduced pressure to give the desilylation product in pure form.

The above-obtained product was mixed with 2-(chloromethyl)quinoline hydrochloride (758 mg, 3.54 mmol, 1.2 equiv), K_2CO_3 (1.22 g, 8.85 mmol, 3.0 equiv) and acetone (15 mL). The mixture was refluxed for 6 h and then acetone was removed under reduced pressure. Water (10 mL) was added and the aqueous phase was extracted with ethyl acetate (3×10 mL). The combined organic layers were dried over $MgSO_4$, filtered and concentrated under reduced pressure to give the desired product **6q** (92%, 1.23 g, 2.71 mmol) as a waxy oil. **^{1}H-NMR (400 MHz, $CDCl_3$):** δ = 8.17−8.11 (m, 2H), 7.84−7.74 (m, 3H), 7.61−7.55 (m, 2H), 7.31−7.29 (m, 2H), 7.18 (d, *J* = 2.8 Hz, 1H), 7.12−7.03 (m, 3H), 6.82 (dd, *J* = 8.8, 2.4 Hz, 1H), 5.45 (t, *J* = 6.8 Hz, 1H), 5.42 (s, 2H), 3.88 (s, 3H), 2.03−1.94 (m, 2H), 0.74 (s, 9H). **^{13}C-NMR (100 MHz, $CDCl_3$):** δ = 168.1, 160.8, 157.3, 150.9, 147.6, 146.5, 137.1, 132.7, 129.9, 128.9, 128.2, 127.8, 127.7, 127.6, 126.7, 125.7, 122.0, 119.1, 114.8, 112.2, 71.3, 51.9, 49.8, 40.9, 31.6, 30.1. **IR (Diamond-ATR, neat):** $\tilde{\nu}$ / cm = 3026, 2948, 2865, 1710, 1597, 1568, 1492, 1429, 1364, 1312, 1234, 1188, 1125, 1087, 1040, 908, 824, 724, 697. **MS (EI, 70 eV):** m/z (%) = 453 (1), 396

(20), 382 (12), 364 (100), 311 (23), 223 (14), 142 (59). **HRMS (EI, 70 eV)** m/z: calcd for [$C_{30}H_{31}NO_3$] 453.2304, found 453.2298.

Synthesis of 4,4'-(2-methyldecane-1,1-diyl)bis(methoxybenzene) (8a):

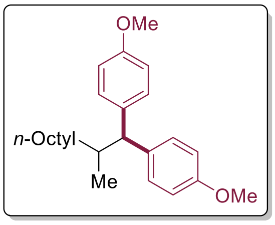

According to **GP4**, 4-$MeOC_6H_4ZnX$ (**2b**, 3.0 mmol, 3.0 equiv, by *Method A*) reacted with 2-methyldecane-1,1-diyl diacetate (**7a**, 1.0 mmol, 1.0 equiv) at 80 °C overnight. After work-up, the residue was purified by column chromatography (SiO_2, *i*-hexane / ethyl acetate = 50 / 1) to give the desired product **8a** (75%, 276 mg, 0.75 mmol) as a colorless oil. **^{1}H-NMR (400 MHz, $CDCl_3$):** δ = 7.19−7.16 (m, 4H), 6.81 (dd, *J* = 8.8, 2.8 Hz, 4H), 3.76 (s, 6H), 3.43 (d, *J* = 10.8 Hz, 1H), 2.28−2.22 (m, 1H), 1.38−1.16 (m, 14H), 0.89 (t, *J* = 6.6 Hz, 3H), 0.83 (d, *J* = 6.4 Hz, 3H). **^{13}C-NMR (100 MHz, $CDCl_3$):** δ = 157.5, 137.5, 137.3, 128.8, 128.7, 113.7, 113.7, 57.5, 55.1, 36.6, 34.8, 31.9, 29.8, 29.6, 29.3, 26.8, 22.7, 18.2, 14.1. **IR (Diamond-ATR, neat):** $\tilde{\nu}$ / cm = 2923, 2853, 1736, 1608, 1582, 1508, 1463, 1376, 1300, 1244, 1174, 1036, 819, 770, 722. **MS (EI, 70 eV):** m/z (%) = 368 (1), 228 (16), 227 (100). **HRMS (EI, 70 eV)** m/z: calcd for [$C_{25}H_{36}O_2$] 368.2715, found 368.2712.

Synthesis of 4,4'-(2-methyldecane-1,1-diyl)bis(N,N-dimethylaniline) (8b):

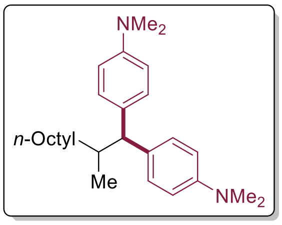

According to **GP4**, 4-(dimethylamino)phenylzinc halide (3.0 mmol, 3.0 equiv, by *Method A*) reacted with 2-methyldecane-1,1-diyl diacetate (**7a**, 1.0 mmol, 1.0 equiv) at 80 °C overnight. After work-up, the residue was purified by column chromatography (SiO_2, *i*-hexane / ethyl acetate = 10 / 1) to give the desired product **8b** (68%, 268 mg, 0.68 mmol) as a waxy oil. **^{1}H-NMR (400 MHz, $CDCl_3$):** δ = 7.14−7.11 (m, 4H), 6.67 (dd, *J* = 8.6, 2.6 Hz, 4H), 3.35 (d, *J* = 10.4 Hz, 1H), 2.89 (s, 12H), 2.28−2.19 (m, 1H), 1.45−1.40 (m, 2H), 1.32−1.16 (m, 12H), 0.89 (t, *J* = 7.0 Hz, 3H), 0.84 (d, *J* = 6.4 Hz, 3H). **^{13}C-NMR (100 MHz, $CDCl_3$):** δ = 148.7, 148.6, 134.2, 134.0, 128.5, 128.4, 112.9, 112.8, 57.2, 40.8, 36.5, 35.0, 31.9, 29.8, 29.7, 29.3, 27.0, 22.7, 18.3, 14.1. **IR (Diamond-ATR, neat):** $\tilde{\nu}$ / cm = 2918, 2848, 2791, 1614, 1516, 1481, 1442, 1344, 1226, 1166, 1126, 1061, 949, 800, 753, 722. **MS (EI, 70 eV):** m/z (%) = 394 (32), 253 (100), 237 (16), 126 (10). **HRMS (EI, 70 eV)** m/z: calcd for [$C_{27}H_{42}N_2$] 394.3348, found 394.3345.

Synthesis of 4,4'-(3-phenylpropane-1,1-diyl)bis(N,N-dimethylaniline) (8c):

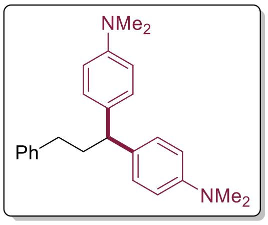

According to **GP4**, 4-(dimethylamino)phenylzinc halide (3.0 mmol, 3.0 equiv, by *Method A*) reacted with 3-phenylpropane-1,1-diyl diacetate (**7b**, 1.0 mmol, 1.0 equiv) at 80 °C overnight. After work-up, the residue was purified by column chromatography (SiO_2, *i*-hexane / ethyl acetate = 10 / 1) to give the desired product **8c** (74%, 265 mg, 0.74 mmol) as a waxy oil. **^{1}H-NMR (400 MHz, $CDCl_3$):** δ = 7.28 (t, *J* = 7.4 Hz, 2H), 7.20−7.17 (m, 3H), 7.15−7.12 (m, 4H), 6.70 (d, *J* = 8.4 Hz, 4H), 3.78 (t, *J* = 7.8 Hz, 1H), 2.92 (s, 12H), 2.62−2.58 (m, 2H), 2.36−2.30 (m, 2H). **^{13}C-NMR (100 MHz, $CDCl_3$):** δ = 148.9, 142.6, 133.9, 128.4, 128.3, 128.2, 125.5, 112.9, 48.7, 40.8, 37.7, 34.3. **IR (Diamond-ATR, neat):** $\tilde{\nu}$ / cm = 3023, 2921, 2854, 2794, 1870, 1611, 1514, 1478, 1442, 1340, 1221, 1162, 1128, 1058, 946, 810, 747, 698. **MS (EI, 70 eV):** m/z (%) = 358 (8), 253 (100), 237 (20), 165 (36). **HRMS (EI, 70 eV)** m/z: calcd for [$C_{25}H_{30}N_2$] 358.2409, found 358.2403.

Synthesis of 5,5'-(3-phenylpropane-1,1-diyl)bis(benzo[d][1,3]dioxole) (8d):

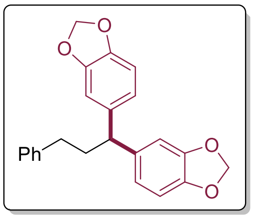

According to **GP4**, benzo[d][1,3]dioxol-5-ylzinc halide (3.0 mmol, 3.0 equiv, by *Method A*) reacted with 3-phenylpropane-1,1-diyl diacetate (**7b**, 1.0 mmol, 1.0 equiv) at 80 °C overnight. After work-up, the residue was purified by column chromatography (SiO_2, *i*-hexane / ethyl acetate = 30 / 1) to give the desired product **8d** (54%, 195 mg, 0.54 mmol) as a waxy oil. **^{1}H-NMR (400 MHz, $CDCl_3$):** δ = 7.30 (t, *J* = 7.4 Hz, 2H), 7.22−7.15 (m, 3H), 6.78−7.71 (m, 6H), 5.92 (s, 4H), 3.77 (t, *J* = 7.8 Hz, 1H), 2.58 (t, *J* = 7.8 Hz, 2H), 2.32−2.26 (m, 2H). **^{13}C-NMR (100 MHz, $CDCl_3$):** δ = 147.7, 145.8, 142.0, 138.9, 128.4, 128.3, 125.8, 120.5, 108.1, 108.0, 100.8, 49.9, 37.4, 34.0. **IR (Diamond-ATR, neat):** $\tilde{\nu}$ / cm = 3024, 2886, 2775, 1846, 1604, 1500, 1481, 1436, 1363, 1233, 1182, 1122, 1096, 1035, 930, 865, 807, 736, 698. **MS (EI, 70 eV):** m/z (%) = 360 (10), 255 (100), 225 (12), 139 (22). **HRMS (EI, 70 eV)** m/z: calcd for [$C_{23}H_{20}O_4$] 360.1362, found 360.1358.

Synthesis of (*E*)-4,4'-(3-phenylprop-2-ene-1,1-diyl)bis(N,N-dimethylaniline) (8c'):

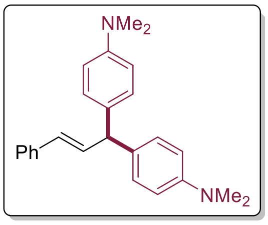

According to **GP4**, 4-(dimethylamino)phenylzinc halide (3.0 mmol, 3.0 equiv, by *Method A*) reacted with (*E*)-3-phenylprop-2-ene-1,1-diyl diacetate (**7b'**, 1.0 mmol, 1.0 equiv) at 80 °C overnight. After work-up, the residue was purified by column chromatography (SiO_2, *i*-hexane / ethyl acetate = 10 / 1) to give the desired product **8c'** (80%, 285 mg, 0.80 mmol) as an orange solid. **M.p.:** 104−105 °C. **^{1}H-NMR (400 MHz, $CDCl_3$):** δ = 7.40 (d, *J* = 7.2 Hz, 2H), 7.31 (t, *J* = 7.4 Hz, 2H), 7.22 (t, *J* = 7.4 Hz, 1H), 7.15 (d, *J* = 8.4 Hz, 4H), 6.75−6.72 (m, 4H), 6.68 (t, *J* = 8.0 Hz, 1H), 6.36 (d, *J* = 15.6 Hz, 1H), 4.77 (d, *J* = 7.6 Hz, 1H),

2.95 (s, 12H). **^{13}C-NMR (100 MHz, $CDCl_3$):** δ = 149.1, 137.7, 134.0, 132.2, 130.1, 129.2, 128.4, 126.9, 126.2, 112.7, 52.3, 40.8. **IR (Diamond-ATR, neat):** $\tilde{\nu}$ / cm = 2884, 1613, 1517, 1446, 1349, 1228, 1164, 1124, 1065, 969, 946, 807, 752, 691. **MS (EI, 70 eV):** m/z (%) = 356 (17), 299 (15), 281 (38), 267 (16), 225 (75), 207 (100), 191 (25). **HRMS (EI, 70 eV)** m/z: calcd for [$C_{25}H_{28}N_2$] 356.2252, found 356.2247.

Synthesis of (*E*)-5,5'-(3-phenylprop-2-ene-1,1-diyl)bis(benzo[d][1,3]dioxole) (8d'):

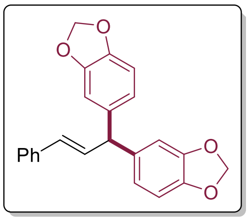

According to **GP4**, benzo[d][1,3]dioxol-5-ylzinc halide (3.0 mmol, 3.0 equiv, by *Method A*) reacted with (*E*)-3-phenylprop-2-ene-1,1-diyl diacetate (**7b'**, 1.0 mmol, 1.0 equiv) at 80 °C overnight. After work-up, the residue was purified by column chromatography (SiO_2, *i*-hexane / ethyl acetate = 30 / 1) to give the desired product **8d'** (52%, 186 mg, 0.52 mmol) as a waxy oil. **^{1}H-NMR (400 MHz, $CDCl_3$):** δ = 7.38 (d, *J* = 7.6 Hz, 2H), 7.31 (t, *J* = 7.6 Hz, 2H), 7.23 (t, *J* = 7.2 Hz, 1H), 6.79−6.71 (m, 6H), 6.59 (dd, *J* = 15.8, 3.4 Hz, 1H), 6.34 (d, *J* = 16.0 Hz, 1H), 5.94 (s, 4H), 4.74 (d, *J* = 7.6 Hz, 1H). **^{13}C-NMR (100 MHz, $CDCl_3$):** δ = 147.7, 146.1, 137.5, 137.1, 132.5, 131.2, 128.5, 127.3, 126.3, 121.4, 109.0, 108.1, 100.9, 53.3. **IR (Diamond-ATR, neat):** $\tilde{\nu}$ / cm = 3023, 2887, 2774, 1738, 1702, 1598, 1500, 1481, 1438, 1352, 1239, 1180, 1097, 1034, 968, 930, 868, 810, 744, 693. **MS (EI, 70 eV):** m/z (%) = 358 (62), 328 (21), 236 (100), 206 (34), 178 (82), 165 (50). **HRMS (EI, 70 eV)** m/z: calcd for [$C_{23}H_{28}O_4$] 358.1205, found 358.1199.

Synthesis of cholesterylmethylene bis(N,N-dimethylaniline) (8e):

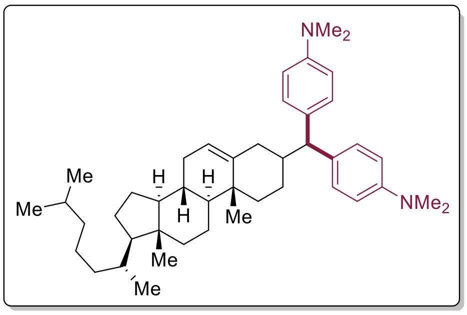

According to **GP4**, 4-(dimethylamino)phenylzinc halide (3.0 mmol, 3.0 equiv, by *Method A*) reacted with the cholesterylmethylene diacetate (**7c**, 1.0 mmol, 1.0 equiv) at 80 °C overnight. After work-up, the residue was purified by column chromatography (SiO_2, *i*-hexane / ethyl acetate = 10 / 1) to give the desired product **8e** (72%, 448 mg, 0.72 mmol) as a white solid. **M.p.:** 225−226 °C. **^{1}H-NMR (400 MHz, $CDCl_3$):** δ = 7.14−7.09 (m, 4H), 6.68−6.64 (m, 4H), 5.19 (d, *J* = 4.0 Hz, 1H), 3.33 (d, *J* = 11.2 Hz, 1H), 2.88 (s, 12H), 2.06−1.78 (m, 7H), 1.56−1.02 (m, 22H), 0.97 (s, 3H), 0.92 (d, *J* = 6.4 Hz, 3H), 0.88 (dd, *J* = 6.4, 1.6 Hz, 6H), 0.67 (s, 3H). **^{13}C-NMR (100 MHz, $CDCl_3$):** δ = 148.7, 143.4, 133.6, 133.3, 128.4, 119.4, 112.9, 112.9, 57.5, 56.8, 56.1, 50.5, 43.1, 42.3, 40.8, 40.8, 39.8, 39.6, 39.5, 38.4, 37.0, 36.2, 35.8, 31.9, 31.8, 28.2, 28.0, 24.3, 23.8, 22.8, 22.6, 20.9, 19.6, 18.7, 11.8. **IR (Diamond-ATR, neat):** $\tilde{\nu}$ / cm = 2931, 2861, 1610, 1514, 1443, 1340, 1222, 1163, 1134, 1060, 948, 852, 801, 755. **MS (EI, 70 eV):**

m/z (%) = 622 (4), 254 (21), 253 (100). **HRMS (EI, 70 eV)** m/z: calcd for [$C_{44}H_{66}N_2$] 622.5226, found 622.5243.

Synthesis of 3-(4-methoxyphenyl)isobenzofuran-1(3*H*)-one (10):

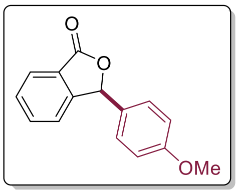

According to **GP1**, 4-methoxyphenylzinc halide (**2b**, 3.0 mmol, 3.0 equiv, by *Method A*) was mixed with an intramolecular diacetate (**9**, 1.0 mmol, 1.0 equiv) in THF. Then, THF was removed in vacuum and toluene was added. The toluene solution was heated at 120 °C for 3 h. After work-up, the residue was purified by column chromatography (SiO_2, *i*-hexane / ethyl acetate = 5 / 1) to give product **10** (74%, 177 mg, 0.74 mmol) as a white solid. **M.p.:** 116−117 °C. **^{1}H-NMR (400 MHz, $CDCl_3$):** δ = 7.95 (d, *J* = 7.6 Hz, 1H), 7.65 (td, *J* = 7.4, 0.8 Hz, 1H), 7.55 (t, *J* = 7.6 Hz, 1H), 7.31 (d, *J* = 7.6 Hz, 1H), 7.19−7.15 (m, 2H), 6.90−6.87 (m, 2H), 6.37 (s, 1H), 3.80 (s, 3H). **^{13}C-NMR (100 MHz, $CDCl_3$):** δ = 170.5, 160.3, 149.7, 134.2, 129.2, 128.8, 128.2, 125.8, 125.5, 122.9, 114.2, 82.7, 55.3. **IR (Diamond-ATR, neat):** $\tilde{\nu}$ / cm = 2968, 1750, 1612, 1513, 1465, 1286, 1253, 1210, 1176, 1096, 1072, 1028, 963, 820, 776, 738, 686. **MS (EI, 70 eV):** m/z (%) = 240 (100), 209 (16), 152 (31), 135 (42), 104 (38). **HRMS (EI, 70 eV)** m/z: calcd for [$C_{15}H_{12}O_3$] 240.0786, found 240.0785.

Synthesis of 1-phenyl-1,2-dihydronaphthalene (11):

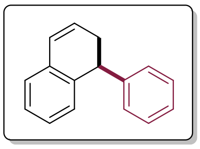

According to **GP3**, 2-allylbenzal diacetate (**1o**, 1.0 mmol, 1.0 equiv) reacted with PhZnX (**2a**, 1.0 mmol, 1.0 equiv, by *Method A*) in THF at 60 °C for 3 h to form **4d**. Then, after addition of 1-penten-5-ylzinc halide (1.5 mmol, 1.5 equiv, by *Method A*) and subsequent removal of THF in vacuum, toluene was added and the reaction mixture was heated at 80 °C for 1 h. After work-up, the residue was purified by column chromatography (SiO_2, *i*-hexane) to give product **11** (58%, 120 mg, 0.58 mmol) as a waxy oil. **^{1}H-NMR (400 MHz, $CDCl_3$):** δ = 7.35−7.30 (m, 2H), 7.27−7.24 (m, 3H), 7.22−7.18 (m, 1H), 7.15−7.08 (m, 2H), 6.83 (d, *J* = 7.6 Hz, 1H), 6.56 (d, *J* = 9.6 Hz, 1H), 6.04−6.00 (m, 1H), 4.15 (t, *J* = 8.8 Hz, 1H), 2.73−2.59 (m, 2H). **^{13}C-NMR (100 MHz, $CDCl_3$):** δ = 144.4, 137.8, 134.1, 128.4, 128.4, 128.0, 127.8, 127.2, 127.2, 126.8, 126.4, 126.1, 43.8, 31.9. **IR (Diamond-ATR, neat):** $\tilde{\nu}$ / cm = 3026, 2927, 2827, 1597, 1492, 1451, 1428, 1306, 1156, 1076, 1031, 942, 886, 781, 745, 698. **MS (EI, 70 eV):** m/z (%) = 206 (100), 205 (36), 191 (41), 178 (28), 128 (55). **HRMS (EI, 70 eV)** m/z: calcd for [$C_{16}H_{14}$] 206.1096, found 206.1090.

6. ICP-MS analysis

ICP-MS analysis was carried out on solvents (THF and toluene), **1a**, **2a** (solvent was removed), and the final reaction mixture (after the reaction of **1a** with **2a** was completed). Samples for solvents were firstly evaporated and the residue was redissolved in diluted nitric acid (3 w/v%) for analysis. Sample of **1a**, **2a**, or their final reaction mixture was firstly treated with nitric acid. After digestion, the sample was dried and the residue was redissolved in diluted nitric acid (3 w/v%) for analysis. As a result, ***Fe***, ***Co***, ***Ni***, ***Cu***, ***Ru***, ***Rh***, ***Pd***, ***Ag***, ***Ir***, ***Pt***, and ***Au*** were found to be less than 1 ppb (within the detection limit).

7. Copies of NMR Spectra

NMR Spectra of **1i, 1n, 1o, and 7a**

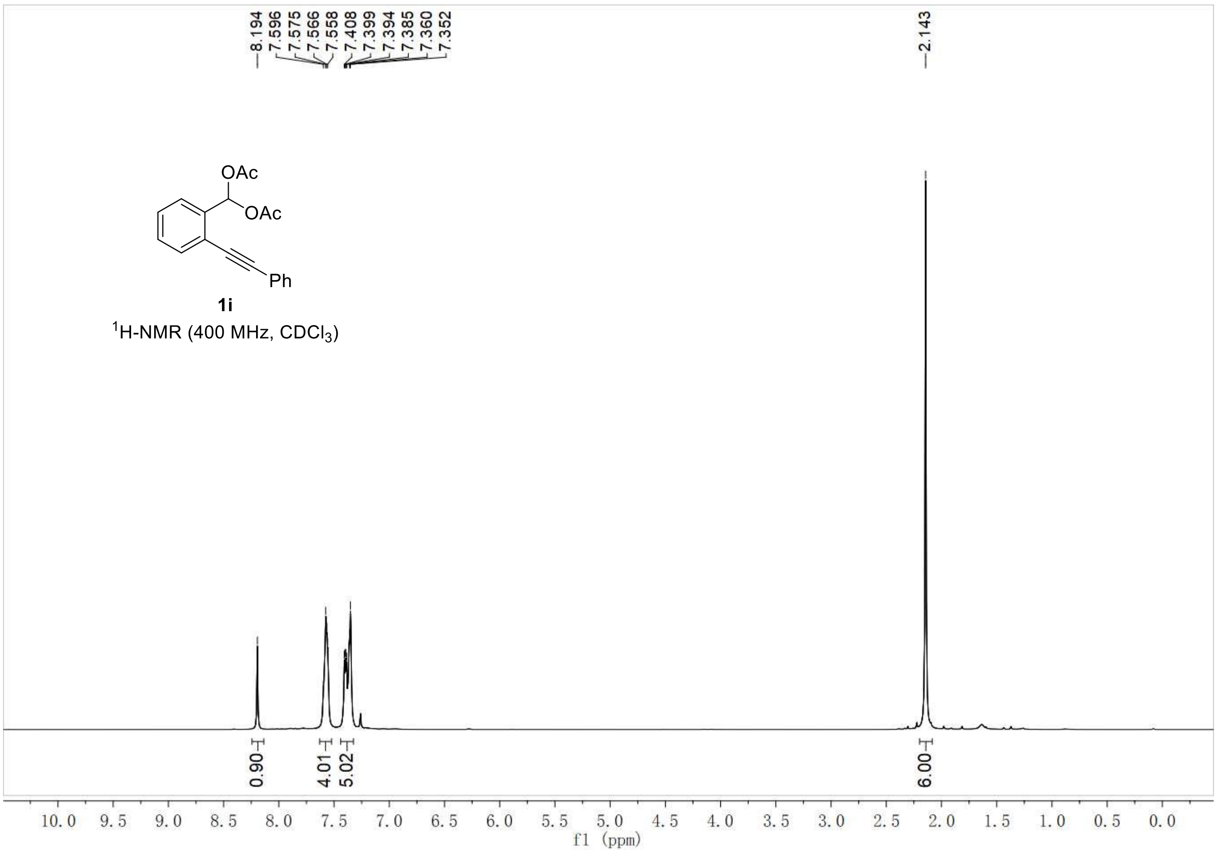

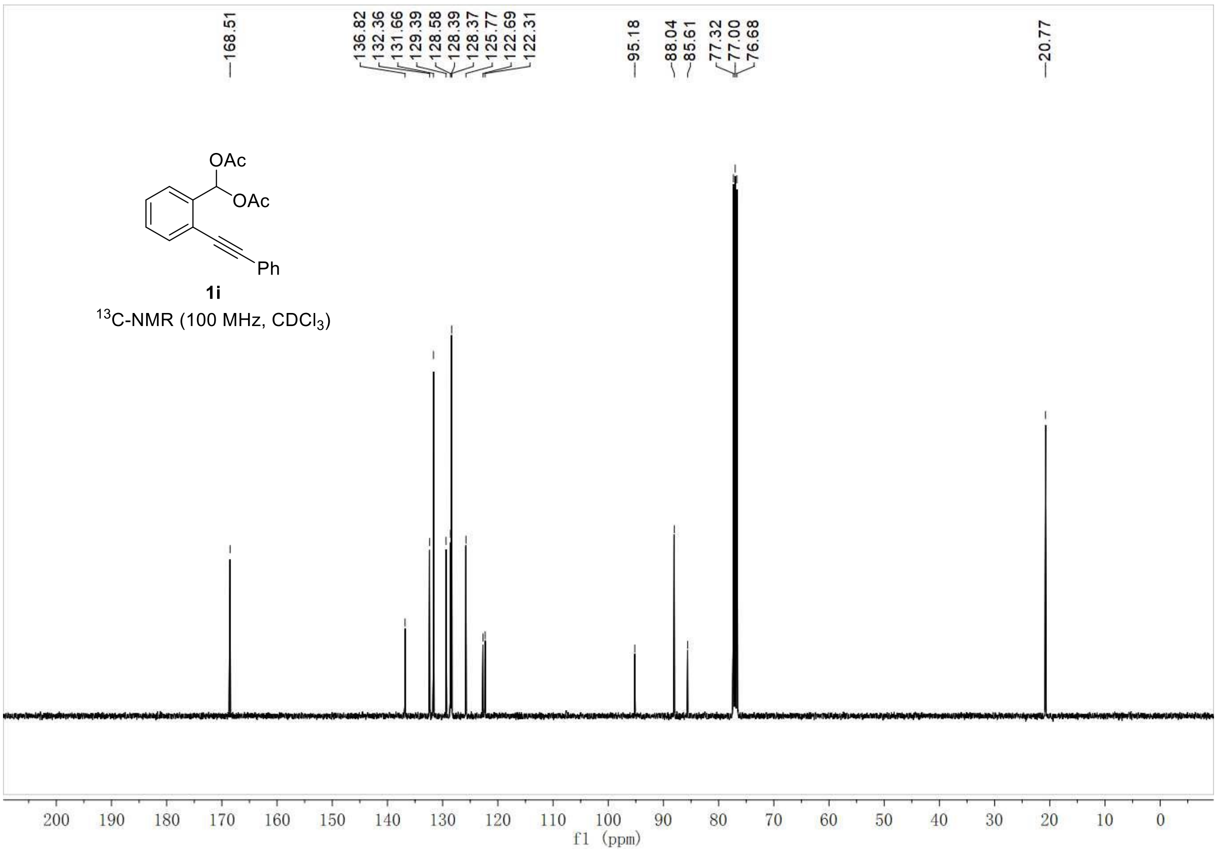

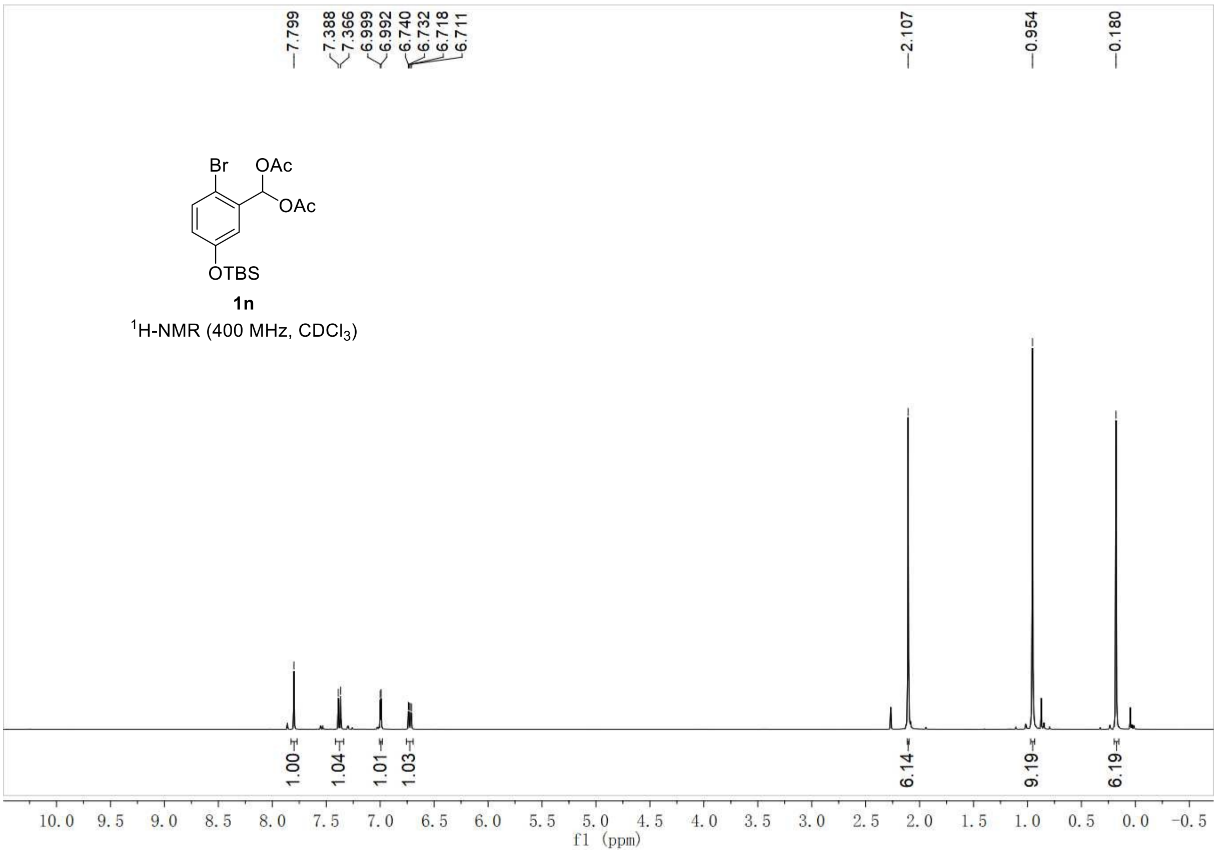
7.799
7.388
7.366
6.999
6.992
6.740
6.732
6.718
6.711
2.107
0.954
0.180
Br
OAc
OAc
OTBS
1n
^{1}H-NMR (400 MHz, $CDCl_3$)
1.00
1.04
1.01
1.03
6.14
9.19
6.19
10.0 9.5 9.0 8.5 8.0 7.5 7.0 6.5 6.0 5.5 5.0 4.5 4.0 3.5 3.0 2.5 2.0 1.5 1.0 0.5 0.0 -0.5
f1 (ppm)

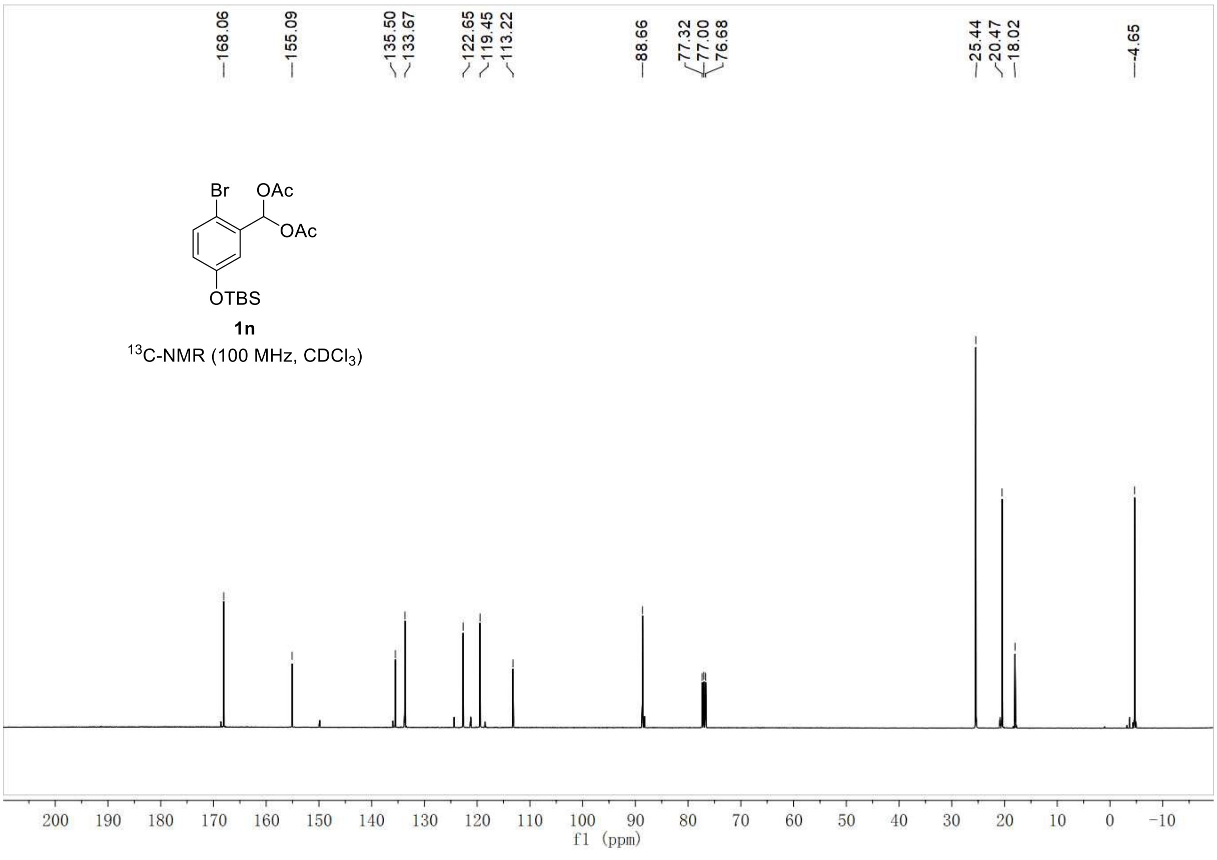
168.06
155.09
135.50
133.67
122.65
119.45
113.22
88.66
77.32
77.00
76.68
25.44
20.47
18.02
-4.65
Br
OAc
OAc
OTBS
1n
^{13}C-NMR (100 MHz, $CDCl_3$)
200 190 180 170 160 150 140 130 120 110 100 90 80 70 60 50 40 30 20 10 0 -10
f1 (ppm)

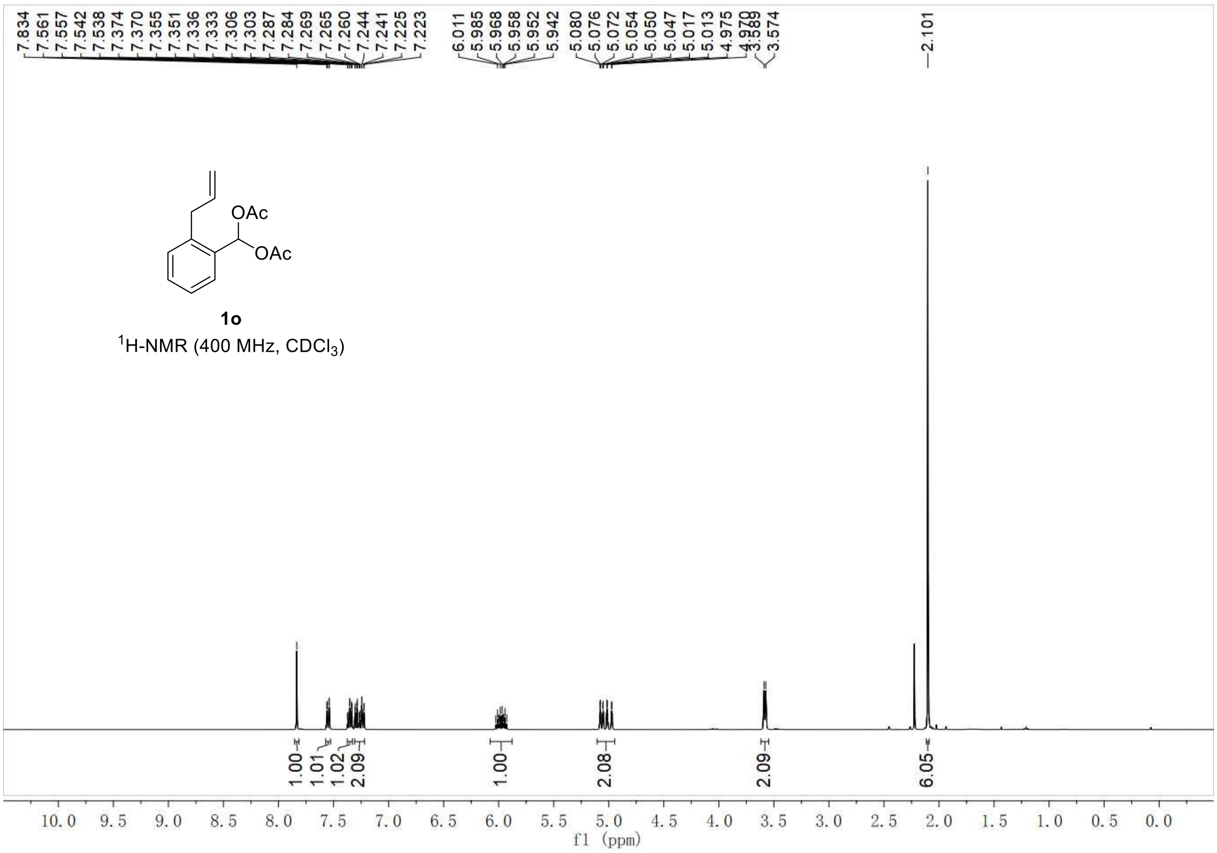
OAc
OAc
1o
1H-NMR (400 MHz, CDCl3)
f1 (ppm)

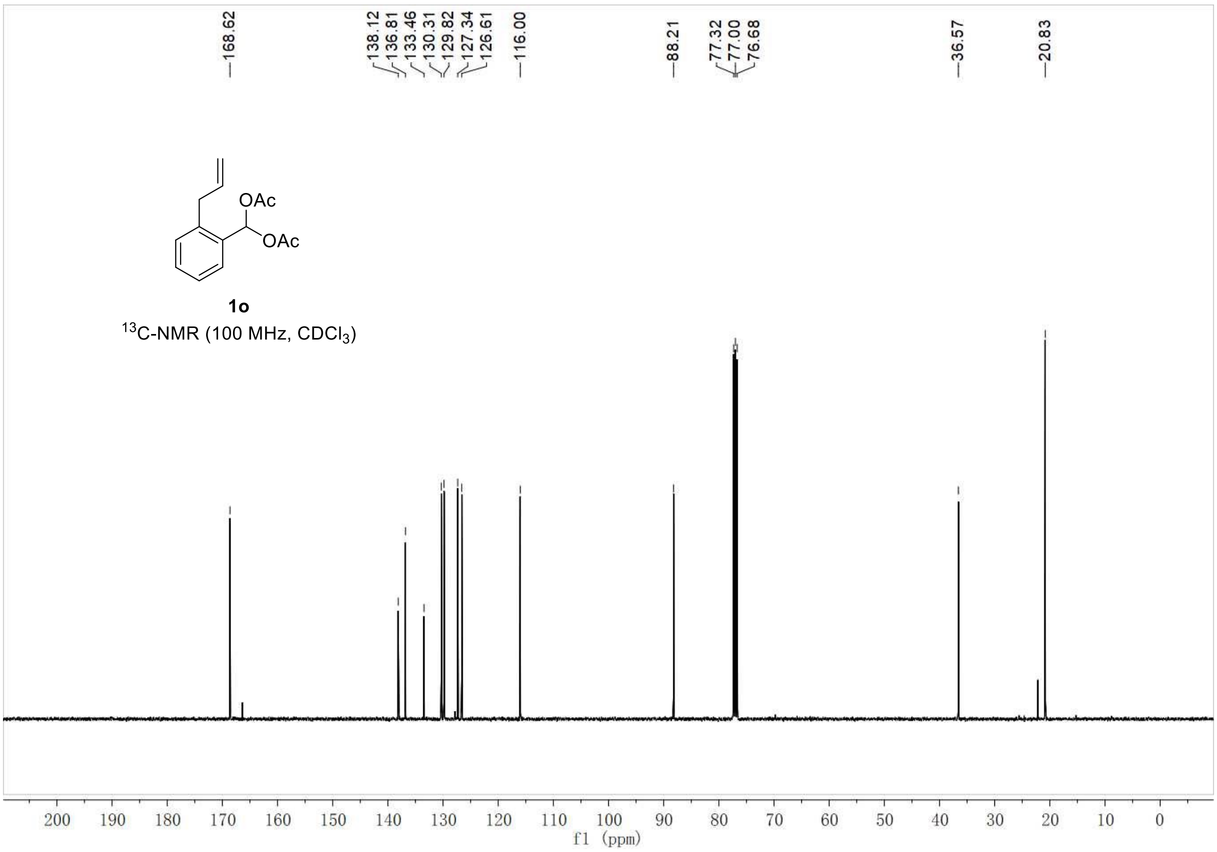
OAc
OAc
1o
13C-NMR (100 MHz, CDCl3)
f1 (ppm)

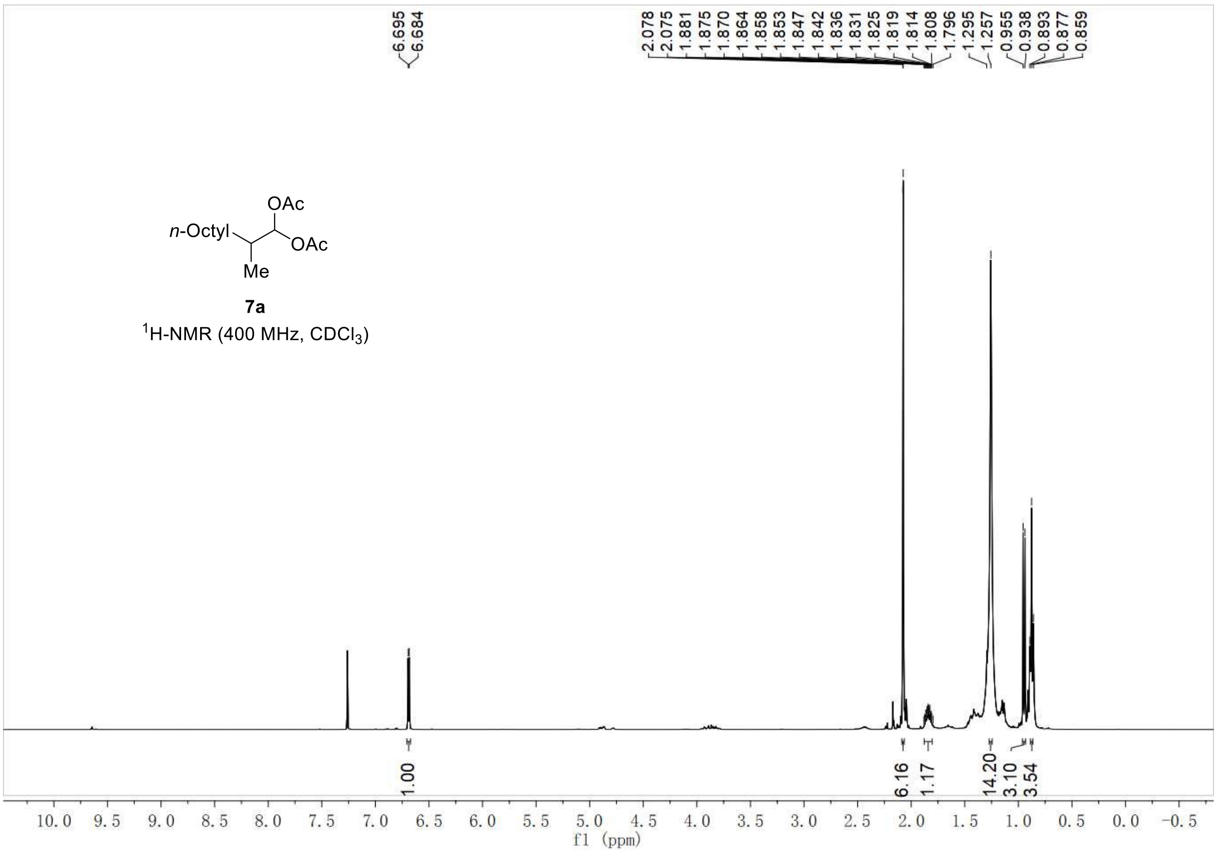
6.695
6.684
2.078
2.075
1.881
1.875
1.870
1.864
1.858
1.853
1.847
1.842
1.836
1.831
1.825
1.819
1.814
1.808
1.796
1.295
1.257
0.955
0.938
0.893
0.877
0.859
OAc
n-Octyl
OAc
Me
7a
1H-NMR (400 MHz, CDCl3)
1.00
6.16
1.17
14.20
3.10
3.54
f1 (ppm)

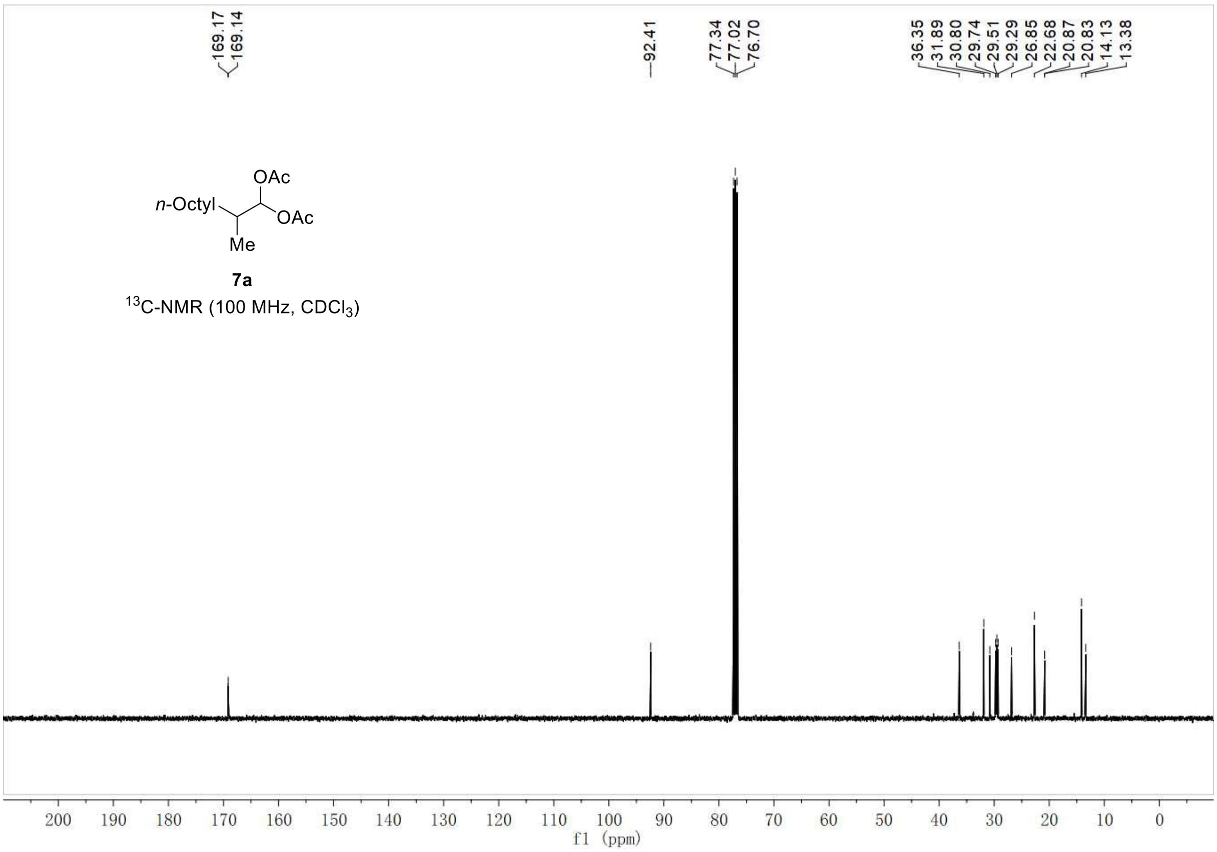
169.17
169.14
92.41
77.34
77.02
76.70
36.35
31.89
30.80
29.74
29.51
29.29
26.85
22.68
20.87
20.83
14.13
13.38
OAc
n-Octyl
OAc
Me
7a
13C-NMR (100 MHz, CDCl3)
f1 (ppm)

NMR Spectra of **4a**

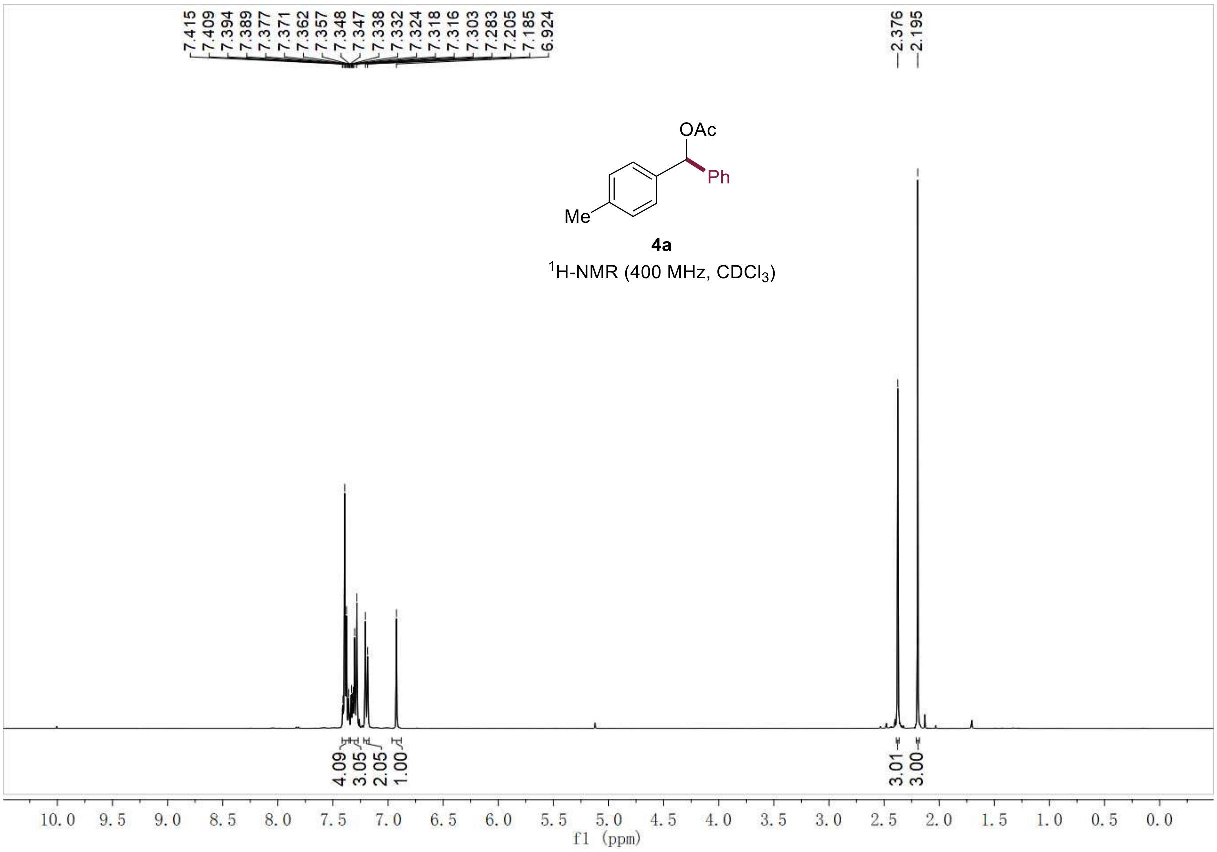

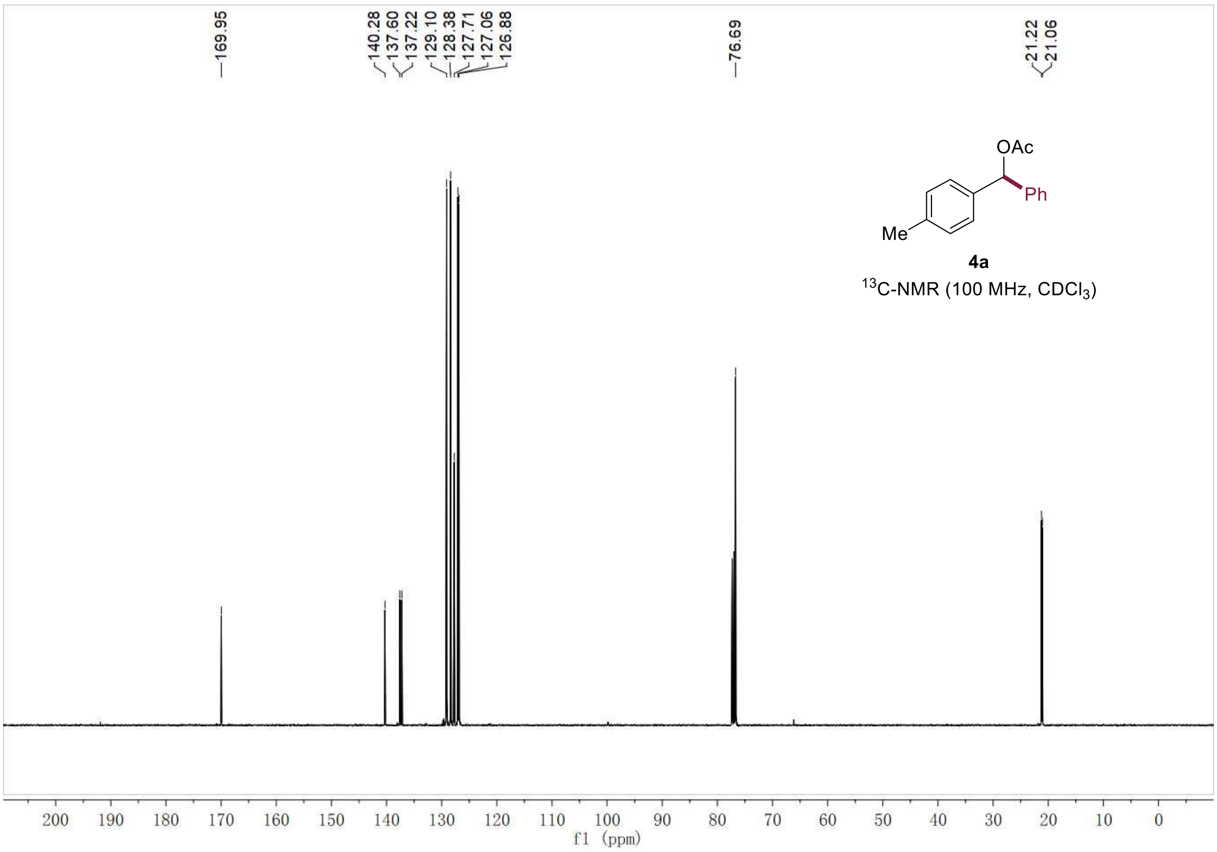

NMR Spectra of **3a-3o**

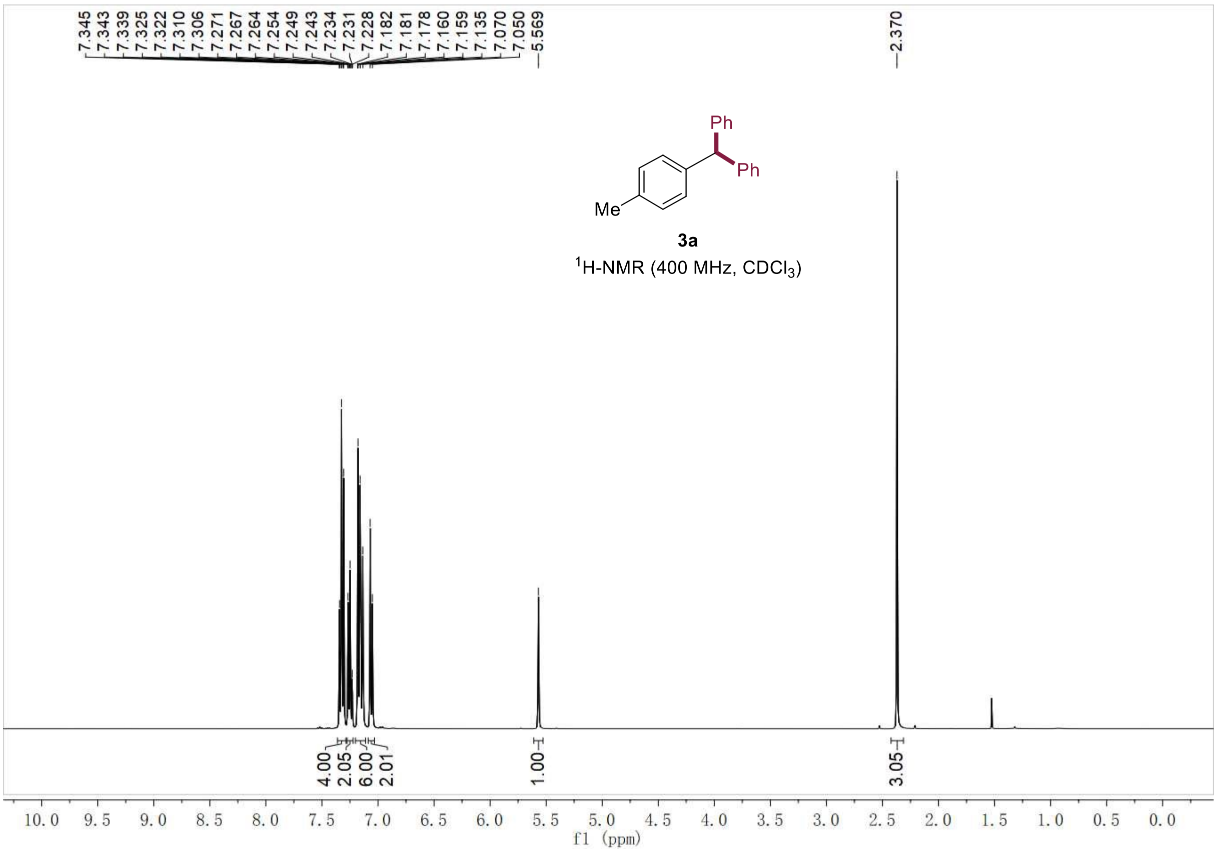

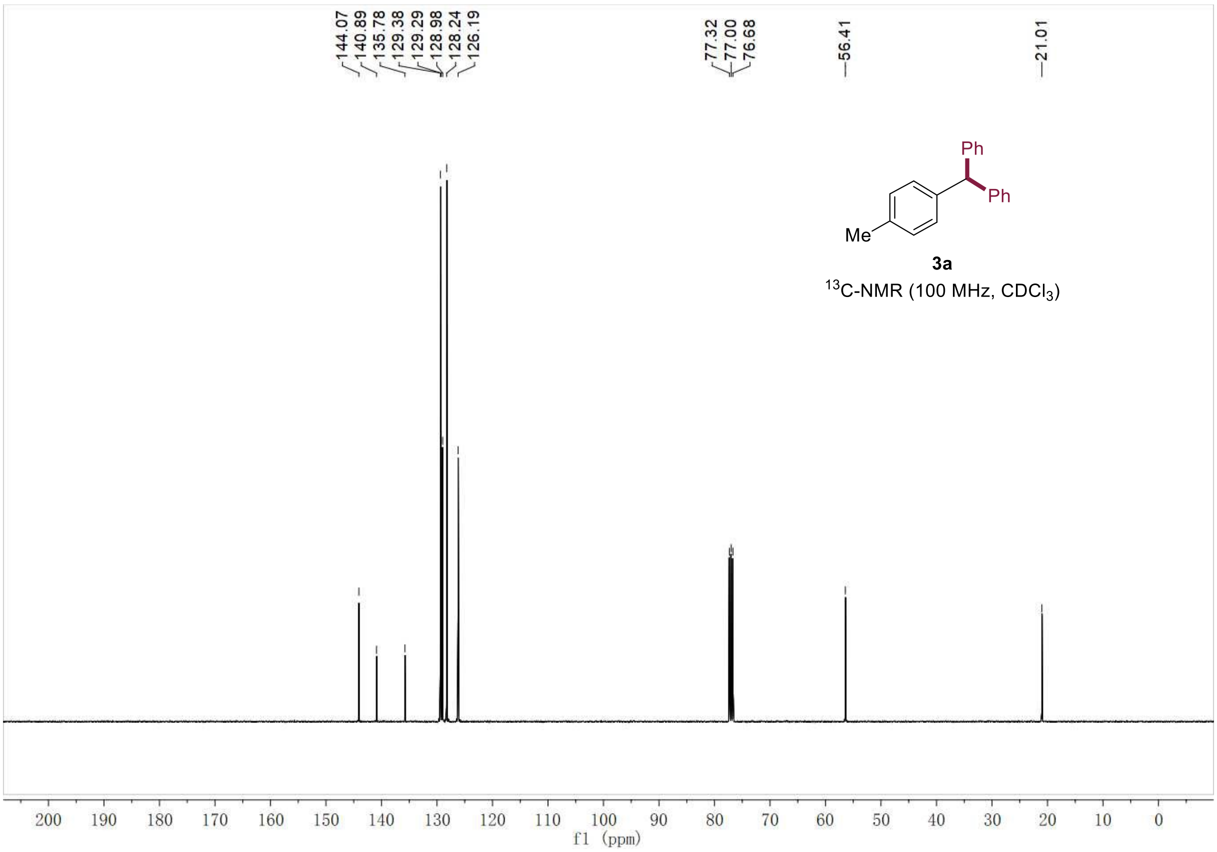

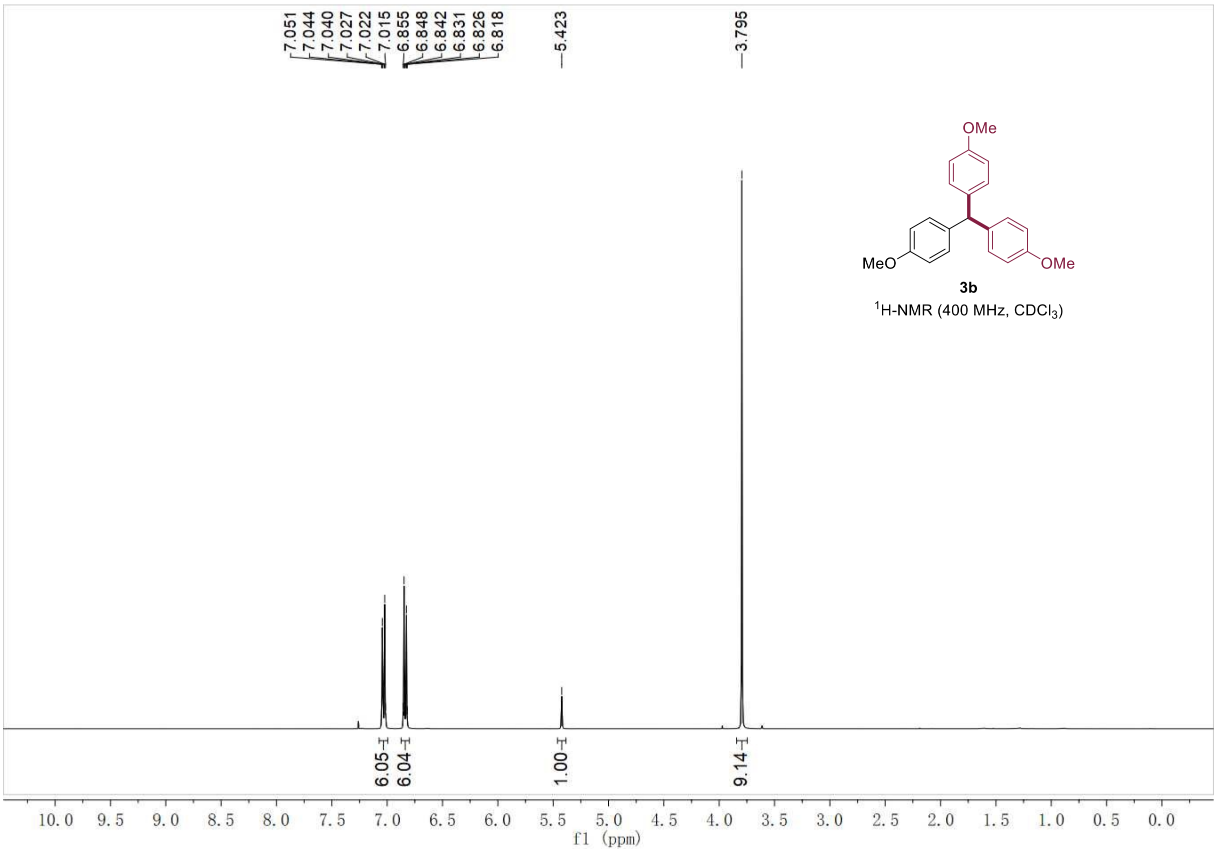
7.051
7.044
7.040
7.027
7.022
7.015
6.855
6.848
6.842
6.831
6.826
6.818
5.423
3.795
OMe
MeO
OMe
3b
1H-NMR (400 MHz, CDCl3)
6.05
6.04
1.00
9.14
10.0
9.5
9.0
8.5
8.0
7.5
7.0
6.5
6.0
5.5
5.0
4.5
4.0
3.5
3.0
2.5
2.0
1.5
1.0
0.5
0.0
f1 (ppm)

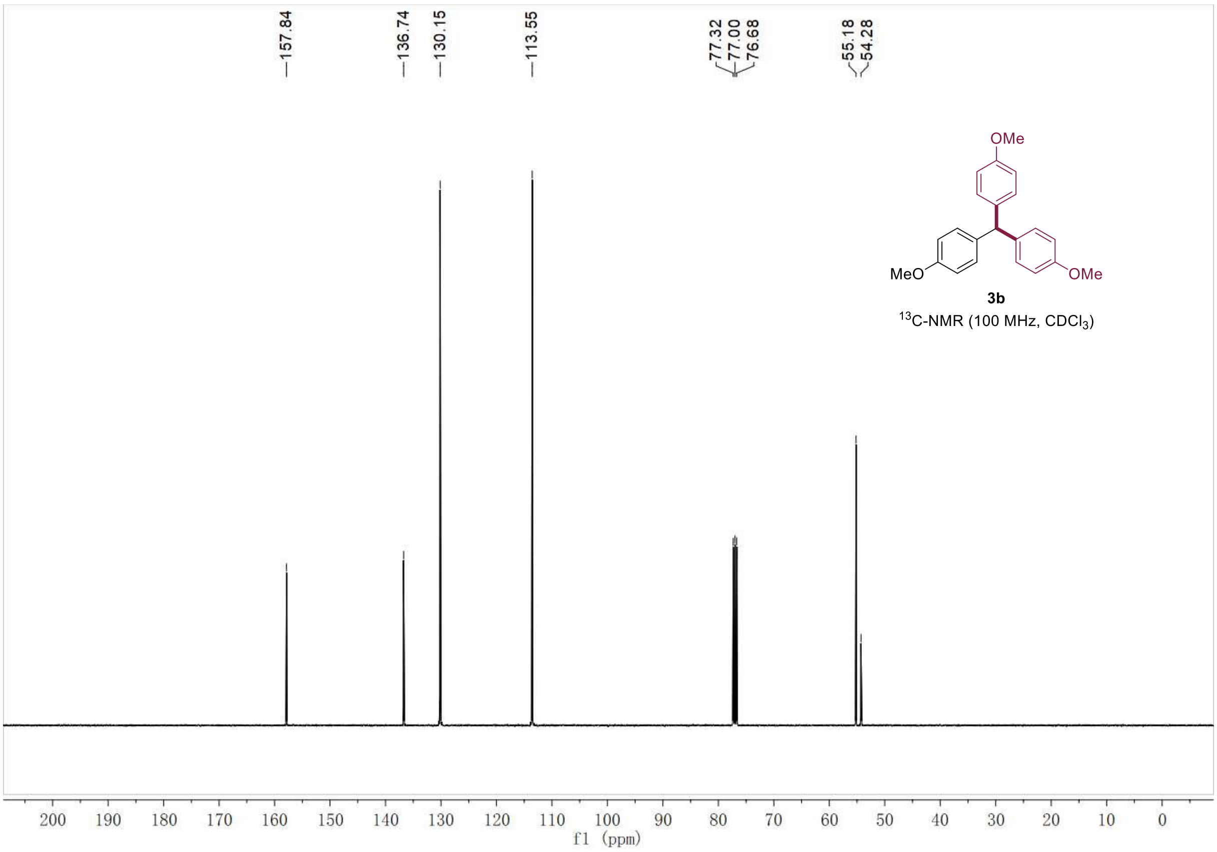
157.84
136.74
130.15
113.55
77.32
77.00
76.68
55.18
54.28
OMe
MeO
OMe
3b
13C-NMR (100 MHz, CDCl3)
200
190
180
170
160
150
140
130
120
110
100
90
80
70
60
50
40
30
20
10
0
f1 (ppm)

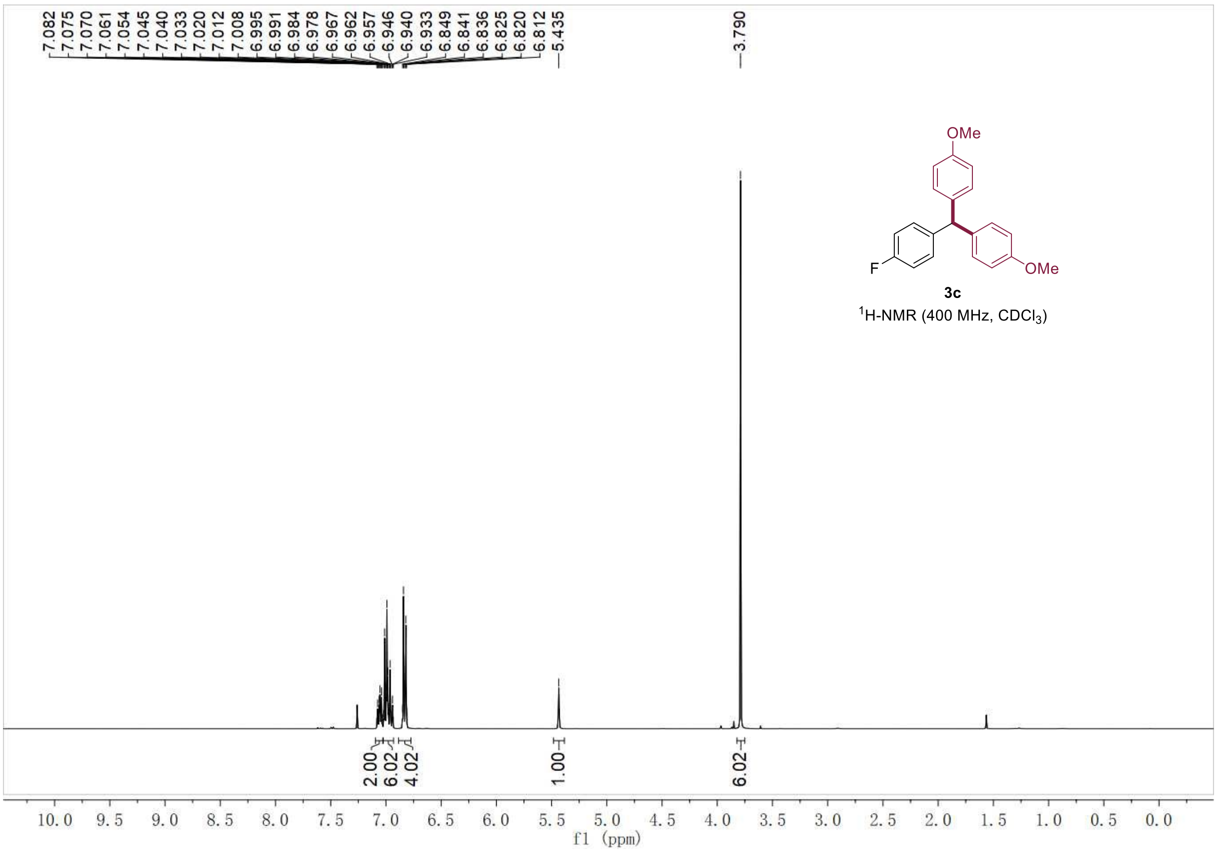
7.082
7.075
7.070
7.061
7.054
7.045
7.040
7.033
7.020
7.012
7.008
6.995
6.991
6.984
6.978
6.967
6.962
6.957
6.946
6.940
6.933
6.849
6.841
6.836
6.825
6.820
6.812
5.435
3.790
OMe
F
OMe
3c
1H-NMR (400 MHz, CDCl3)
2.00
6.02
4.02
1.00
6.02
10.0 9.5 9.0 8.5 8.0 7.5 7.0 6.5 6.0 5.5 5.0 4.5 4.0 3.5 3.0 2.5 2.0 1.5 1.0 0.5 0.0
f1 (ppm)

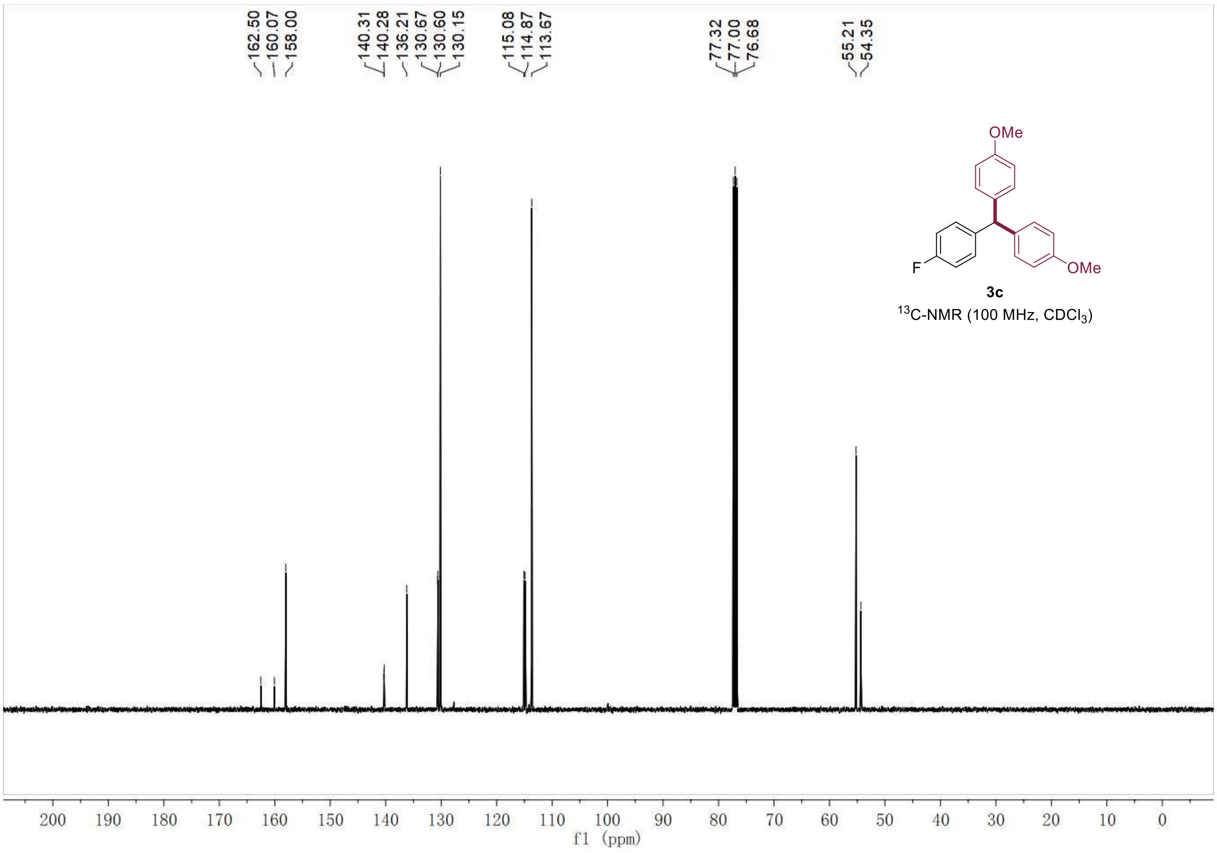
162.50
160.07
158.00
140.31
140.28
136.21
130.67
130.60
130.15
115.08
114.87
113.67
77.32
77.00
76.68
55.21
54.35
OMe
F
OMe
3c
13C-NMR (100 MHz, CDCl3)
200 190 180 170 160 150 140 130 120 110 100 90 80 70 60 50 40 30 20 10 0
f1 (ppm)

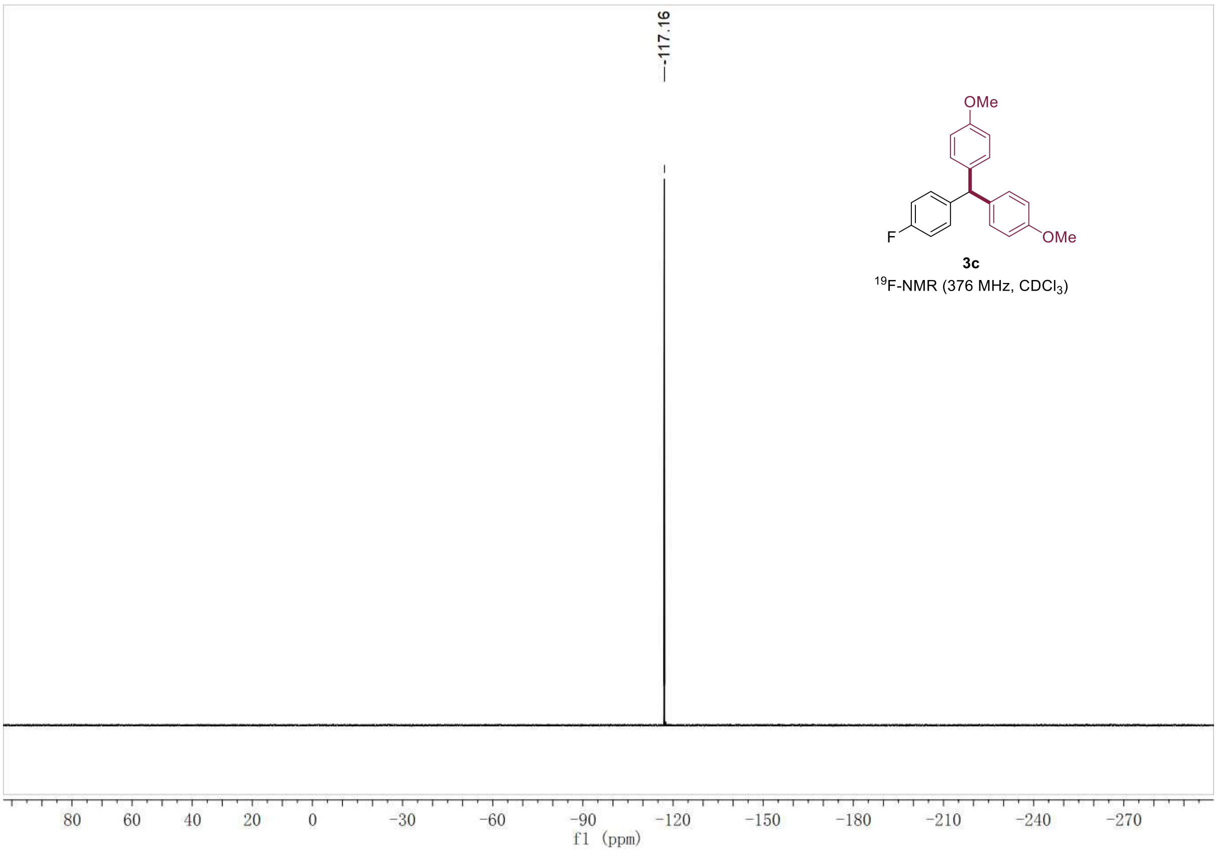
-117.16
OMe
F
OMe
3c
^{19}F-NMR (376 MHz, CDCl$_3$)
80 60 40 20 0 -30 -60 -90 -120 -150 -180 -210 -240 -270
f1 (ppm)

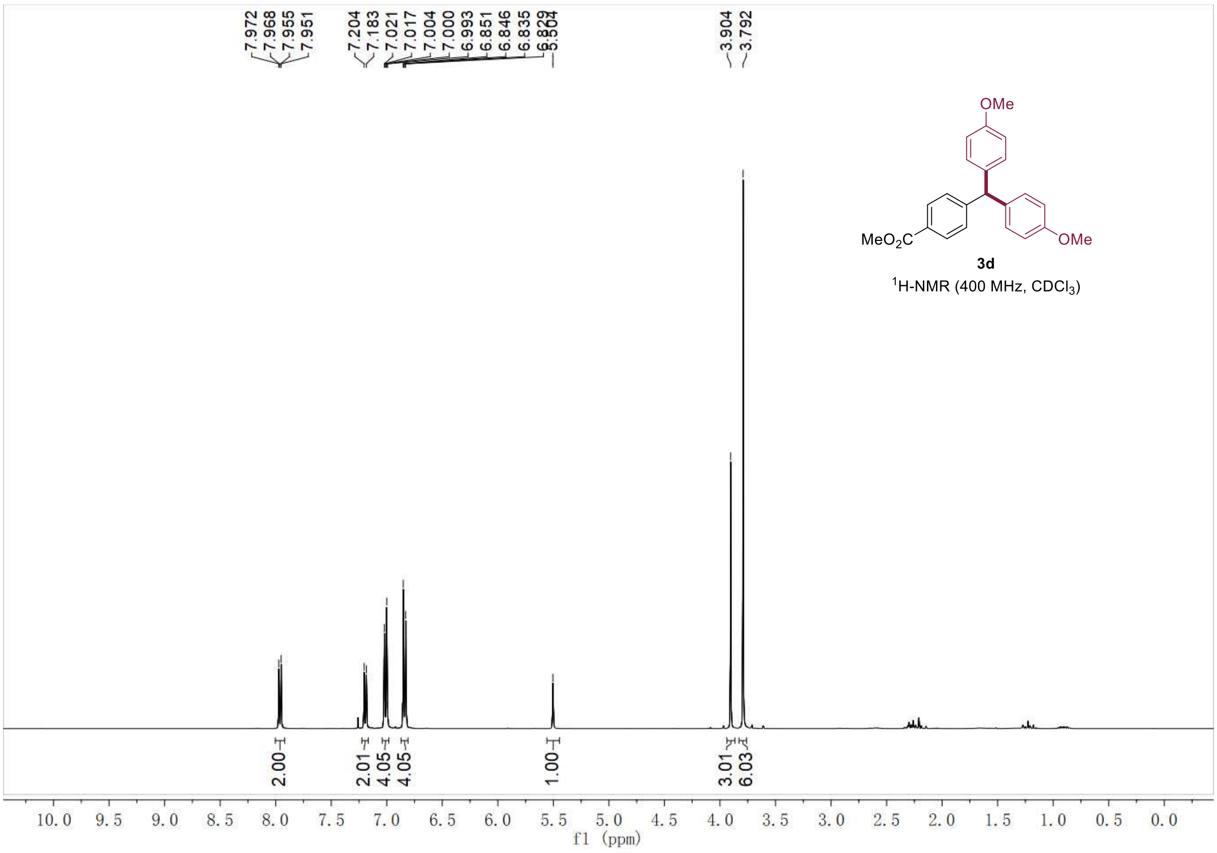
7.972
7.968
7.955
7.951
7.204
7.183
7.021
7.017
7.004
7.000
6.993
6.851
6.846
6.835
3.904
3.792
OMe
MeO$_2$C
OMe
3d
^{1}H-NMR (400 MHz, CDCl$_3$)
2.00
2.01
4.05
4.05
1.00
3.01
6.03
10.0 9.5 9.0 8.5 8.0 7.5 7.0 6.5 6.0 5.5 5.0 4.5 4.0 3.5 3.0 2.5 2.0 1.5 1.0 0.5 0.0
f1 (ppm)

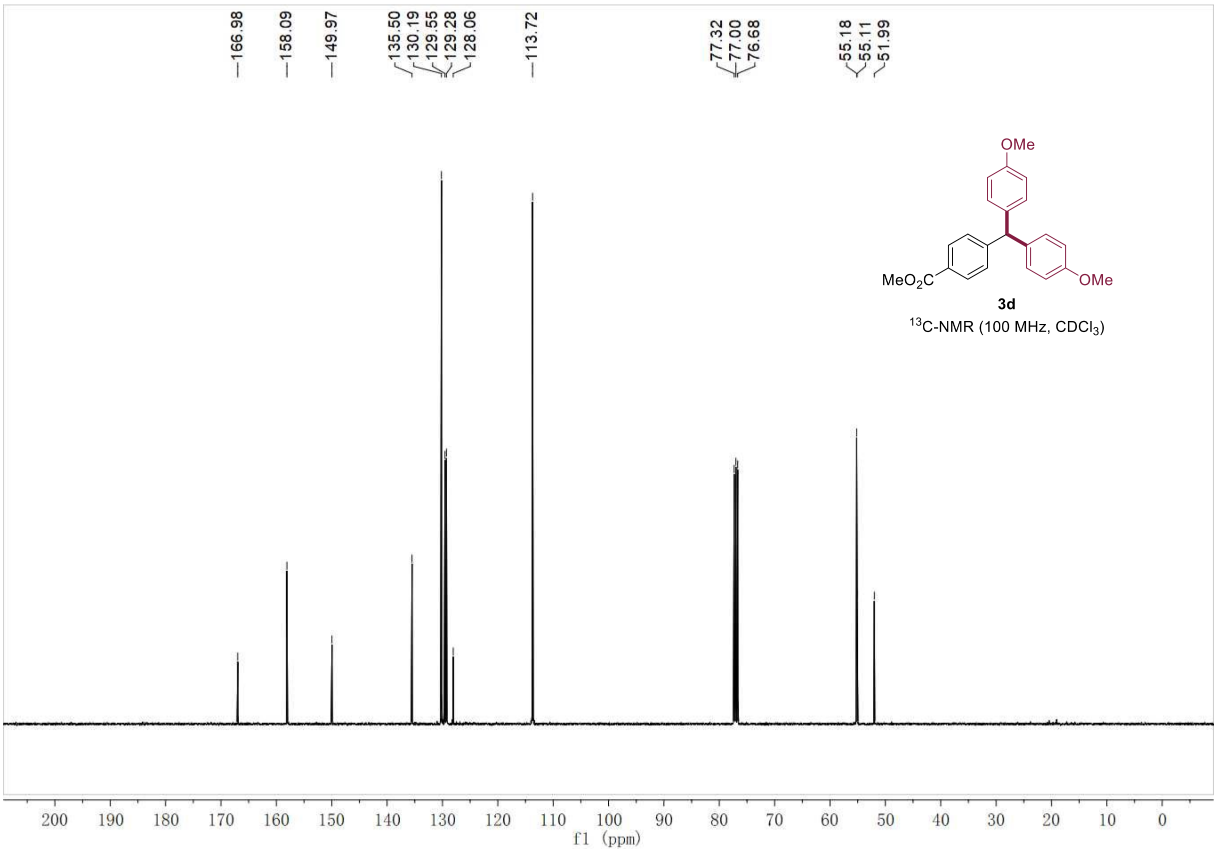

166.98
158.09
149.97
135.50
130.19
129.55
129.28
128.06
113.72
77.32
77.00
76.68
55.18
55.11
51.99
OMe
MeO2C
OMe
3d
13C-NMR (100 MHz, CDCl3)
f1 (ppm)

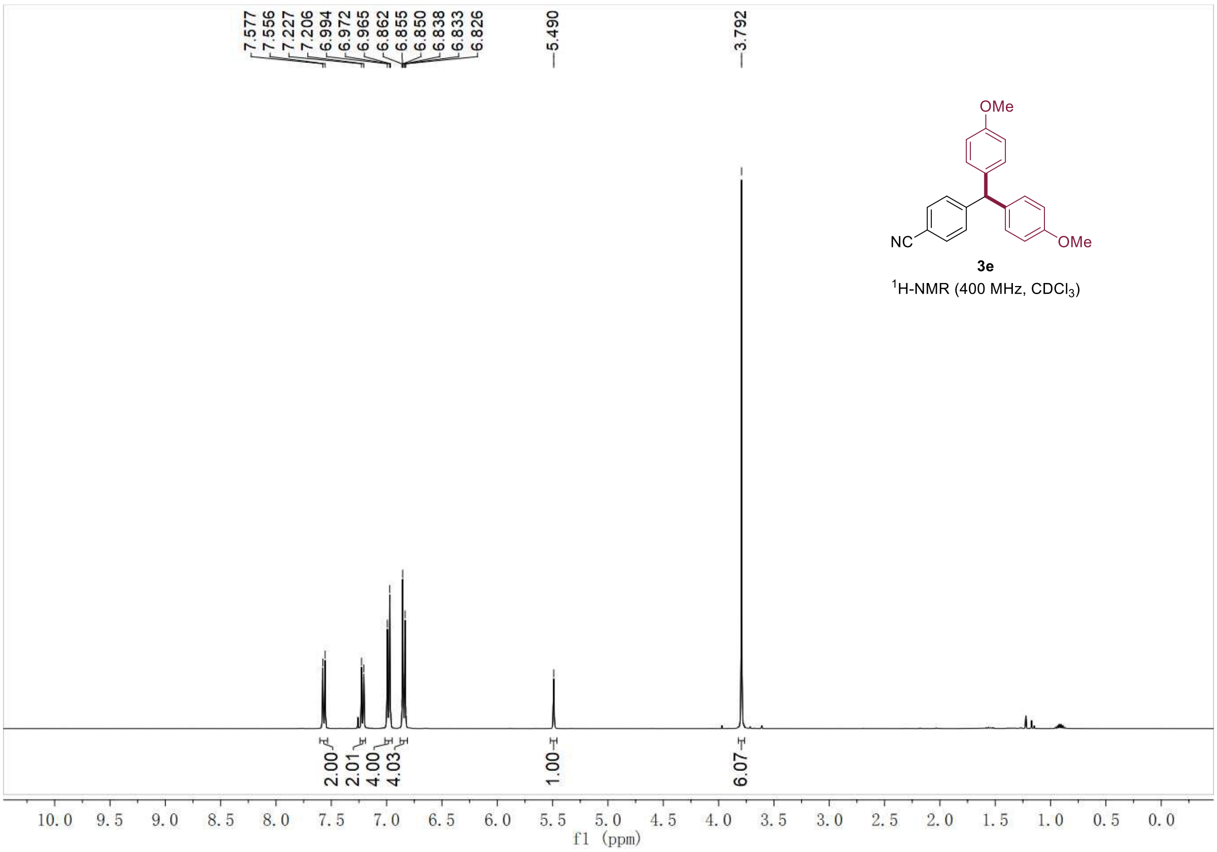

7.577
7.556
7.227
7.206
6.994
6.972
6.965
6.862
6.855
6.850
6.838
6.833
6.826
5.490
3.792
OMe
NC
OMe
3e
1H-NMR (400 MHz, CDCl3)
2.00
2.01
4.00
4.03
1.00
6.07
f1 (ppm)

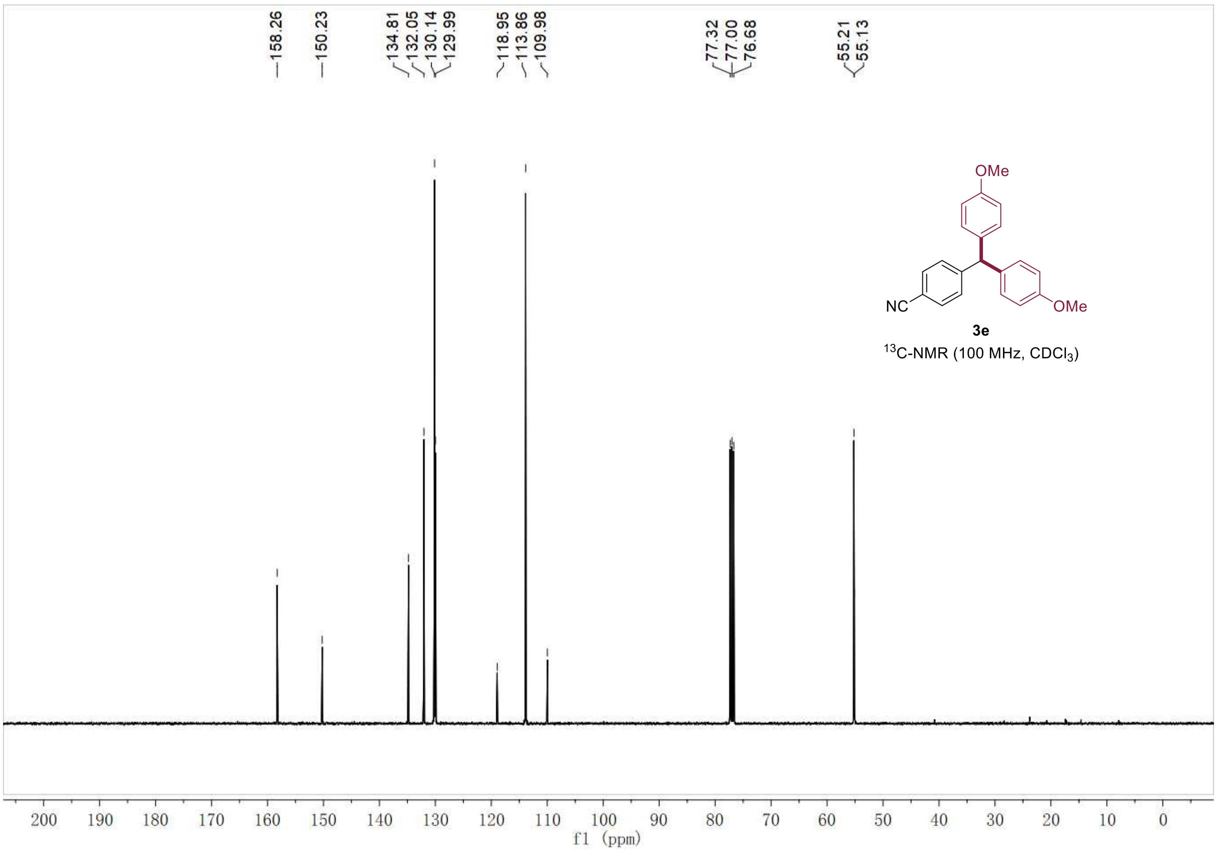
158.26
150.23
134.81
132.05
130.14
129.99
118.95
113.86
109.98
77.32
77.00
76.68
55.21
55.13
OMe
NC
OMe
3e
^{13}C-NMR (100 MHz, CDCl$_3$)
200 190 180 170 160 150 140 130 120 110 100 90 80 70 60 50 40 30 20 10 0
f1 (ppm)

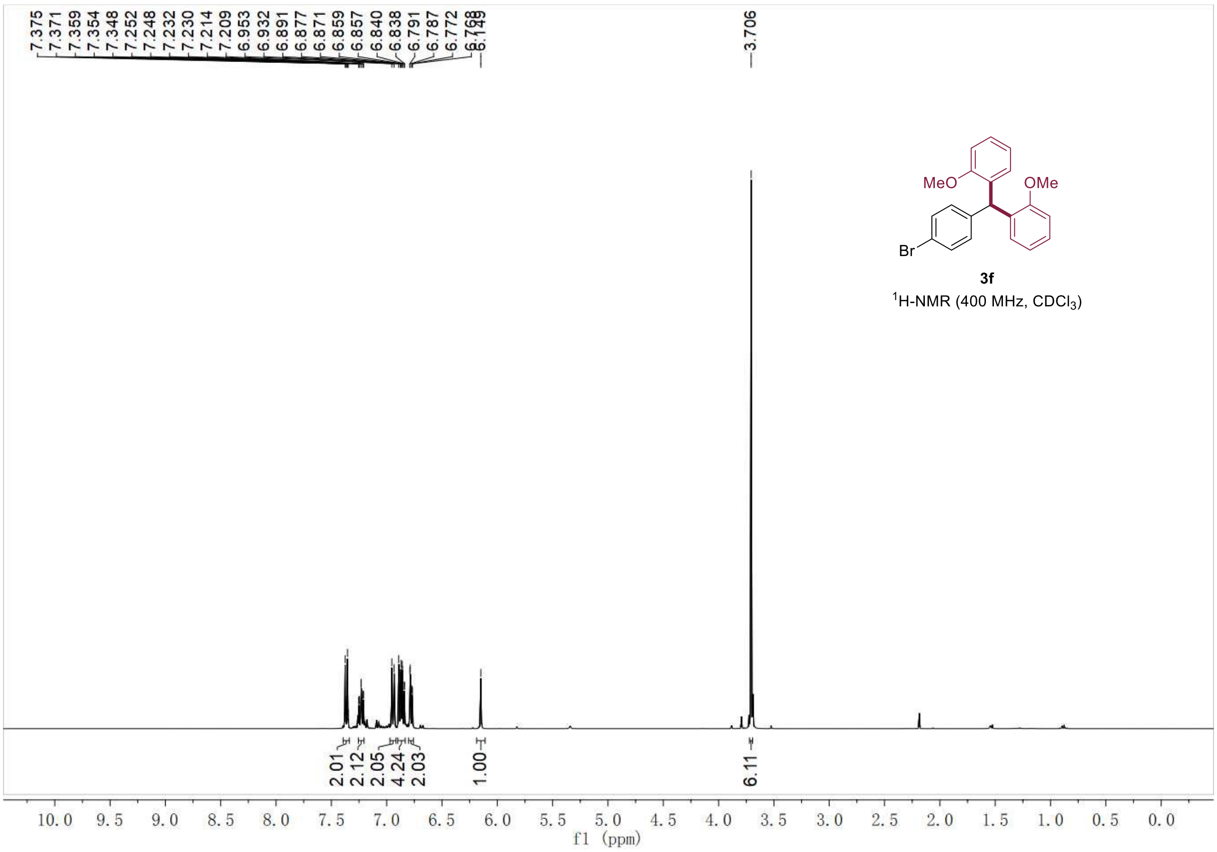
7.375
7.371
7.359
7.354
7.348
7.252
7.248
7.232
7.230
7.214
7.209
6.953
6.932
6.891
6.877
6.871
6.859
6.857
6.840
6.838
6.791
6.787
6.772
3.706
MeO
OMe
Br
3f
^{1}H-NMR (400 MHz, CDCl$_3$)
2.01
2.12
2.05
4.24
2.03
1.00
6.11
10.0 9.5 9.0 8.5 8.0 7.5 7.0 6.5 6.0 5.5 5.0 4.5 4.0 3.5 3.0 2.5 2.0 1.5 1.0 0.5 0.0
f1 (ppm)

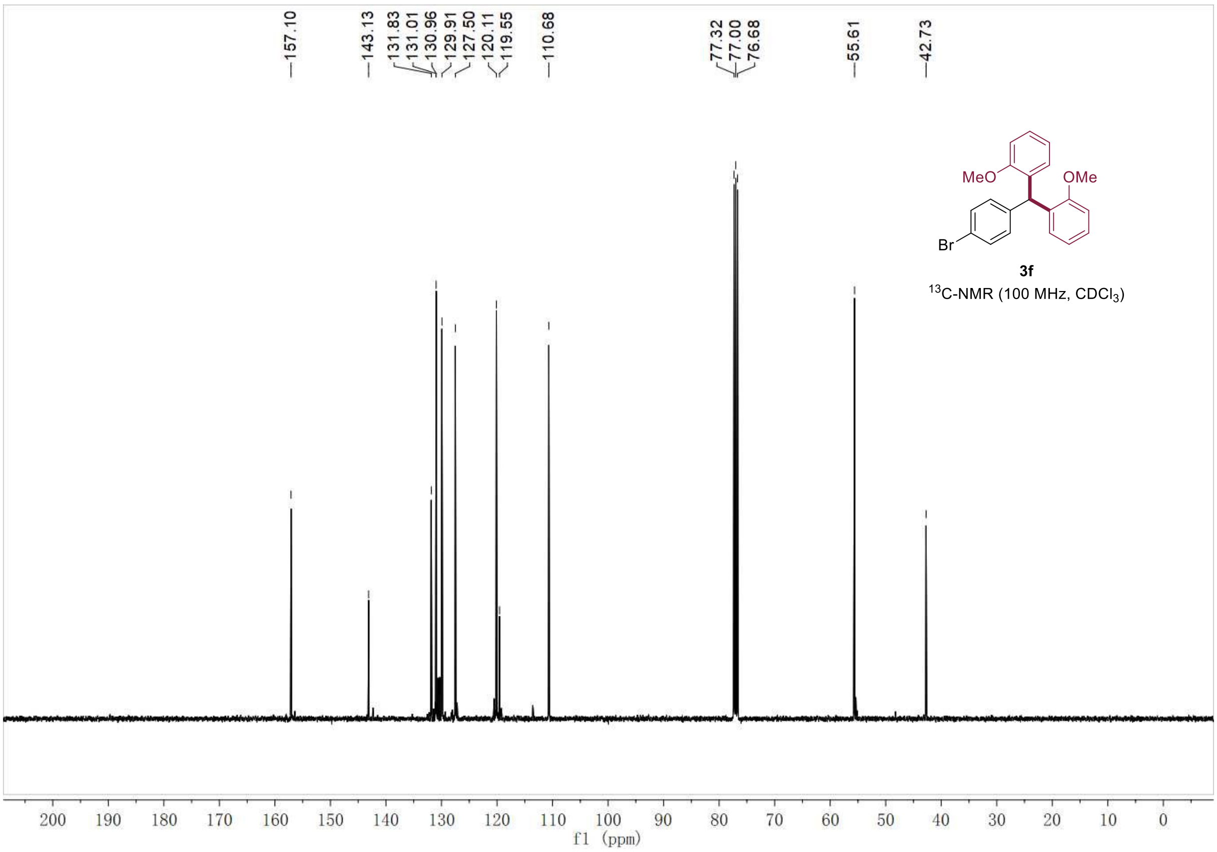
157.10
143.13
131.83
131.01
130.96
129.91
127.50
120.11
119.55
110.68
77.32
77.00
76.68
55.61
42.73
MeO
OMe
Br
3f
13C-NMR (100 MHz, CDCl3)
f1 (ppm)

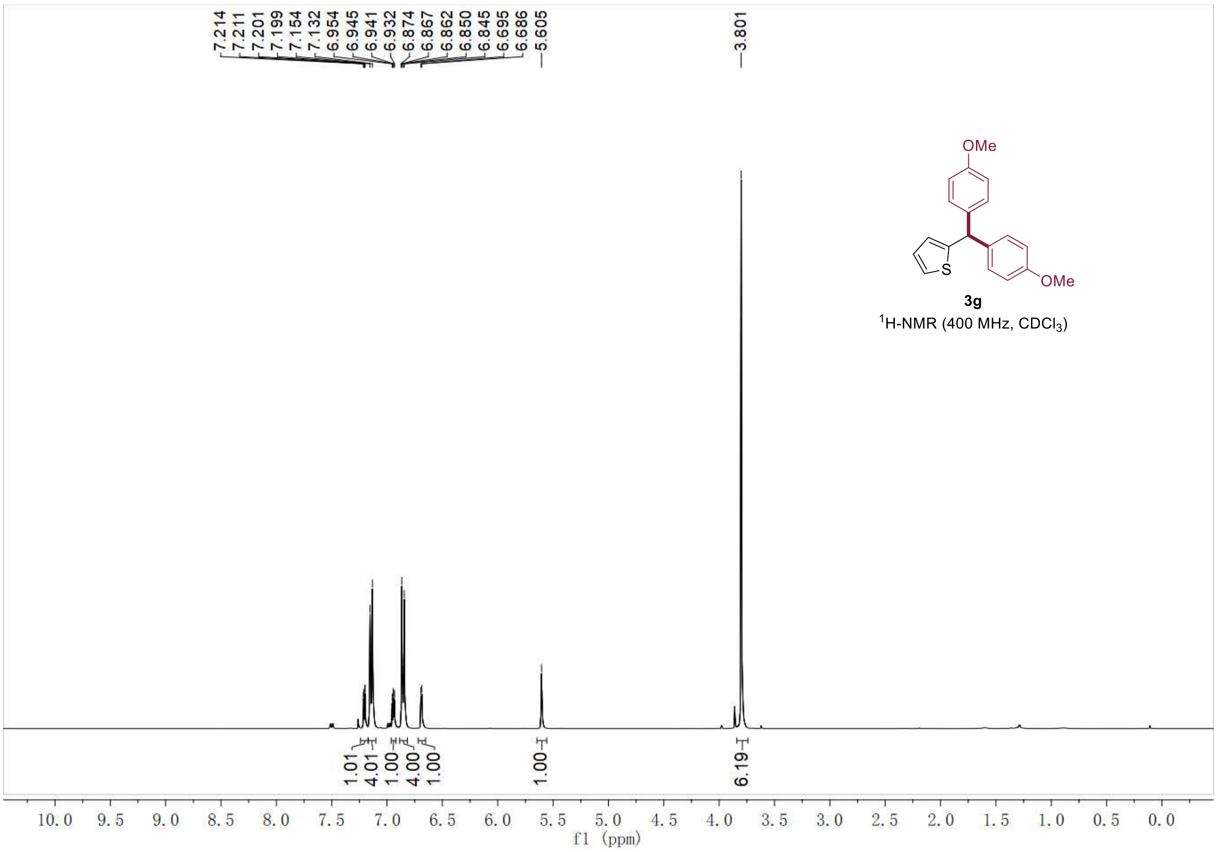
7.214
7.211
7.201
7.199
7.154
7.132
6.954
6.945
6.941
6.932
6.874
6.867
6.862
6.850
6.845
6.695
6.686
5.605
3.801
OMe
S
OMe
3g
1H-NMR (400 MHz, CDCl3)
1.01
4.01
1.00
4.00
1.00
1.00
6.19
f1 (ppm)

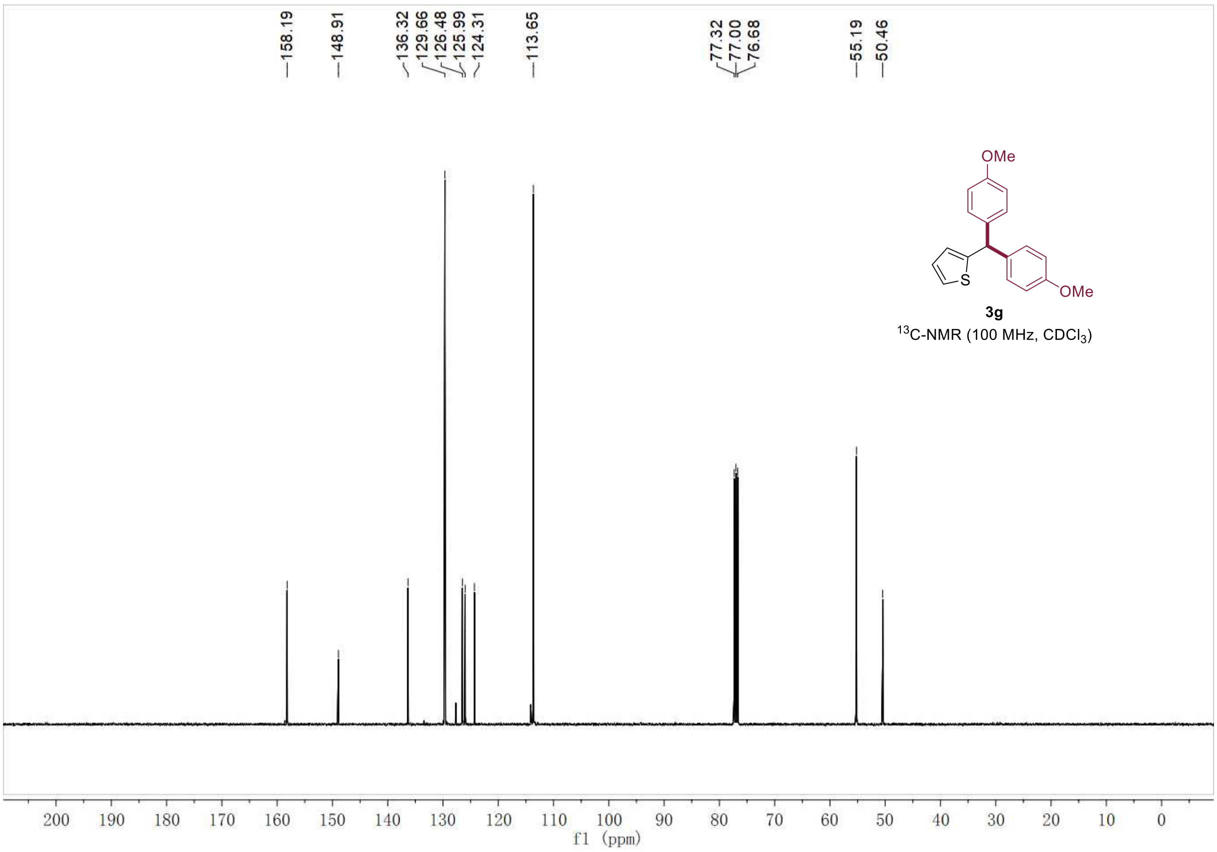
158.19
148.91
136.32
129.66
126.48
125.99
124.31
113.65
77.32
77.00
76.68
55.19
50.46
OMe
S
OMe
3g
13C-NMR (100 MHz, CDCl3)
200 190 180 170 160 150 140 130 120 110 100 90 80 70 60 50 40 30 20 10 0
f1 (ppm)

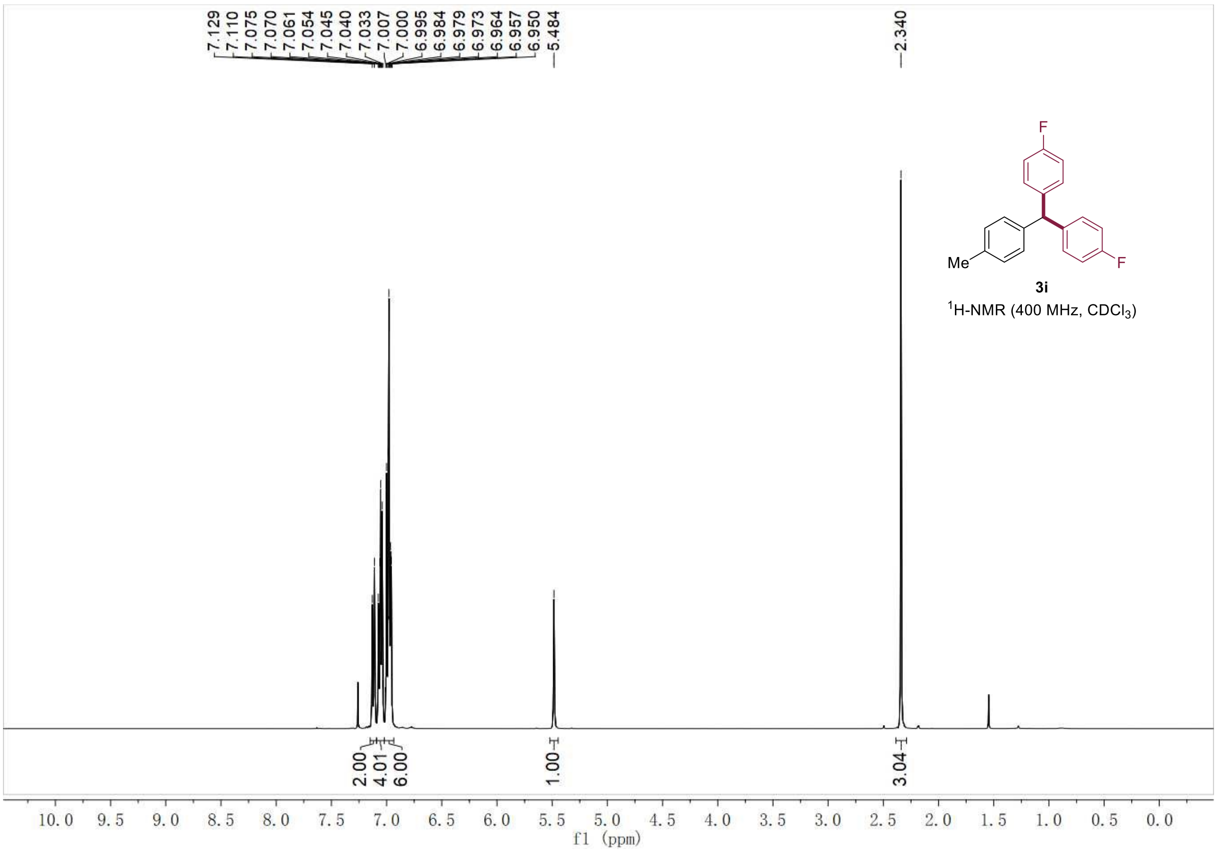
7.129
7.110
7.075
7.070
7.061
7.054
7.045
7.040
7.033
7.007
7.000
6.995
6.984
6.979
6.973
6.964
6.957
6.950
5.484
2.340
F
Me
F
3i
1H-NMR (400 MHz, CDCl3)
2.00
4.01
6.00
1.00
3.04
10.0 9.5 9.0 8.5 8.0 7.5 7.0 6.5 6.0 5.5 5.0 4.5 4.0 3.5 3.0 2.5 2.0 1.5 1.0 0.5 0.0
f1 (ppm)

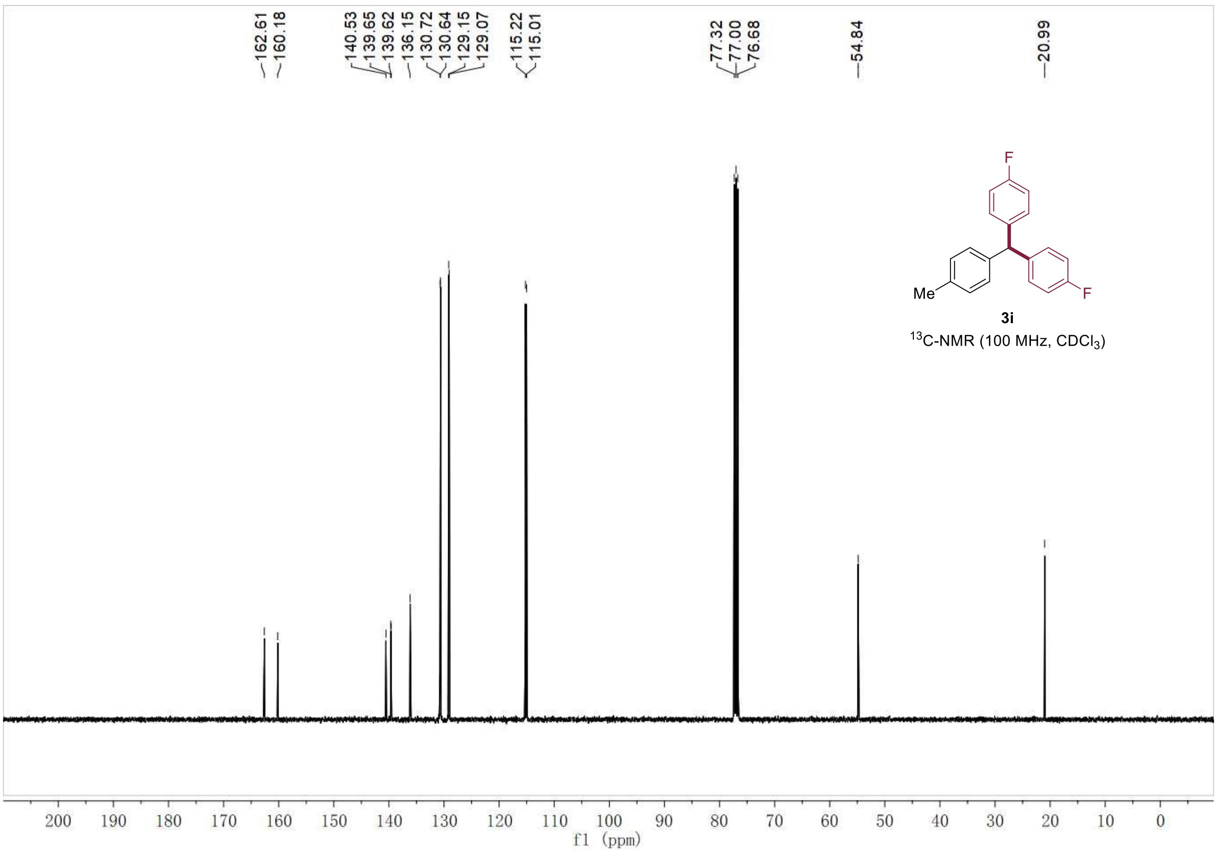
162.61
160.18
140.53
139.65
139.62
136.15
130.72
130.64
129.15
129.07
115.22
115.01
77.32
77.00
76.68
54.84
20.99
F
Me
F
3i
13C-NMR (100 MHz, CDCl3)
f1 (ppm)

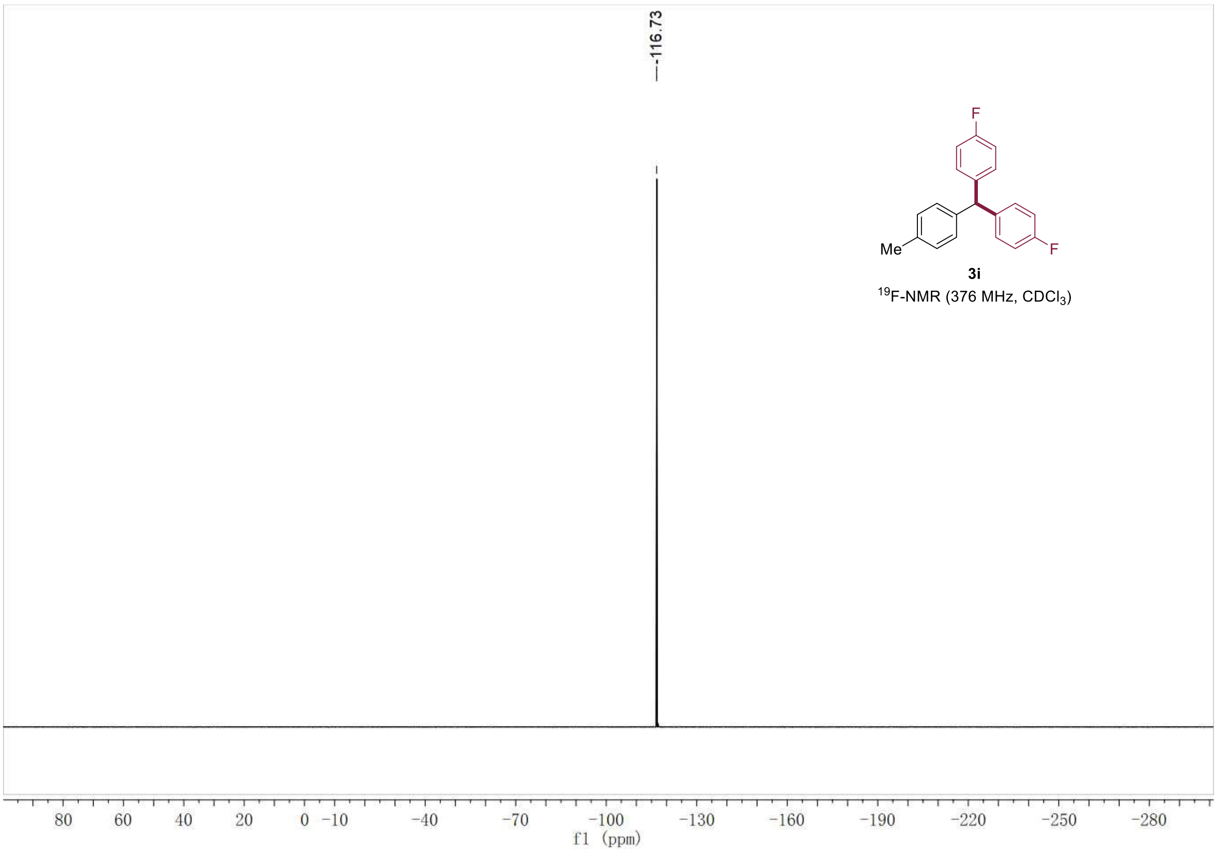
-116.73
F
Me
F
3i
19F-NMR (376 MHz, CDCl3)
f1 (ppm)

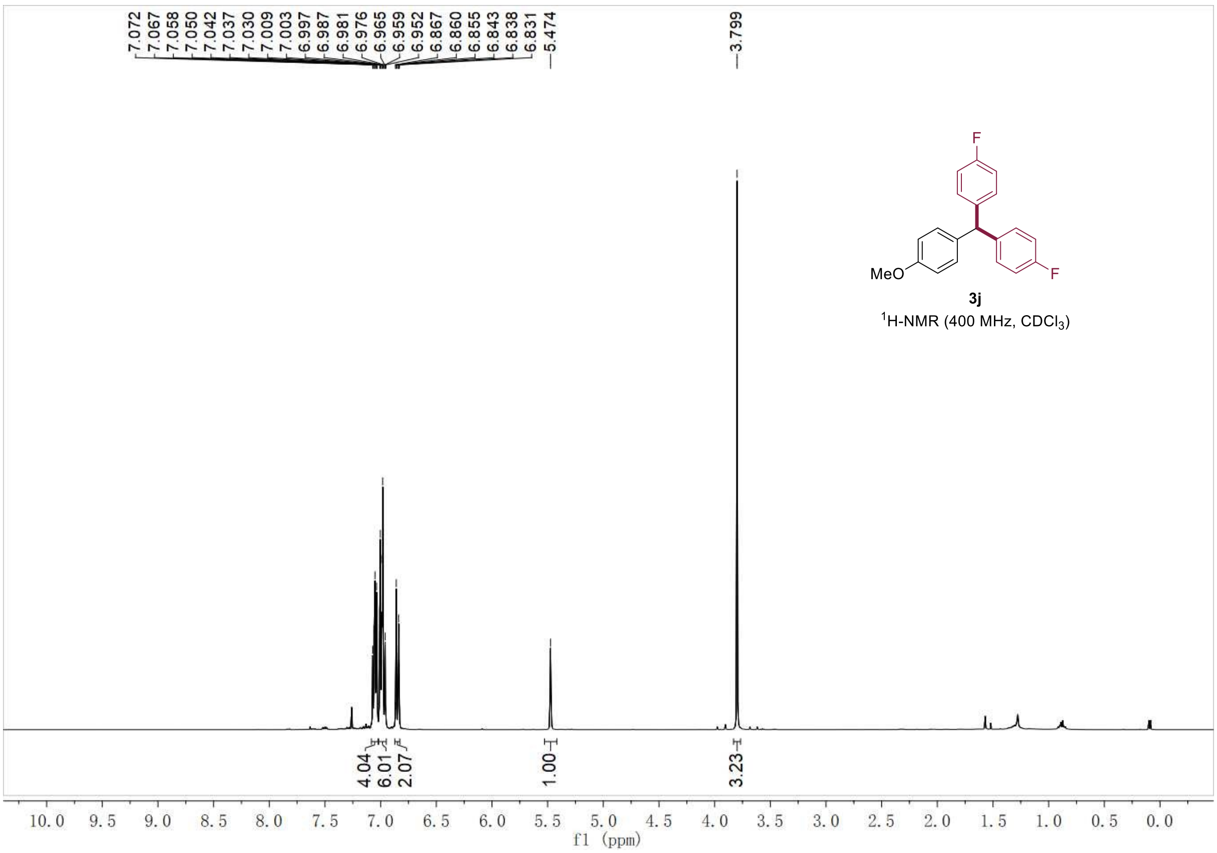
7.072
7.067
7.058
7.050
7.042
7.037
7.030
7.009
7.003
6.997
6.987
6.981
6.976
6.965
6.959
6.952
6.867
6.860
6.855
6.843
6.838
6.831
5.474
3.799
F
MeO
F
3j
1H-NMR (400 MHz, CDCl3)
4.04
6.01
2.07
1.00
3.23
10.0 9.5 9.0 8.5 8.0 7.5 7.0 6.5 6.0 5.5 5.0 4.5 4.0 3.5 3.0 2.5 2.0 1.5 1.0 0.5 0.0
f1 (ppm)

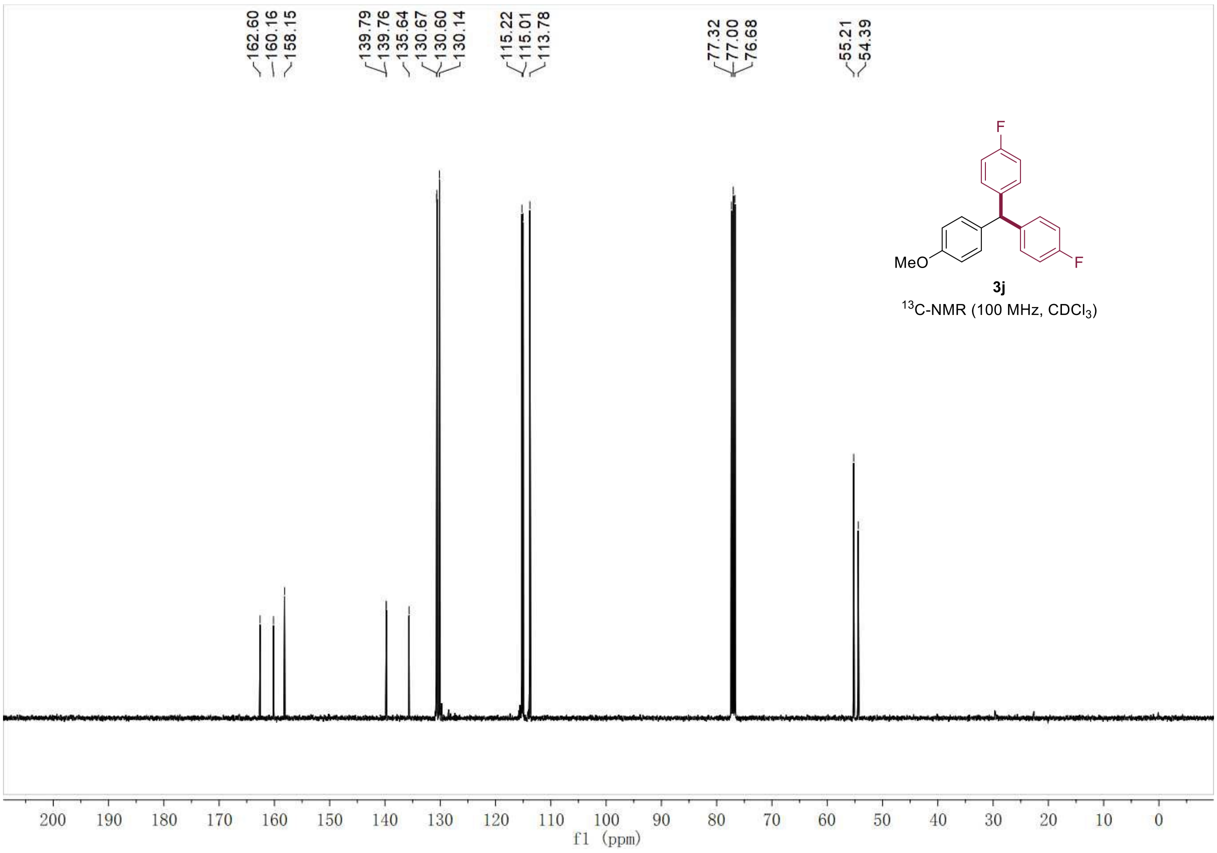
162.60
160.16
158.15
139.79
139.76
135.64
130.67
130.60
130.14
115.22
115.01
113.78
77.32
77.00
76.68
55.21
54.39
F
MeO
F
3j
13C-NMR (100 MHz, CDCl3)
200 190 180 170 160 150 140 130 120 110 100 90 80 70 60 50 40 30 20 10 0
f1 (ppm)

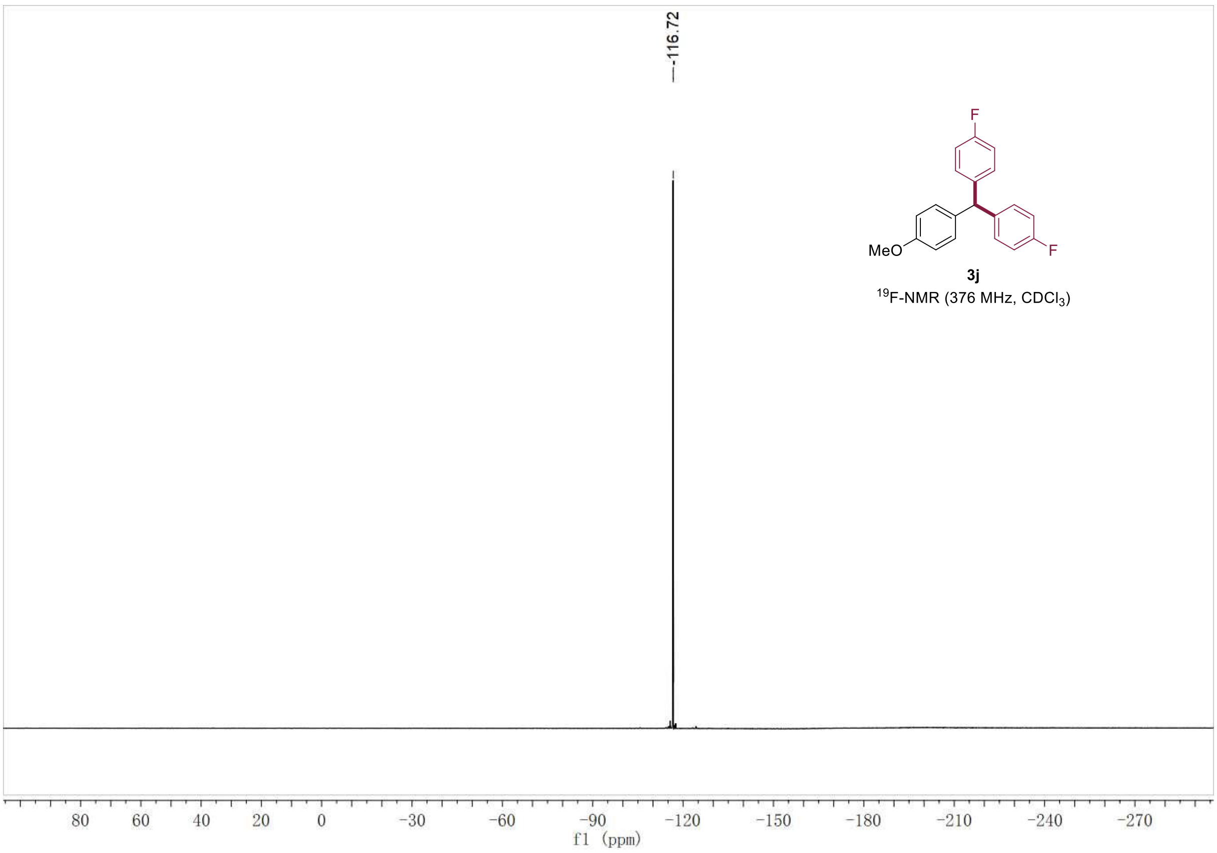
-116.72
MeO
F
F
3j
19F-NMR (376 MHz, CDCl3)
80 60 40 20 0 -30 -60 -90 -120 -150 -180 -210 -240 -270
f1 (ppm)

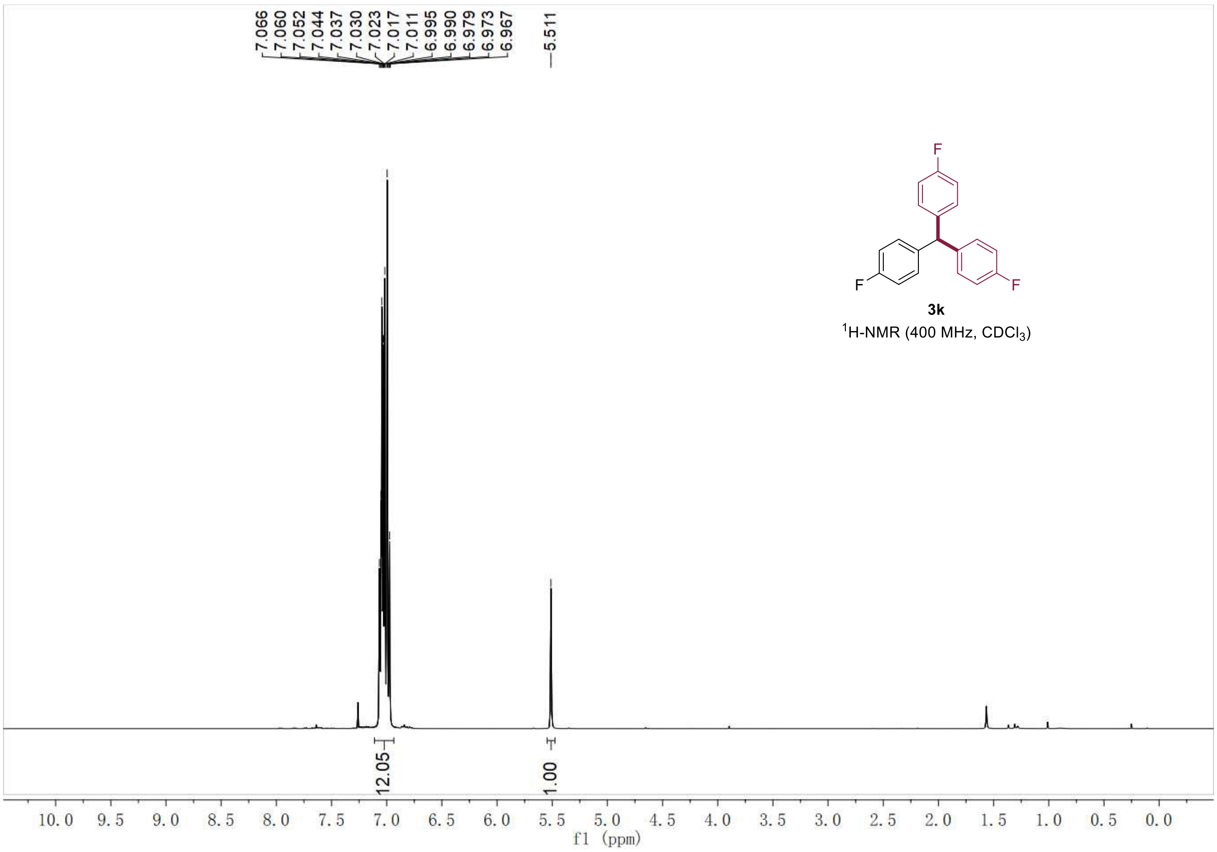
7.066
7.060
7.052
7.044
7.037
7.030
7.023
7.017
7.011
6.995
6.990
6.979
6.973
6.967
5.511
F
F
F
3k
1H-NMR (400 MHz, CDCl3)
12.05
1.00
10.0 9.5 9.0 8.5 8.0 7.5 7.0 6.5 6.0 5.5 5.0 4.5 4.0 3.5 3.0 2.5 2.0 1.5 1.0 0.5 0.0
f1 (ppm)

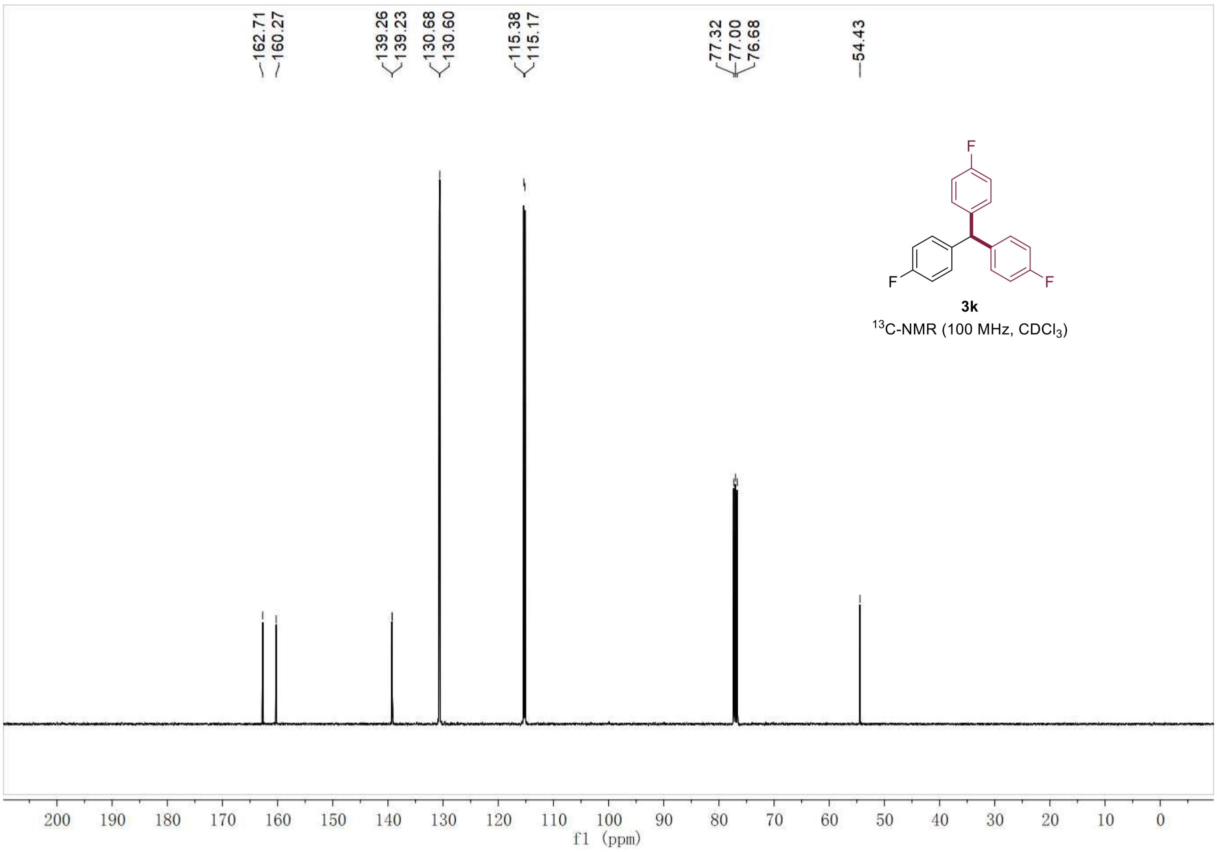
162.71
160.27
139.26
139.23
130.68
130.60
115.38
115.17
77.32
77.00
76.68
54.43
F
F
F
3k
13C-NMR (100 MHz, CDCl3)
200 190 180 170 160 150 140 130 120 110 100 90 80 70 60 50 40 30 20 10 0
f1 (ppm)

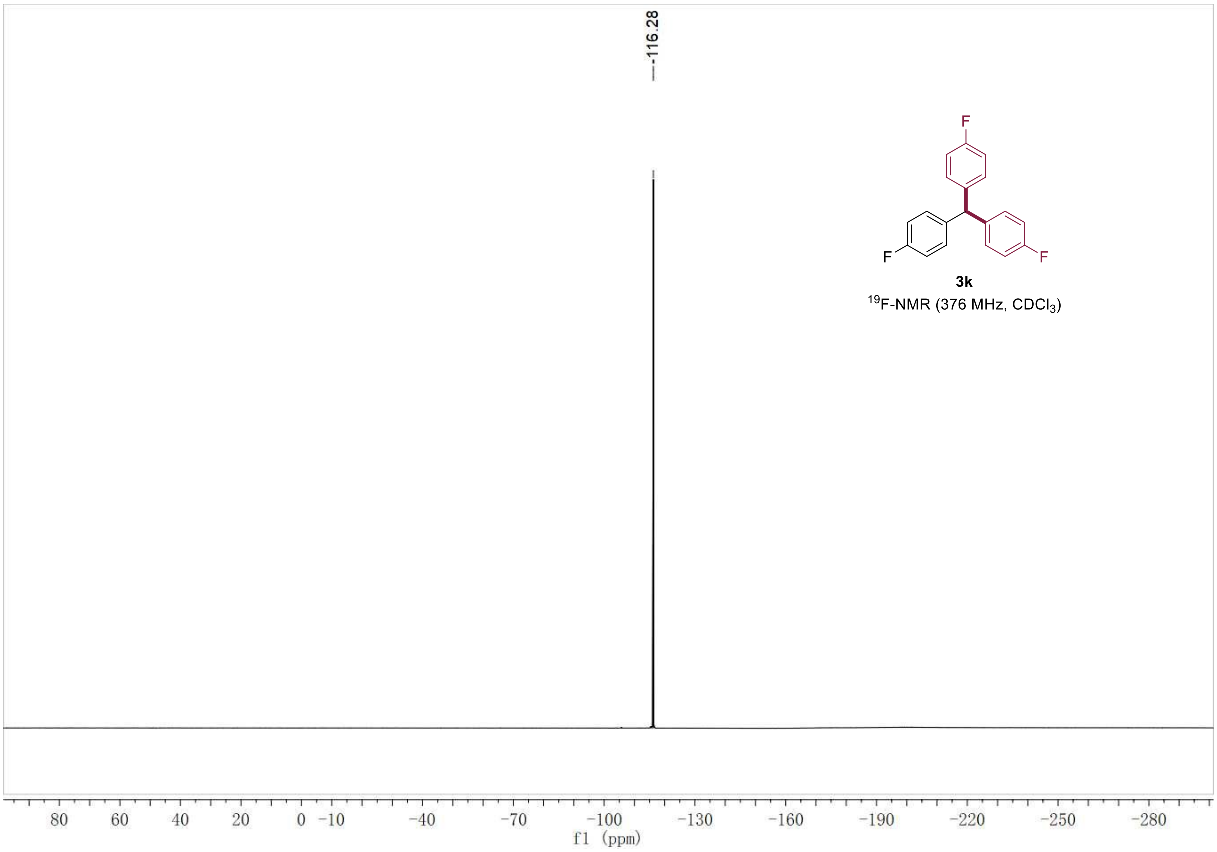
-116.28
F
F
F
3k
19F-NMR (376 MHz, CDCl3)
80 60 40 20 0 -10 -40 -70 -100 -130 -160 -190 -220 -250 -280
f1 (ppm)

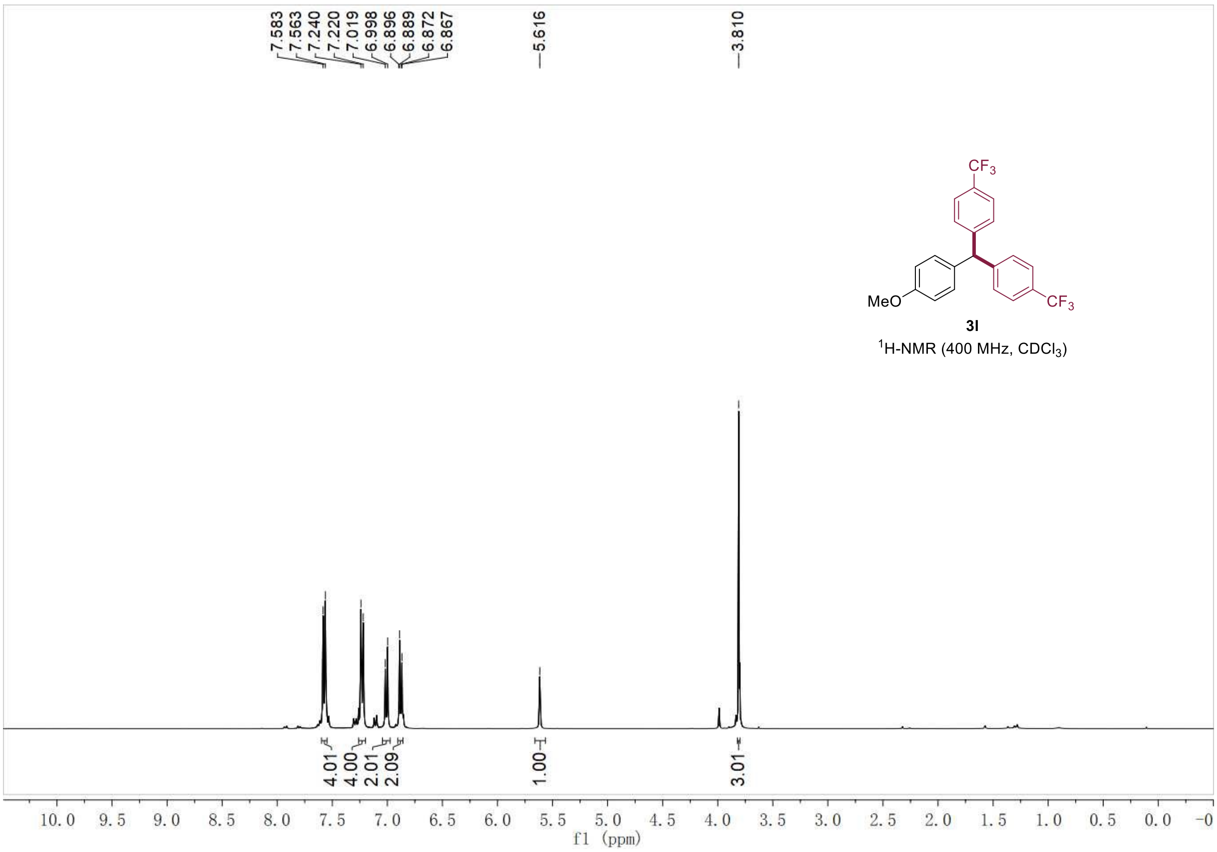

7.583
7.563
7.240
7.220
7.019
6.998
6.896
6.889
6.872
6.867
5.616
3.810
CF3
MeO
CF3
3l
1H-NMR (400 MHz, CDCl3)
4.01
4.00
2.01
2.09
1.00
3.01
f1 (ppm)

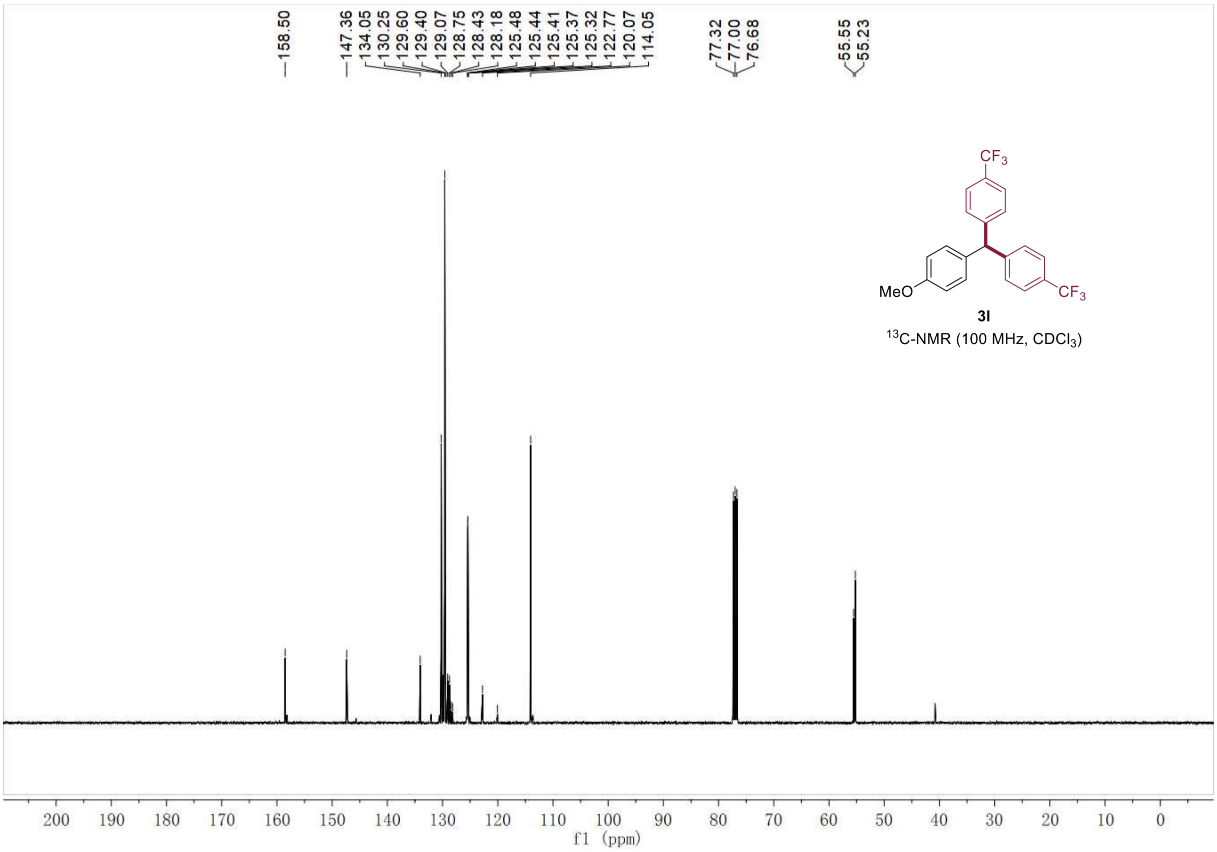

158.50
147.36
134.05
130.25
129.60
129.40
129.07
128.75
128.43
128.18
125.48
125.44
125.41
125.37
125.32
122.77
120.07
114.05
77.32
77.00
76.68
55.55
55.23
CF3
MeO
CF3
3l
13C-NMR (100 MHz, CDCl3)
f1 (ppm)

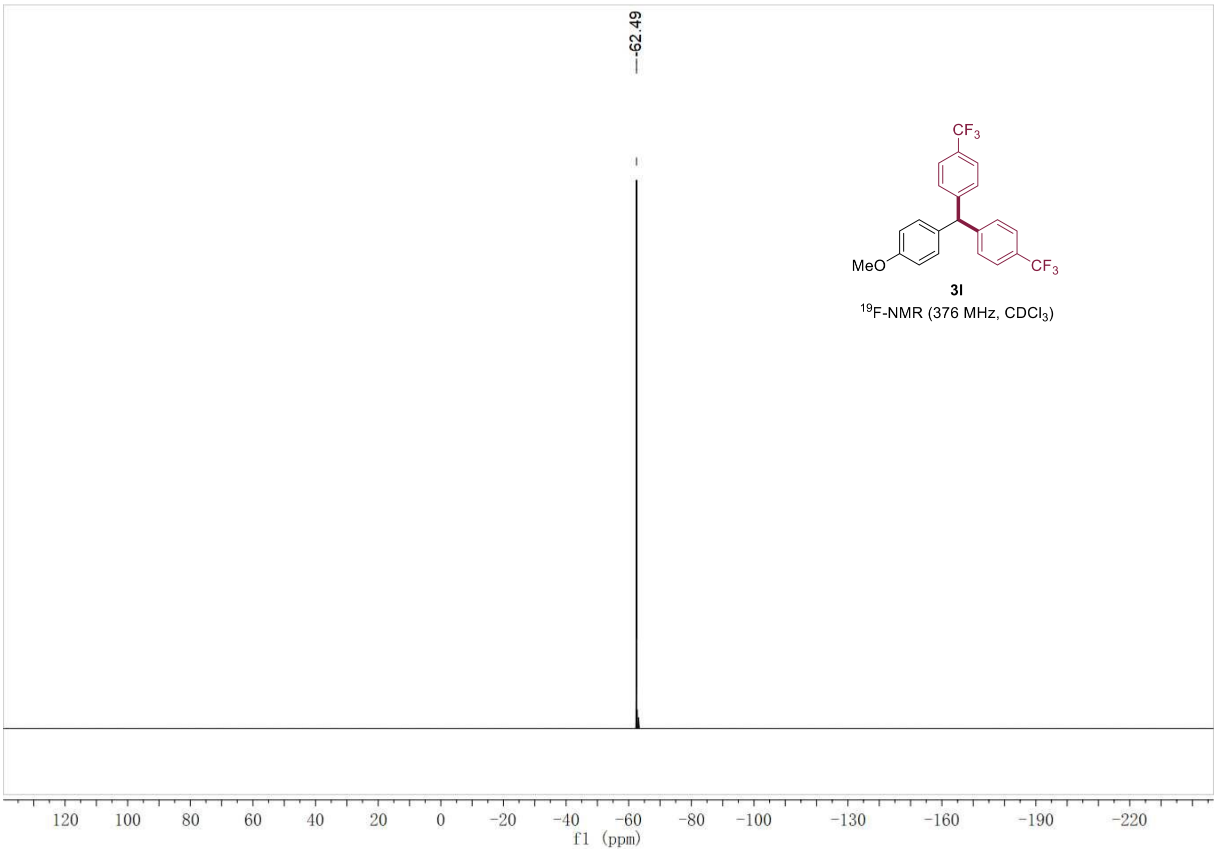
-62.49
CF3
MeO
CF3
3l
19F-NMR (376 MHz, CDCl3)
f1 (ppm)

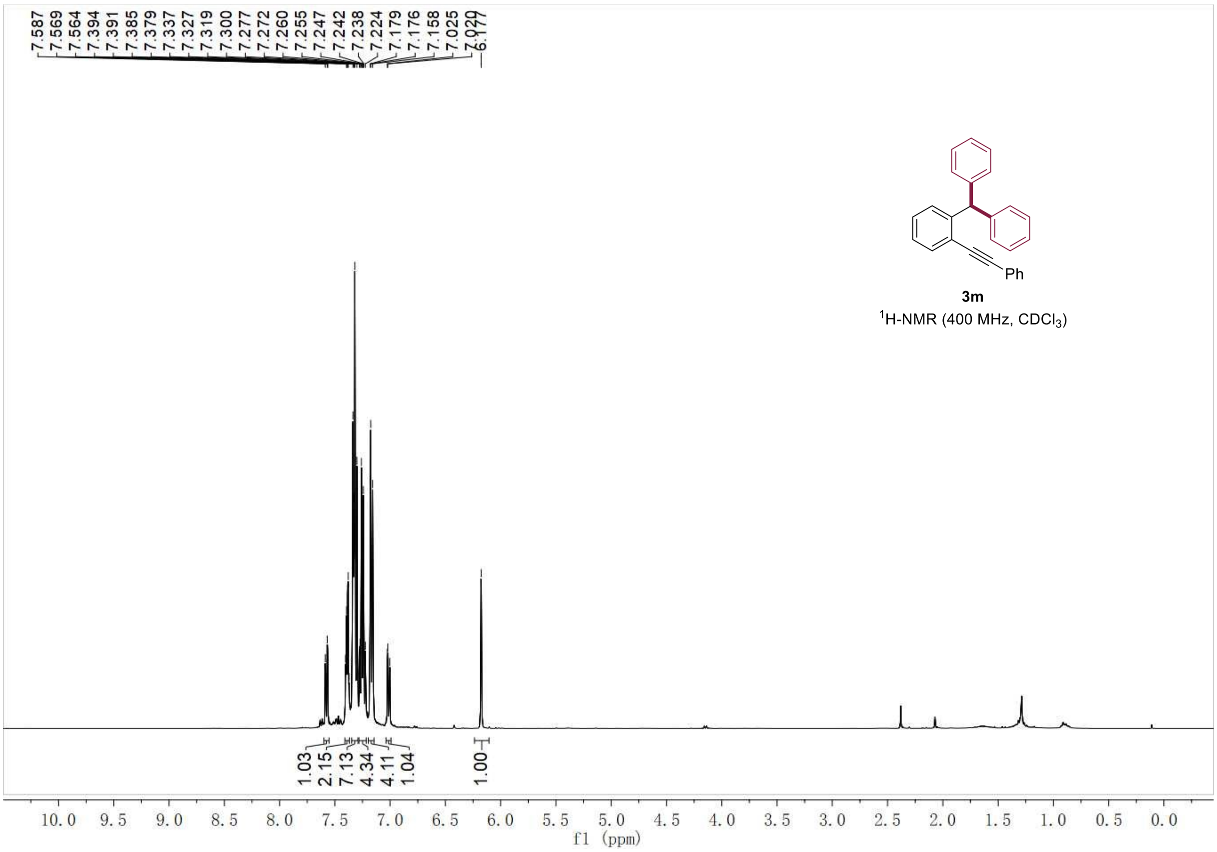
7.587
7.569
7.564
7.394
7.391
7.385
7.379
7.337
7.327
7.319
7.300
7.277
7.272
7.260
7.255
7.247
7.242
7.238
7.224
7.179
7.176
7.158
7.025
7.020
6.177
Ph
3m
1H-NMR (400 MHz, CDCl3)
1.03
2.15
7.13
4.34
4.11
1.04
1.00
f1 (ppm)

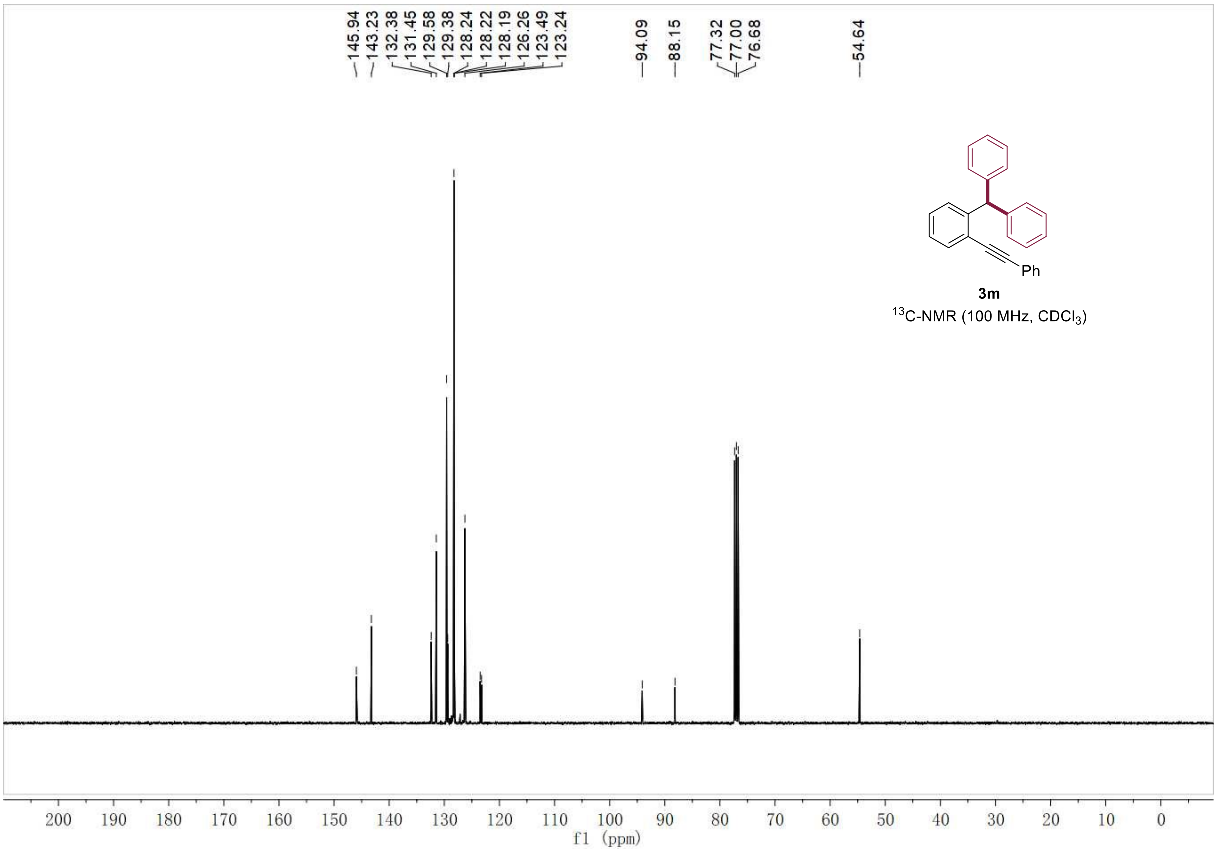
145.94
143.23
132.38
131.45
129.58
129.38
128.24
128.22
128.19
126.26
123.49
123.24
94.09
88.15
77.32
77.00
76.68
54.64
Ph
3m
^{13}C-NMR (100 MHz, CDCl$_3$)
200 190 180 170 160 150 140 130 120 110 100 90 80 70 60 50 40 30 20 10 0
f1 (ppm)

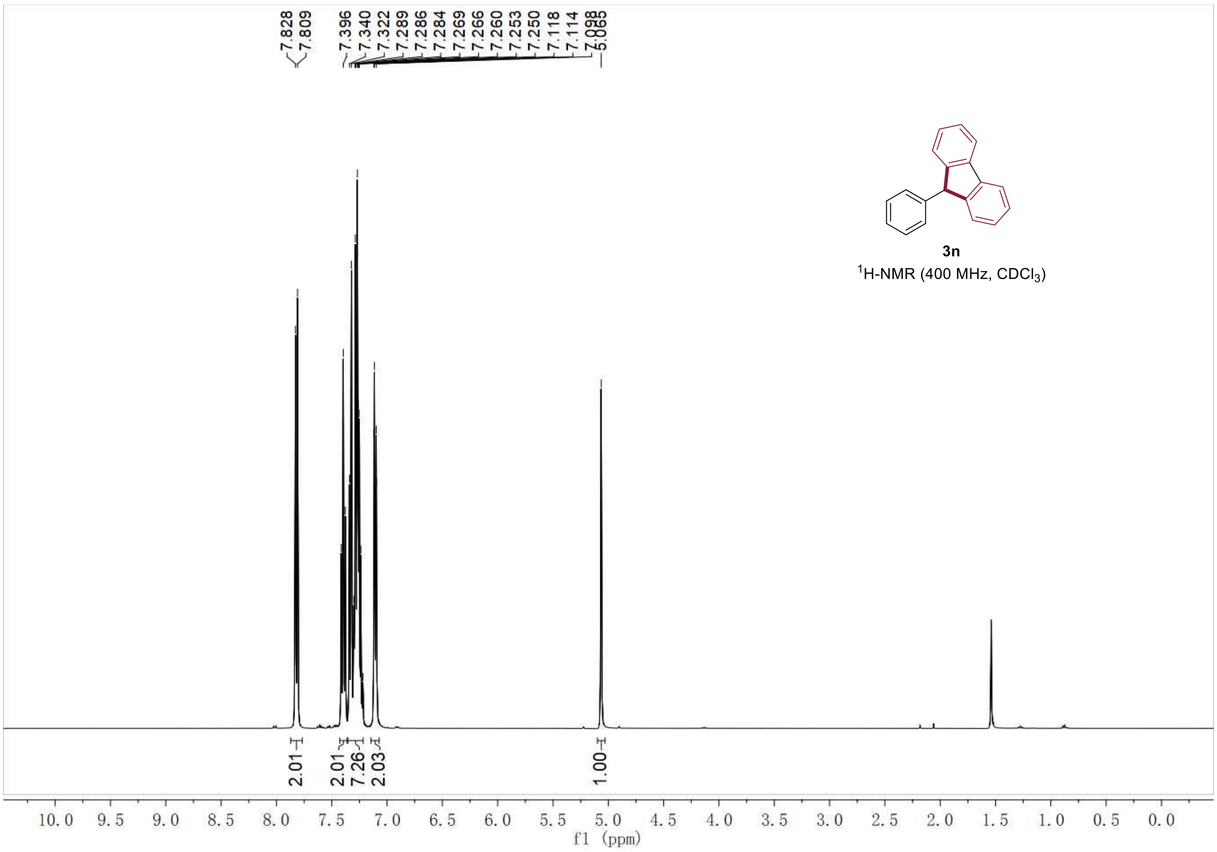
7.828
7.809
7.396
7.340
7.322
7.289
7.286
7.284
7.269
7.266
7.260
7.253
7.250
7.118
7.114
7.098
5.065
3n
^{1}H-NMR (400 MHz, CDCl$_3$)
2.01
2.01
7.26
2.03
1.00
10.0 9.5 9.0 8.5 8.0 7.5 7.0 6.5 6.0 5.5 5.0 4.5 4.0 3.5 3.0 2.5 2.0 1.5 1.0 0.5 0.0
f1 (ppm)

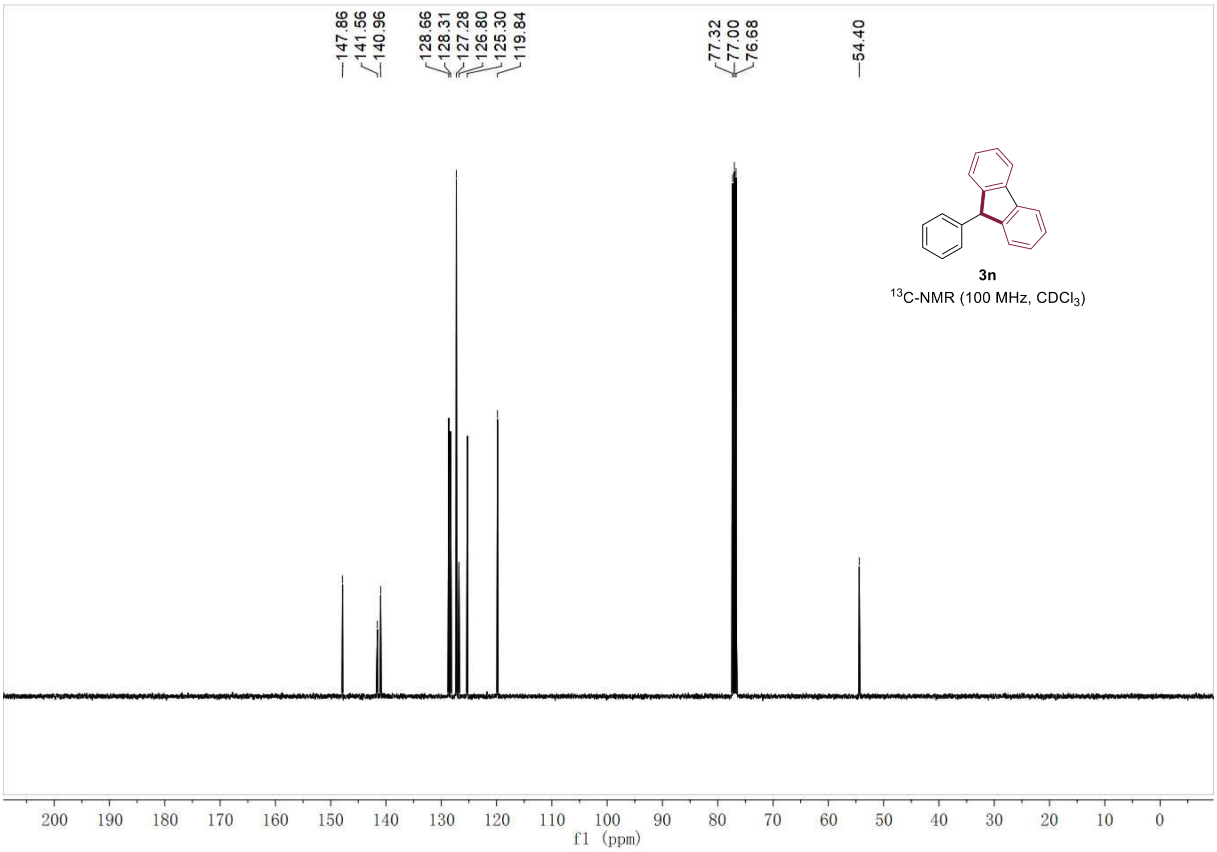
147.86
141.56
140.96
128.66
128.31
127.28
126.80
125.30
119.84
77.32
77.00
76.68
54.40
3n
^{13}C-NMR (100 MHz, $CDCl_3$)
200 190 180 170 160 150 140 130 120 110 100 90 80 70 60 50 40 30 20 10 0
f1 (ppm)

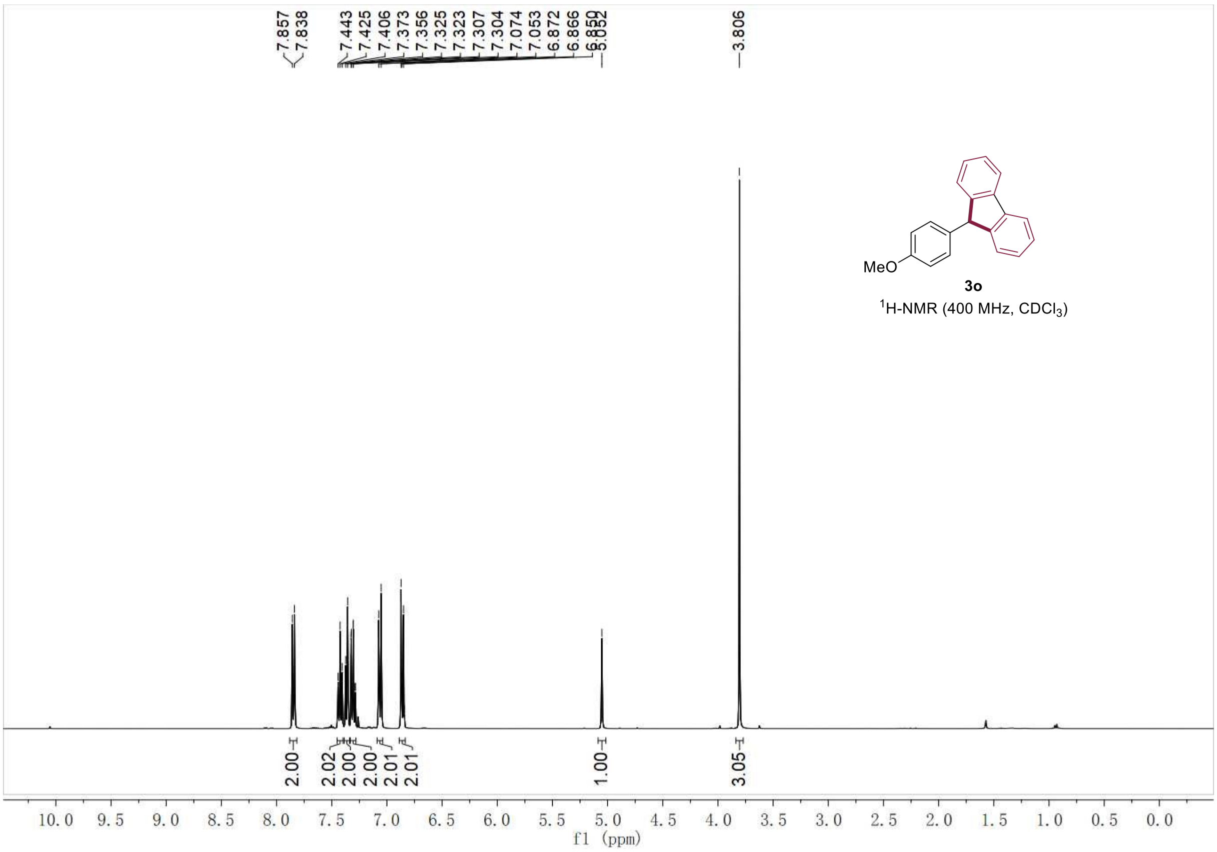
7.857
7.838
7.443
7.425
7.406
7.373
7.356
7.325
7.323
7.307
7.304
7.074
7.053
6.872
6.866
6.850
5.052
3.806
MeO
3o
^{1}H-NMR (400 MHz, $CDCl_3$)
2.00
2.02
2.00
2.00
2.01
2.01
1.00
3.05
10.0 9.5 9.0 8.5 8.0 7.5 7.0 6.5 6.0 5.5 5.0 4.5 4.0 3.5 3.0 2.5 2.0 1.5 1.0 0.5 0.0
f1 (ppm)

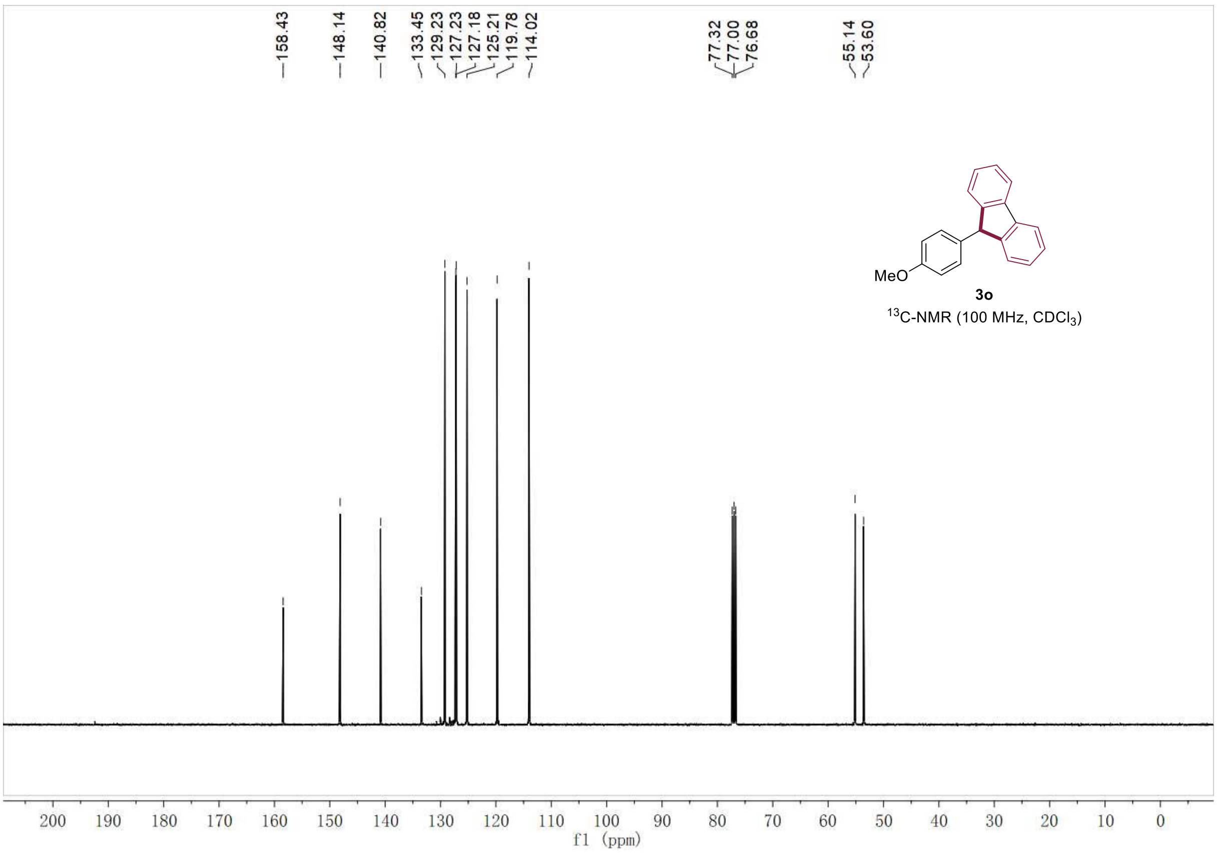
158.43
148.14
140.82
133.45
129.23
127.23
127.18
125.21
119.78
114.02
77.32
77.00
76.68
55.14
53.60
MeO
3o
^{13}C-NMR (100 MHz, CDCl$_3$)
200 190 180 170 160 150 140 130 120 110 100 90 80 70 60 50 40 30 20 10 0
f1 (ppm)

NMR Spectra of **5a-5v**

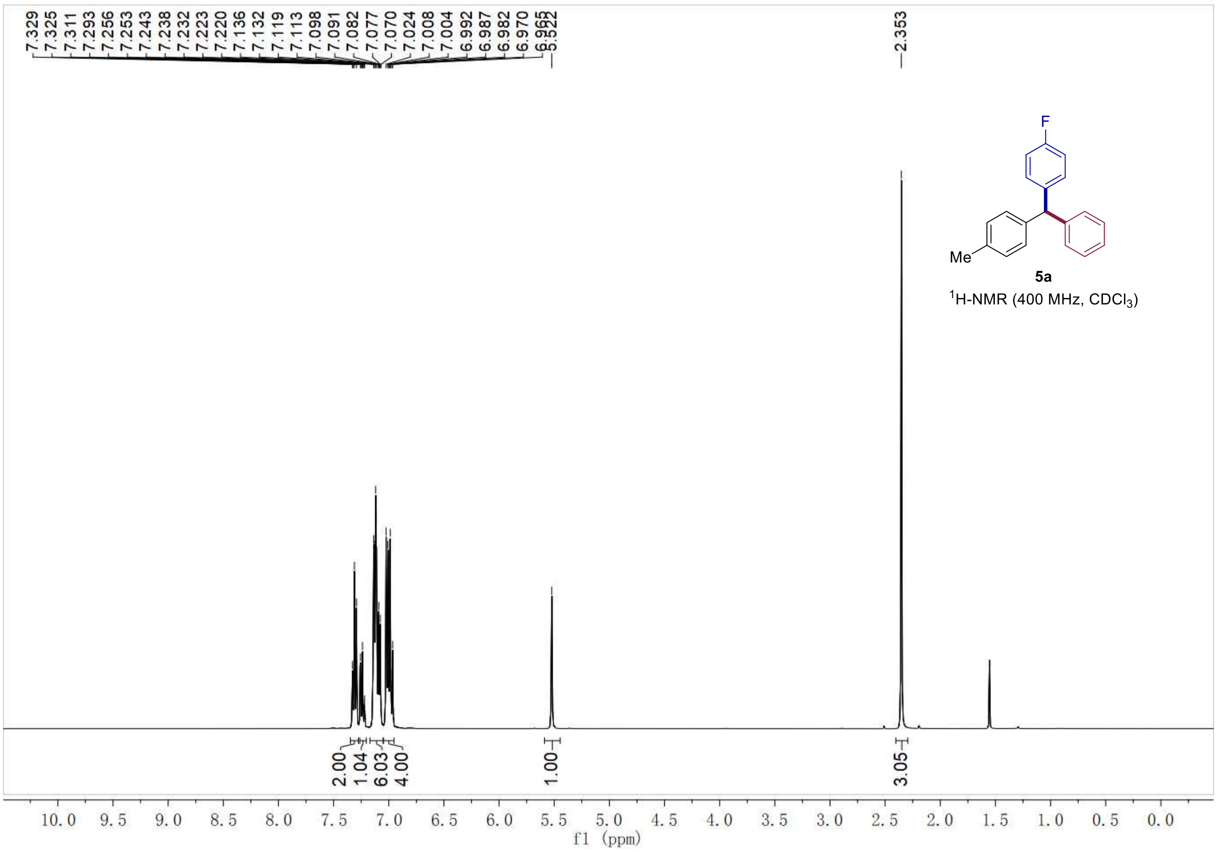

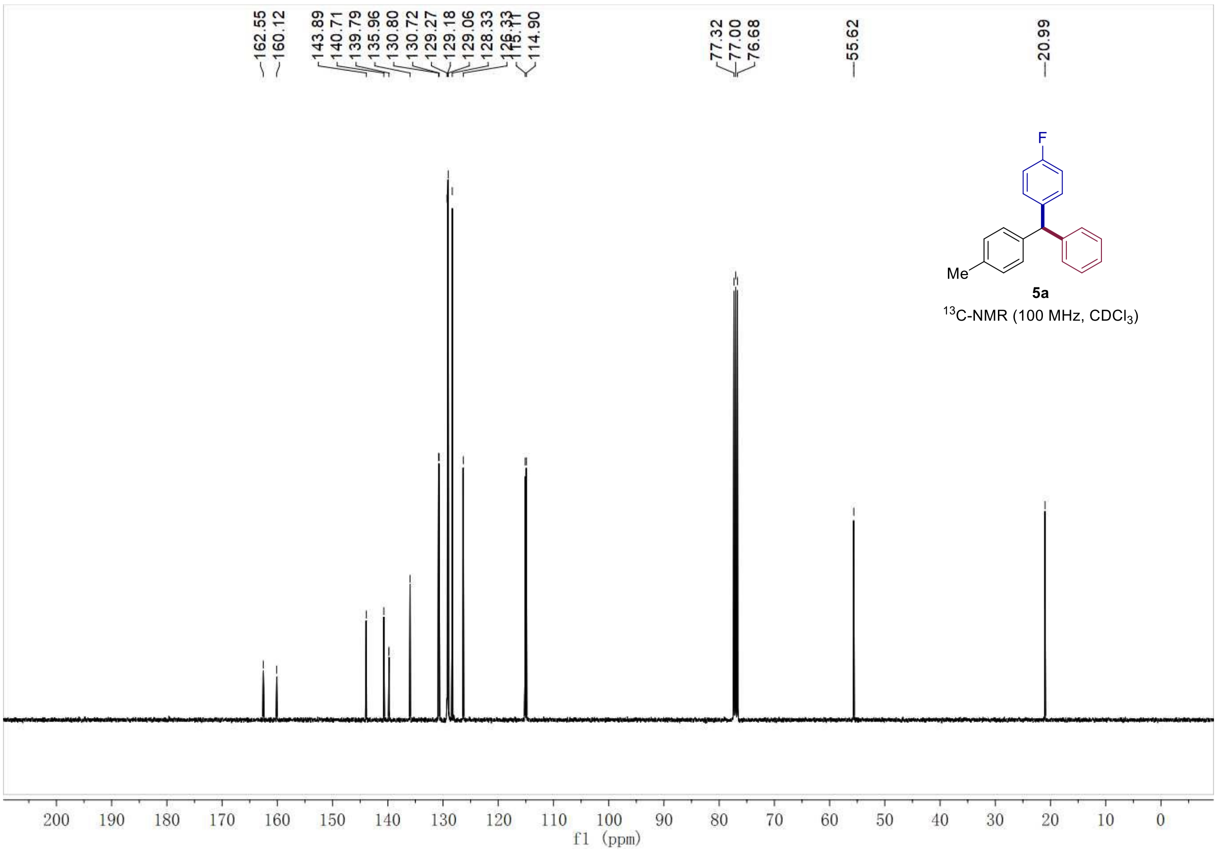

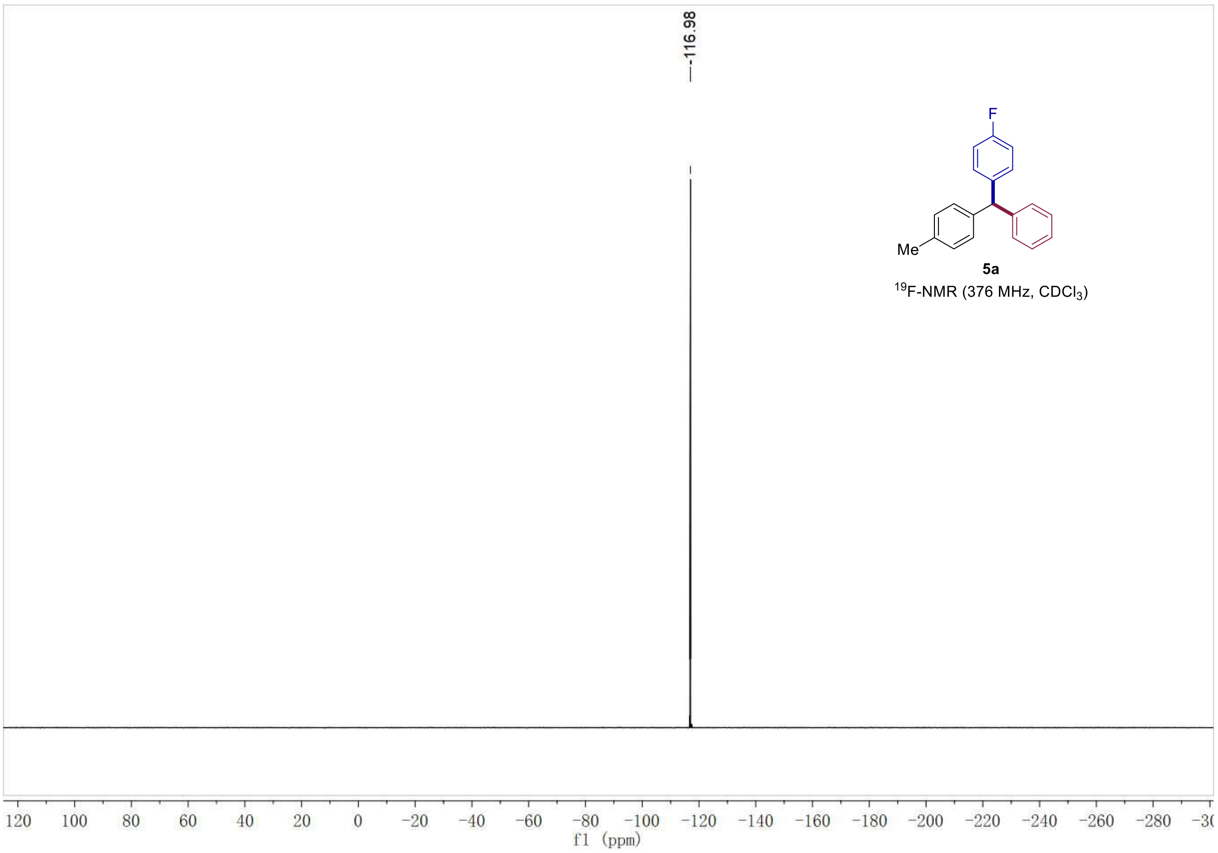

-116.98
F
Me
5a
^{19}F-NMR (376 MHz, CDCl$_3$)
120 100 80 60 40 20 0 -20 -40 -60 -80 -100 -120 -140 -160 -180 -200 -220 -240 -260 -280 -300
f1 (ppm)

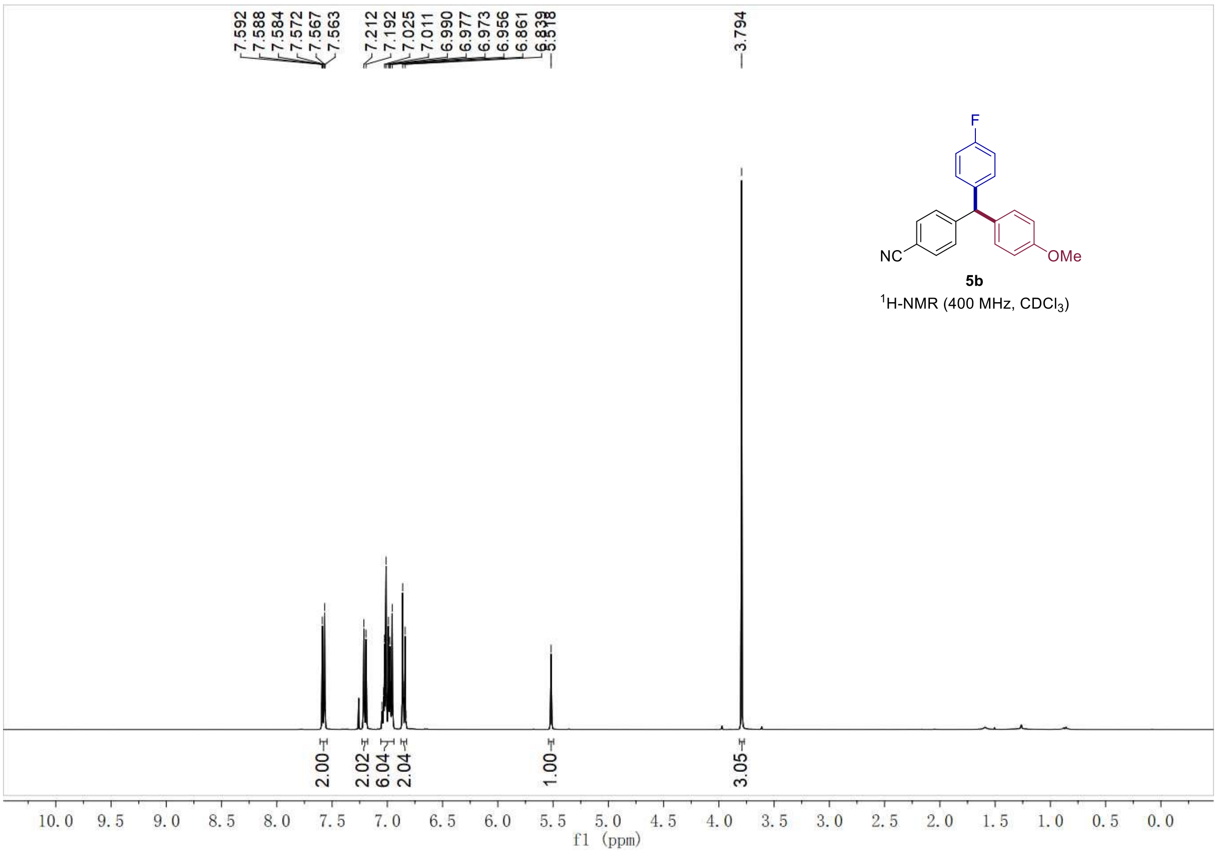

7.592 7.588 7.584 7.572 7.567 7.563 7.212 7.192 7.025 7.011 6.990 6.977 6.973 6.956 6.861 6.839 5.518
3.794
F
NC
OMe
5b
^{1}H-NMR (400 MHz, CDCl$_3$)
2.00 2.02 6.04 2.04 1.00 3.05
10.0 9.5 9.0 8.5 8.0 7.5 7.0 6.5 6.0 5.5 5.0 4.5 4.0 3.5 3.0 2.5 2.0 1.5 1.0 0.5 0.0
f1 (ppm)

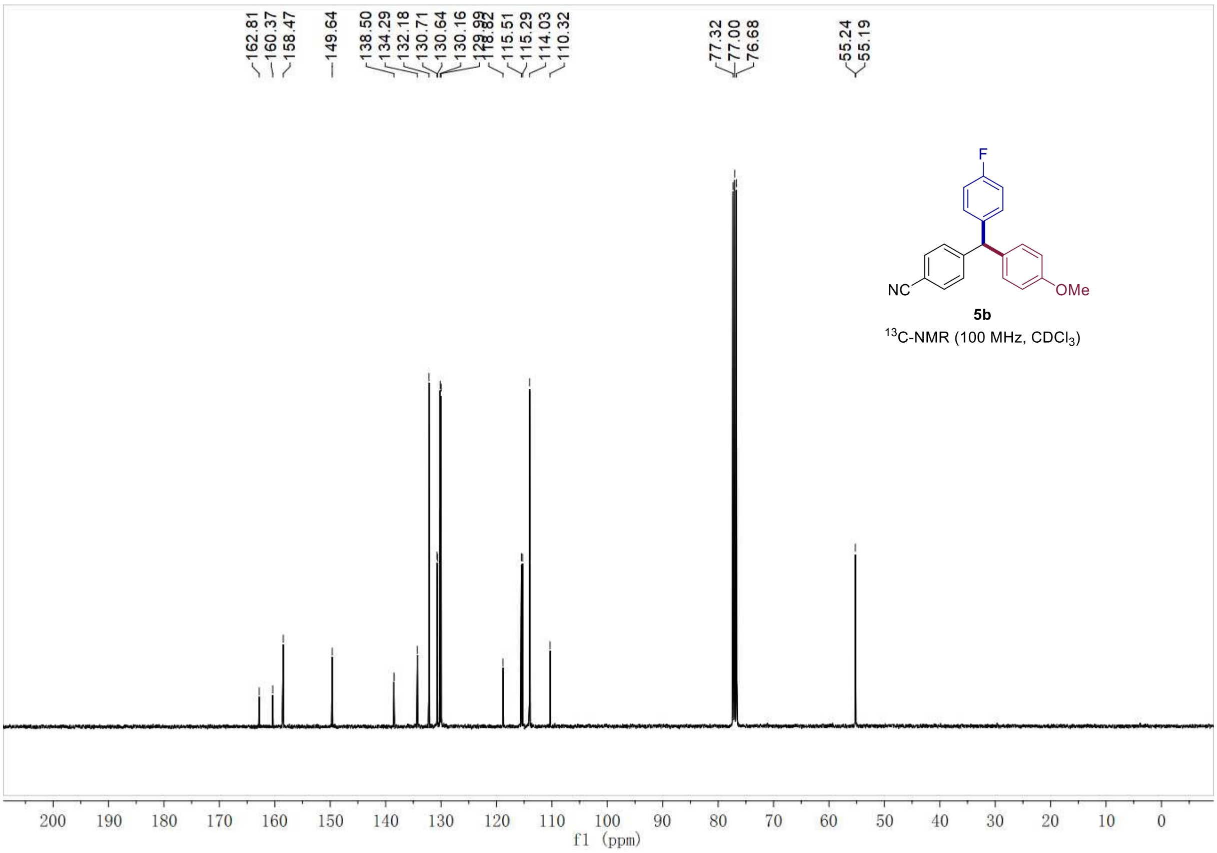
162.81
160.37
158.47
149.64
138.50
134.29
132.18
130.71
130.64
130.16
129.99
118.82
115.51
115.29
114.03
110.32
77.32
77.00
76.68
55.24
55.19
F
NC
OMe
5b
13C-NMR (100 MHz, CDCl3)
f1 (ppm)

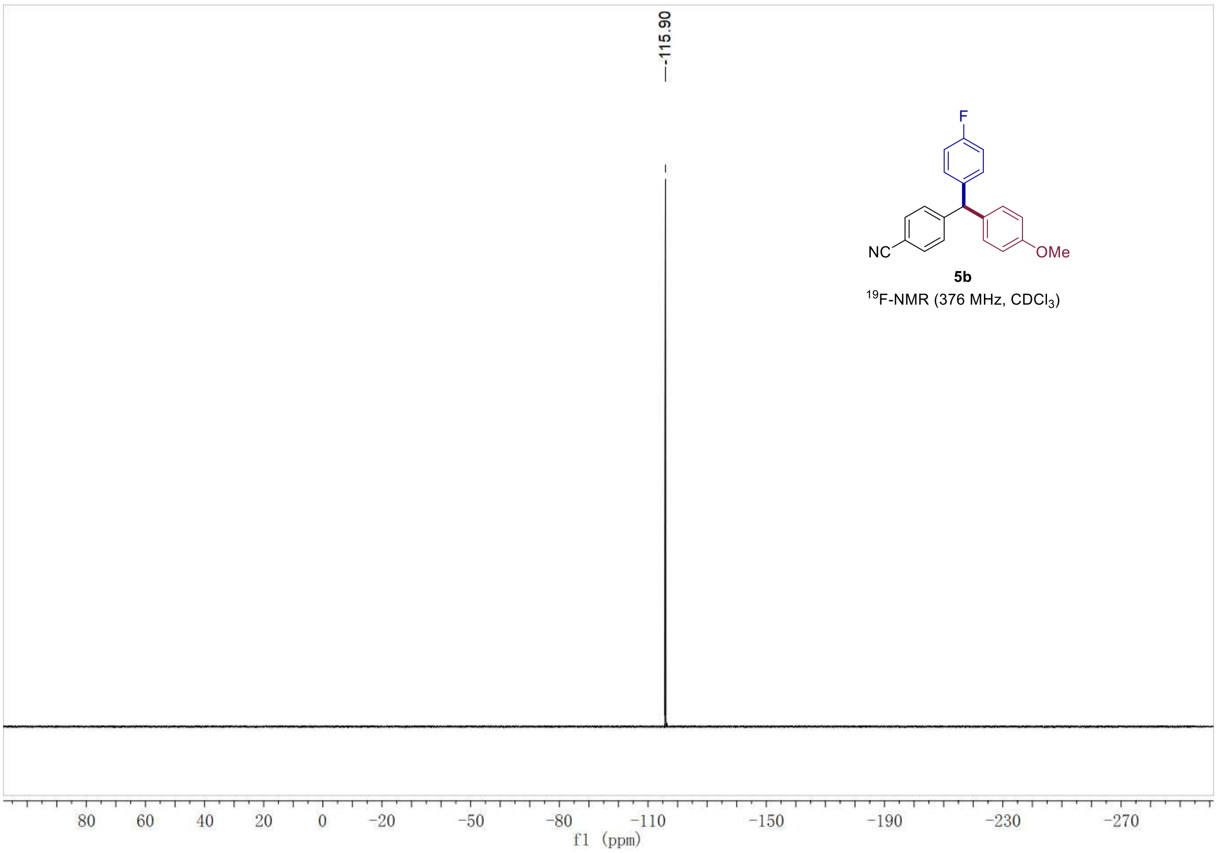
-115.90
F
NC
OMe
5b
19F-NMR (376 MHz, CDCl3)
f1 (ppm)

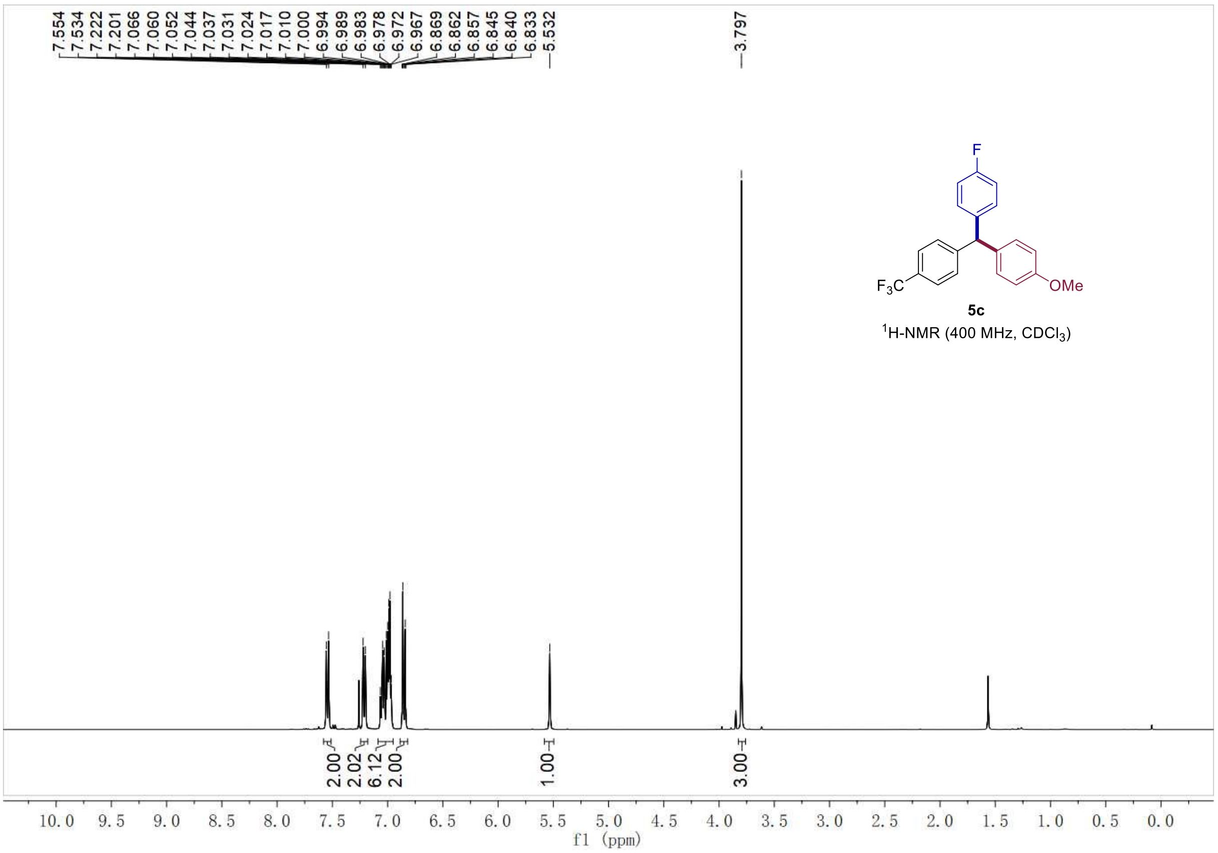
7.554
7.534
7.222
7.201
7.066
7.060
7.052
7.044
7.037
7.031
7.024
7.017
7.010
7.000
6.994
6.989
6.983
6.978
6.972
6.967
6.869
6.862
6.857
6.845
6.840
6.833
5.532
3.797
F
F3C
OMe
5c
1H-NMR (400 MHz, CDCl3)
2.00
2.02
6.12
2.00
1.00
3.00
10.0 9.5 9.0 8.5 8.0 7.5 7.0 6.5 6.0 5.5 5.0 4.5 4.0 3.5 3.0 2.5 2.0 1.5 1.0 0.5 0.0
f1 (ppm)

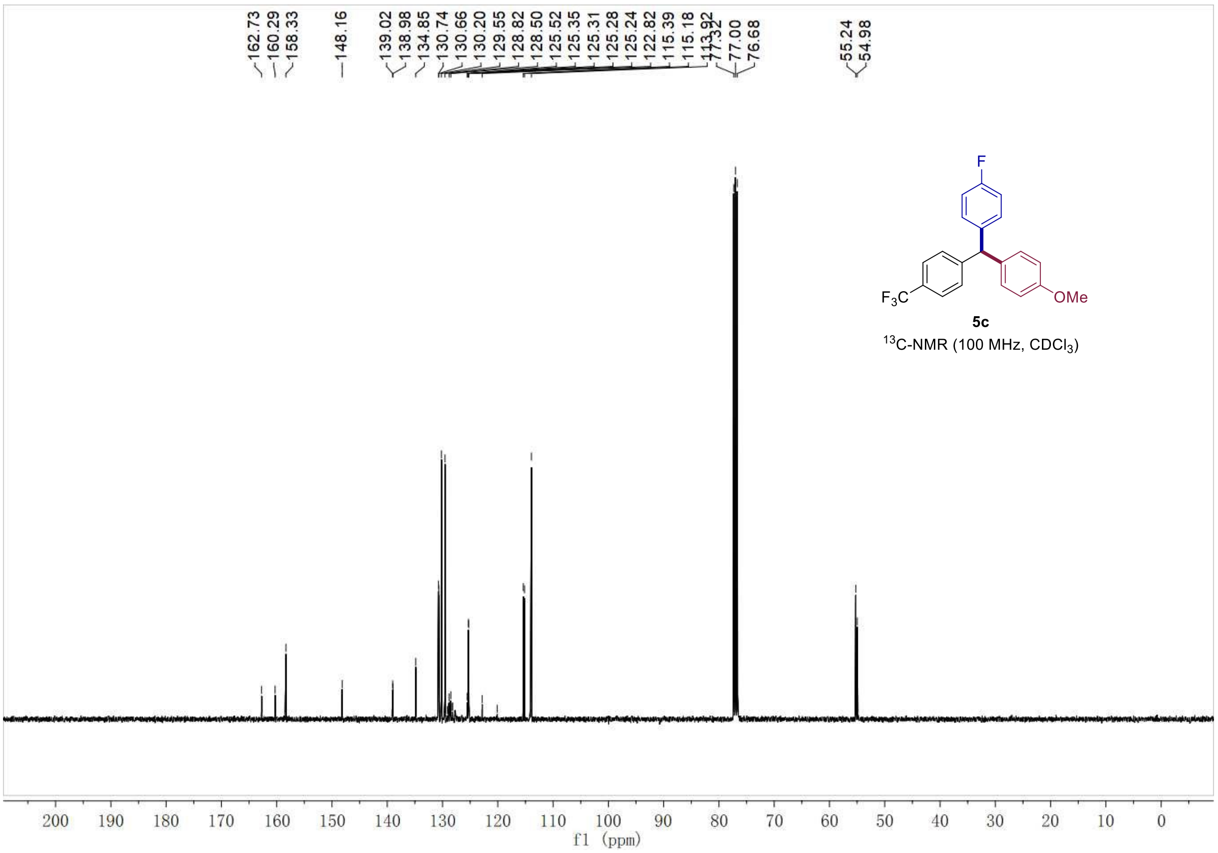
162.73
160.29
158.33
148.16
139.02
138.98
134.85
130.74
130.66
130.20
129.55
128.82
128.50
125.52
125.35
125.31
125.28
125.24
122.82
115.39
115.18
113.92
77.32
77.00
76.68
55.24
54.98
F
F3C
OMe
5c
13C-NMR (100 MHz, CDCl3)
200 190 180 170 160 150 140 130 120 110 100 90 80 70 60 50 40 30 20 10 0
f1 (ppm)

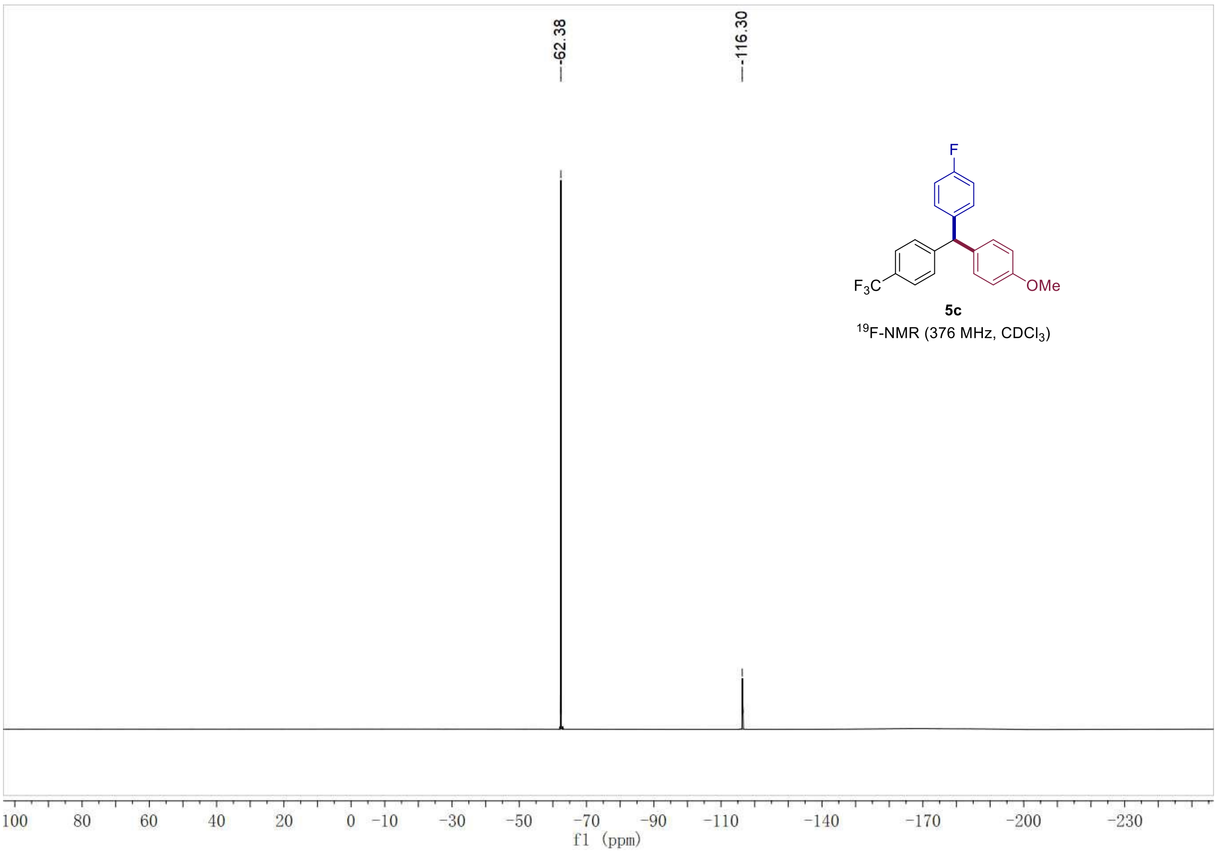
-62.38
-116.30
F
F3C
OMe
5c
19F-NMR (376 MHz, CDCl3)
100 80 60 40 20 0 -10 -30 -50 -70 -90 -110 -140 -170 -200 -230
f1 (ppm)

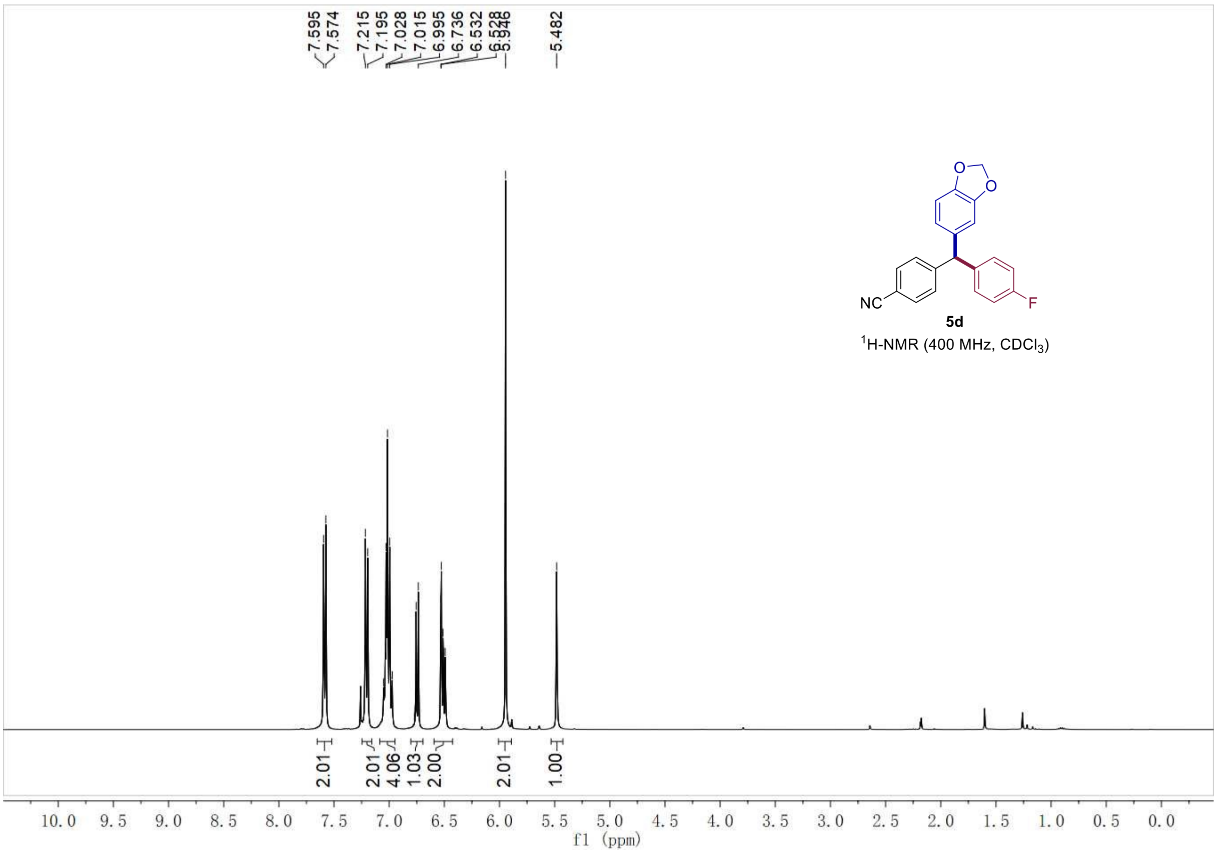
7.595
7.574
7.215
7.195
7.028
7.015
6.995
6.736
6.532
6.528
5.946
5.482
O
O
NC
F
5d
1H-NMR (400 MHz, CDCl3)
2.01
2.01
4.06
1.03
2.00
2.01
1.00
10.0 9.5 9.0 8.5 8.0 7.5 7.0 6.5 6.0 5.5 5.0 4.5 4.0 3.5 3.0 2.5 2.0 1.5 1.0 0.5 0.0
f1 (ppm)

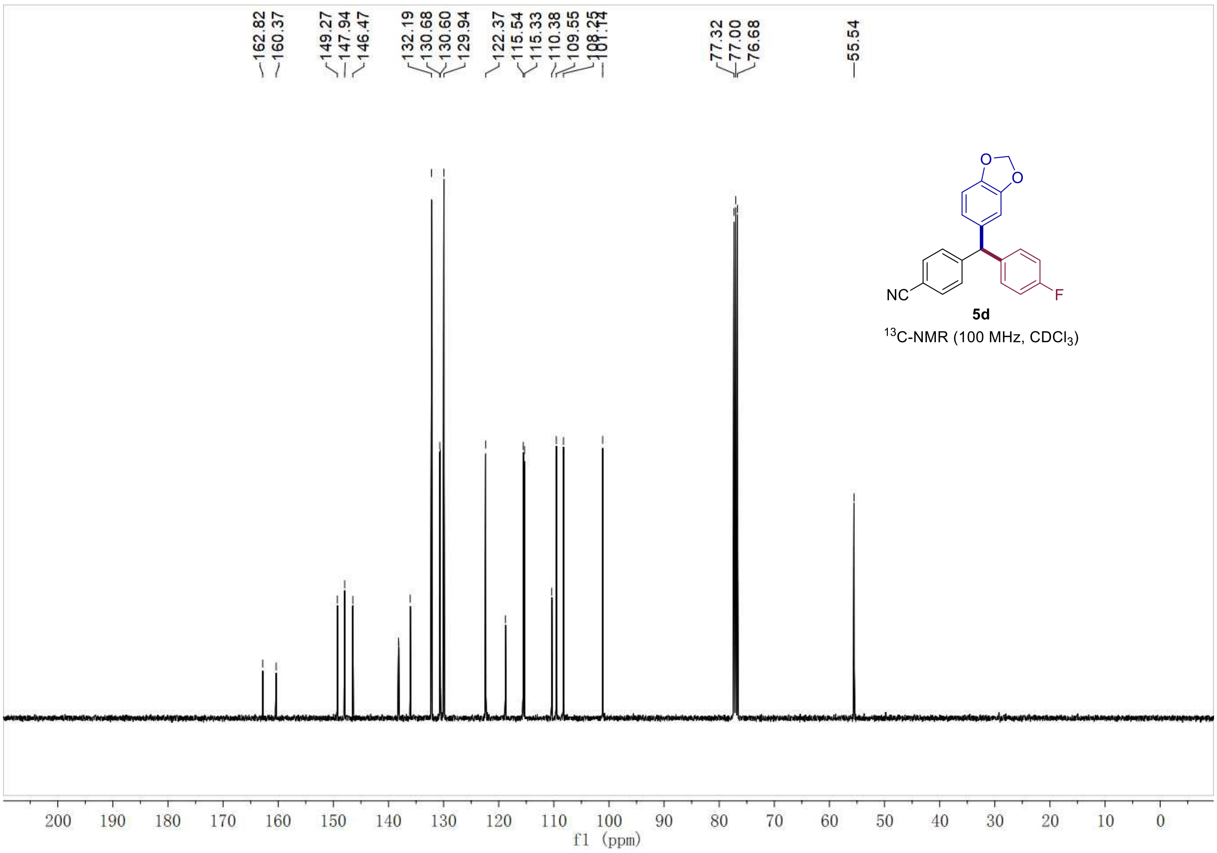
162.82
160.37
149.27
147.94
146.47
132.19
130.68
130.60
129.94
122.37
115.54
115.33
110.38
109.55
108.25
101.14
77.32
77.00
76.68
55.54
NC
O
O
F
5d
13C-NMR (100 MHz, CDCl3)
f1 (ppm)

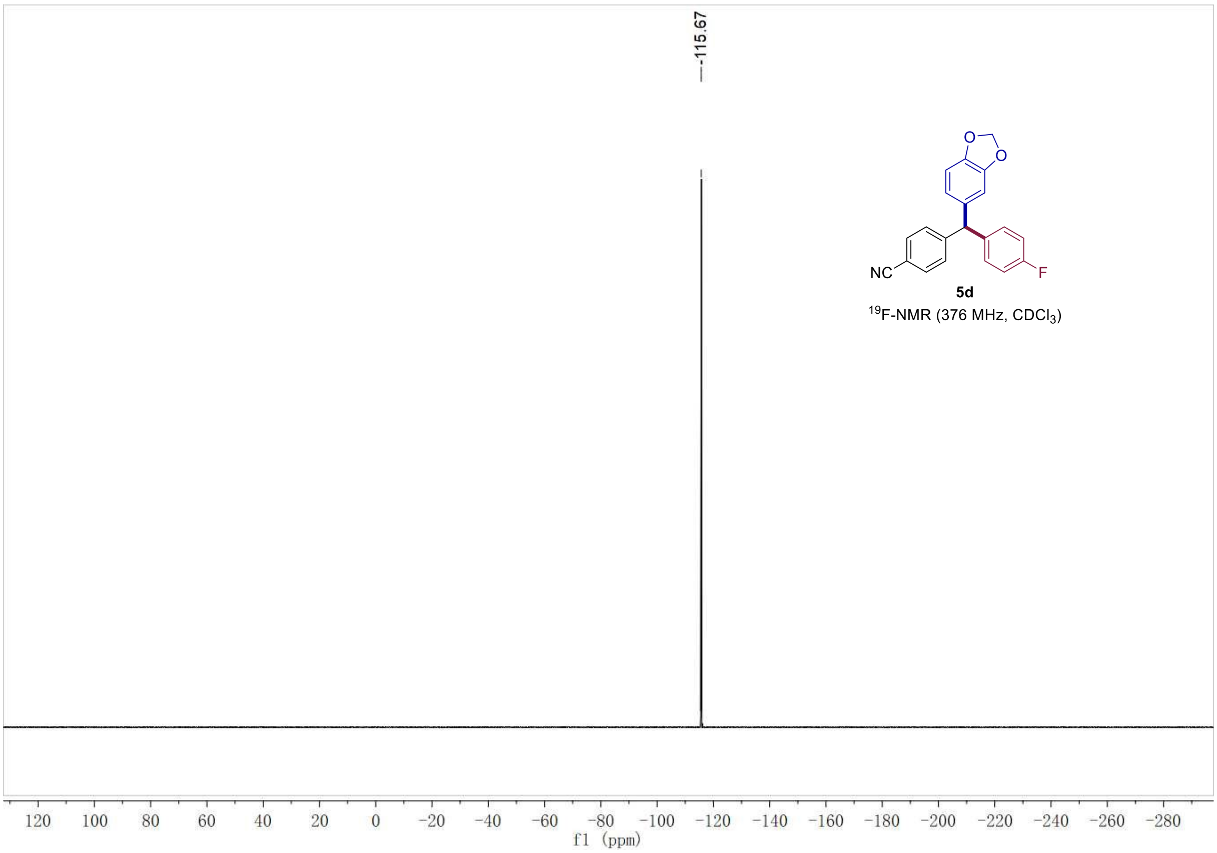
-115.67
NC
O
O
F
5d
19F-NMR (376 MHz, CDCl3)
f1 (ppm)

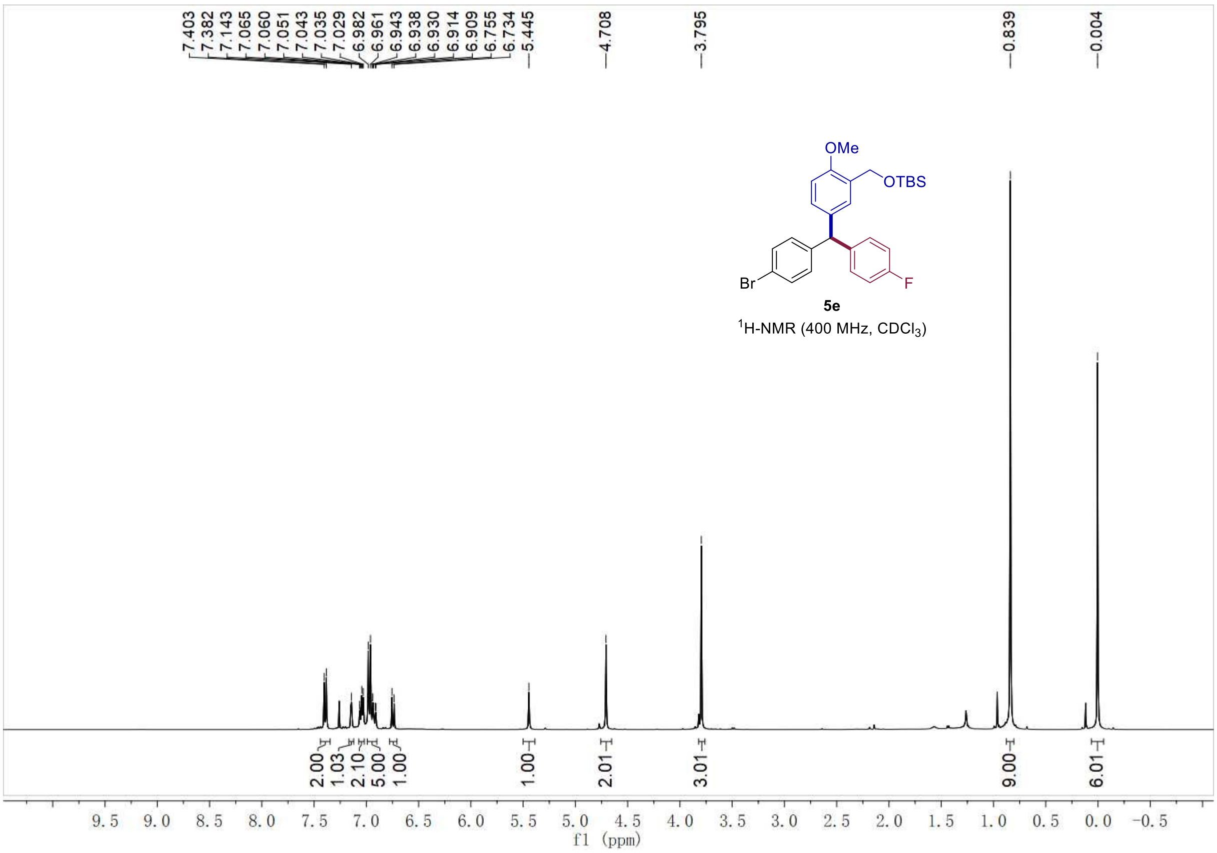
7.403
7.382
7.143
7.065
7.060
7.051
7.043
7.035
7.029
6.982
6.961
6.943
6.938
6.930
6.914
6.909
6.755
6.734
5.445
4.708
3.795
0.839
0.004
OMe
OTBS
Br
F
5e
^{1}H-NMR (400 MHz, CDCl$_3$)
2.00
1.03
2.10
5.00
1.00
1.00
2.01
3.01
9.00
6.01
9.5 9.0 8.5 8.0 7.5 7.0 6.5 6.0 5.5 5.0 4.5 4.0 3.5 3.0 2.5 2.0 1.5 1.0 0.5 0.0 -0.5
f1 (ppm)

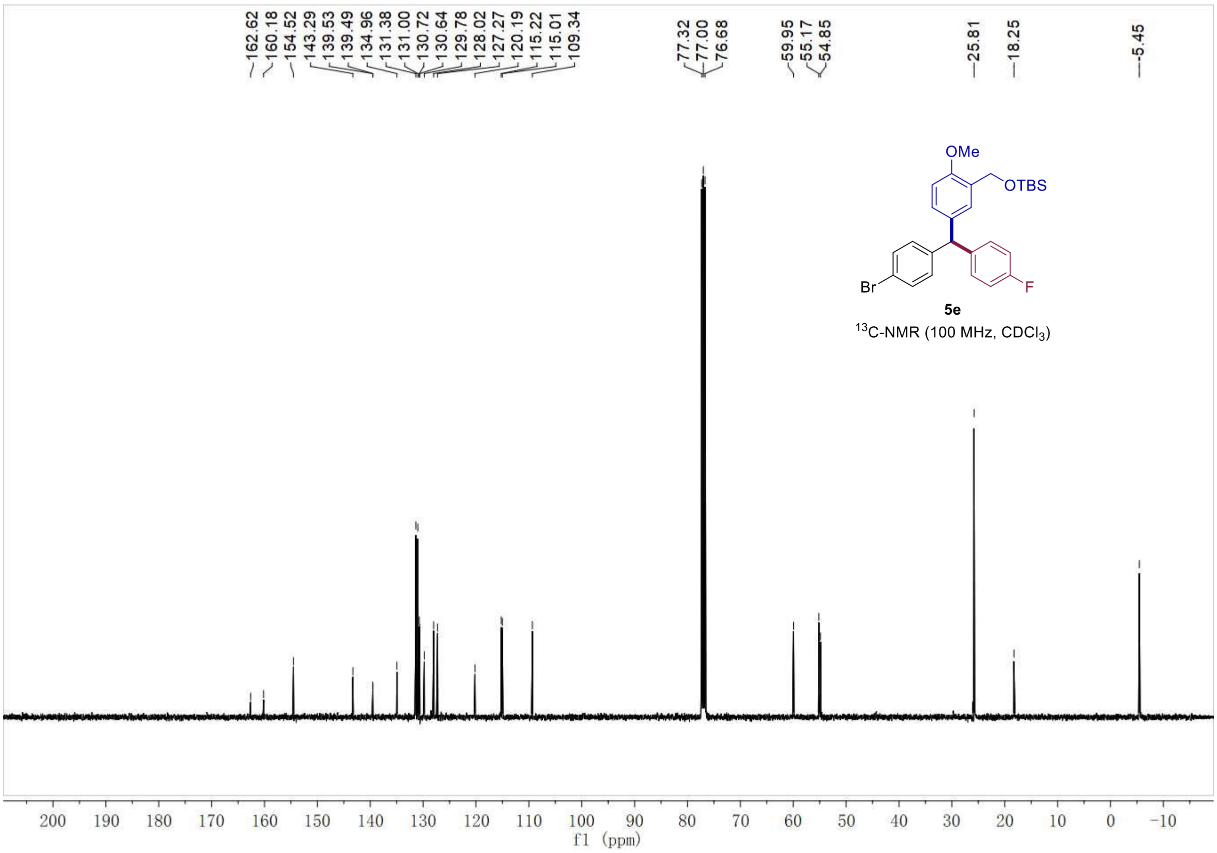
162.62
160.18
154.52
143.29
139.53
139.49
134.96
131.38
131.00
130.72
130.64
129.78
128.02
127.27
120.19
115.22
115.01
109.34
77.32
77.00
76.68
59.95
55.17
54.85
25.81
18.25
-5.45
OMe
OTBS
Br
F
5e
^{13}C-NMR (100 MHz, CDCl$_3$)
200 190 180 170 160 150 140 130 120 110 100 90 80 70 60 50 40 30 20 10 0 -10
f1 (ppm)

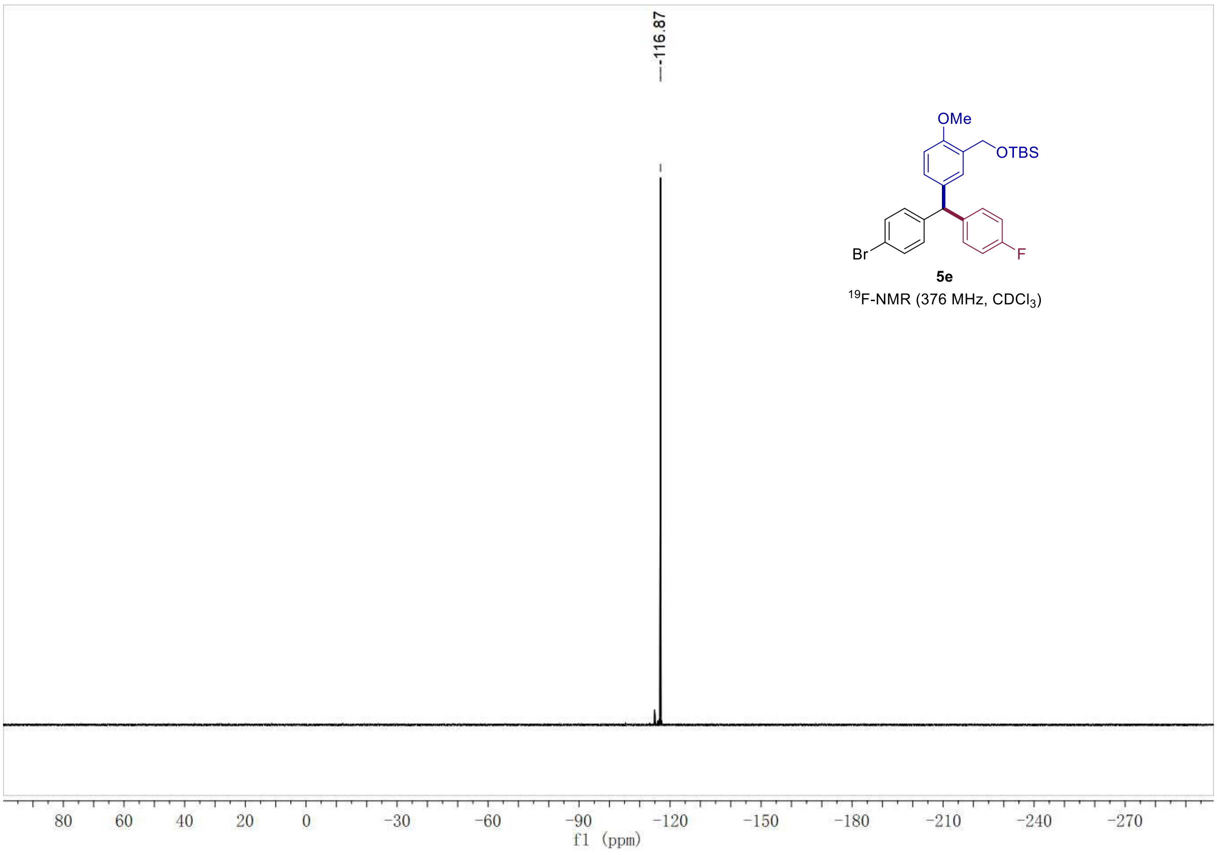
-116.87
OMe
OTBS
Br
F
5e
19F-NMR (376 MHz, CDCl3)
80 60 40 20 0 -30 -60 -90 -120 -150 -180 -210 -240 -270
f1 (ppm)

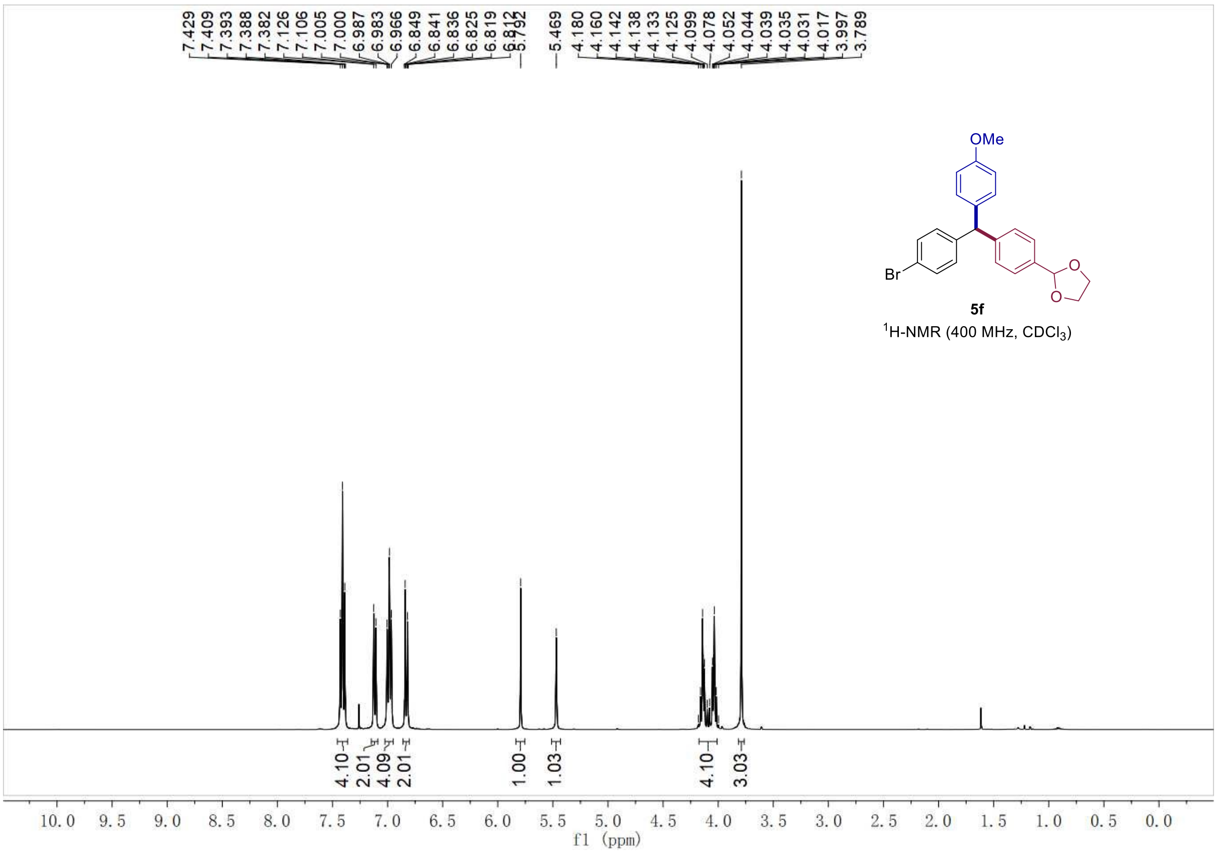
7.429 7.409 7.393 7.388 7.382 7.126 7.106 7.005 7.000 6.987 6.983 6.966 6.849 6.841 6.836 6.825 6.819 6.812 5.792 5.469 4.180 4.160 4.142 4.138 4.133 4.125 4.099 4.078 4.052 4.044 4.039 4.035 4.031 4.017 3.997 3.789
OMe
Br
O
O
5f
1H-NMR (400 MHz, CDCl3)
4.10 2.01 4.09 2.01 1.00 1.03 4.10 3.03
10.0 9.5 9.0 8.5 8.0 7.5 7.0 6.5 6.0 5.5 5.0 4.5 4.0 3.5 3.0 2.5 2.0 1.5 1.0 0.5 0.0
f1 (ppm)

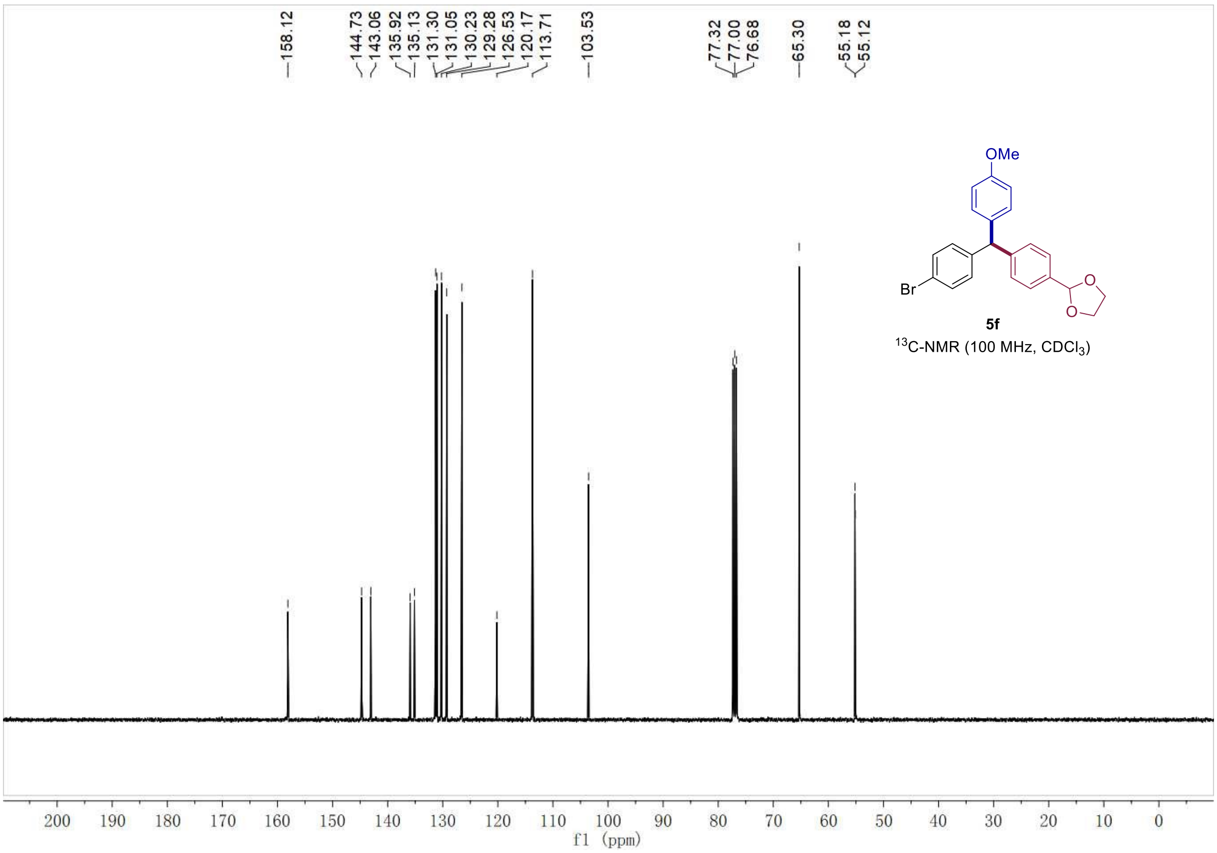
158.12
144.73
143.06
135.92
135.13
131.30
131.05
130.23
129.28
126.53
120.17
113.71
103.53
77.32
77.00
76.68
65.30
55.18
55.12
OMe
Br
O
O
5f
13C-NMR (100 MHz, CDCl3)
f1 (ppm)

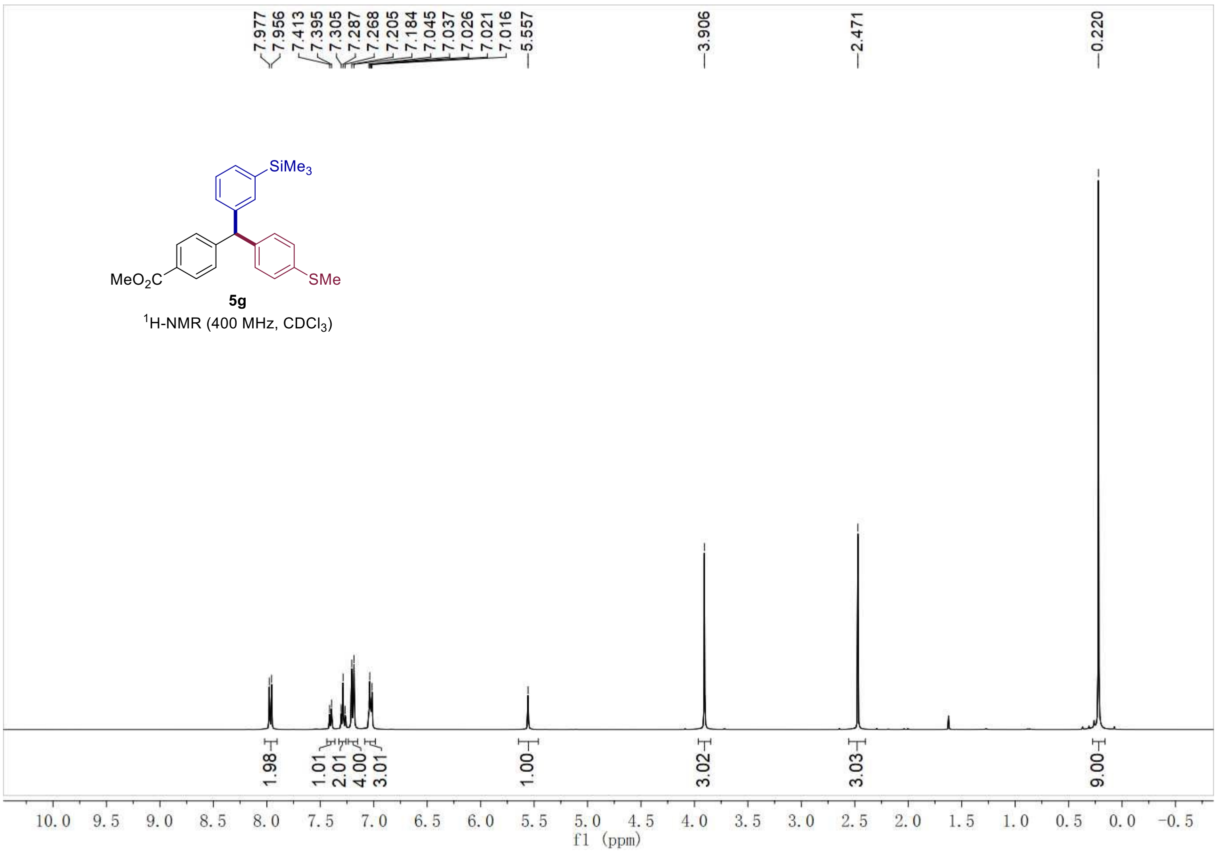
7.977
7.956
7.413
7.395
7.305
7.287
7.268
7.205
7.184
7.045
7.037
7.026
7.021
7.016
5.557
3.906
2.471
0.220
SiMe3
MeO2C
SMe
5g
1H-NMR (400 MHz, CDCl3)
1.98
1.01
2.01
4.00
3.01
1.00
3.02
3.03
9.00
f1 (ppm)

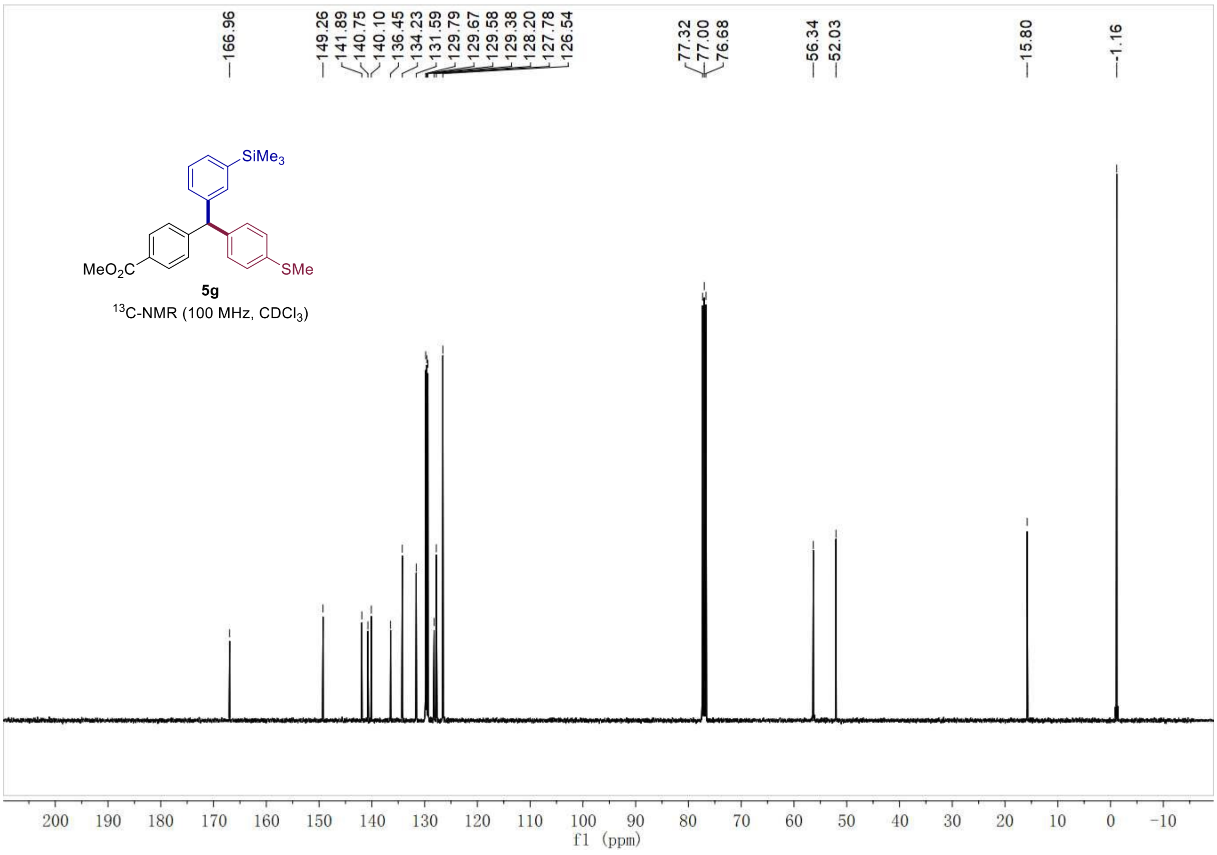

166.96
149.26
141.89
140.75
140.10
136.45
134.23
131.59
129.79
129.67
129.58
129.38
128.20
127.78
126.54
77.32
77.00
76.68
56.34
52.03
15.80
-1.16
SiMe3
MeO2C
SMe
5g
13C-NMR (100 MHz, CDCl3)
f1 (ppm)

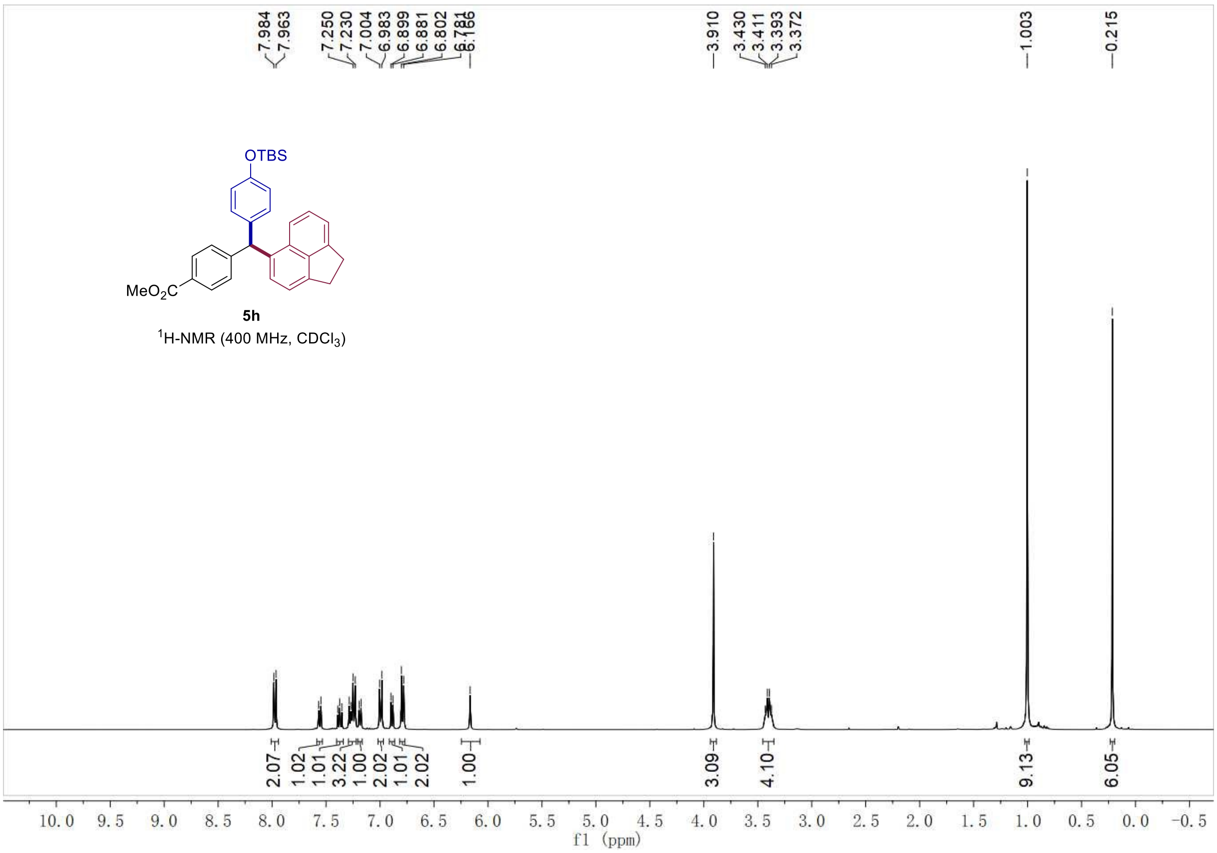

7.984
7.963
7.250
7.230
7.004
6.983
6.899
6.881
6.802
3.910
3.430
3.411
3.393
3.372
1.003
0.215
OTBS
MeO2C
5h
1H-NMR (400 MHz, CDCl3)
2.07
1.02
1.01
3.22
1.00
2.02
1.01
2.02
1.00
3.09
4.10
9.13
6.05
f1 (ppm)

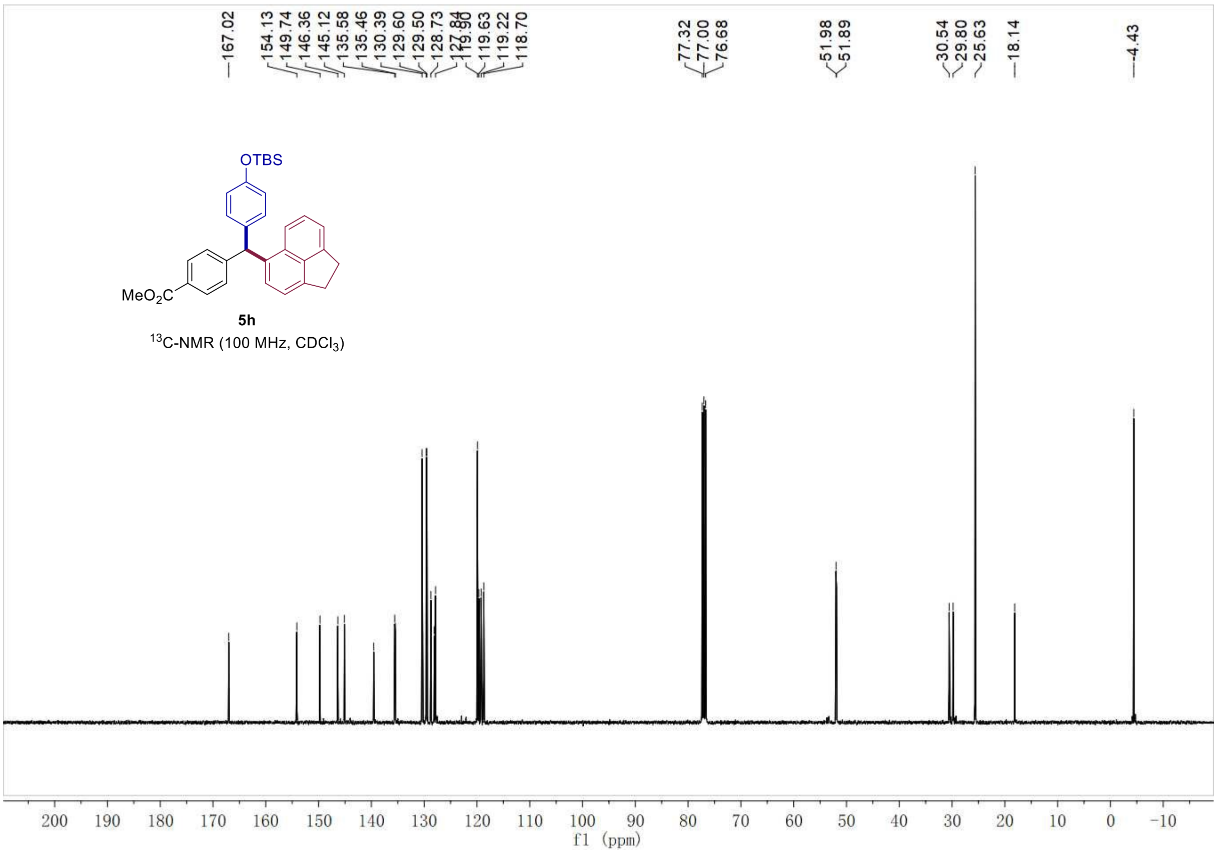

167.02
154.13
149.74
146.36
145.12
135.58
135.46
130.39
129.60
129.50
128.73
119.63
119.22
118.70
77.32
77.00
76.68
51.98
51.89
30.54
29.80
25.63
18.14
-4.43
OTBS
MeO2C
5h
13C-NMR (100 MHz, CDCl3)
200 190 180 170 160 150 140 130 120 110 100 90 80 70 60 50 40 30 20 10 0 -10
f1 (ppm)

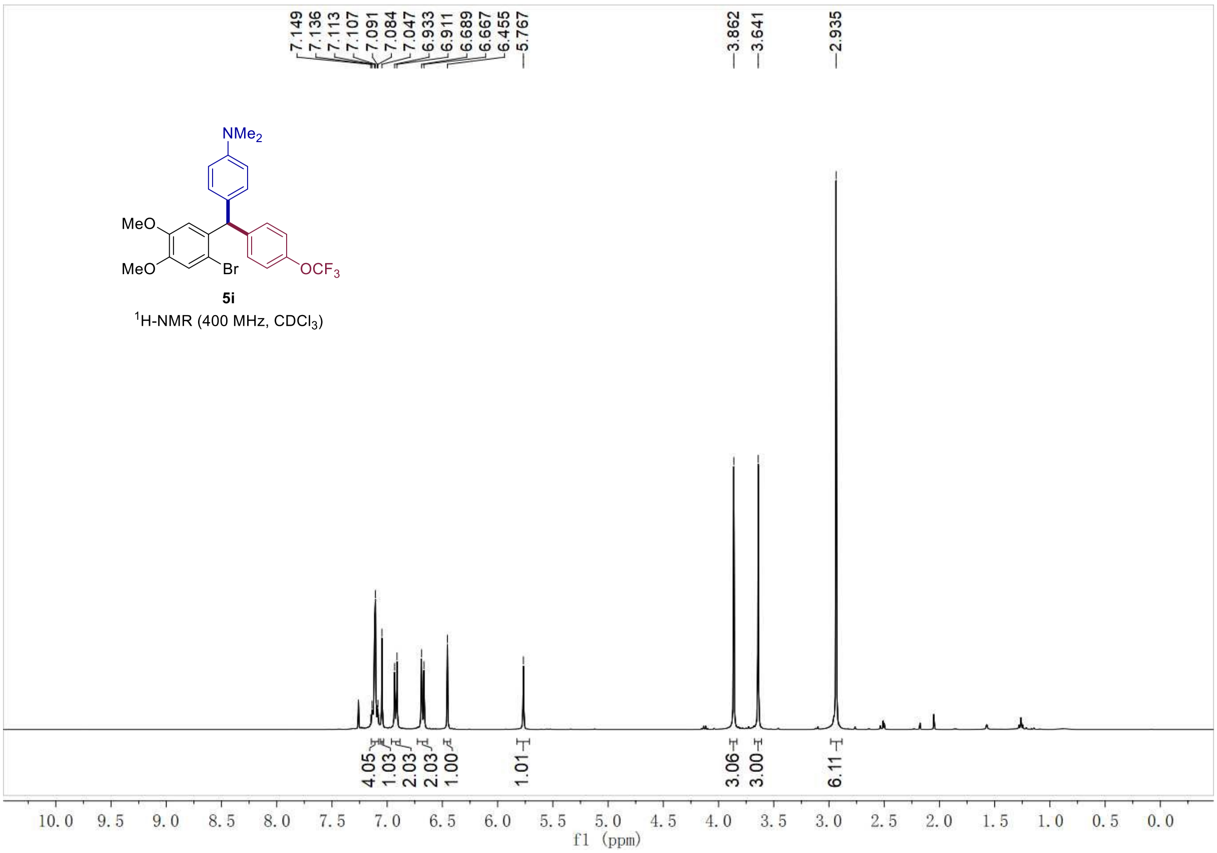

7.149
7.136
7.113
7.107
7.091
7.084
7.047
6.933
6.911
6.689
6.667
6.455
5.767
3.862
3.641
2.935
NMe2
MeO
MeO
Br
OCF3
5i
1H-NMR (400 MHz, CDCl3)
4.05
1.03
2.03
2.03
1.00
1.01
3.06
3.00
6.11
10.0 9.5 9.0 8.5 8.0 7.5 7.0 6.5 6.0 5.5 5.0 4.5 4.0 3.5 3.0 2.5 2.0 1.5 1.0 0.5 0.0
f1 (ppm)

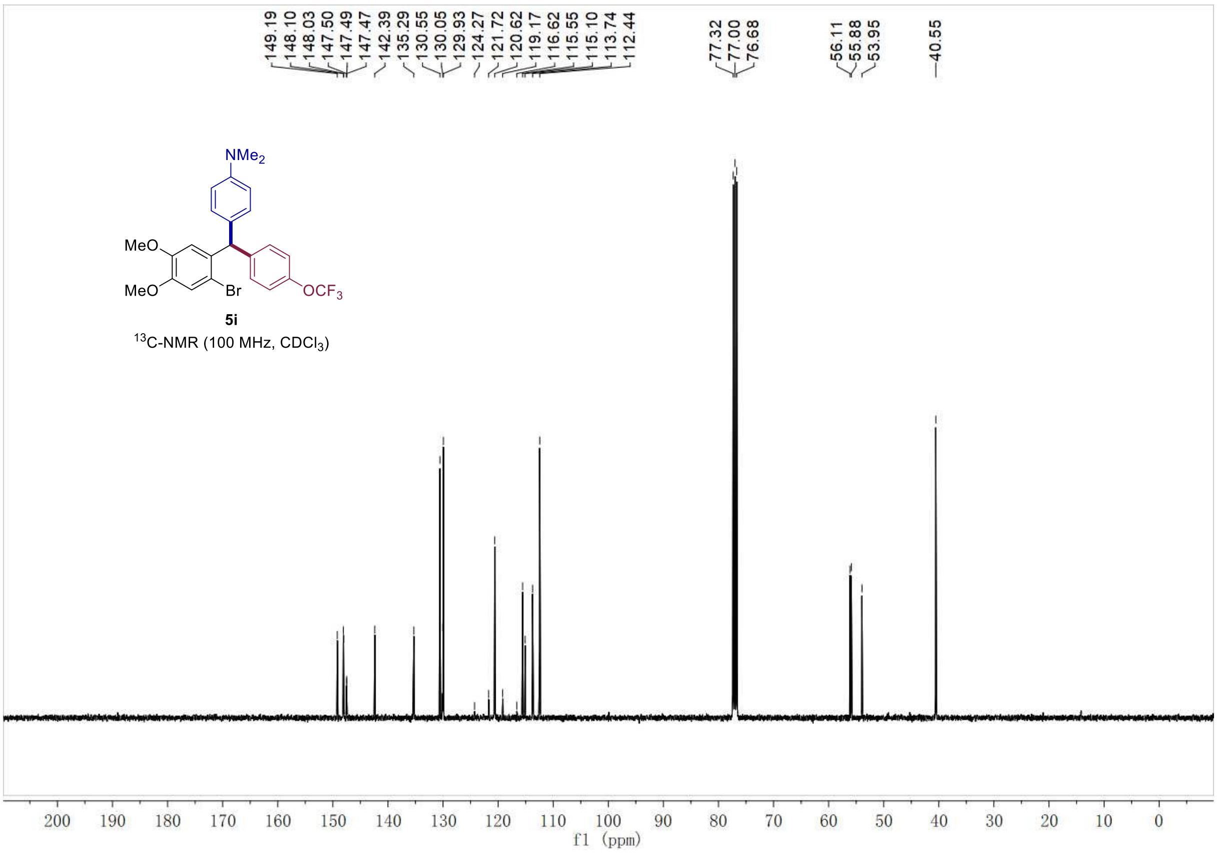
149.19
148.10
148.03
147.50
147.49
147.47
142.39
135.29
130.55
130.05
129.93
124.27
121.72
120.62
119.17
116.62
115.55
115.10
113.74
112.44
77.32
77.00
76.68
56.11
55.88
53.95
40.55
NMe2
MeO
MeO
Br
OCF3
5i
13C-NMR (100 MHz, CDCl3)
f1 (ppm)

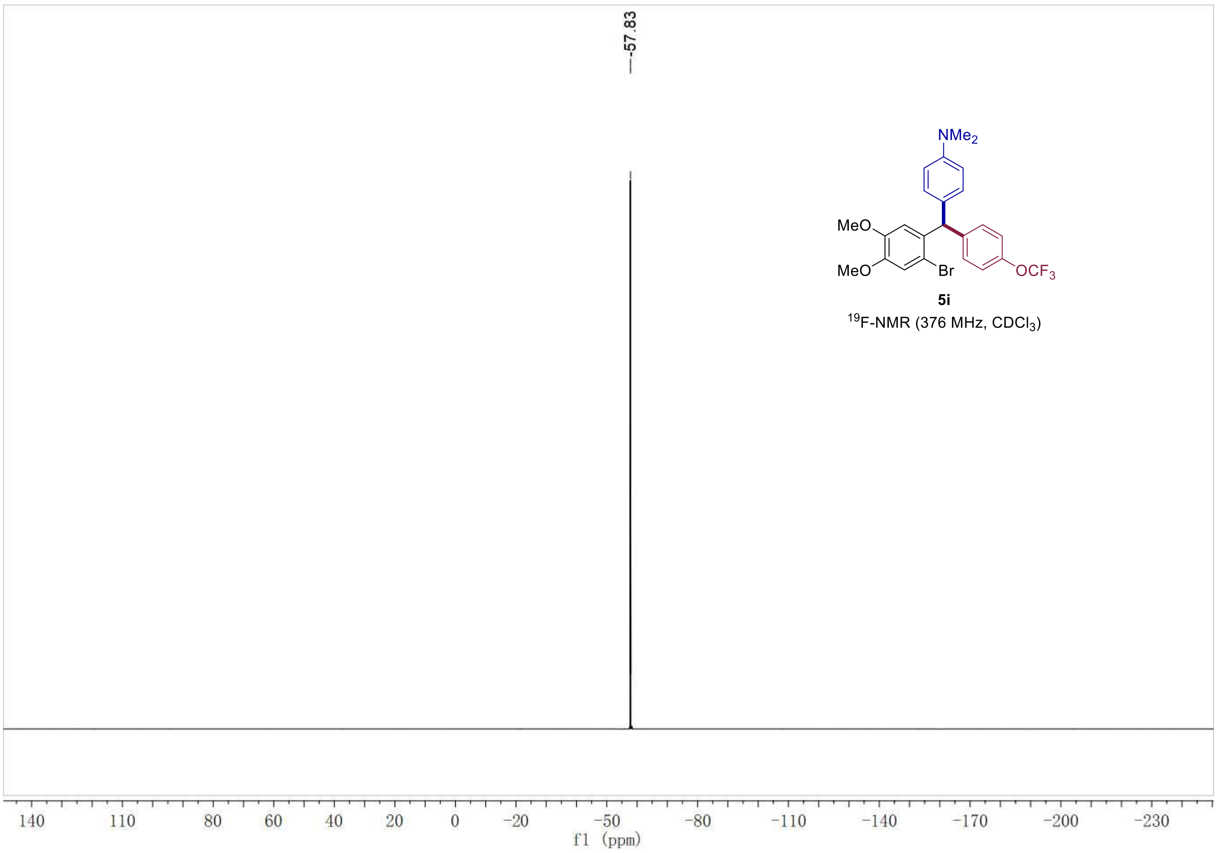
-57.83
NMe2
MeO
MeO
Br
OCF3
5i
19F-NMR (376 MHz, CDCl3)
f1 (ppm)

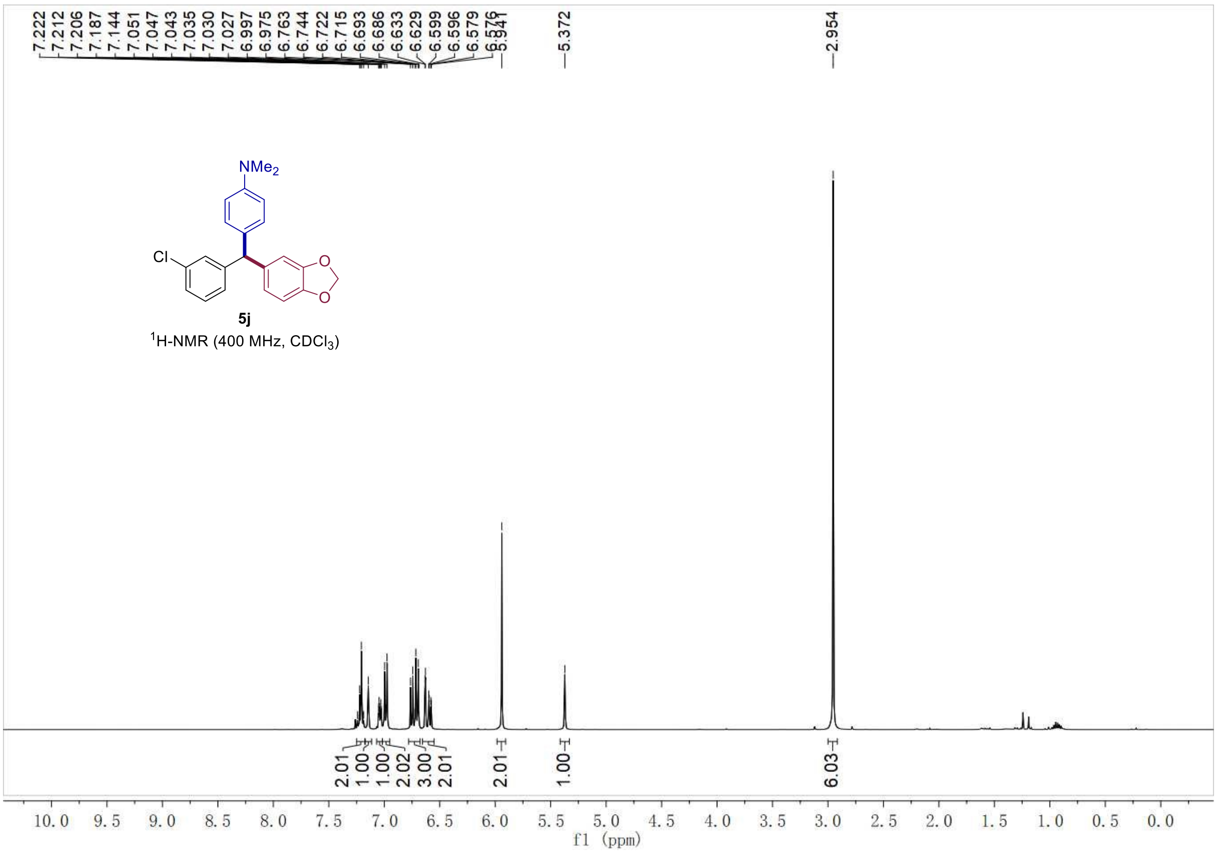
7.222
7.212
7.206
7.187
7.144
7.051
7.047
7.043
7.035
7.030
7.027
6.997
6.975
6.763
6.744
6.722
6.715
6.693
6.686
6.633
6.629
6.599
6.596
6.579
6.576
5.941
5.372
2.954
NMe2
Cl
O
O
5j
1H-NMR (400 MHz, CDCl3)
2.01
1.00
1.00
2.02
3.00
2.01
2.01
1.00
6.03
10.0 9.5 9.0 8.5 8.0 7.5 7.0 6.5 6.0 5.5 5.0 4.5 4.0 3.5 3.0 2.5 2.0 1.5 1.0 0.5 0.0
f1 (ppm)

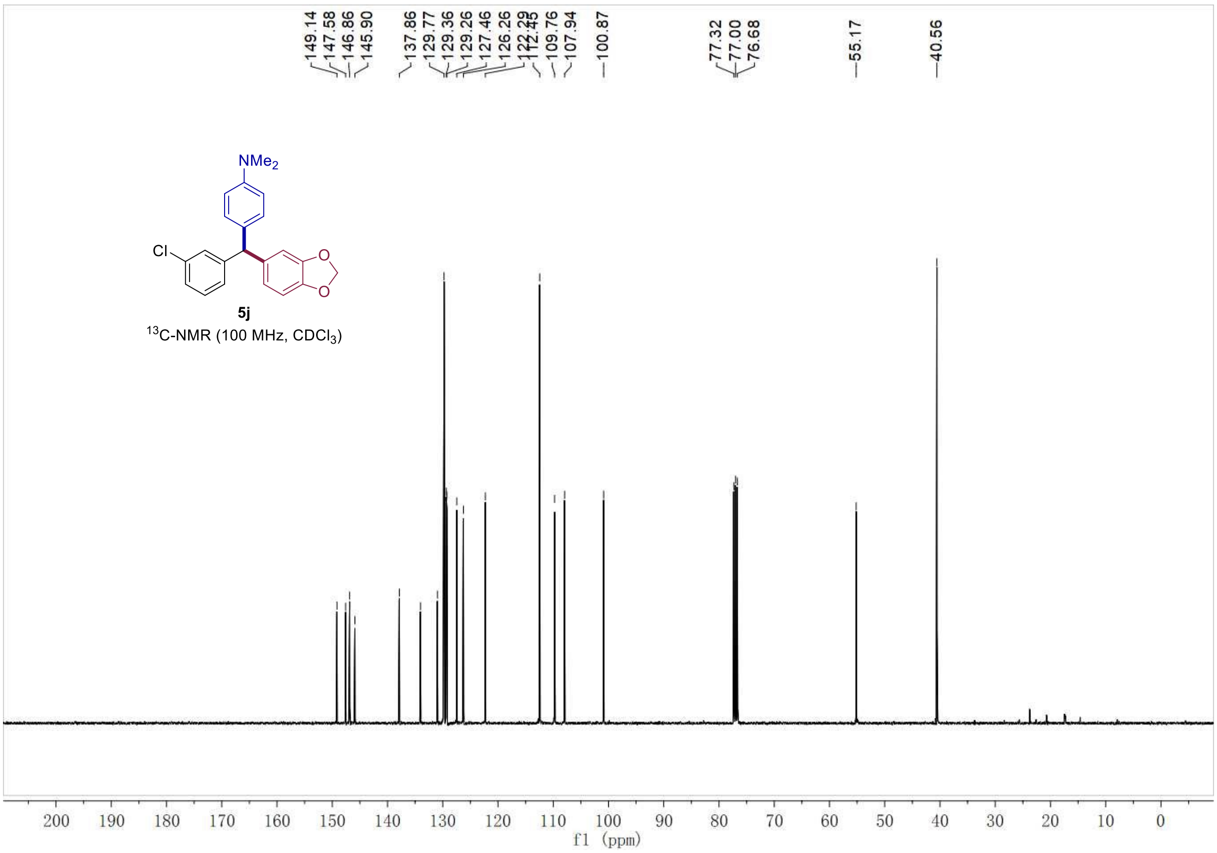
149.14
147.58
146.86
145.90
137.86
129.77
129.36
129.26
127.46
126.26
122.29
112.45
109.76
107.94
100.87
77.32
77.00
76.68
55.17
40.56
NMe2
Cl
O
O
5j
13C-NMR (100 MHz, CDCl3)
200 190 180 170 160 150 140 130 120 110 100 90 80 70 60 50 40 30 20 10 0
f1 (ppm)

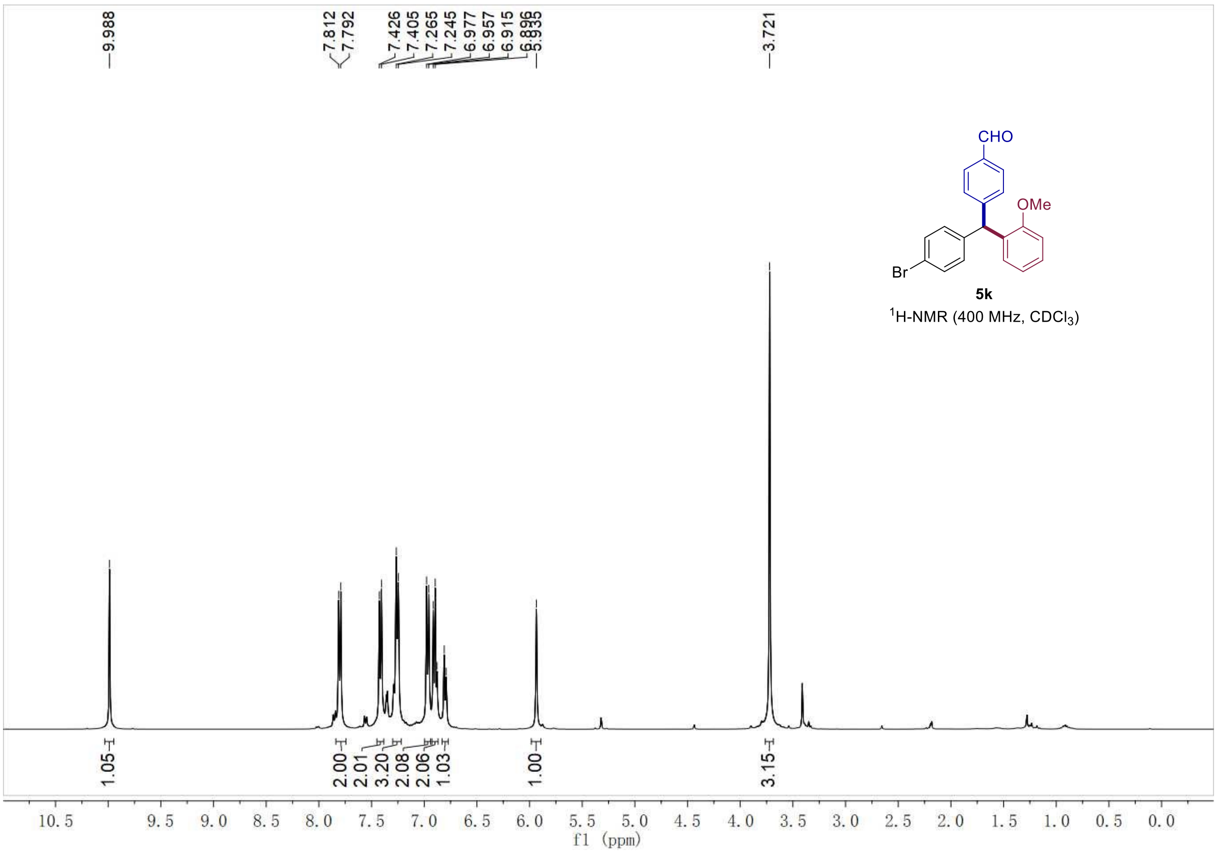
9.988
7.812
7.792
7.426
7.405
7.265
7.245
6.977
6.957
6.915
3.721
CHO
OMe
Br
5k
^{1}H-NMR (400 MHz, $CDCl_3$)
1.05
2.00
2.01
3.20
2.08
2.06
1.03
1.00
3.15
f1 (ppm)

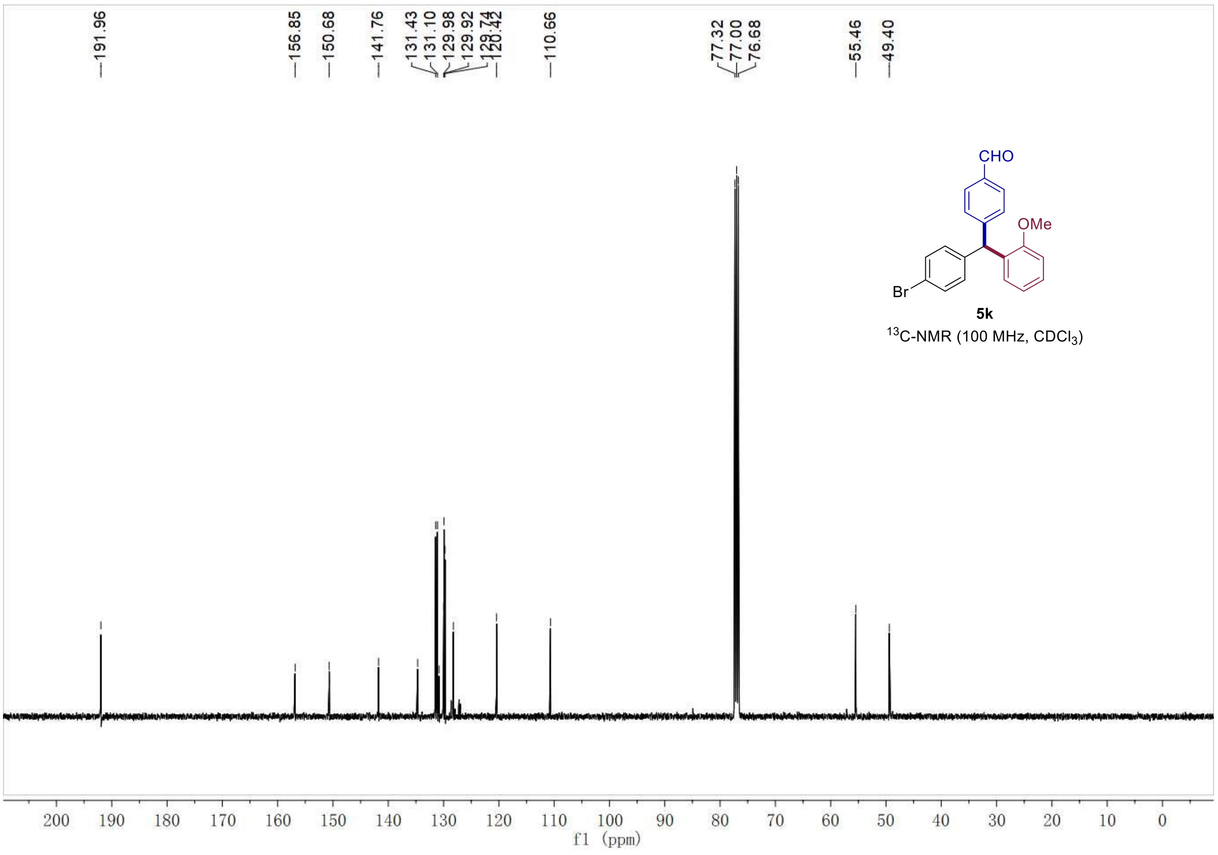
191.96
156.85
150.68
141.76
131.43
131.10
129.98
129.92
110.66
77.32
77.00
76.68
55.46
49.40
CHO
OMe
Br
5k
^{13}C-NMR (100 MHz, $CDCl_3$)
f1 (ppm)

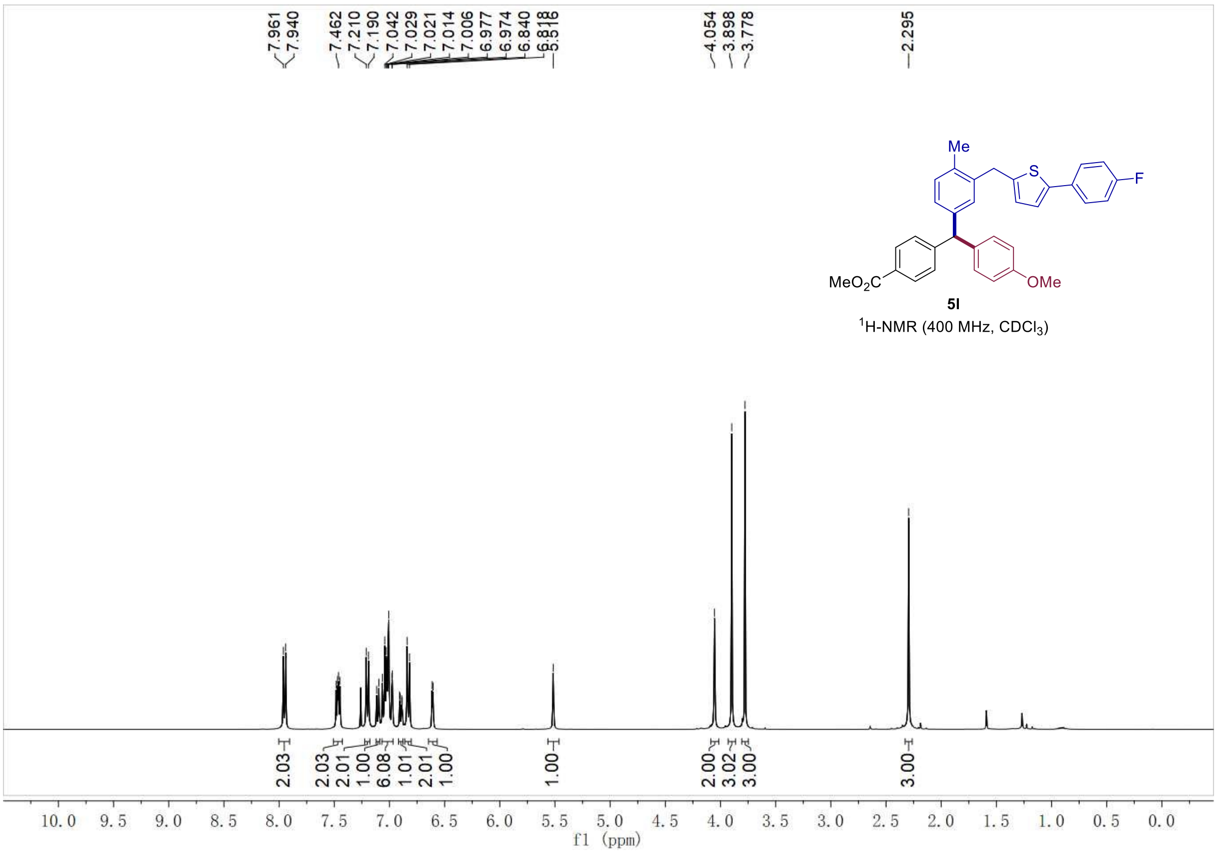
7.961
7.940
7.462
7.210
7.190
7.042
7.029
7.021
7.014
7.006
6.977
6.974
6.840
4.054
3.898
3.778
2.295
Me
S
F
MeO2C
OMe
5l
1H-NMR (400 MHz, CDCl3)
2.03
2.03
2.01
1.00
6.08
1.01
2.01
1.00
1.00
2.00
3.02
3.00
3.00
f1 (ppm)

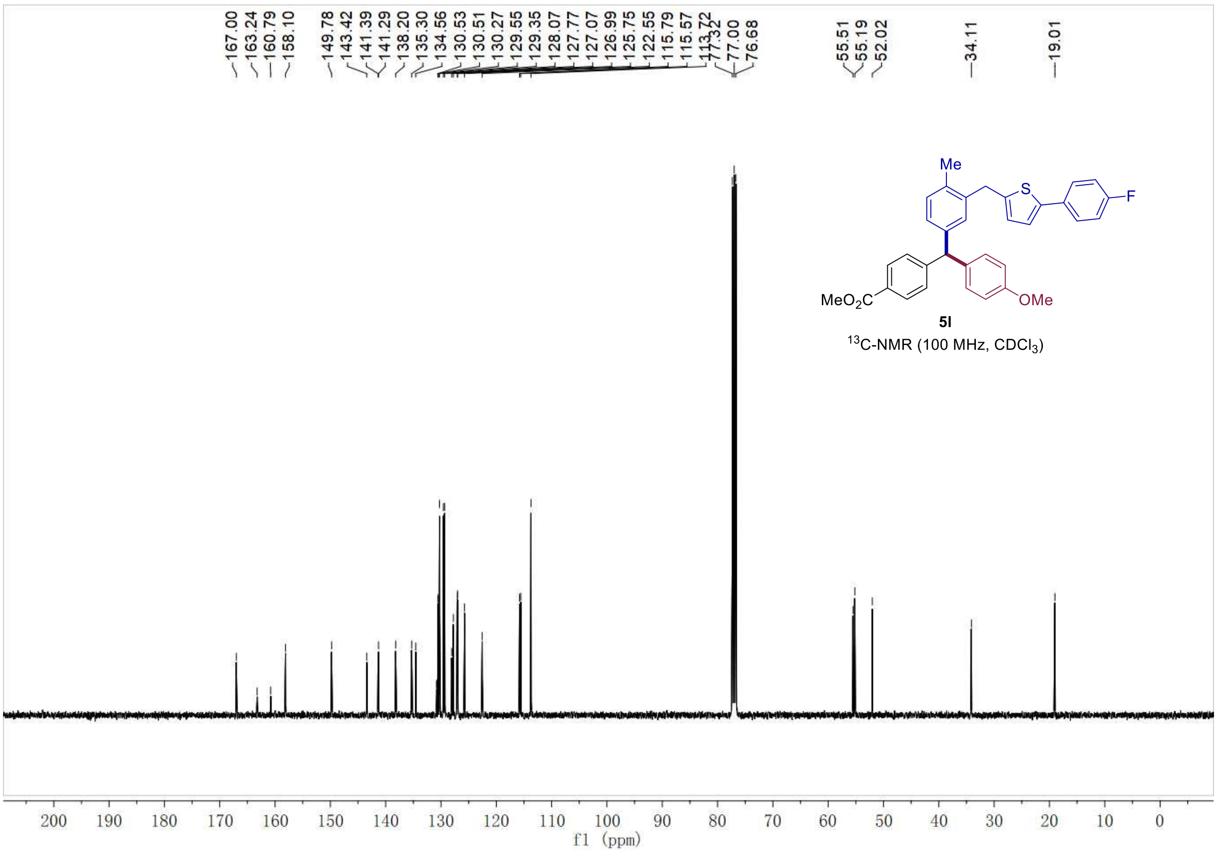
167.00
163.24
160.79
158.10
149.78
143.42
141.39
141.29
138.20
135.30
134.56
130.53
130.51
130.27
129.55
129.35
128.07
127.77
127.07
126.99
125.75
122.55
115.79
115.57
77.00
76.68
55.51
55.19
52.02
34.11
19.01
Me
S
F
MeO2C
OMe
5l
13C-NMR (100 MHz, CDCl3)
f1 (ppm)

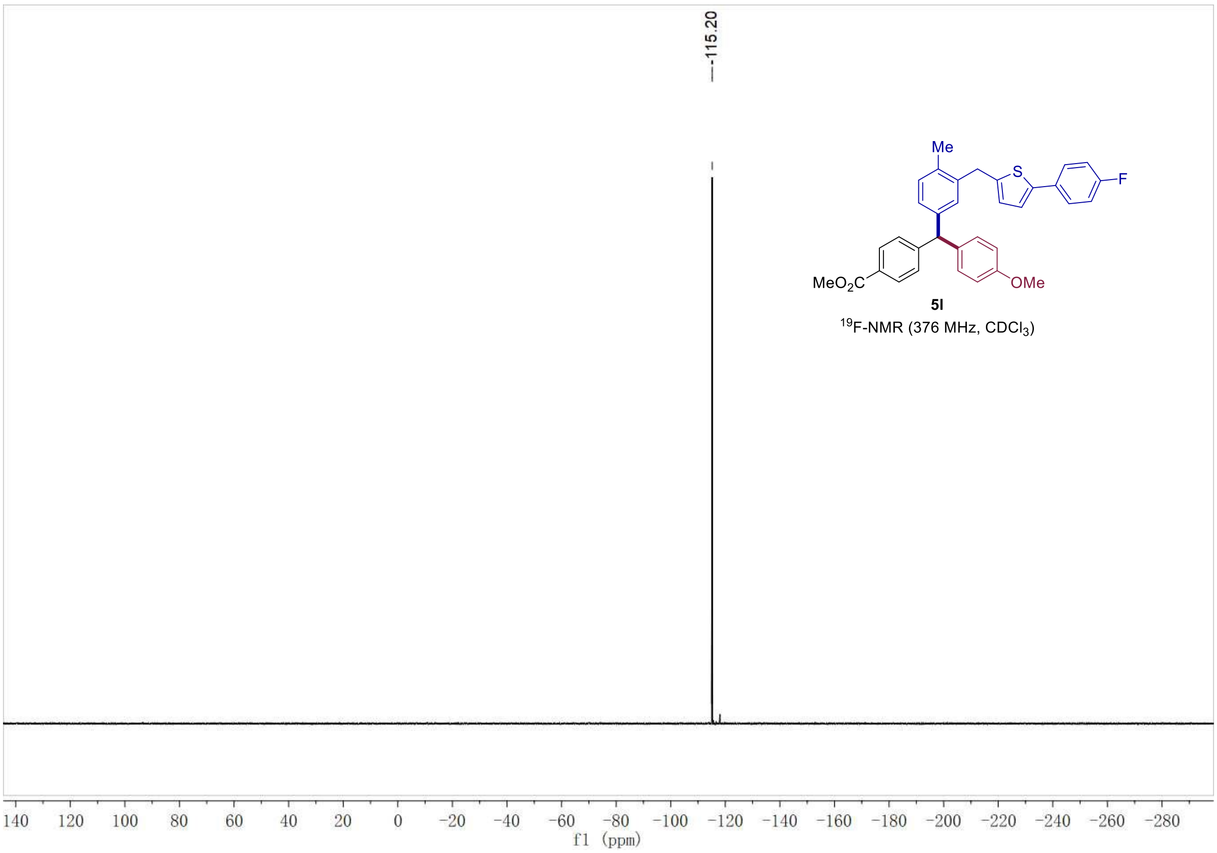

-115.20
Me
S
F
MeO2C
OMe
5l
19F-NMR (376 MHz, CDCl3)
140 120 100 80 60 40 20 0 -20 -40 -60 -80 -100 -120 -140 -160 -180 -200 -220 -240 -260 -280
f1 (ppm)

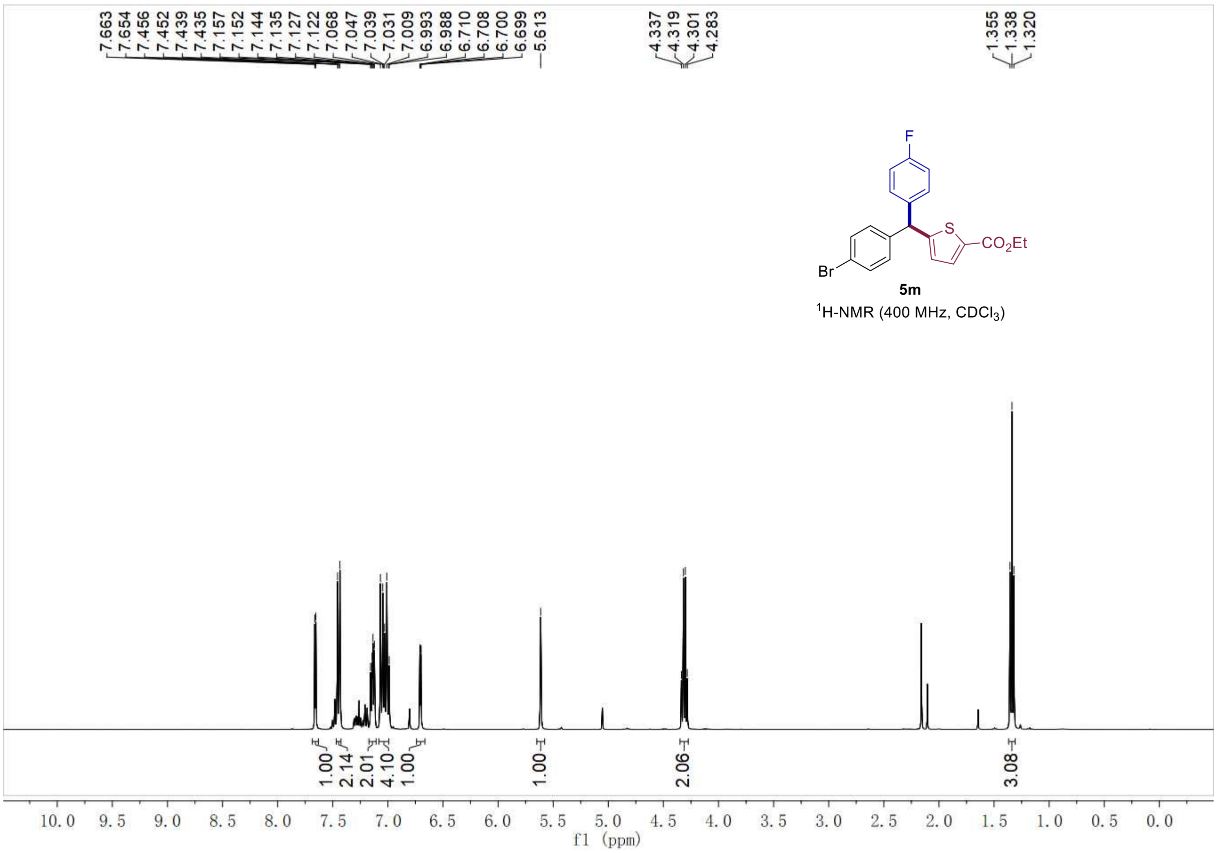

7.663 7.654 7.456 7.452 7.439 7.435 7.157 7.152 7.144 7.135 7.127 7.122 7.068 7.047 7.039 7.031 7.009 6.993 6.988 6.710 6.708 6.700 6.699 5.613
4.337 4.319 4.301 4.283
1.355 1.338 1.320
F
S
CO2Et
Br
5m
1H-NMR (400 MHz, CDCl3)
1.00 2.14 2.01 4.10 1.00
1.00
2.06
3.08
10.0 9.5 9.0 8.5 8.0 7.5 7.0 6.5 6.0 5.5 5.0 4.5 4.0 3.5 3.0 2.5 2.0 1.5 1.0 0.5 0.0
f1 (ppm)

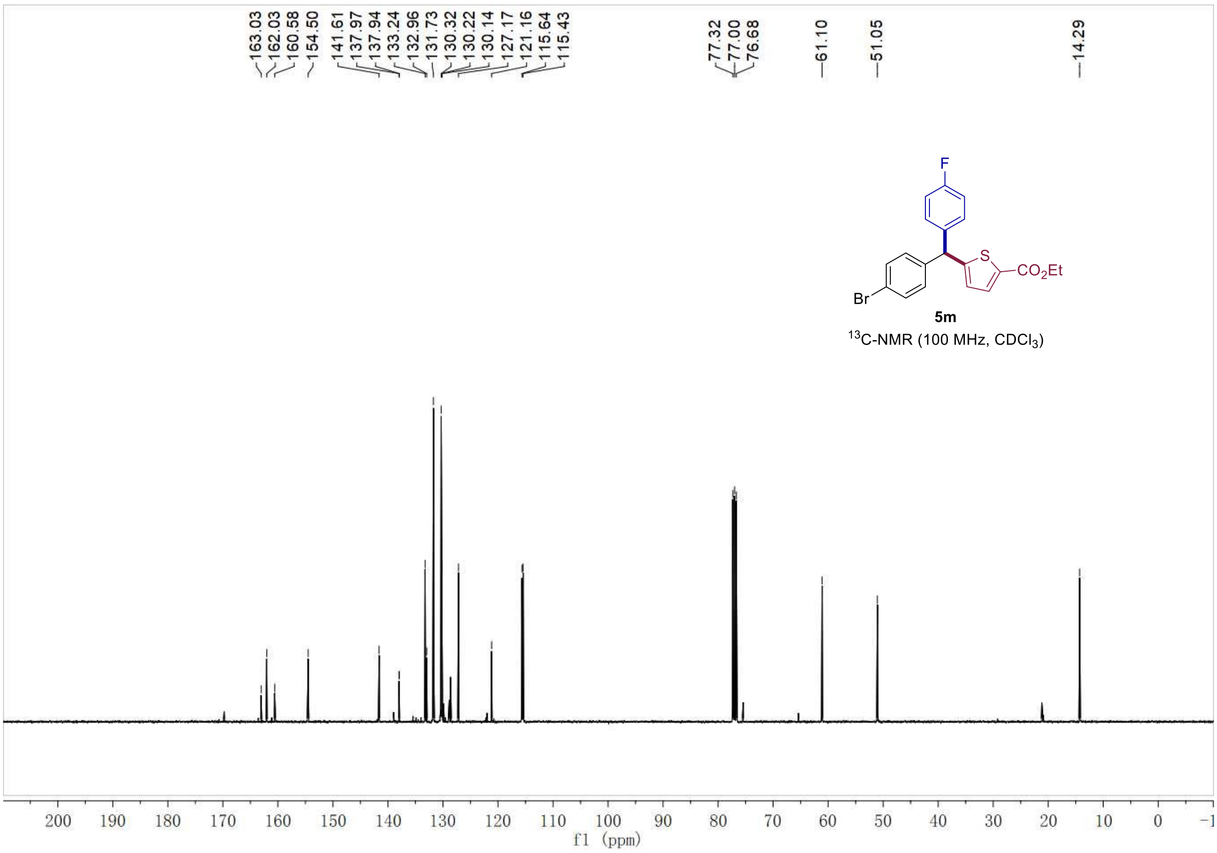
163.03
162.03
160.58
154.50
141.61
137.97
137.94
133.24
132.96
131.73
130.32
130.22
130.14
127.17
121.16
115.64
115.43
77.32
77.00
76.68
61.10
51.05
14.29
F
S
CO2Et
Br
5m
13C-NMR (100 MHz, CDCl3)
f1 (ppm)

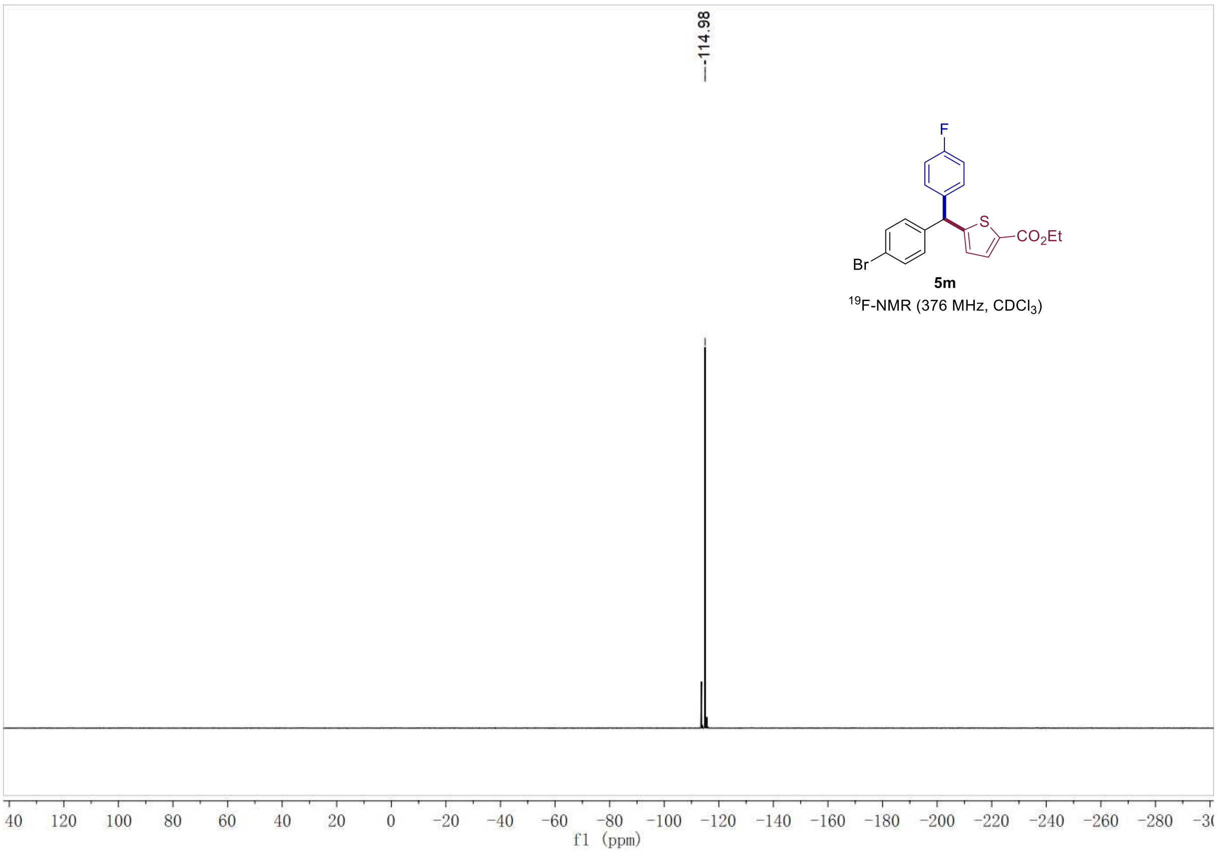
-114.98
F
S
CO2Et
Br
5m
19F-NMR (376 MHz, CDCl3)
f1 (ppm)

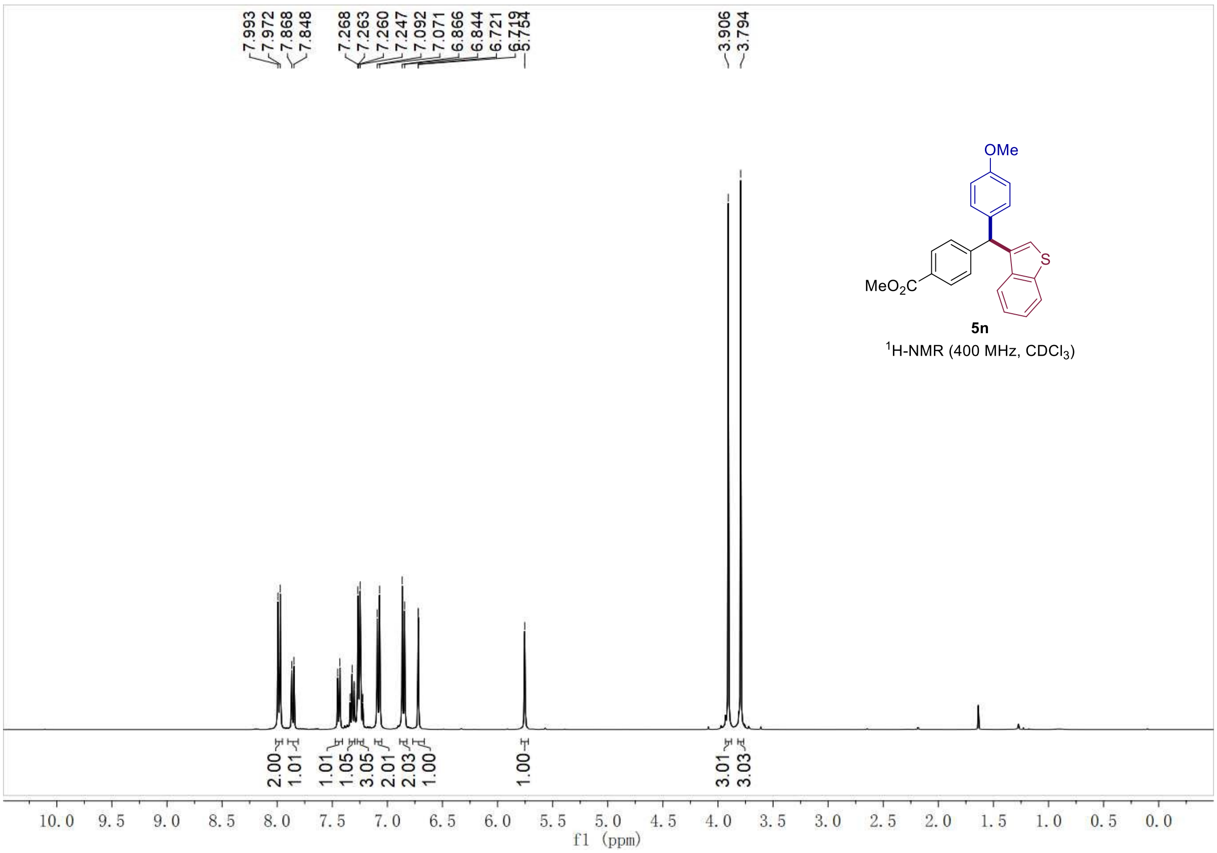
7.993
7.972
7.868
7.848
7.268
7.263
7.260
7.247
7.092
7.071
6.866
6.844
6.721
3.906
3.794
OMe
MeO2C
S
5n
1H-NMR (400 MHz, CDCl3)
2.00
1.01
1.01
1.05
3.05
2.01
2.03
1.00
1.00
3.01
3.03
f1 (ppm)

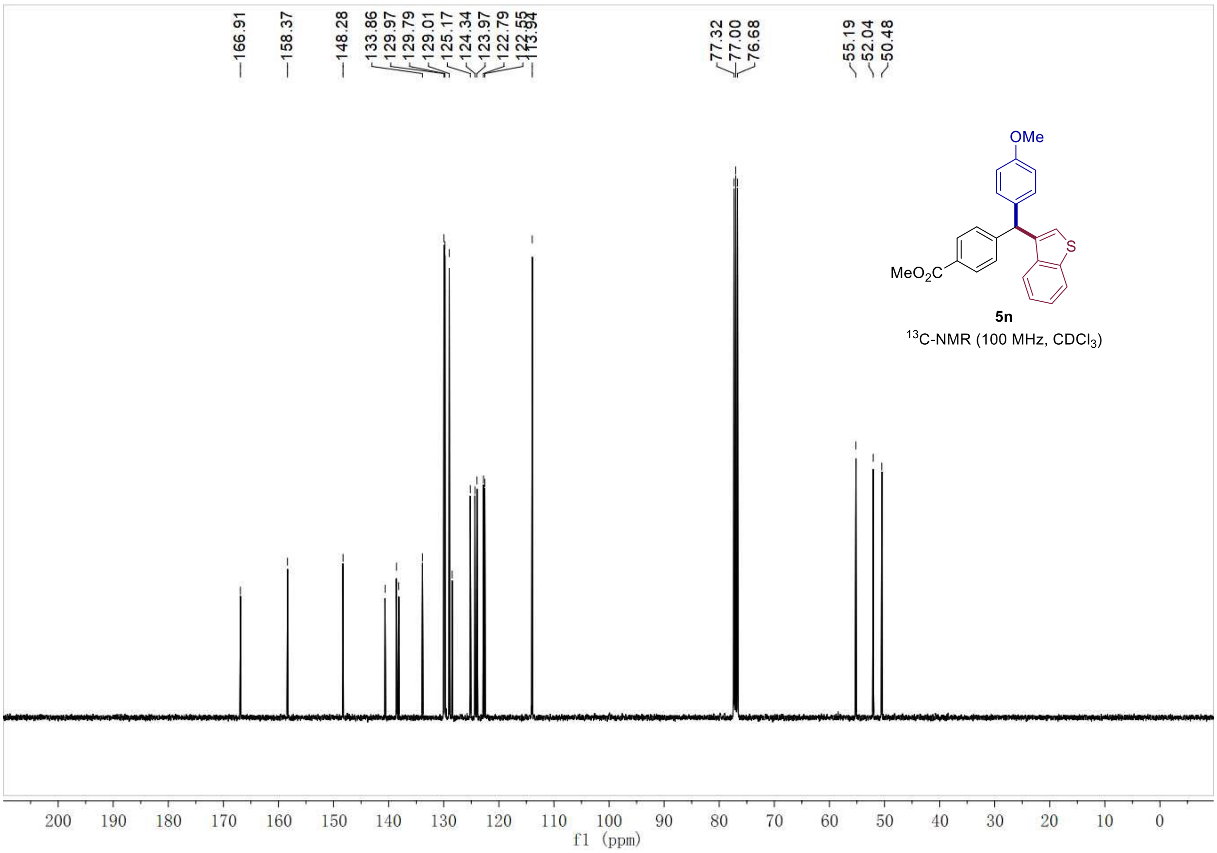
166.91
158.37
148.28
133.86
129.97
129.79
129.01
125.17
124.34
123.97
122.79
77.32
77.00
76.68
55.19
52.04
50.48
OMe
MeO2C
S
5n
13C-NMR (100 MHz, CDCl3)
f1 (ppm)

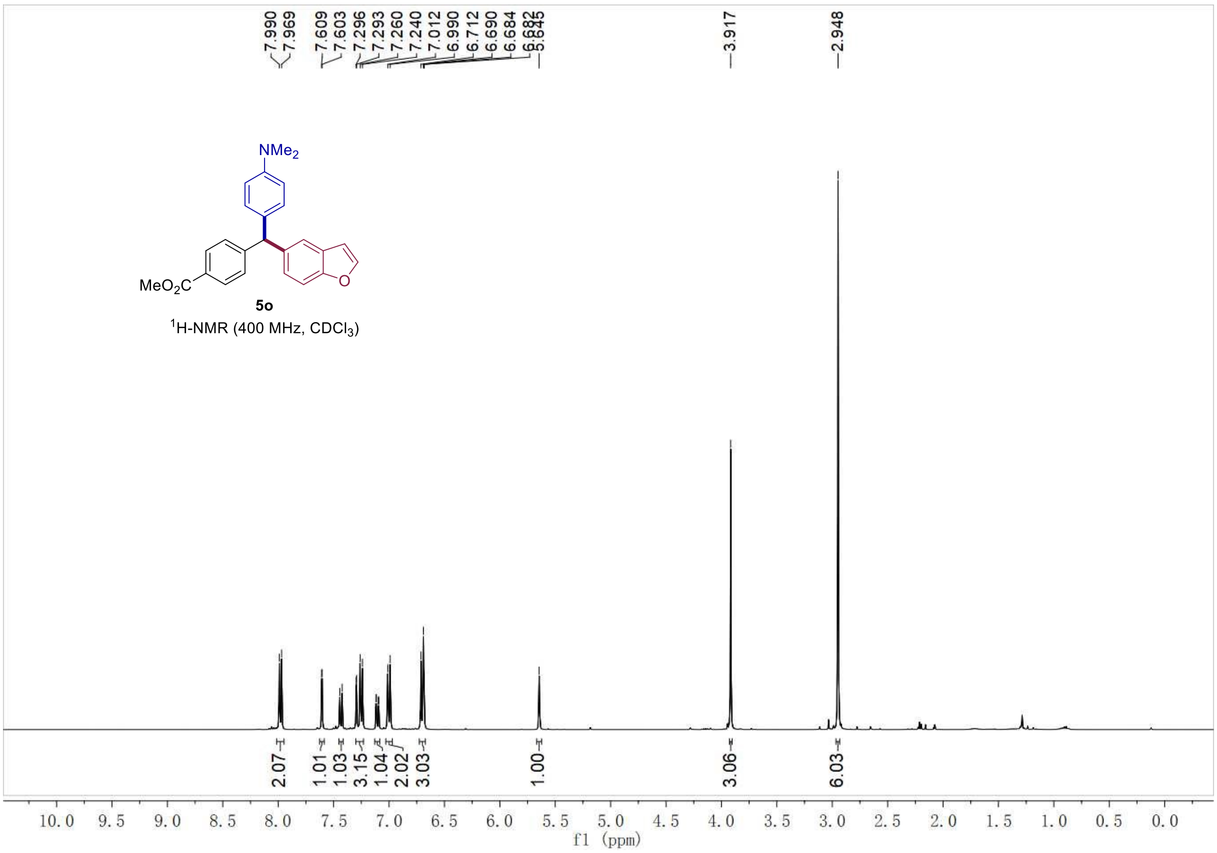
7.990
7.969
7.609
7.603
7.296
7.293
7.260
7.240
7.012
6.990
6.712
6.690
6.684
[illegible]
3.917
2.948
NMe2
MeO2C
O
5o
1H-NMR (400 MHz, CDCl3)
2.07
1.01
1.03
3.15
1.04
2.02
3.03
1.00
3.06
6.03
f1 (ppm)

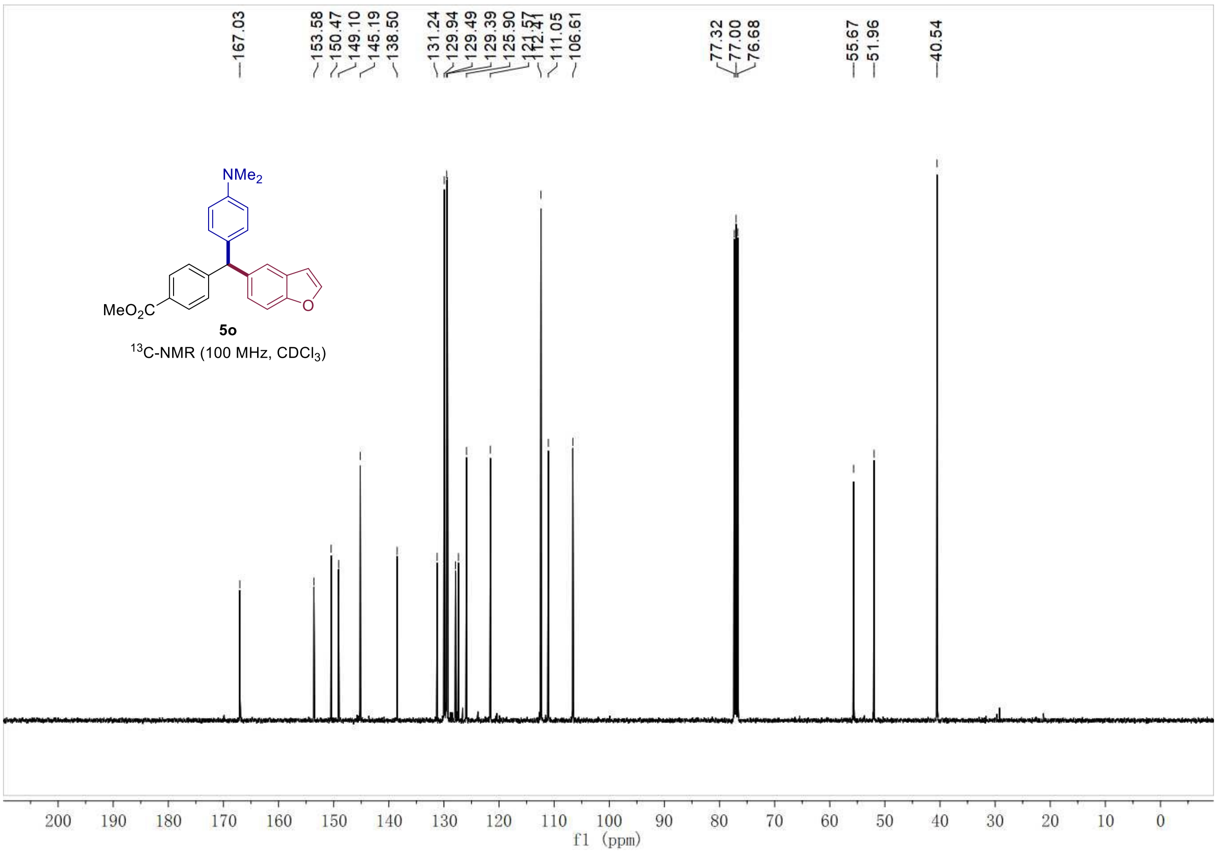
167.03
153.58
150.47
149.10
145.19
138.50
131.24
129.94
129.49
129.39
125.90
111.05
106.61
77.32
77.00
76.68
55.67
51.96
40.54
NMe2
MeO2C
O
5o
13C-NMR (100 MHz, CDCl3)
f1 (ppm)

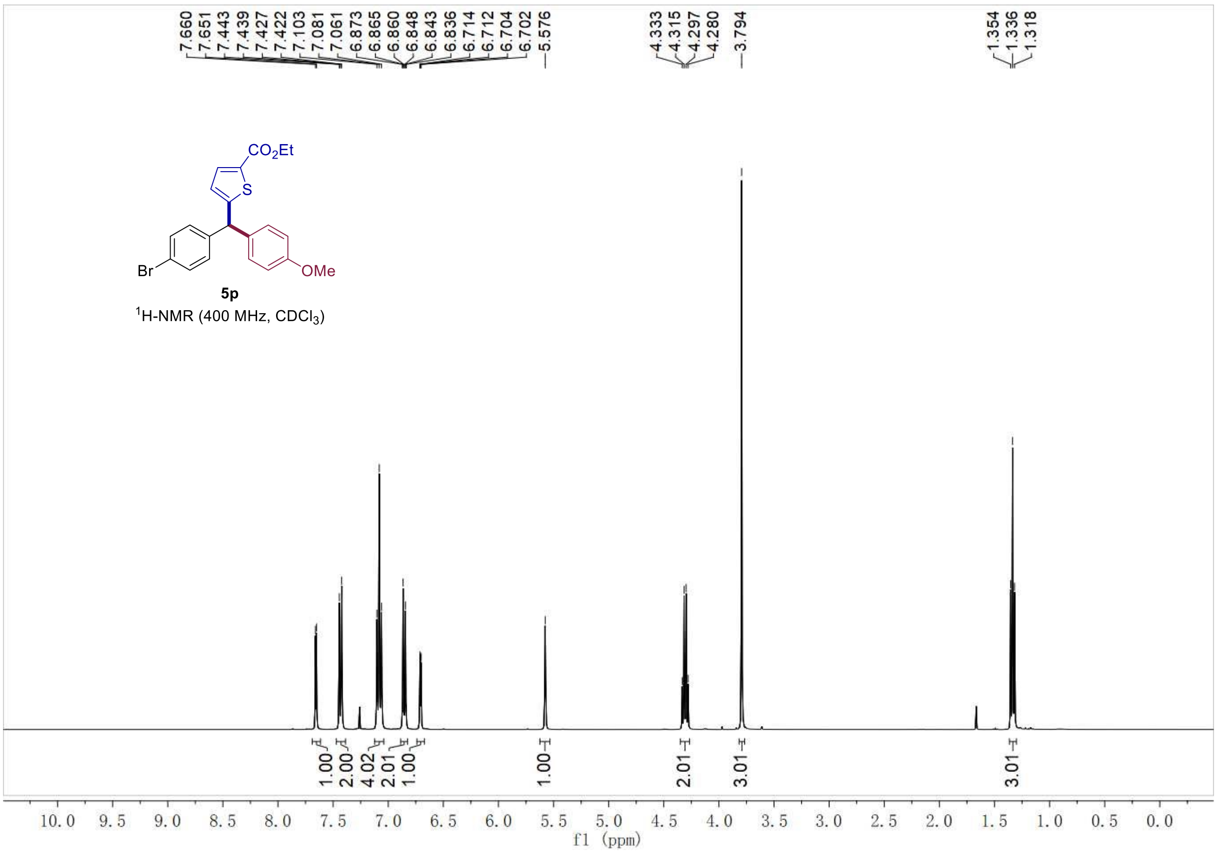
7.660
7.651
7.443
7.439
7.427
7.422
7.103
7.081
7.061
6.873
6.865
6.860
6.848
6.843
6.836
6.714
6.712
6.704
6.702
5.576
4.333
4.315
4.297
4.280
3.794
1.354
1.336
1.318
CO_2Et
S
Br
OMe
5p
^{1}H-NMR (400 MHz, $CDCl_3$)
1.00
2.00
4.02
2.01
1.00
1.00
2.01
3.01
3.01
10.0 9.5 9.0 8.5 8.0 7.5 7.0 6.5 6.0 5.5 5.0 4.5 4.0 3.5 3.0 2.5 2.0 1.5 1.0 0.5 0.0
f1 (ppm)

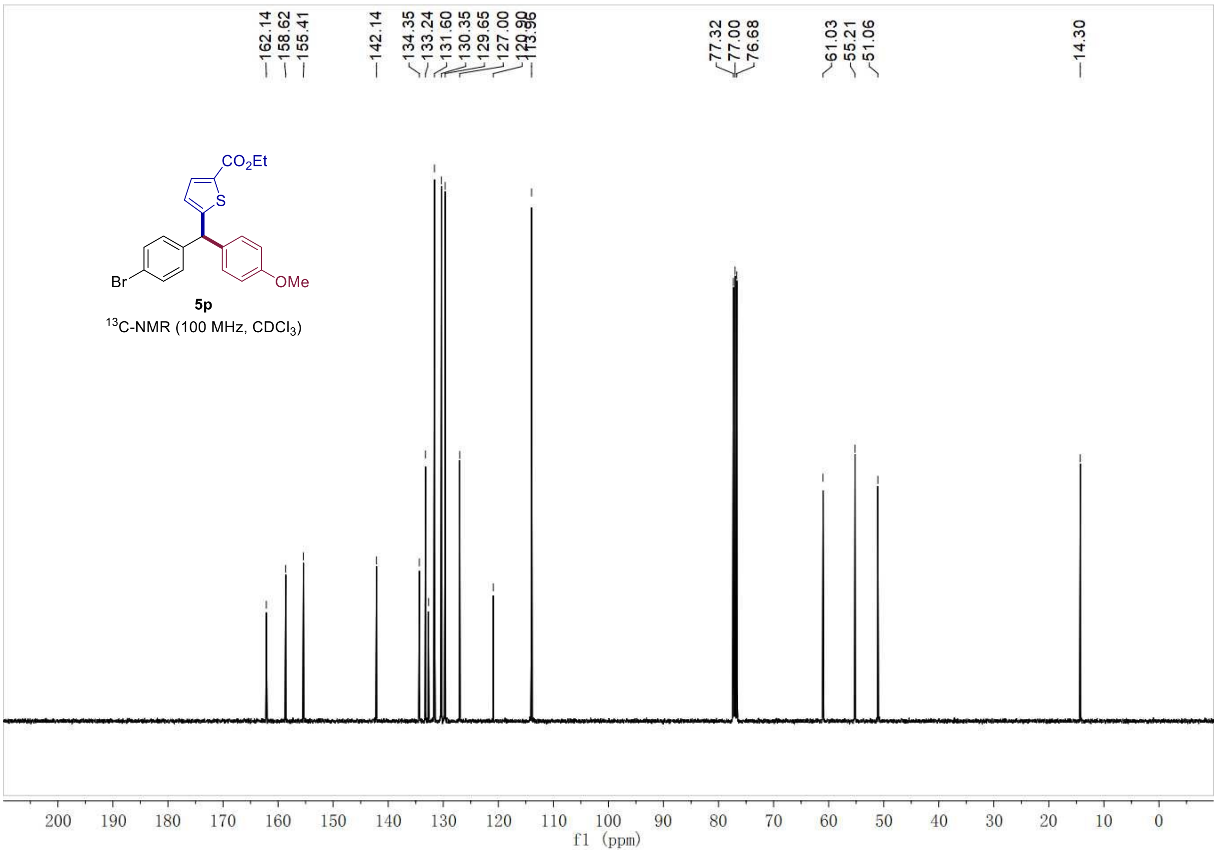
162.14
158.62
155.41
142.14
134.35
133.24
131.60
130.35
129.65
127.00
120.90
113.96
77.32
77.00
76.68
61.03
55.21
51.06
14.30
CO_2Et
S
Br
OMe
5p
^{13}C-NMR (100 MHz, $CDCl_3$)
200 190 180 170 160 150 140 130 120 110 100 90 80 70 60 50 40 30 20 10 0
f1 (ppm)

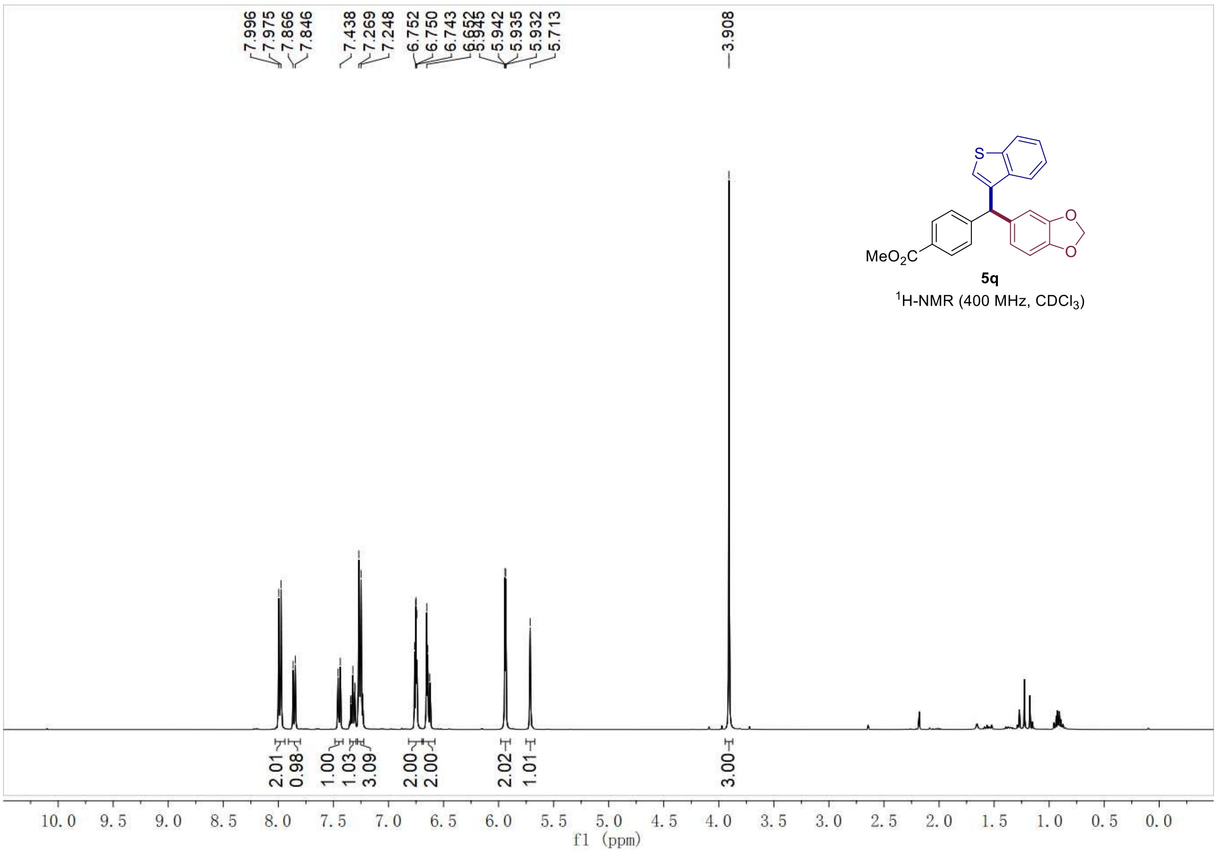
7.996
7.975
7.866
7.846
7.438
7.269
7.248
6.752
6.750
6.743
6.652
5.945
5.942
5.935
5.932
5.713
3.908
S
MeO2C
O
O
5q
1H-NMR (400 MHz, CDCl3)
2.01
0.98
1.00
1.03
3.09
2.00
2.00
2.02
1.01
3.00
10.0 9.5 9.0 8.5 8.0 7.5 7.0 6.5 6.0 5.5 5.0 4.5 4.0 3.5 3.0 2.5 2.0 1.5 1.0 0.5 0.0
f1 (ppm)

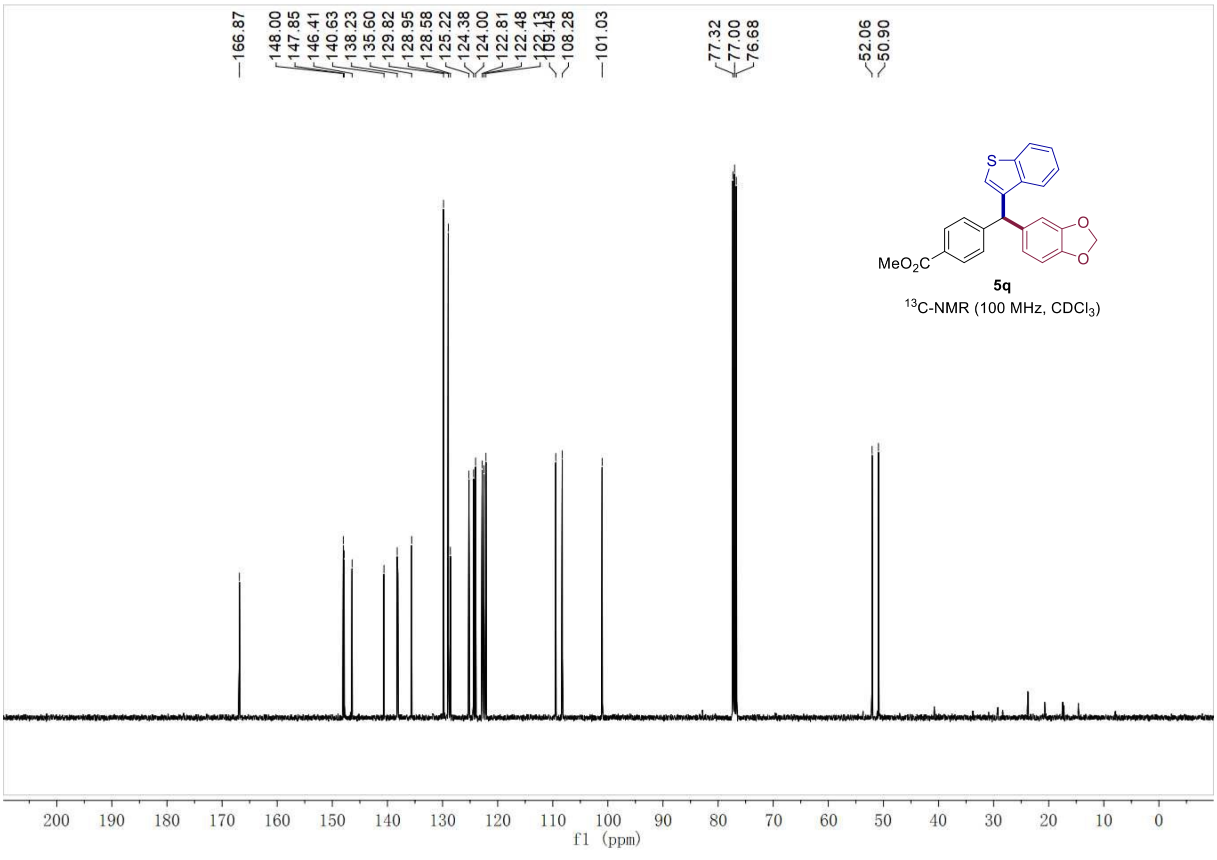
166.87
148.00
147.85
146.41
140.63
138.23
135.60
129.82
128.95
128.58
125.22
124.38
124.00
122.81
122.48
122.13
109.45
108.28
101.03
77.32
77.00
76.68
52.06
50.90
S
MeO2C
O
O
5q
13C-NMR (100 MHz, CDCl3)
200 190 180 170 160 150 140 130 120 110 100 90 80 70 60 50 40 30 20 10 0
f1 (ppm)

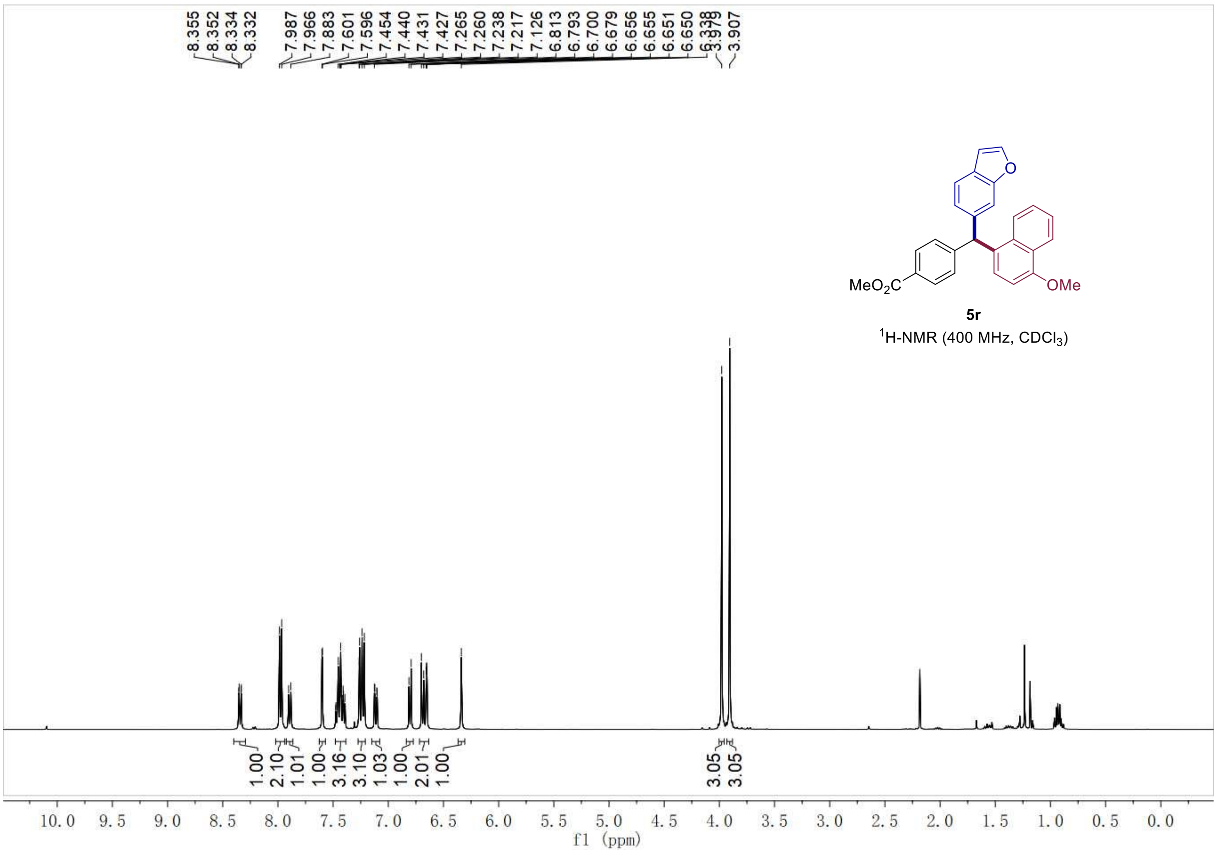
8.355
8.352
8.334
8.332
7.987
7.966
7.883
7.601
7.596
7.454
7.440
7.431
7.427
7.265
7.260
7.238
7.217
7.126
6.813
6.793
6.700
6.679
6.656
6.655
6.651
6.650
3.907
MeO2C
OMe
5r
1H-NMR (400 MHz, CDCl3)
1.00
2.10
1.01
1.00
3.16
3.10
1.03
1.00
2.01
1.00
3.05
3.05
f1 (ppm)

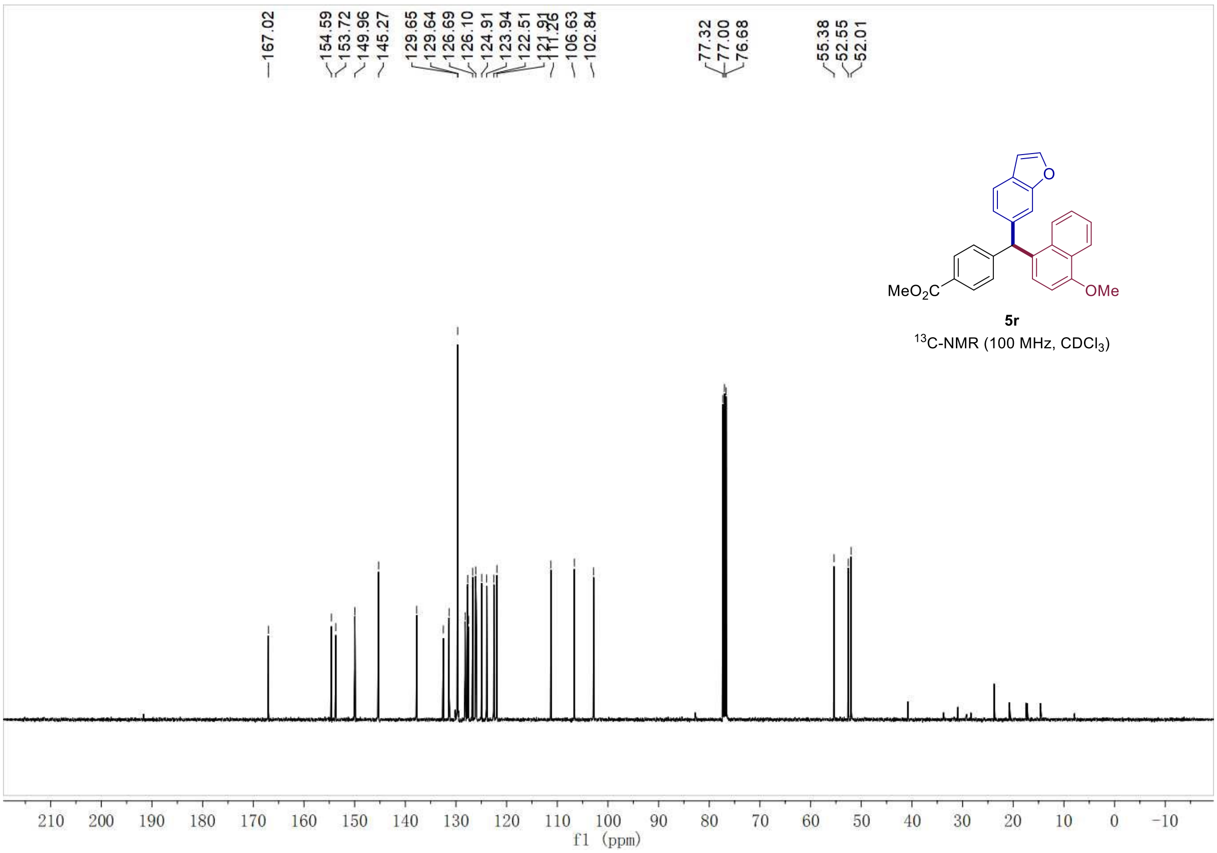
167.02
154.59
153.72
149.96
145.27
129.65
129.64
126.69
126.10
124.91
123.94
122.51
106.63
102.84
77.32
77.00
76.68
55.38
52.55
52.01
MeO2C
OMe
5r
13C-NMR (100 MHz, CDCl3)
f1 (ppm)

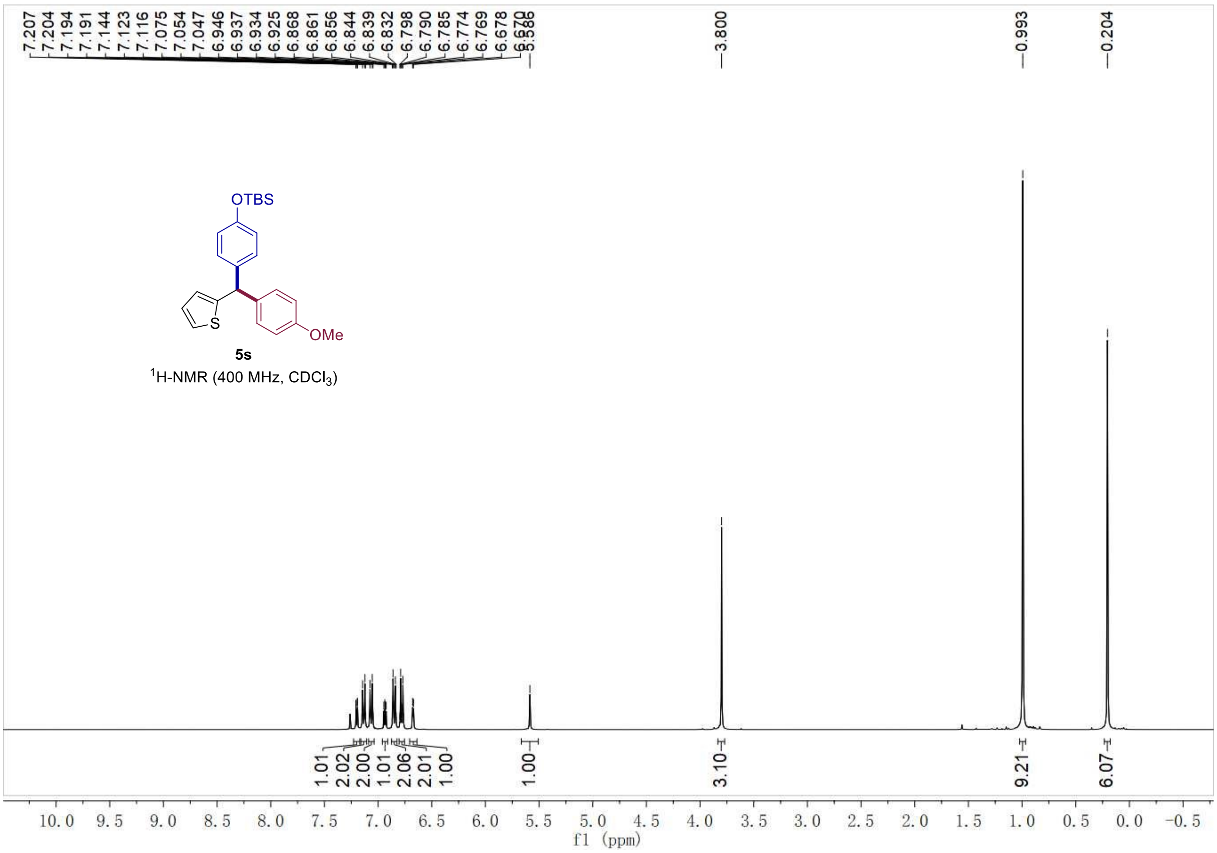
7.207
7.204
7.194
7.191
7.144
7.123
7.116
7.075
7.054
7.047
6.946
6.937
6.934
6.925
6.868
6.861
6.856
6.844
6.839
6.832
6.798
6.790
6.785
6.774
6.769
6.678
6.670
5.586
3.800
0.993
0.204
OTBS
S
OMe
5s
^{1}H-NMR (400 MHz, $CDCl_3$)
1.01
2.02
2.00
1.01
2.06
2.01
1.00
1.00
3.10
9.21
6.07
10.0 9.5 9.0 8.5 8.0 7.5 7.0 6.5 6.0 5.5 5.0 4.5 4.0 3.5 3.0 2.5 2.0 1.5 1.0 0.5 0.0 -0.5
f1 (ppm)

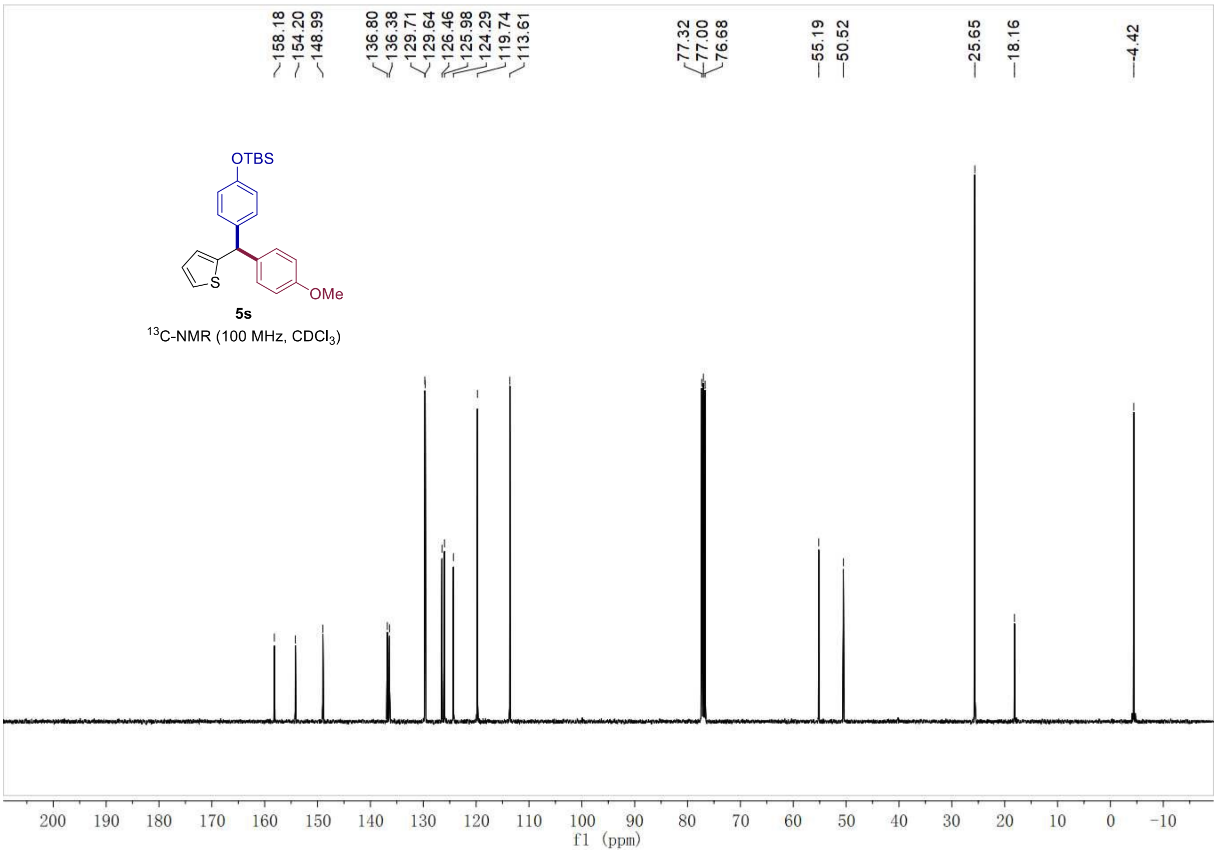
158.18
154.20
148.99
136.80
136.38
129.71
129.64
126.46
125.98
124.29
119.74
113.61
77.32
77.00
76.68
55.19
50.52
25.65
18.16
-4.42
OTBS
S
OMe
5s
^{13}C-NMR (100 MHz, $CDCl_3$)
200 190 180 170 160 150 140 130 120 110 100 90 80 70 60 50 40 30 20 10 0 -10
f1 (ppm)

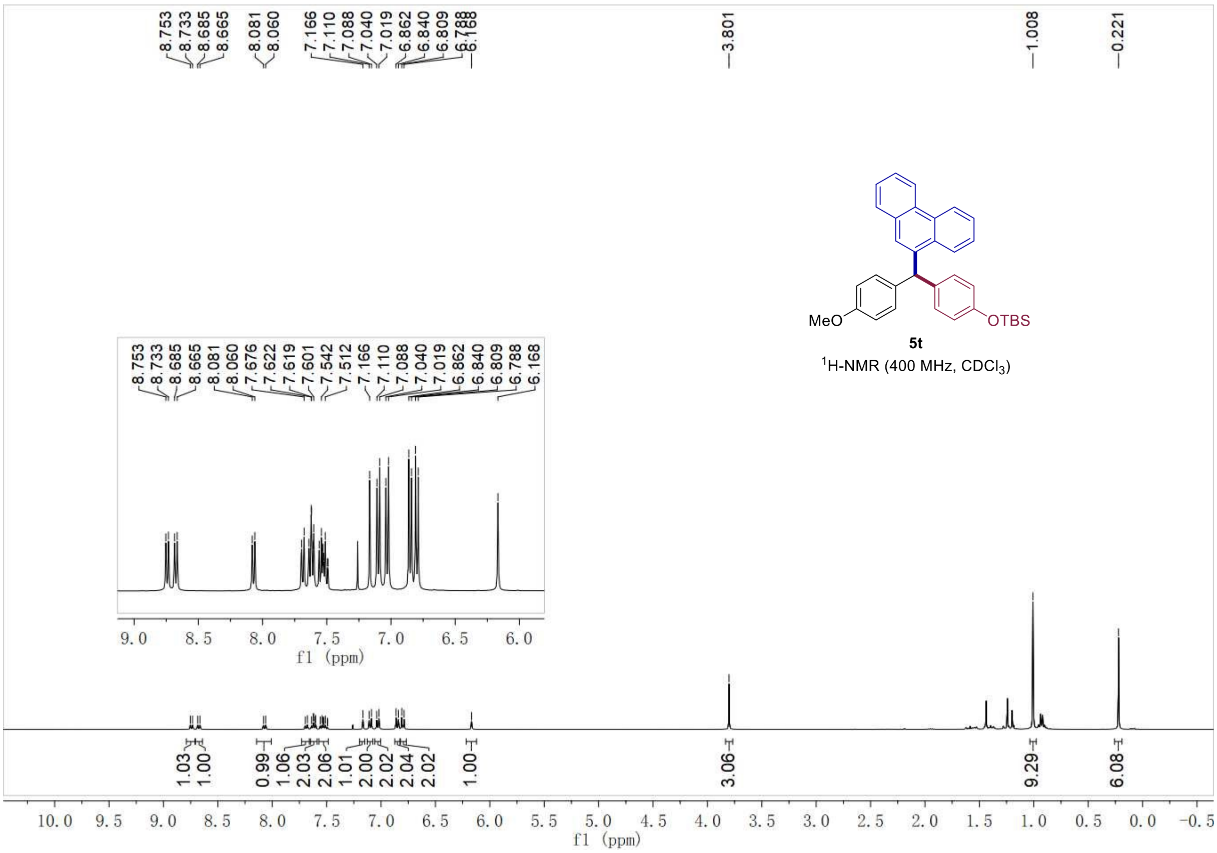
8.753
8.733
8.685
8.665
8.081
8.060
7.166
7.110
7.088
7.040
7.019
6.862
6.840
6.809
3.801
1.008
0.221
MeO
OTBS
5t
^{1}H-NMR (400 MHz, $CDCl_3$)
1.03
1.00
0.99
1.06
2.03
2.06
1.01
2.00
2.02
2.04
2.02
1.00
3.06
9.29
6.08
f1 (ppm)

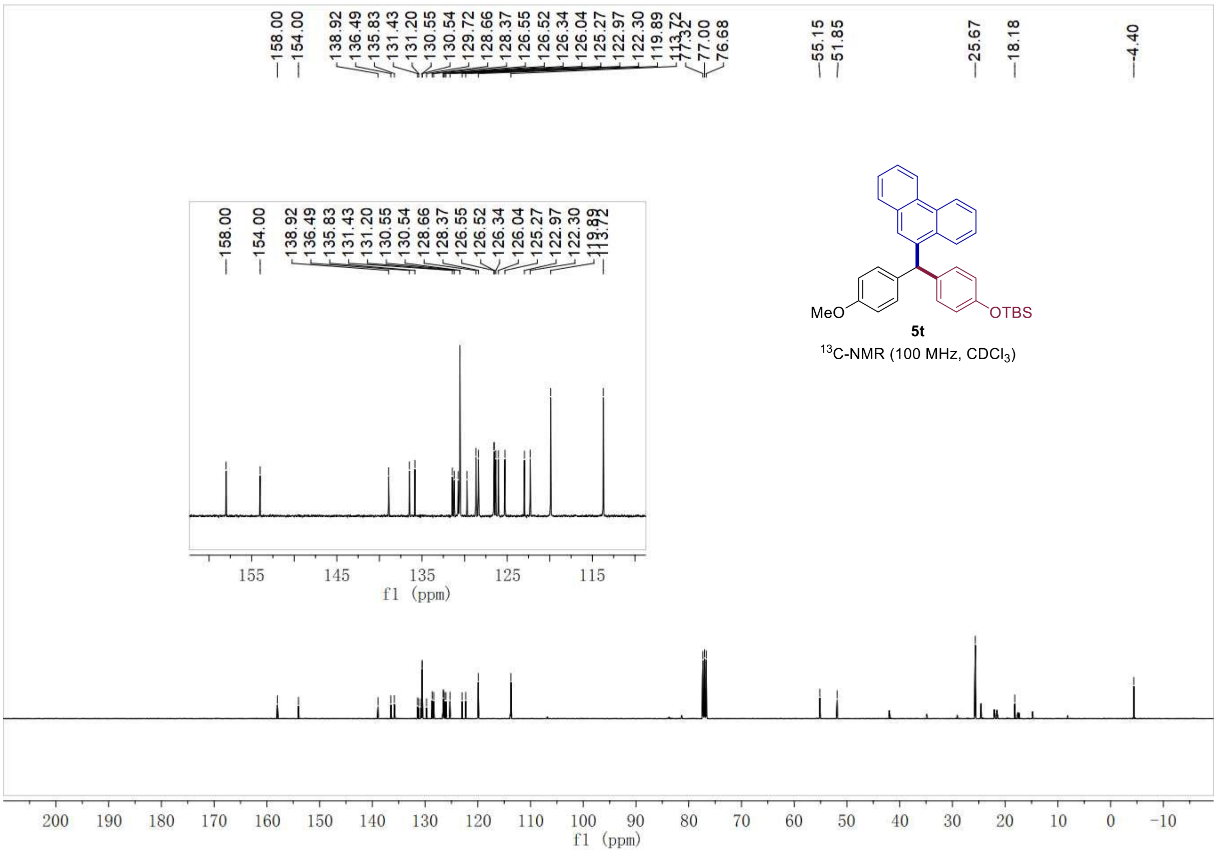
158.00
154.00
138.92
136.49
135.83
131.43
131.20
130.55
130.54
129.72
128.66
128.37
126.55
126.52
126.34
126.04
125.27
122.97
122.30
119.89
77.00
76.68
55.15
51.85
25.67
18.18
-4.40
MeO
OTBS
5t
^{13}C-NMR (100 MHz, $CDCl_3$)
f1 (ppm)

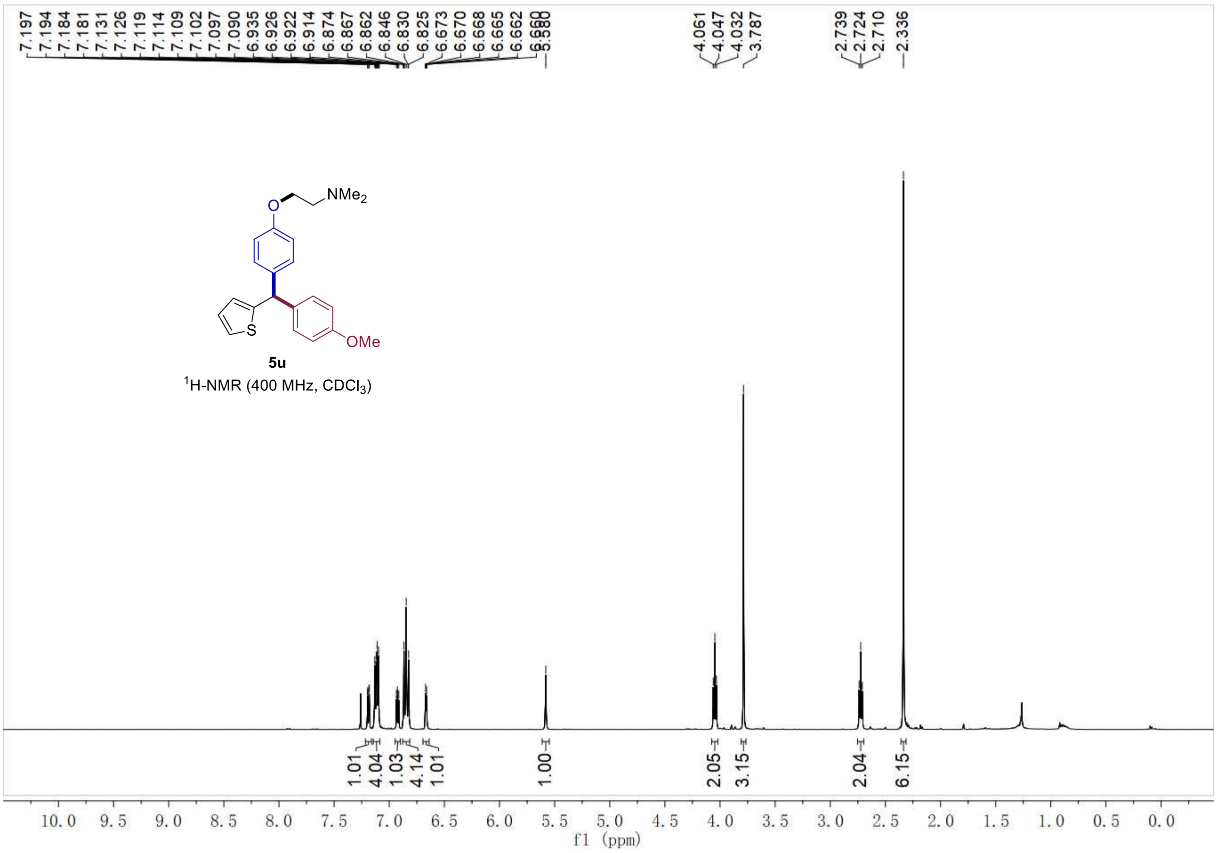
7.197
7.194
7.184
7.181
7.131
7.126
7.119
7.114
7.109
7.102
7.097
7.090
6.935
6.926
6.922
6.914
6.874
6.867
6.862
6.846
6.830
6.825
6.673
6.670
6.668
6.665
6.662
6.660
5.580
4.061
4.047
4.032
3.787
2.739
2.724
2.710
2.336
NMe2
O
S
OMe
5u
1H-NMR (400 MHz, CDCl3)
1.01
4.04
1.03
4.14
1.01
1.00
2.05
3.15
2.04
6.15
10.0 9.5 9.0 8.5 8.0 7.5 7.0 6.5 6.0 5.5 5.0 4.5 4.0 3.5 3.0 2.5 2.0 1.5 1.0 0.5 0.0
f1 (ppm)

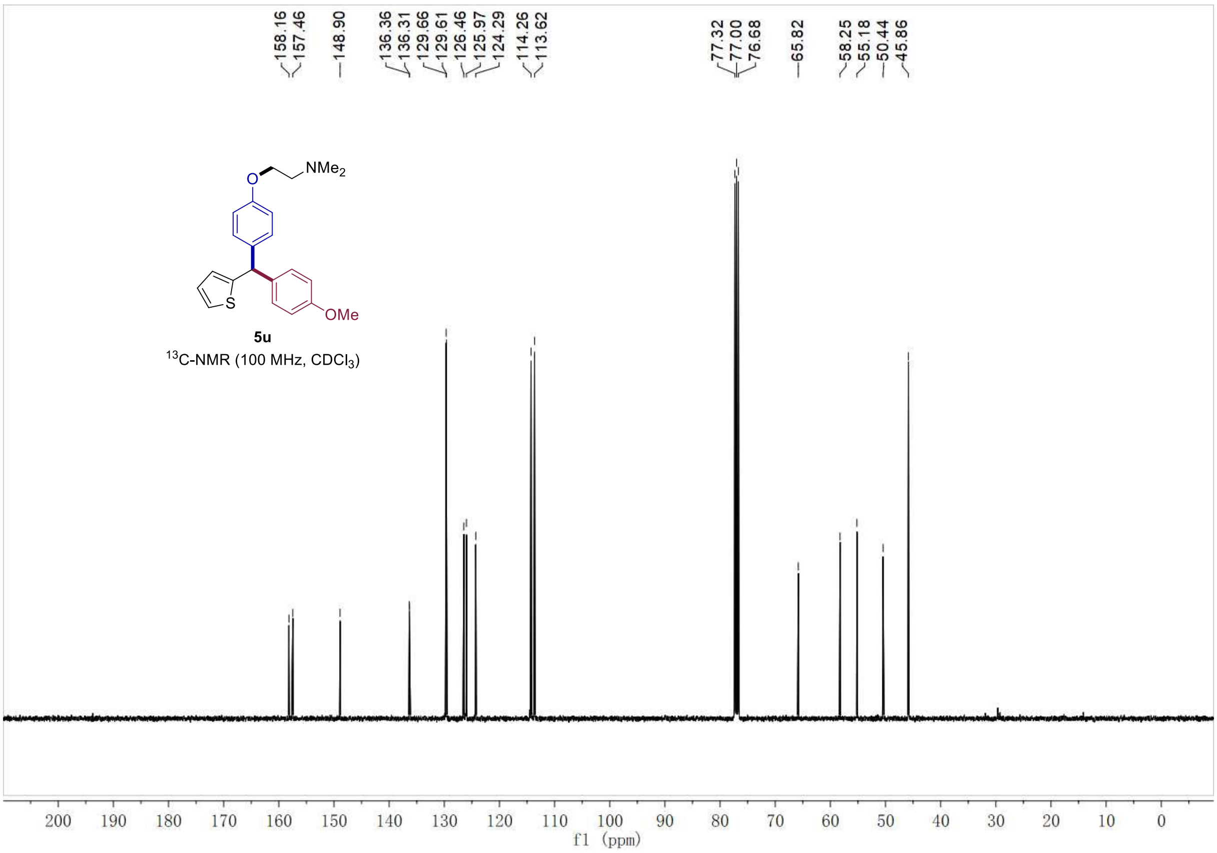
158.16
157.46
148.90
136.36
136.31
129.66
129.61
126.46
125.97
124.29
114.26
113.62
77.32
77.00
76.68
65.82
58.25
55.18
50.44
45.86
NMe2
O
S
OMe
5u
13C-NMR (100 MHz, CDCl3)
200 190 180 170 160 150 140 130 120 110 100 90 80 70 60 50 40 30 20 10 0
f1 (ppm)

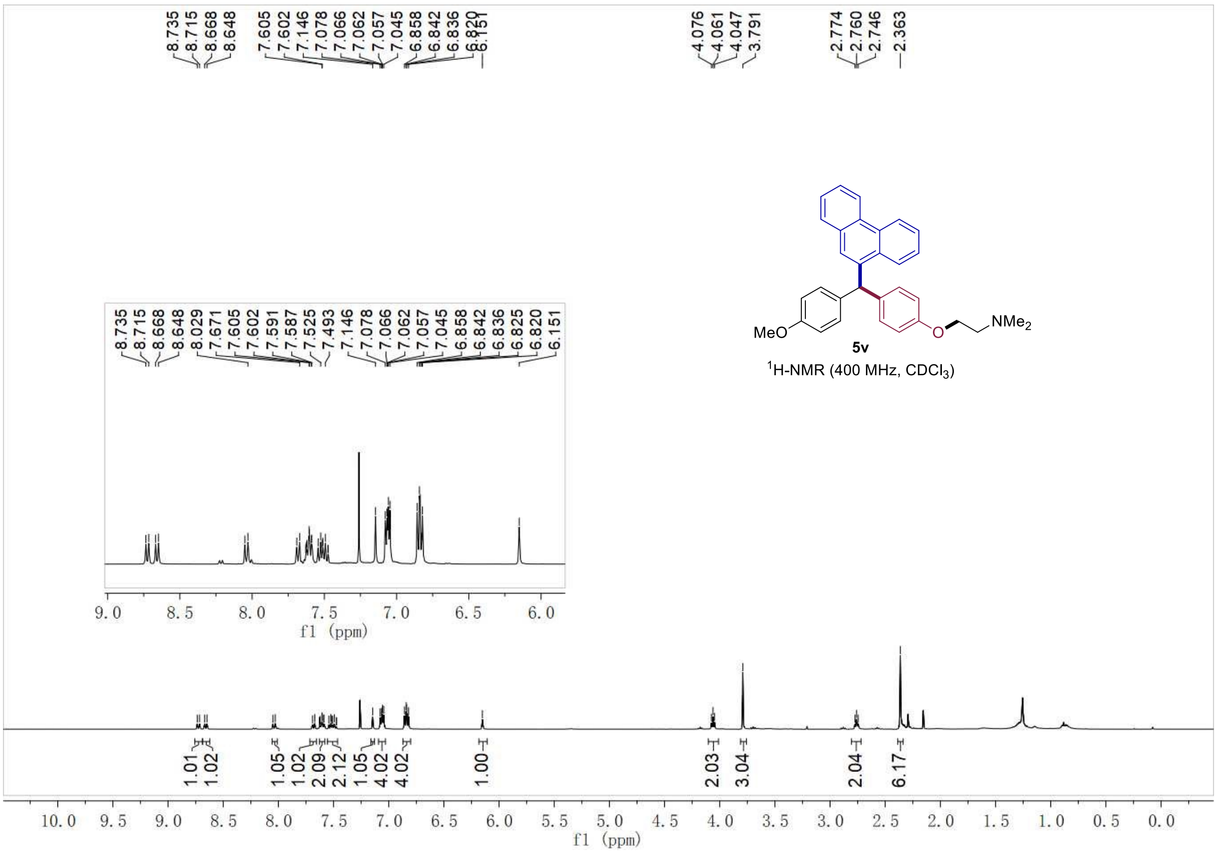

MeO
NMe2
5v
1H-NMR (400 MHz, CDCl3)
f1 (ppm)

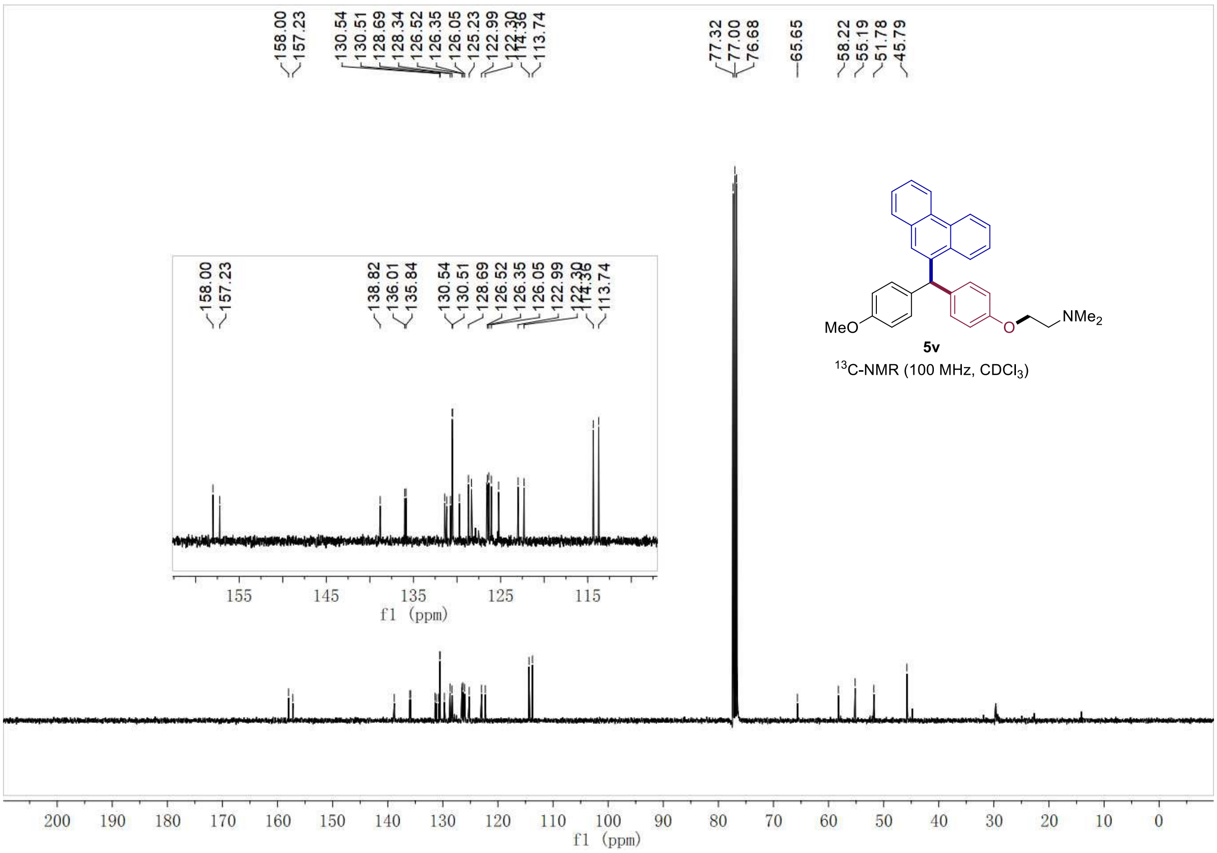

MeO
NMe2
5v
13C-NMR (100 MHz, CDCl3)
f1 (ppm)

NMR Spectra of **6a-6q**

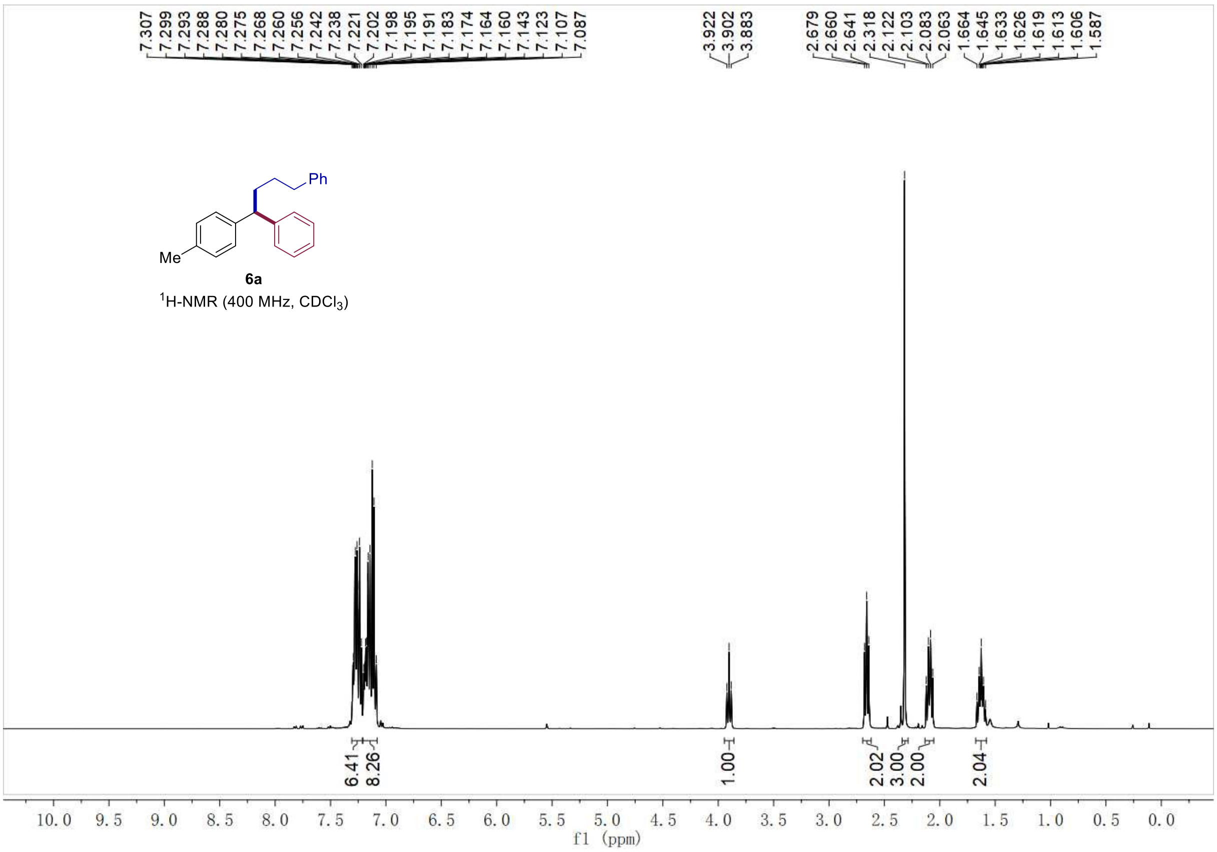

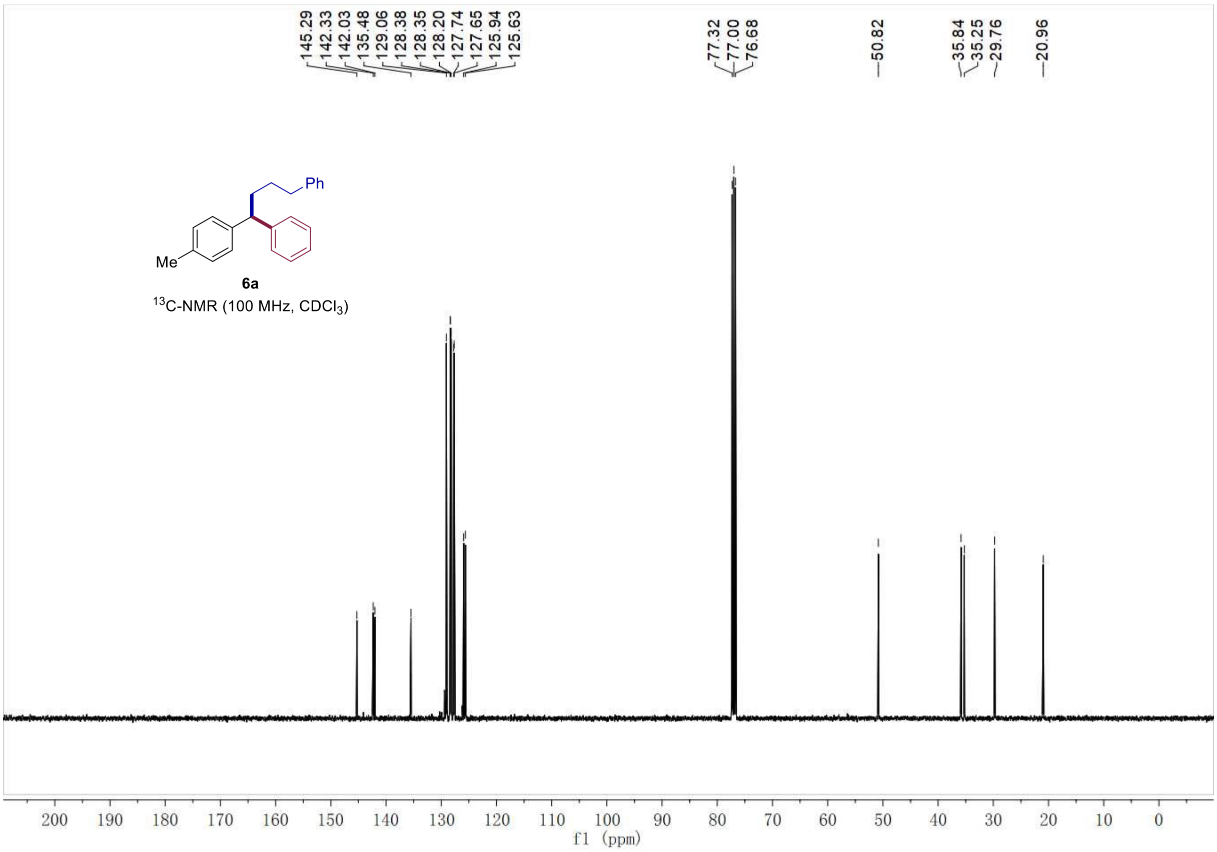

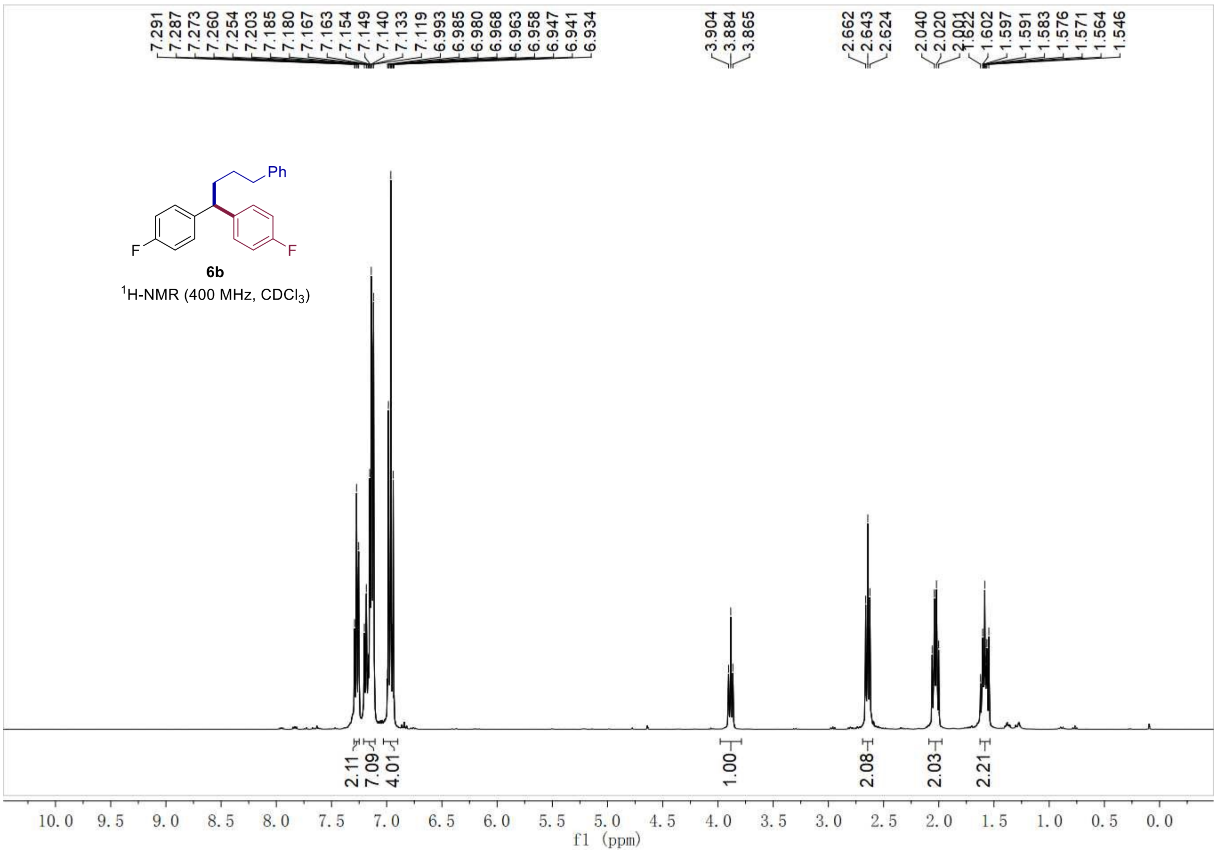
7.291 7.287 7.273 7.260 7.254 7.203 7.185 7.180 7.167 7.163 7.154 7.149 7.140 7.133 7.119 6.993 6.985 6.980 6.968 6.963 6.958 6.941 6.934
3.904 3.884 3.865
2.662 2.643 2.624
2.040 2.020 2.001 1.622 1.602 1.597 1.591 1.583 1.576 1.571 1.564 1.546
Ph
F
F
6b
1H-NMR (400 MHz, CDCl3)
2.11 7.09 4.01
1.00
2.08
2.03
2.21
10.0 9.5 9.0 8.5 8.0 7.5 7.0 6.5 6.0 5.5 5.0 4.5 4.0 3.5 3.0 2.5 2.0 1.5 1.0 0.5 0.0
f1 (ppm)

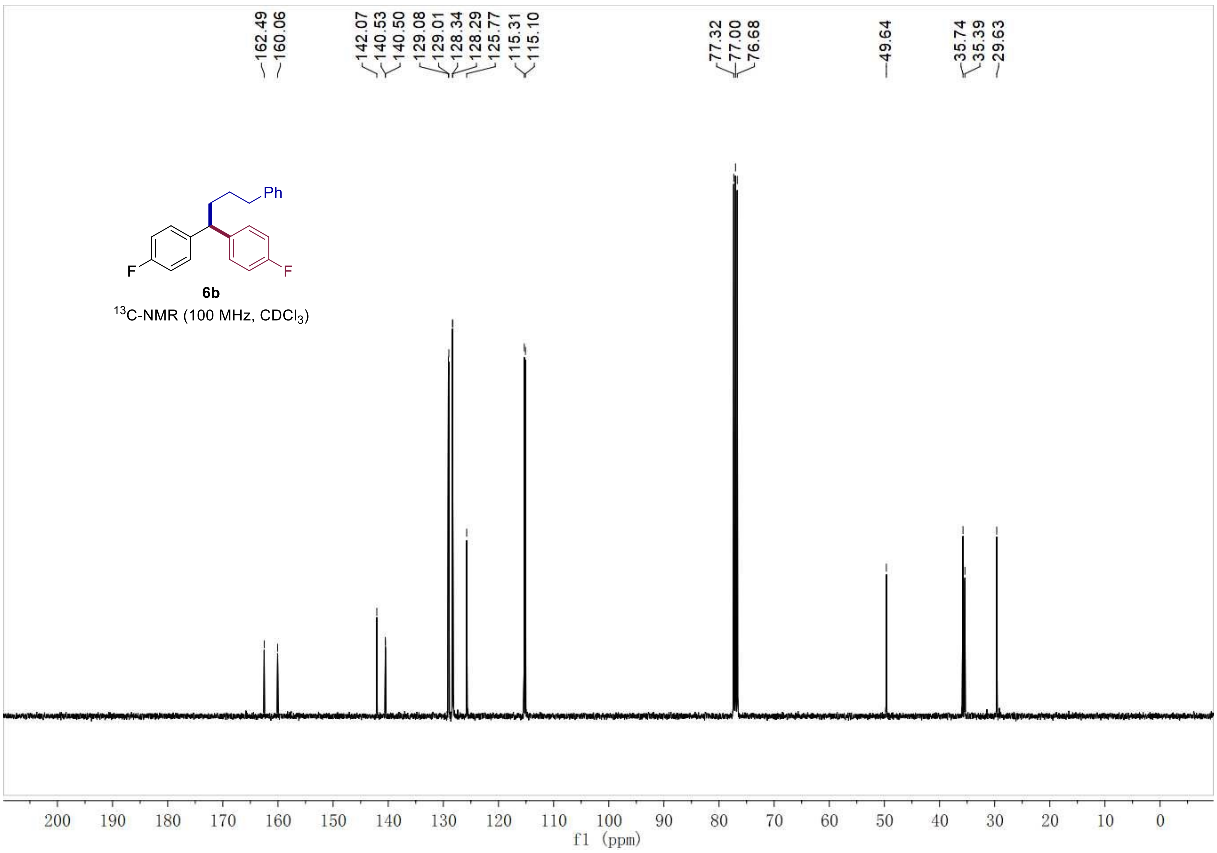
162.49 160.06
142.07 140.53 140.50 129.08 129.01 128.34 128.29 125.77 115.31 115.10
77.32 77.00 76.68
49.64
35.74 35.39 29.63
Ph
F
F
6b
13C-NMR (100 MHz, CDCl3)
200 190 180 170 160 150 140 130 120 110 100 90 80 70 60 50 40 30 20 10 0
f1 (ppm)

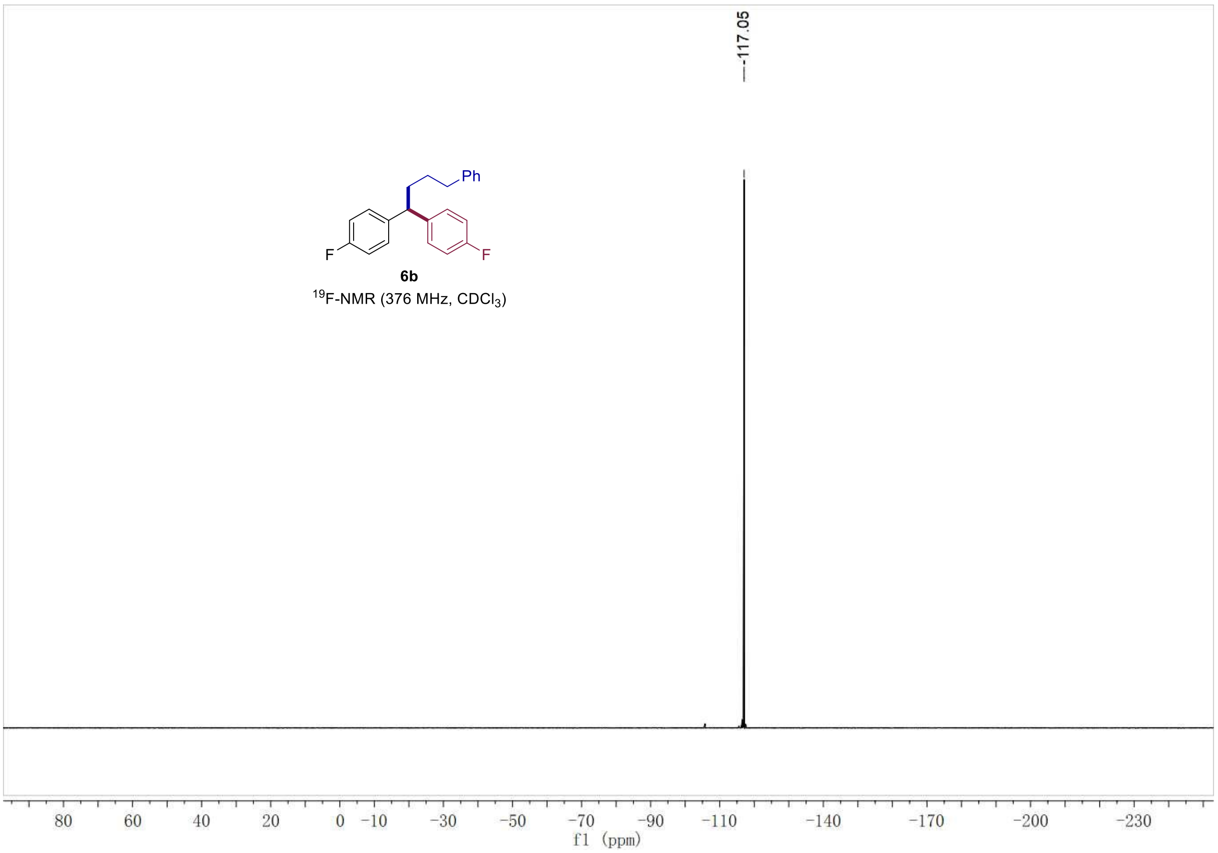
-117.05
Ph
F
F
6b
19F-NMR (376 MHz, CDCl3)
80 60 40 20 0 -10 -30 -50 -70 -90 -110 -140 -170 -200 -230
f1 (ppm)

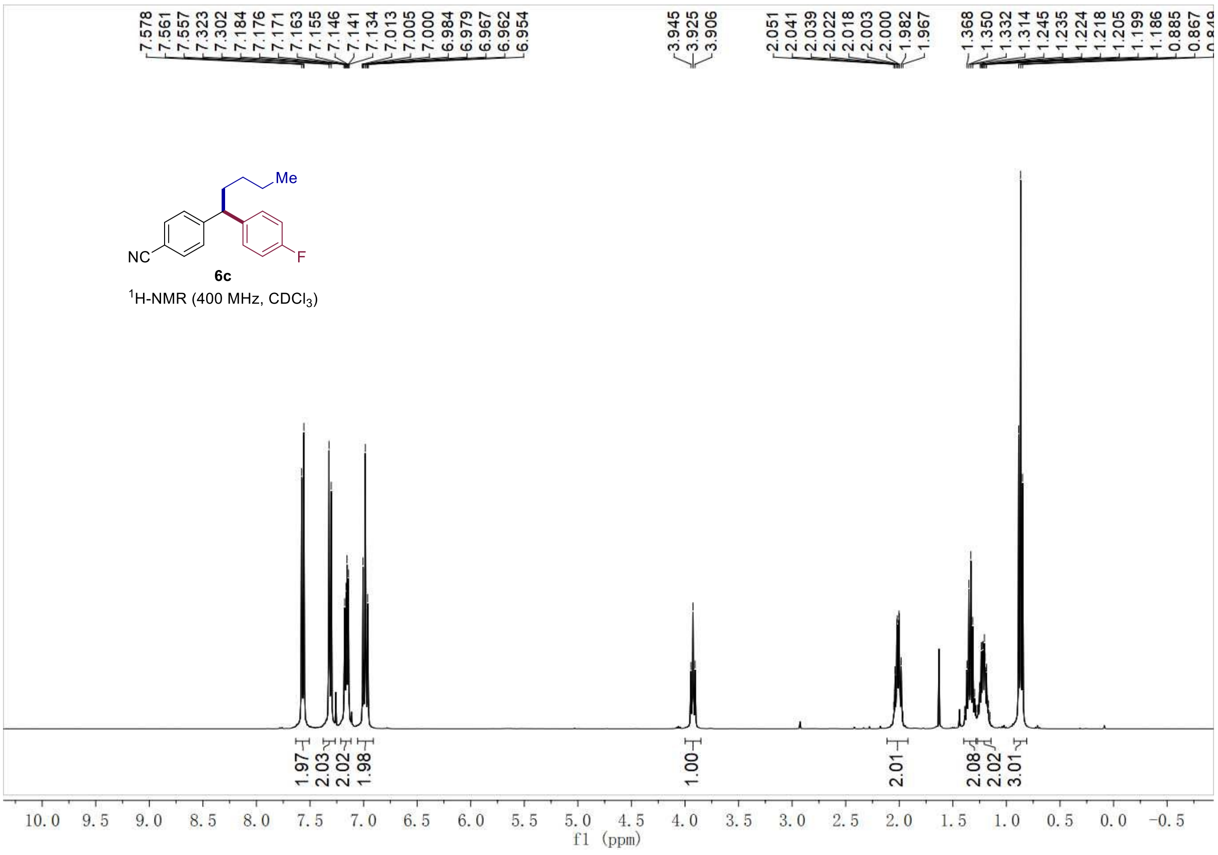
7.578 7.561 7.557 7.323 7.302 7.184 7.176 7.171 7.163 7.155 7.146 7.141 7.134 7.013 7.005 7.000 6.984 6.979 6.967 6.962 6.954
3.945 3.925 3.906
2.051 2.041 2.039 2.022 2.018 2.003 2.000 1.982 1.967
1.368 1.350 1.332 1.314 1.245 1.235 1.224 1.218 1.205 1.199 1.186 0.885 0.867
Me
NC
F
6c
1H-NMR (400 MHz, CDCl3)
1.97 2.03 2.02 1.98
1.00
2.01
2.08 2.02 3.01
10.0 9.5 9.0 8.5 8.0 7.5 7.0 6.5 6.0 5.5 5.0 4.5 4.0 3.5 3.0 2.5 2.0 1.5 1.0 0.5 0.0 -0.5
f1 (ppm)

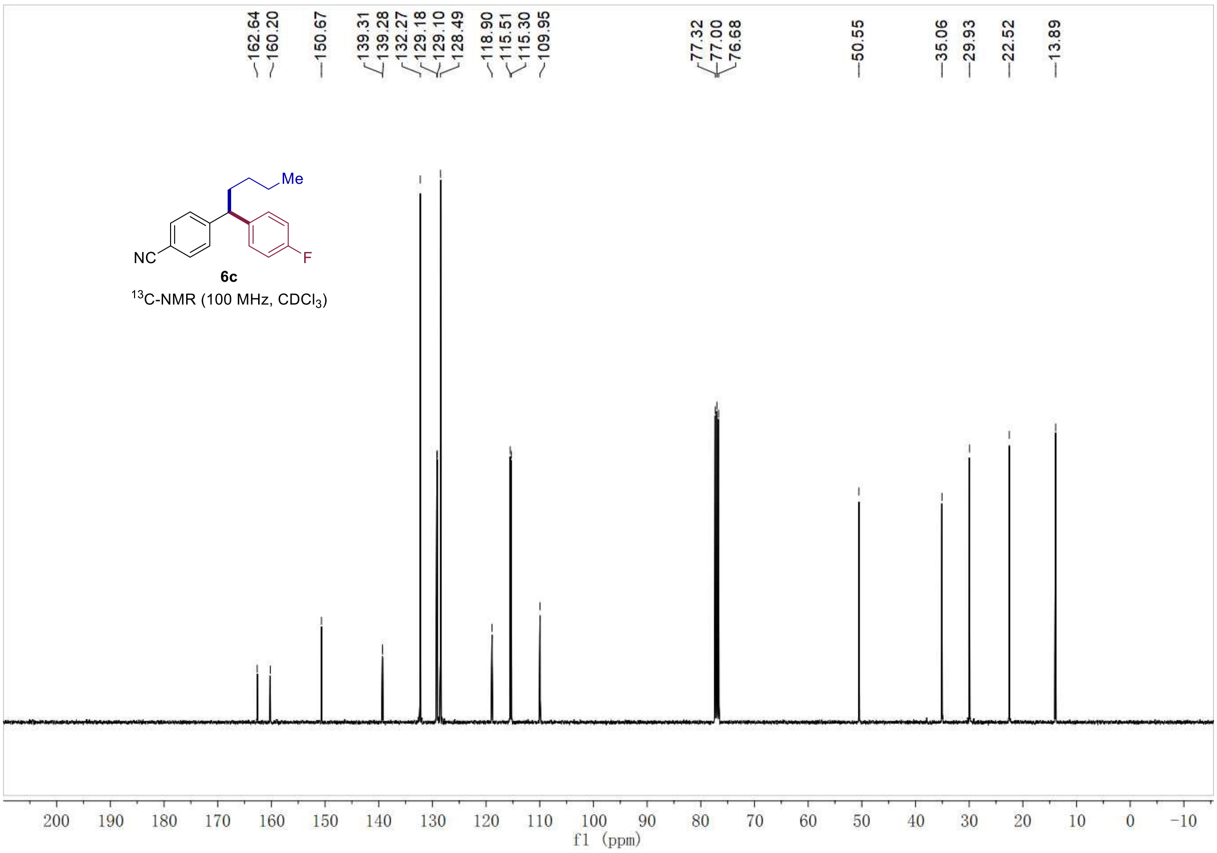
162.64
160.20
150.67
139.31
139.28
132.27
129.18
129.10
128.49
118.90
115.51
115.30
109.95
77.32
77.00
76.68
50.55
35.06
29.93
22.52
13.89
Me
NC
F
6c
^{13}C-NMR (100 MHz, $CDCl_3$)
200 190 180 170 160 150 140 130 120 110 100 90 80 70 60 50 40 30 20 10 0 −10
f1 (ppm)

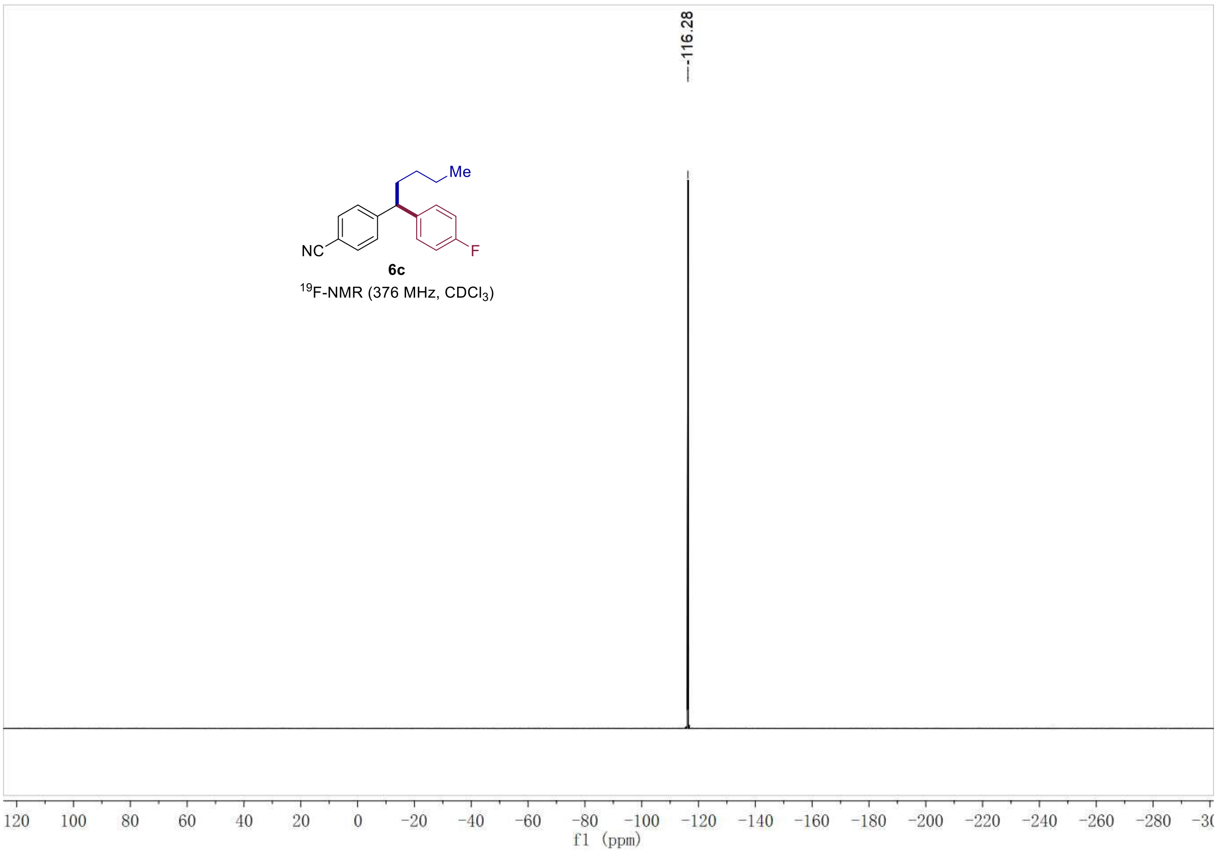
-116.28
Me
NC
F
6c
^{19}F-NMR (376 MHz, $CDCl_3$)
120 100 80 60 40 20 0 −20 −40 −60 −80 −100 −120 −140 −160 −180 −200 −220 −240 −260 −280
f1 (ppm)

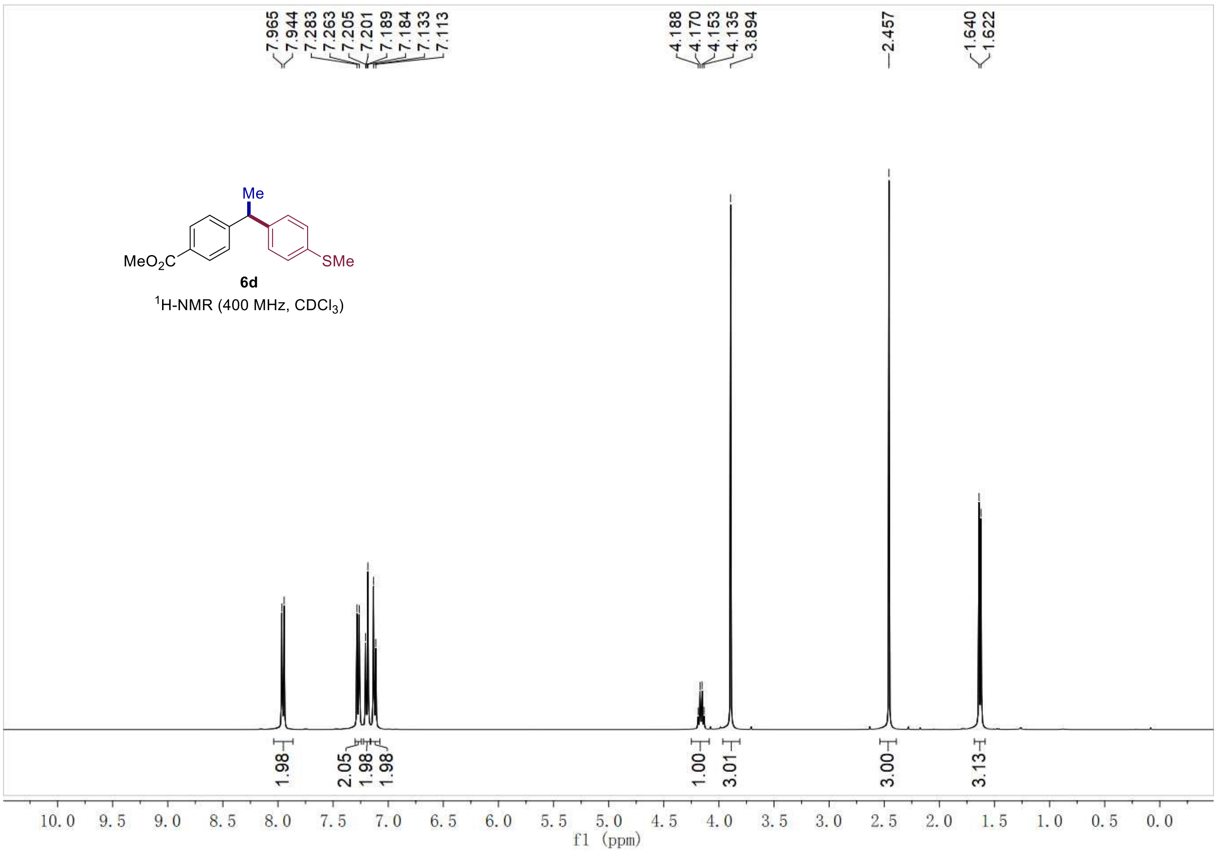
7.965
7.944
7.283
7.263
7.205
7.201
7.189
7.184
7.133
7.113
4.188
4.170
4.153
4.135
3.894
2.457
1.640
1.622
Me
MeO2C
SMe
6d
1H-NMR (400 MHz, CDCl3)
1.98
2.05
1.98
1.98
1.00
3.01
3.00
3.13
10.0 9.5 9.0 8.5 8.0 7.5 7.0 6.5 6.0 5.5 5.0 4.5 4.0 3.5 3.0 2.5 2.0 1.5 1.0 0.5 0.0
f1 (ppm)

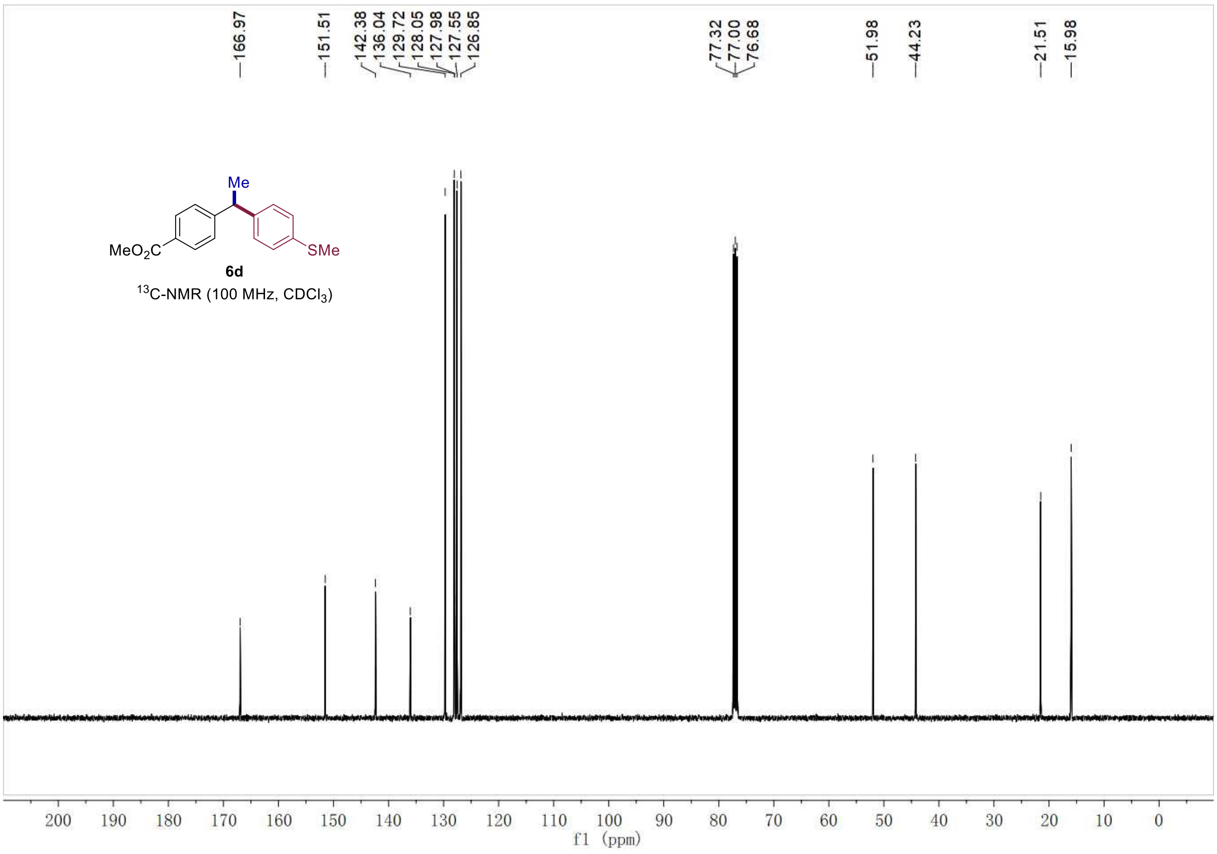
166.97
151.51
142.38
136.04
129.72
128.05
127.98
127.55
126.85
77.32
77.00
76.68
51.98
44.23
21.51
15.98
Me
MeO2C
SMe
6d
13C-NMR (100 MHz, CDCl3)
200 190 180 170 160 150 140 130 120 110 100 90 80 70 60 50 40 30 20 10 0
f1 (ppm)

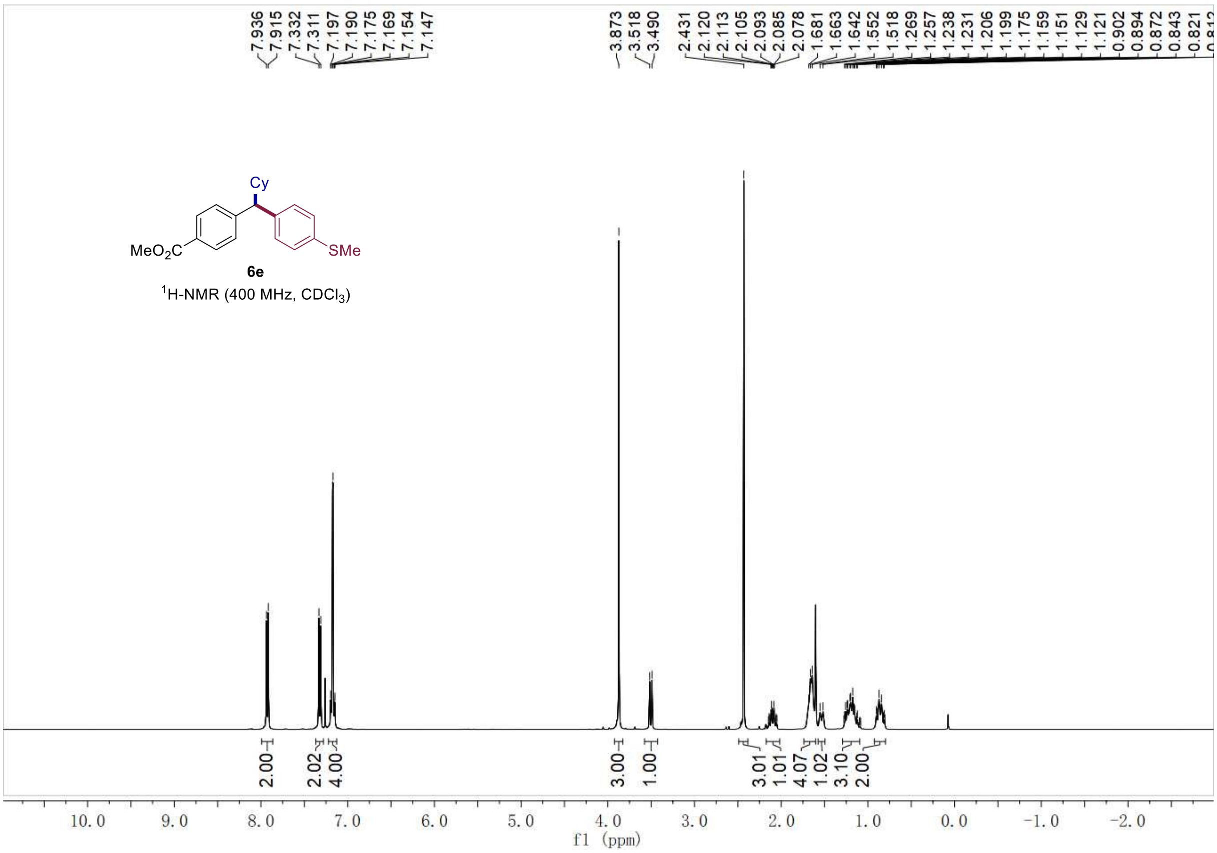
7.936
7.915
7.332
7.311
7.197
7.190
7.175
7.169
7.154
7.147
3.873
3.518
3.490
2.431
2.120
2.113
2.105
2.093
2.085
2.078
1.681
1.663
1.642
1.552
1.518
1.269
1.257
1.238
1.231
1.206
1.199
1.175
1.159
1.151
1.129
1.121
0.902
0.894
0.872
0.843
0.821
Cy
MeO2C
SMe
6e
1H-NMR (400 MHz, CDCl3)
2.00
2.02
4.00
3.00
1.00
3.01
1.01
4.07
1.02
3.10
2.00
10.0
9.0
8.0
7.0
6.0
5.0
4.0
3.0
2.0
1.0
0.0
-1.0
-2.0
f1 (ppm)

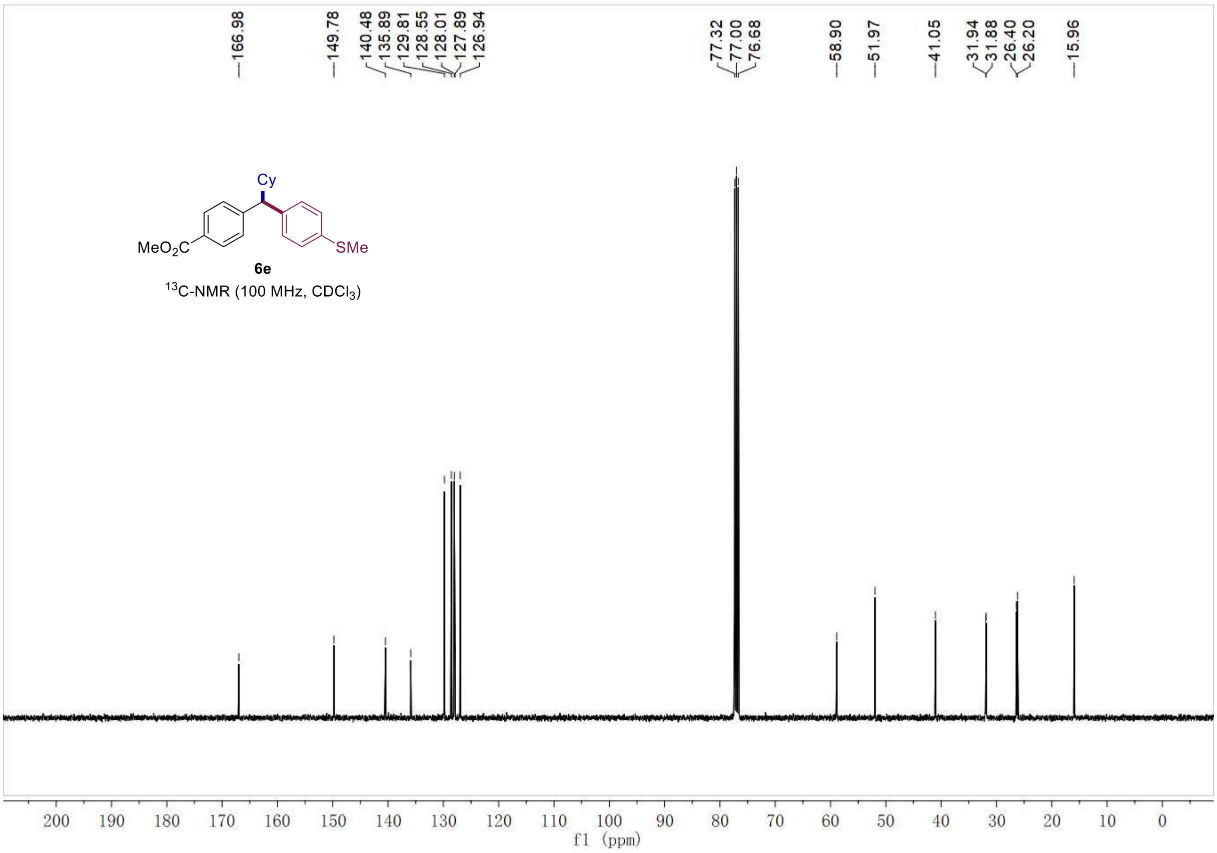
166.98
149.78
140.48
135.89
129.81
128.55
128.01
127.89
126.94
77.32
77.00
76.68
58.90
51.97
41.05
31.94
31.88
26.40
26.20
15.96
Cy
MeO2C
SMe
6e
13C-NMR (100 MHz, CDCl3)
200
190
180
170
160
150
140
130
120
110
100
90
80
70
60
50
40
30
20
10
0
f1 (ppm)

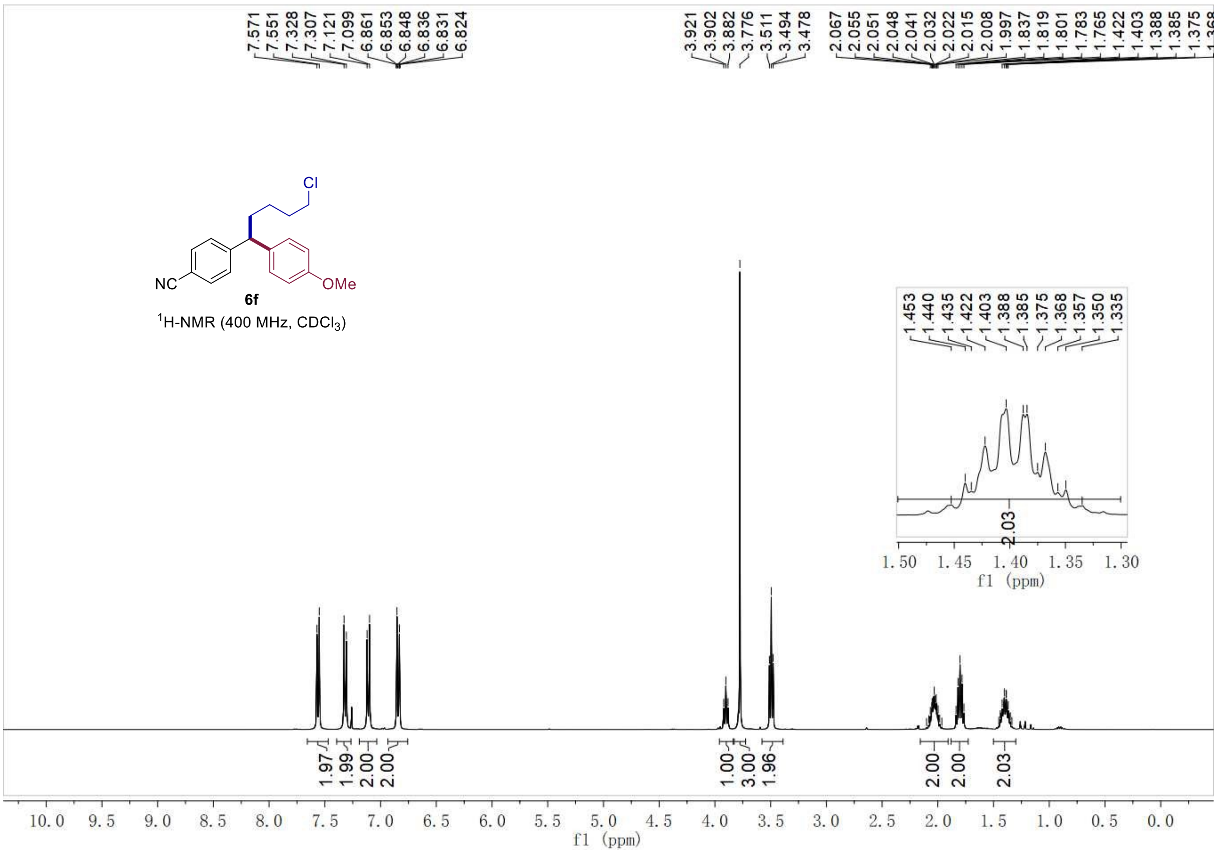
Cl
NC
OMe
6f
1H-NMR (400 MHz, CDCl3)
f1 (ppm)

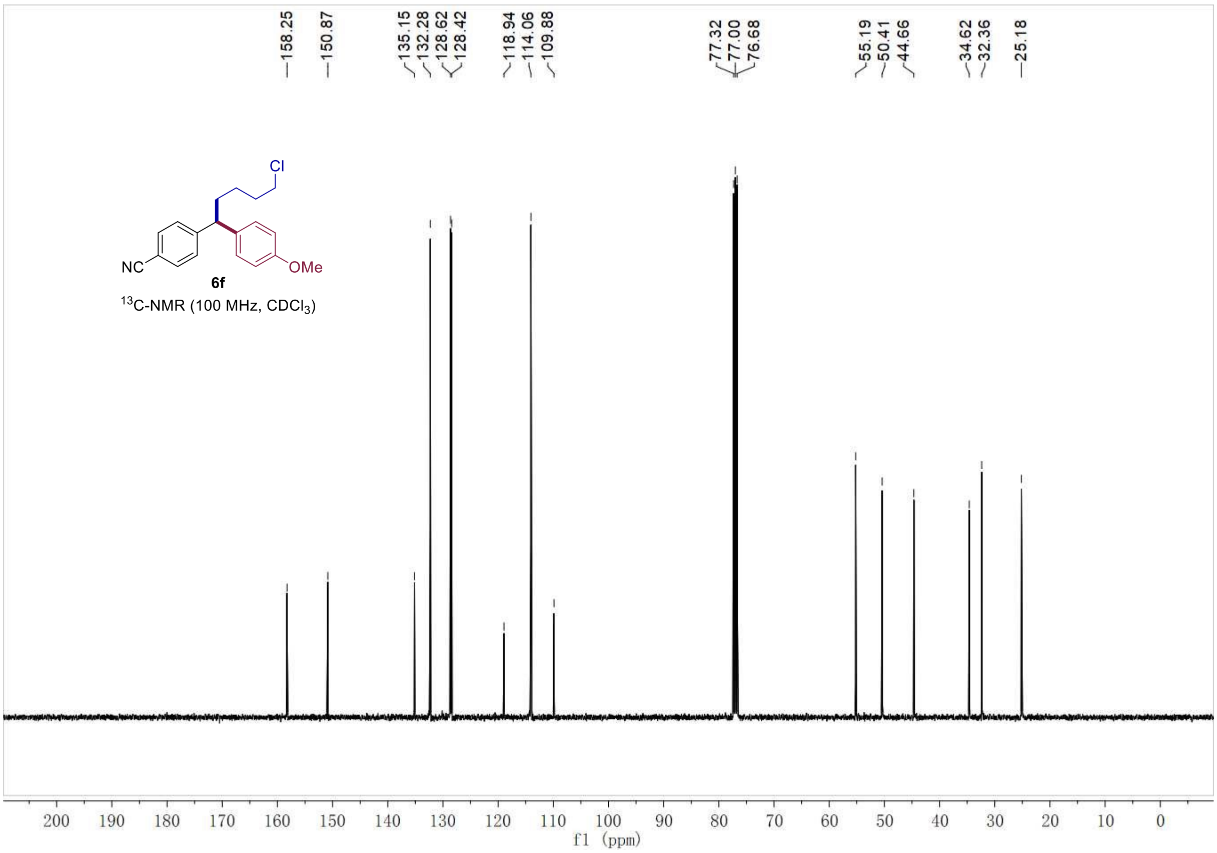
Cl
NC
OMe
6f
13C-NMR (100 MHz, CDCl3)
f1 (ppm)

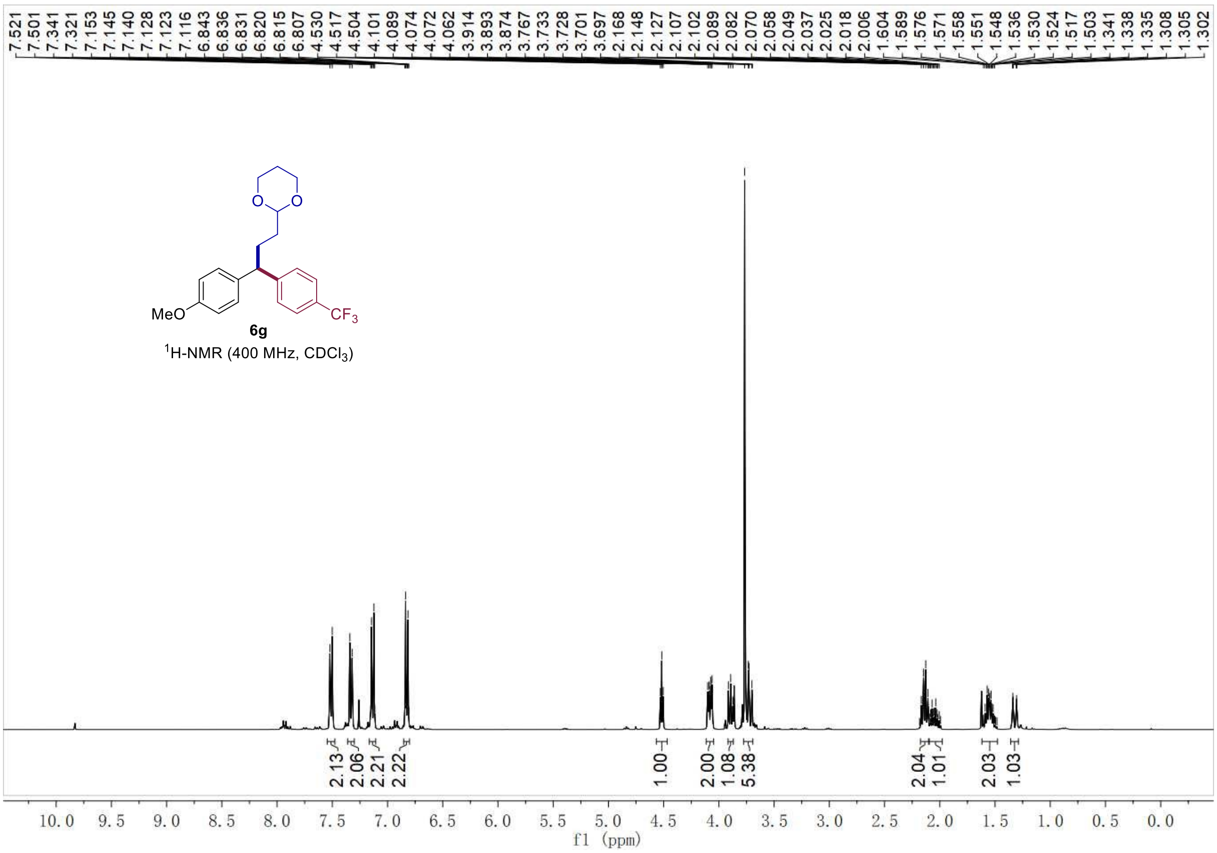
7.521
7.501
7.341
7.321
7.153
7.145
7.140
7.128
7.123
7.116
6.843
6.836
6.831
6.820
6.815
6.807
4.530
4.517
4.504
4.101
4.089
4.074
4.072
4.062
3.914
3.893
3.874
3.767
3.733
3.728
3.701
3.697
2.168
2.148
2.127
2.107
2.102
2.089
2.082
2.070
2.058
2.049
2.037
2.025
2.018
2.006
1.604
1.589
1.576
1.571
1.558
1.551
1.548
1.536
1.530
1.524
1.517
1.503
1.341
1.338
1.335
1.308
1.305
1.302
MeO
CF3
6g
1H-NMR (400 MHz, CDCl3)
2.13
2.06
2.21
2.22
1.00
2.00
1.08
5.38
2.04
1.01
2.03
1.03
f1 (ppm)

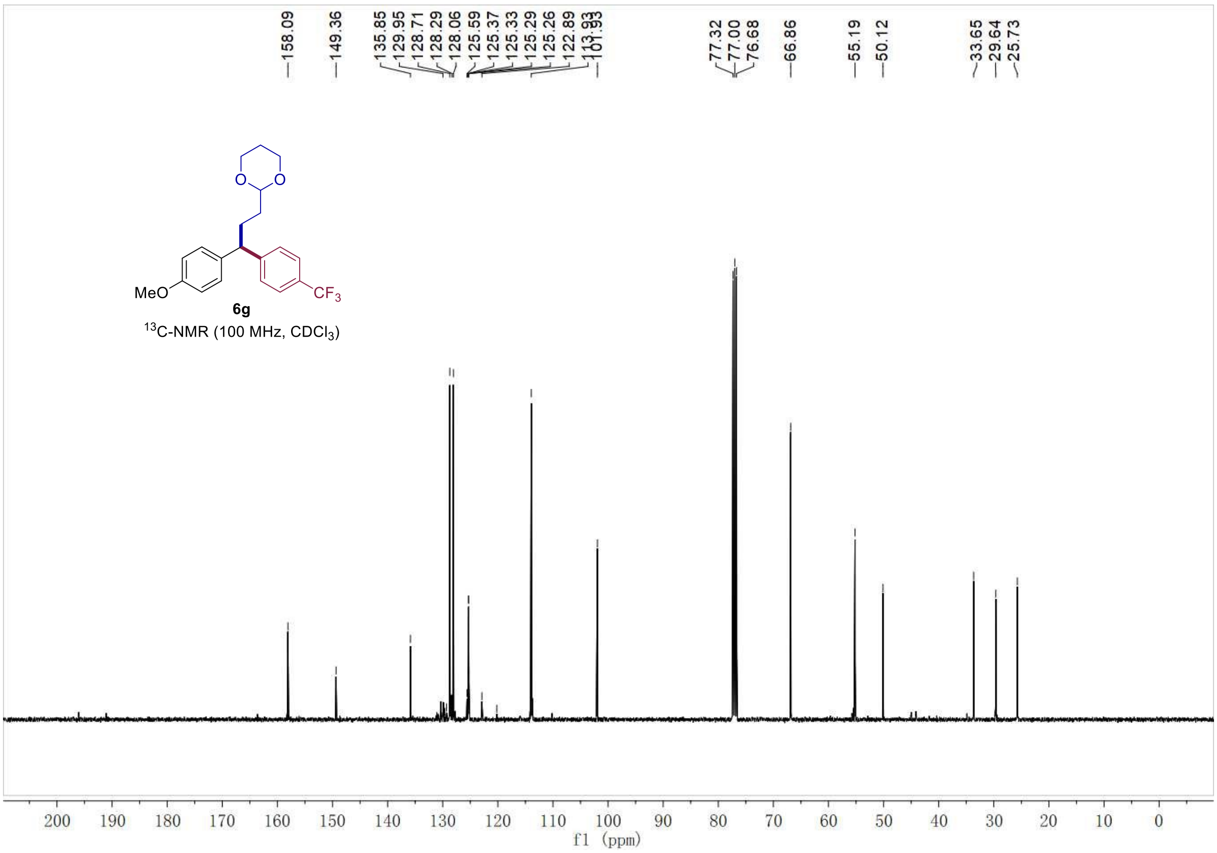
158.09
149.36
135.85
129.95
128.71
128.29
128.06
125.59
125.37
125.33
125.29
125.26
122.89
113.93
101.93
77.32
77.00
76.68
66.86
55.19
50.12
33.65
29.64
25.73
MeO
CF3
6g
13C-NMR (100 MHz, CDCl3)
f1 (ppm)

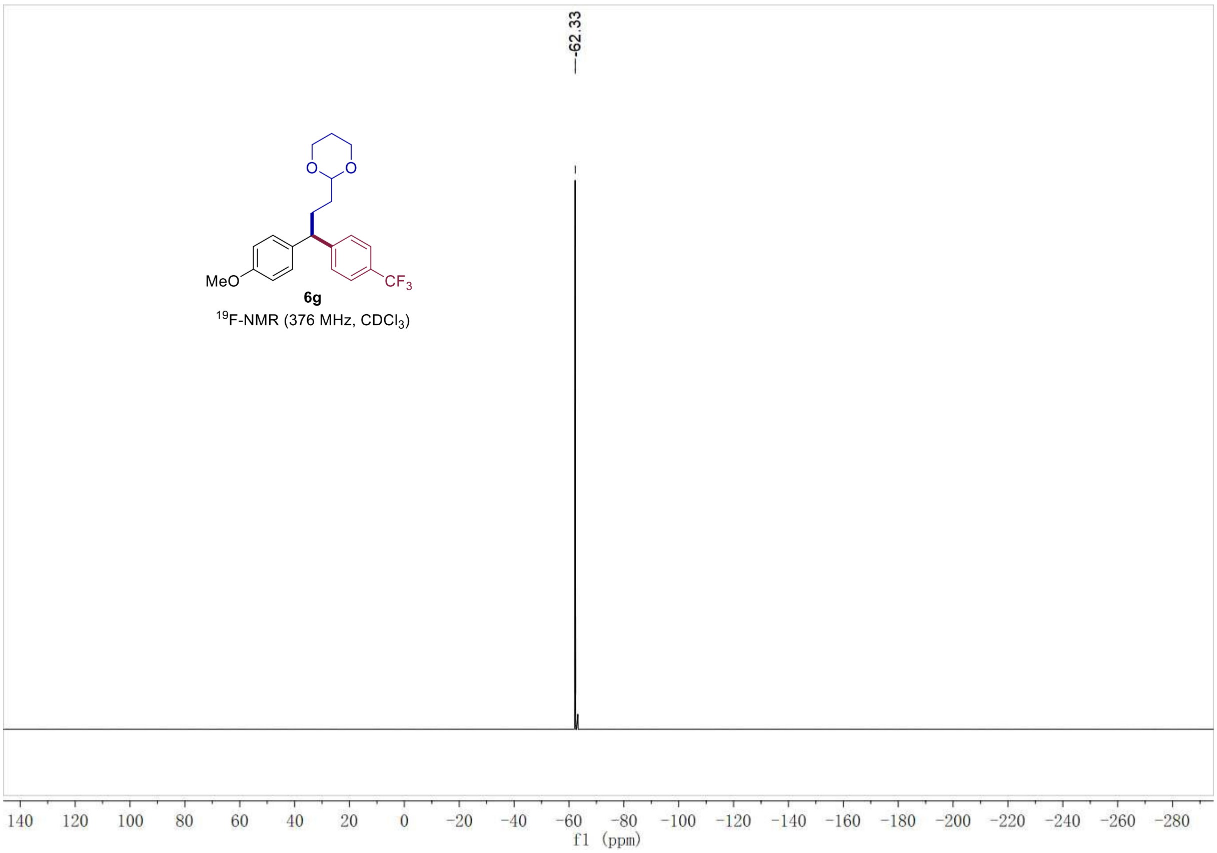
-62.33
O
O
MeO
CF3
6g
19F-NMR (376 MHz, CDCl3)
140 120 100 80 60 40 20 0 -20 -40 -60 -80 -100 -120 -140 -160 -180 -200 -220 -240 -260 -280
f1 (ppm)

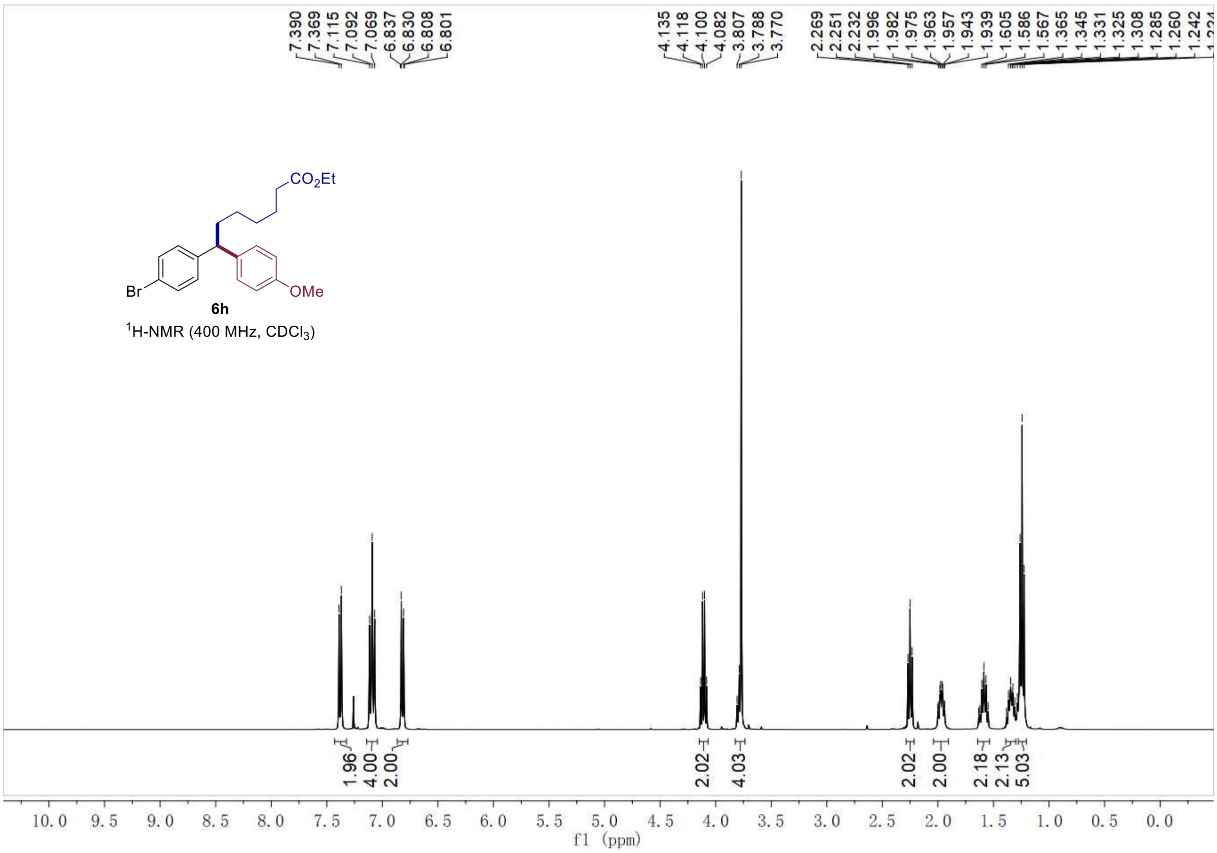
7.390
7.369
7.115
7.092
7.069
6.837
6.830
6.808
6.801
4.135
4.118
4.100
4.082
3.807
3.788
3.770
2.269
2.251
2.232
1.996
1.982
1.975
1.963
1.957
1.943
1.939
1.605
1.586
1.567
1.365
1.345
1.331
1.325
1.308
1.285
1.260
1.242
CO2Et
Br
OMe
6h
1H-NMR (400 MHz, CDCl3)
1.96
4.00
2.00
2.02
4.03
2.02
2.00
2.18
2.13
5.03
10.0 9.5 9.0 8.5 8.0 7.5 7.0 6.5 6.0 5.5 5.0 4.5 4.0 3.5 3.0 2.5 2.0 1.5 1.0 0.5 0.0
f1 (ppm)

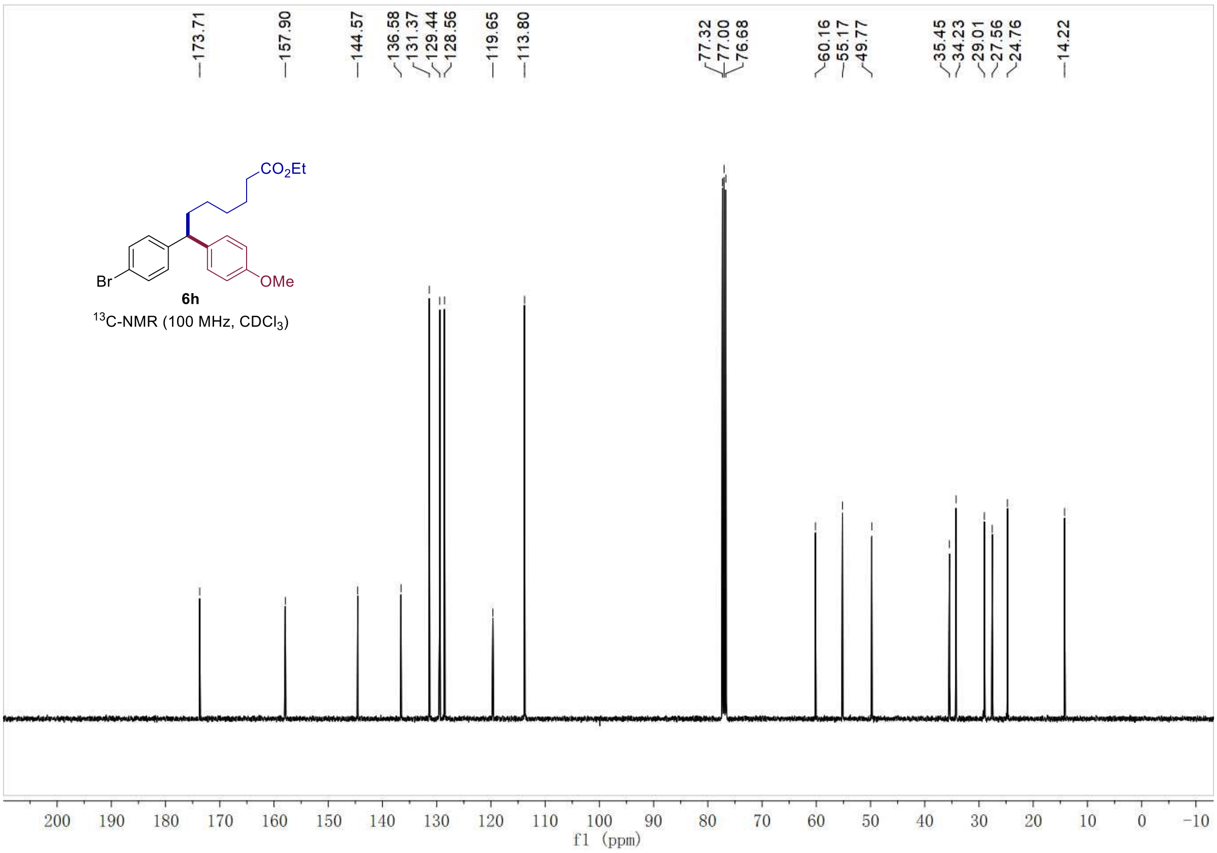
173.71
157.90
144.57
136.58
131.37
129.44
128.56
119.65
113.80
77.32
77.00
76.68
60.16
55.17
49.77
35.45
34.23
29.01
27.56
24.76
14.22
CO2Et
Br
OMe
6h
13C-NMR (100 MHz, CDCl3)
f1 (ppm)

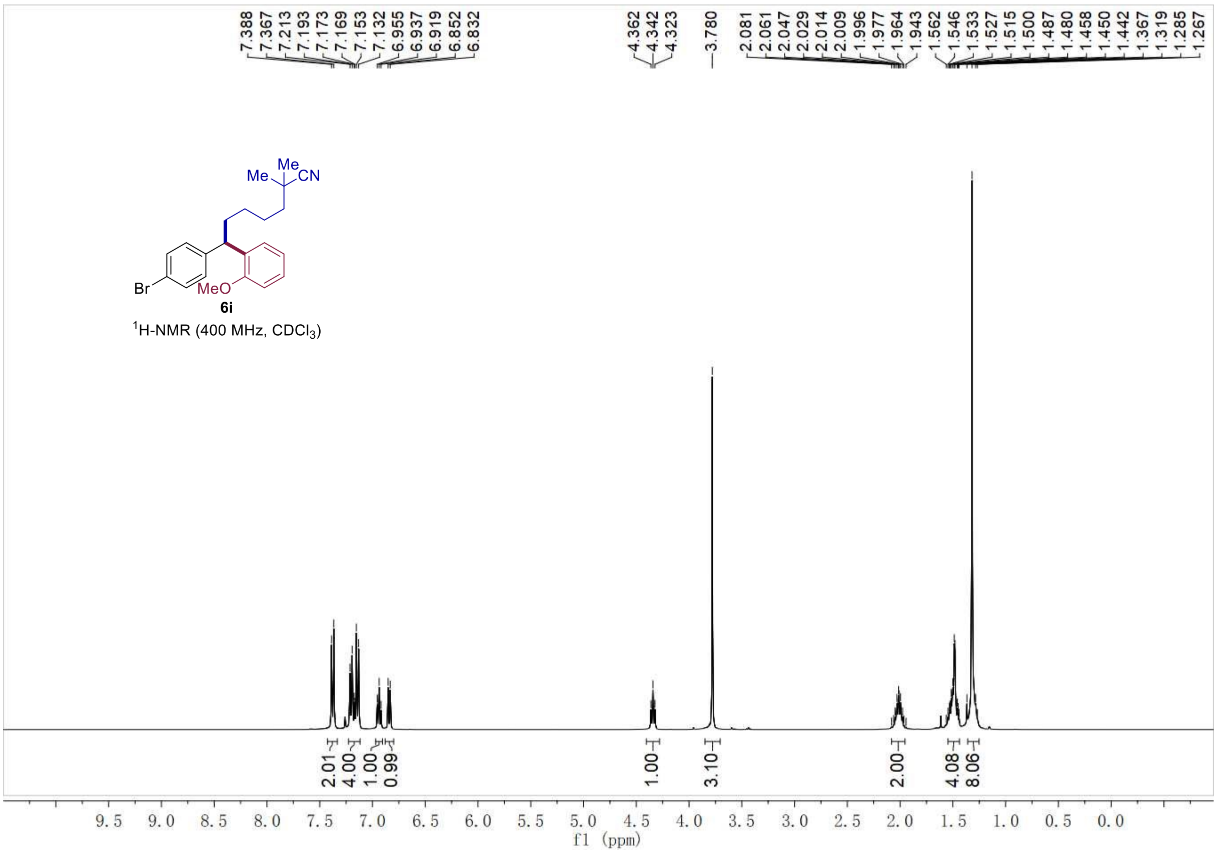
7.388
7.367
7.213
7.193
7.173
7.169
7.153
7.132
6.955
6.937
6.919
6.852
6.832
4.362
4.342
4.323
3.780
2.081
2.061
2.047
2.029
2.014
2.009
1.996
1.977
1.964
1.943
1.562
1.546
1.533
1.527
1.515
1.500
1.487
1.480
1.458
1.450
1.442
1.367
1.319
1.285
1.267
Me
Me
CN
Br
MeO
6i
1H-NMR (400 MHz, CDCl3)
2.01
4.00
1.00
0.99
1.00
3.10
2.00
4.08
8.06
f1 (ppm)

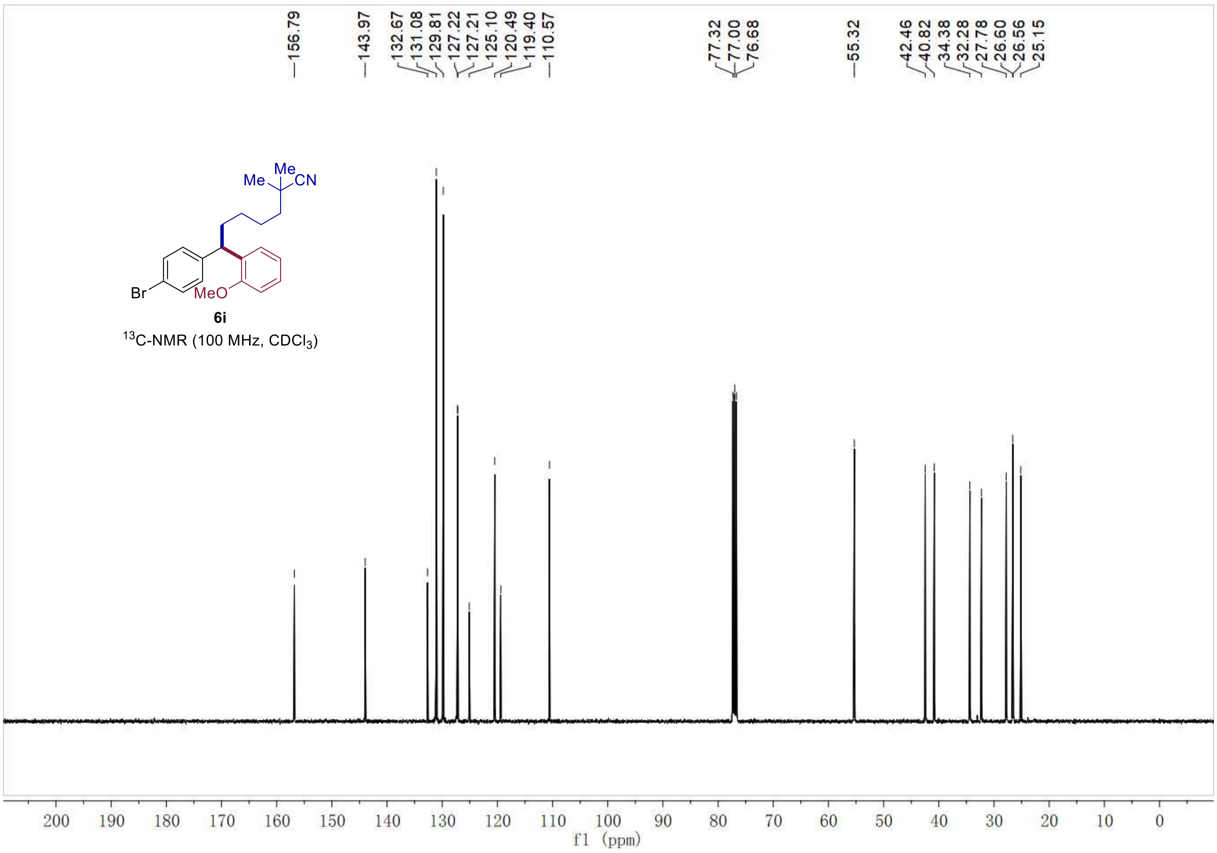

6i
^{13}C-NMR (100 MHz, $CDCl_3$)

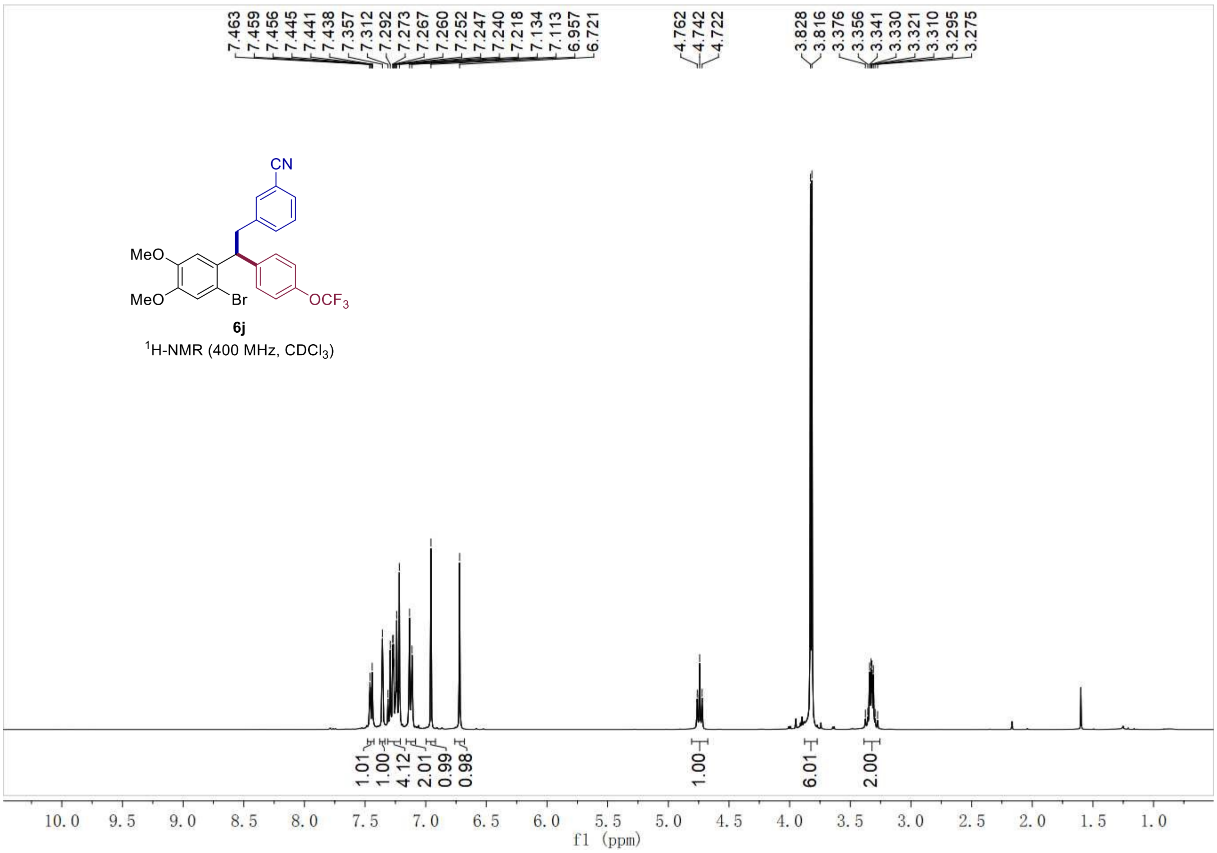

6j
^{1}H-NMR (400 MHz, $CDCl_3$)

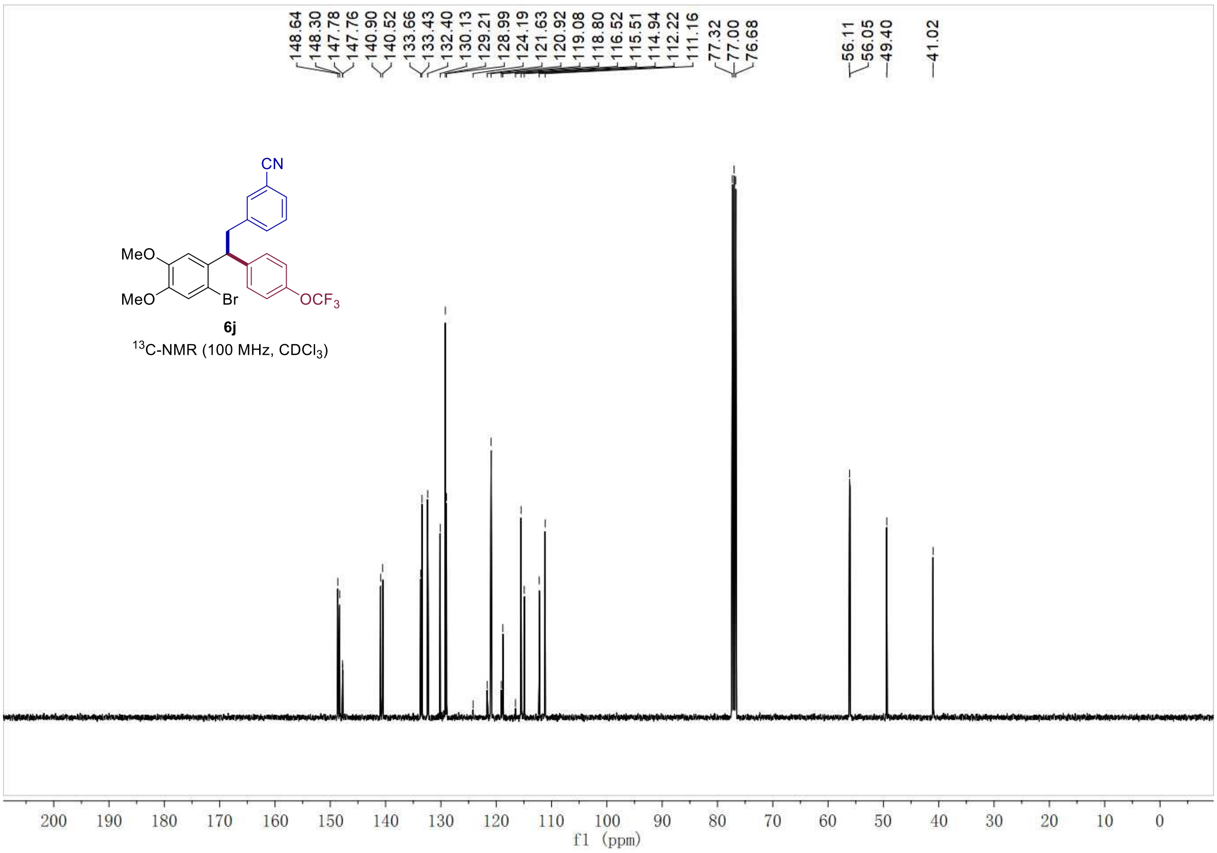
148.64
148.30
147.78
147.76
140.90
140.52
133.66
133.43
132.40
130.13
129.21
128.99
124.19
121.63
120.92
119.08
118.80
116.52
115.51
114.94
112.22
111.16
77.32
77.00
76.68
56.11
56.05
49.40
41.02
CN
MeO
MeO
Br
OCF3
6j
13C-NMR (100 MHz, CDCl3)
200 190 180 170 160 150 140 130 120 110 100 90 80 70 60 50 40 30 20 10 0
f1 (ppm)

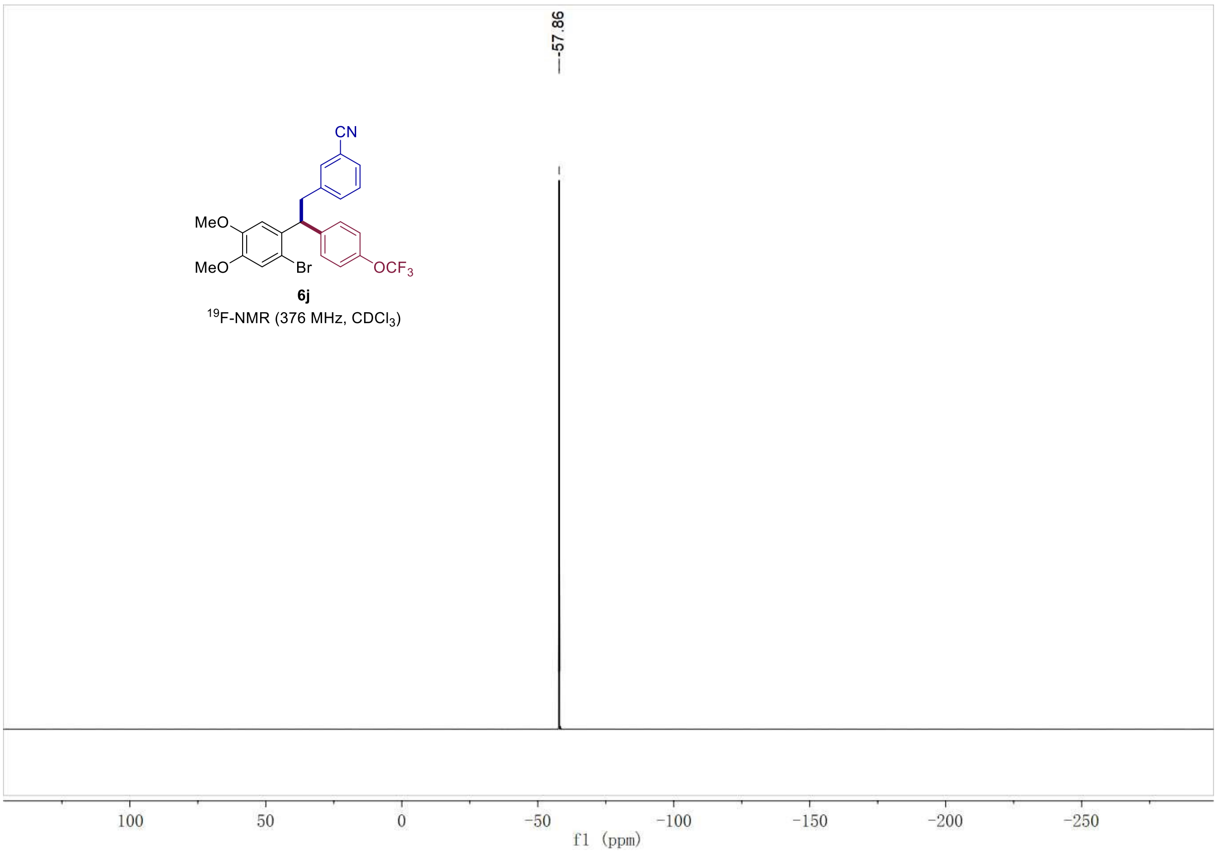
-57.86
CN
MeO
MeO
Br
OCF3
6j
19F-NMR (376 MHz, CDCl3)
100 50 0 -50 -100 -150 -200 -250
f1 (ppm)

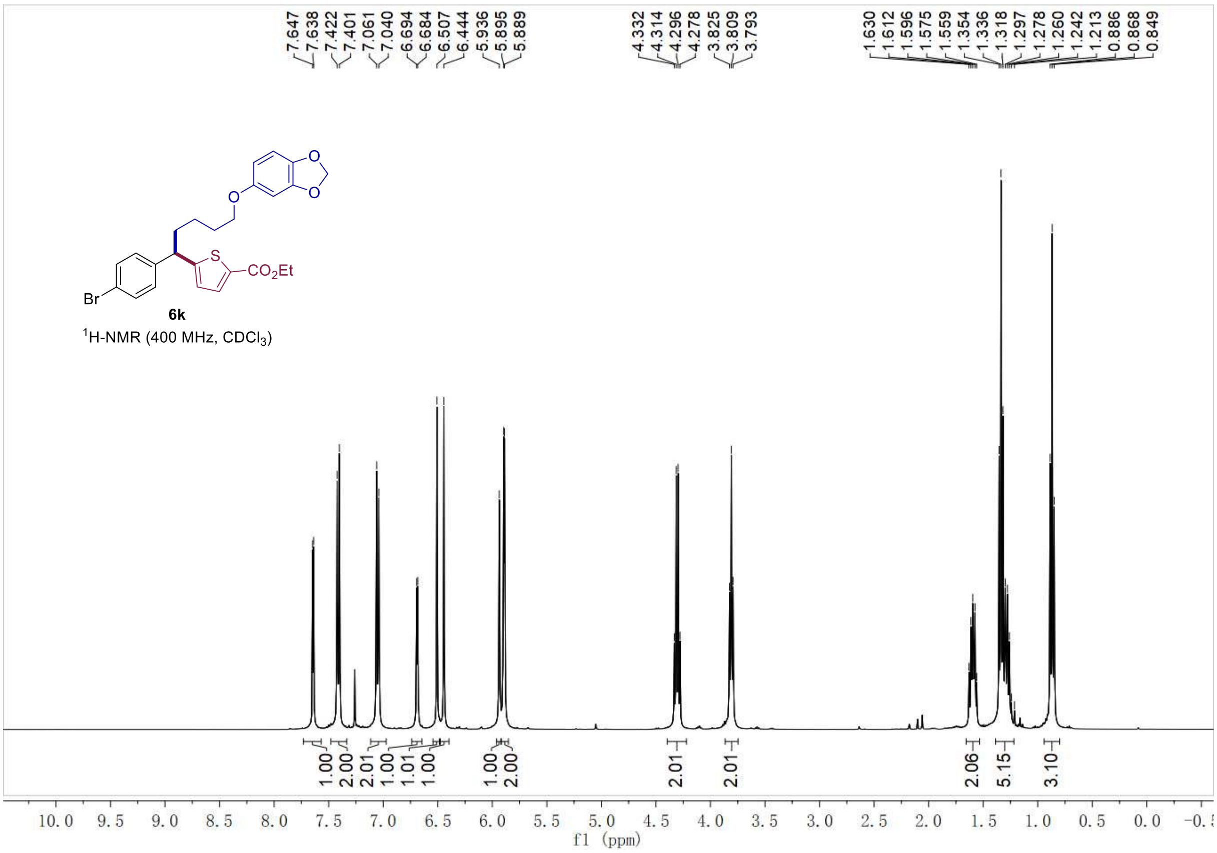
7.647
7.638
7.422
7.401
7.061
7.040
6.694
6.684
6.507
6.444
5.936
5.895
5.889
4.332
4.314
4.296
4.278
3.825
3.809
3.793
1.630
1.612
1.596
1.575
1.559
1.354
1.336
1.318
1.297
1.278
1.260
1.242
1.213
0.886
0.868
0.849
O
O
O
S
CO2Et
Br
6k
1H-NMR (400 MHz, CDCl3)
1.00
2.00
2.01
1.00
1.01
1.00
1.00
2.00
2.01
2.01
2.06
5.15
3.10
f1 (ppm)

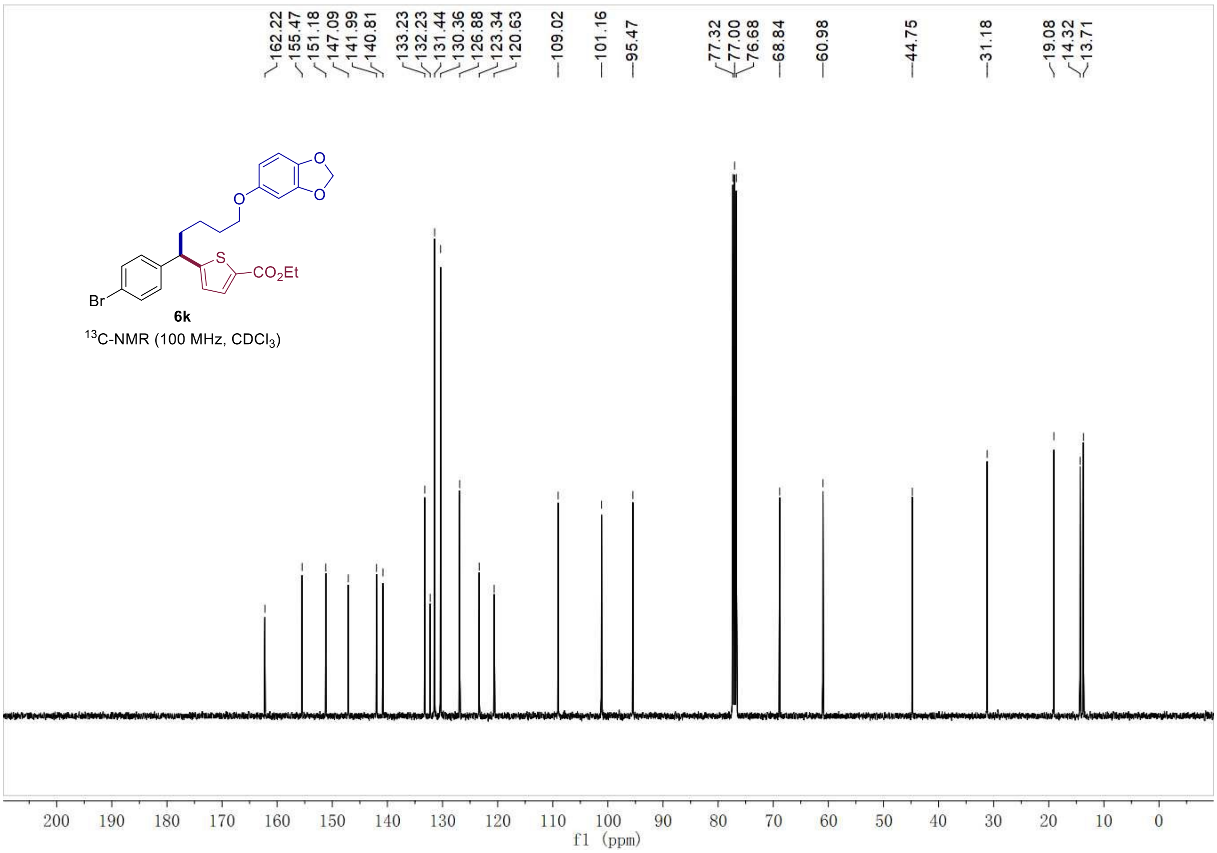
162.22
155.47
151.18
147.09
141.99
140.81
133.23
132.23
131.44
130.36
126.88
123.34
120.63
109.02
101.16
95.47
77.32
77.00
76.68
68.84
60.98
44.75
31.18
19.08
14.32
13.71
O
O
O
S
CO2Et
Br
6k
13C-NMR (100 MHz, CDCl3)
f1 (ppm)

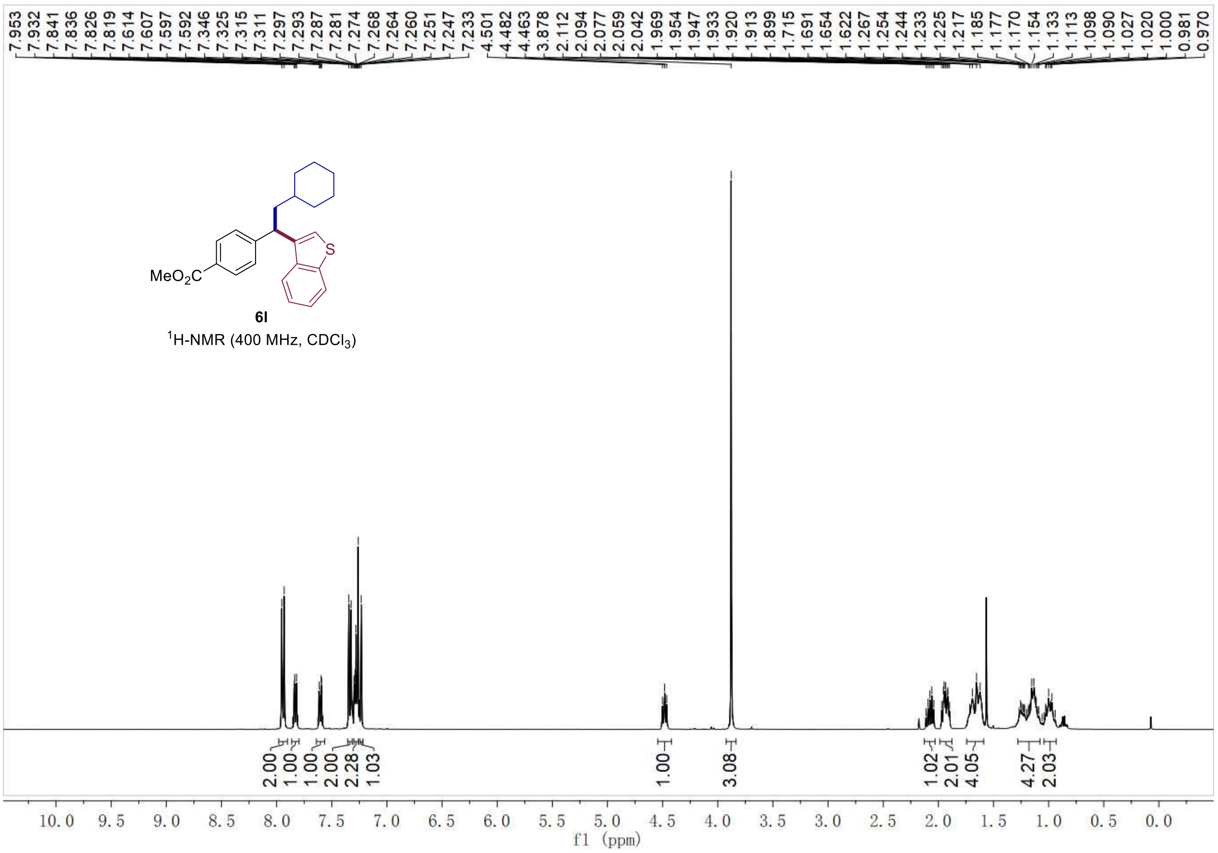
7.953 7.932 7.841 7.836 7.826 7.819 7.614 7.607 7.597 7.592 7.346 7.325 7.315 7.311 7.297 7.293 7.287 7.281 7.274 7.268 7.264 7.260 7.251 7.247 7.233 4.501 4.482 4.463 3.878 2.112 2.094 2.077 2.059 2.042 1.969 1.954 1.947 1.933 1.920 1.913 1.899 1.715 1.691 1.654 1.622 1.267 1.254 1.244 1.233 1.225 1.217 1.185 1.177 1.170 1.154 1.133 1.113 1.098 1.090 1.027 1.020 1.000 0.981 0.970
MeO2C
S
6l
1H-NMR (400 MHz, CDCl3)
2.00 1.00 1.00 2.00 2.28 1.03
1.00
3.08
1.02 2.01 4.05
4.27 2.03
f1 (ppm)

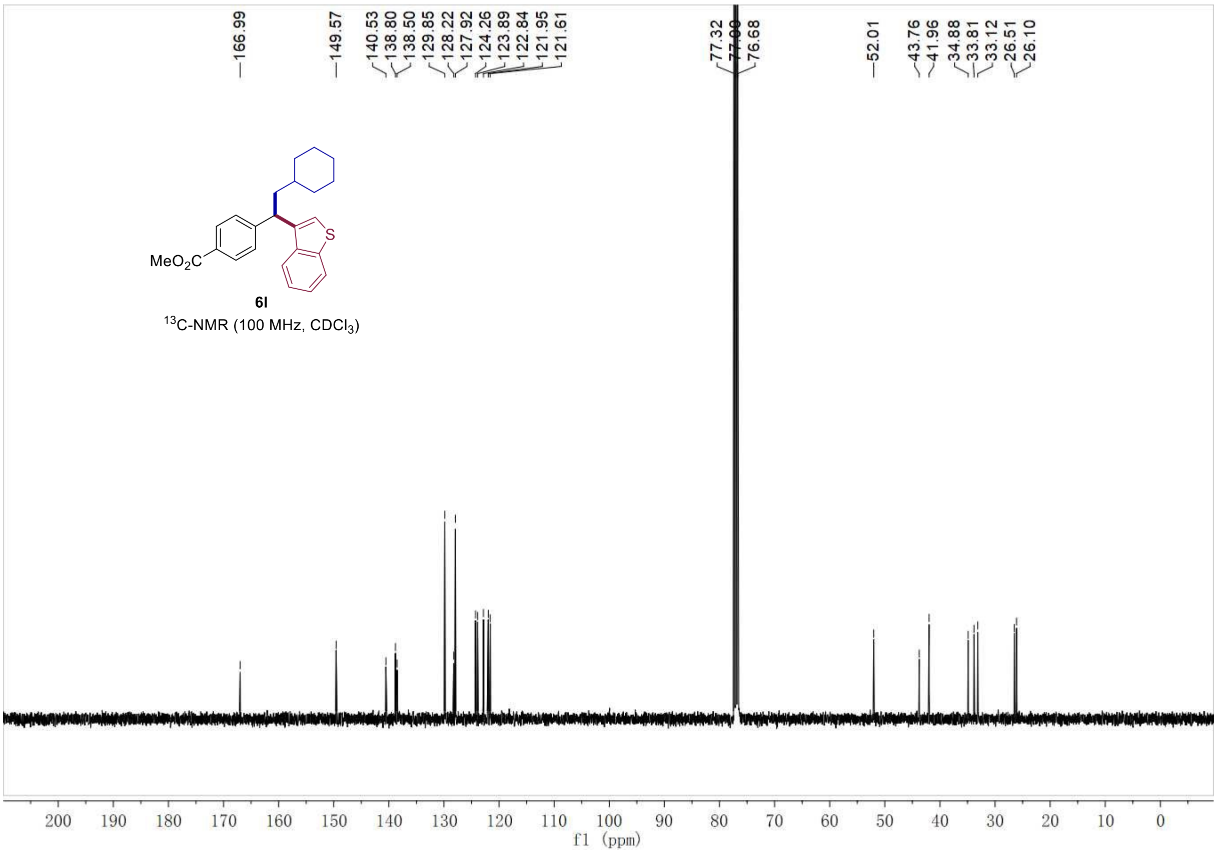
166.99
149.57
140.53 138.80 138.50 129.85 128.22 127.92 124.26 123.89 122.84 121.95 121.61
77.32 77.00 76.68
52.01
43.76 41.96 34.88 33.81 33.12 26.51 26.10
MeO2C
S
6l
13C-NMR (100 MHz, CDCl3)
f1 (ppm)

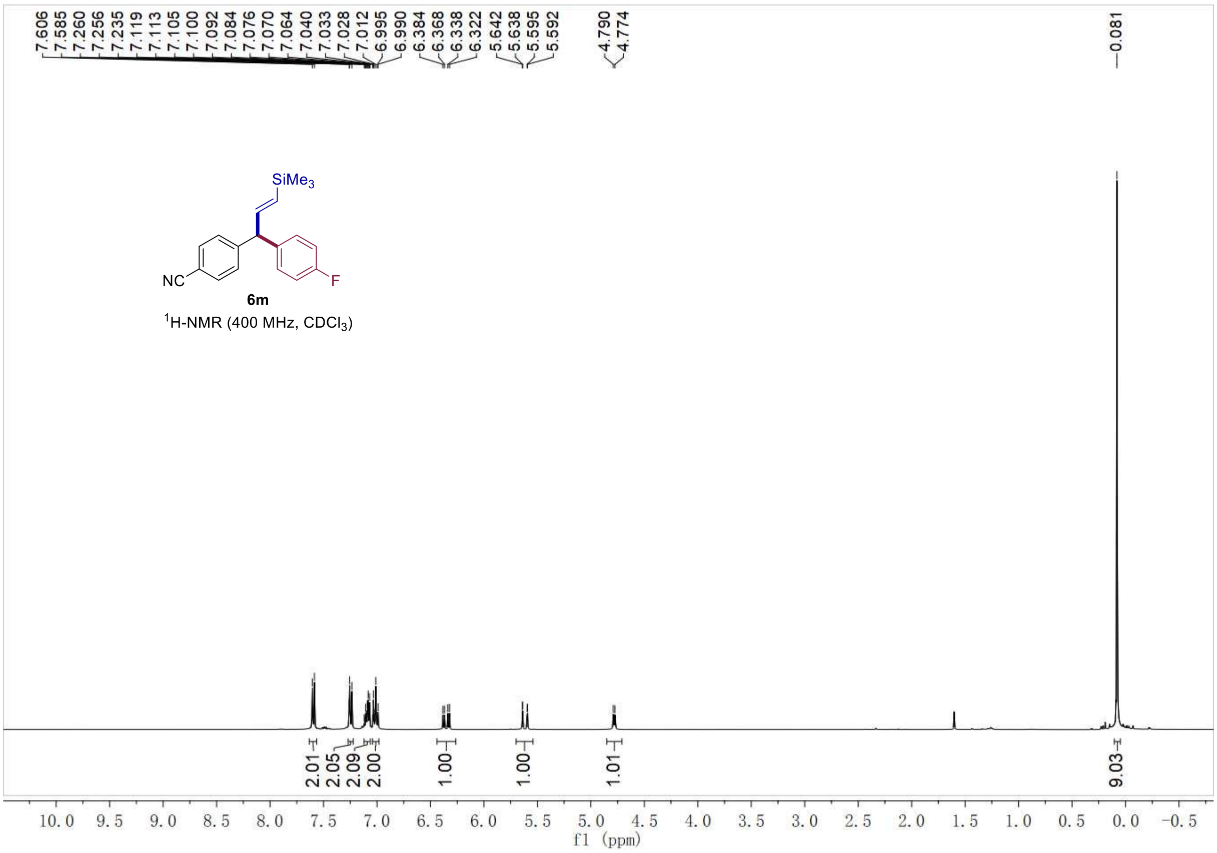
SiMe3
NC
F
6m
1H-NMR (400 MHz, CDCl3)
7.606 7.585 7.260 7.256 7.235 7.119 7.113 7.105 7.100 7.092 7.084 7.076 7.070 7.064 7.040 7.033 7.028 7.012 6.995 6.990 6.384 6.368 6.338 6.322 5.642 5.638 5.595 5.592 4.790 4.774 0.081
2.01 2.05 2.09 2.00 1.00 1.00 1.01 9.03
f1 (ppm)

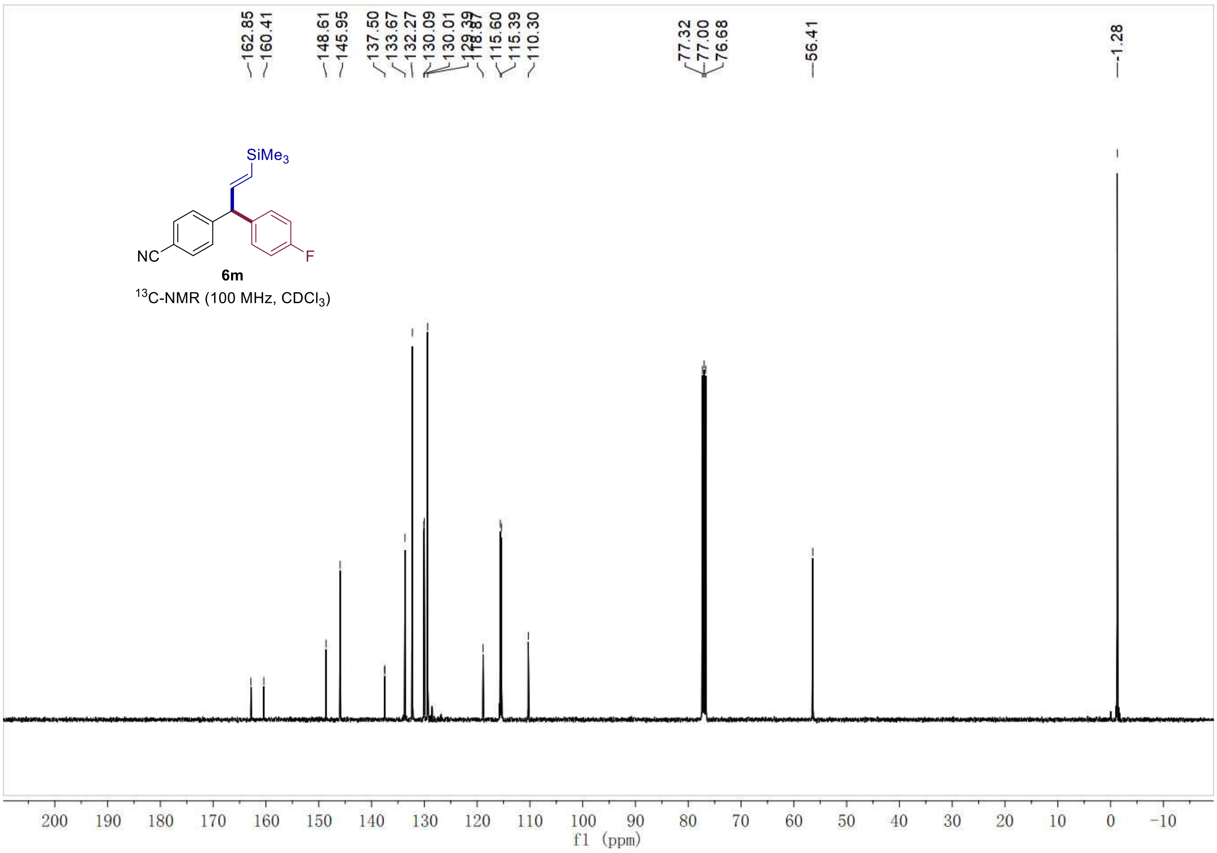
SiMe3
NC
F
6m
13C-NMR (100 MHz, CDCl3)
162.85 160.41 148.61 145.95 137.50 133.67 132.27 130.09 130.01 115.60 115.39 110.30 77.32 77.00 76.68 56.41 -1.28
f1 (ppm)

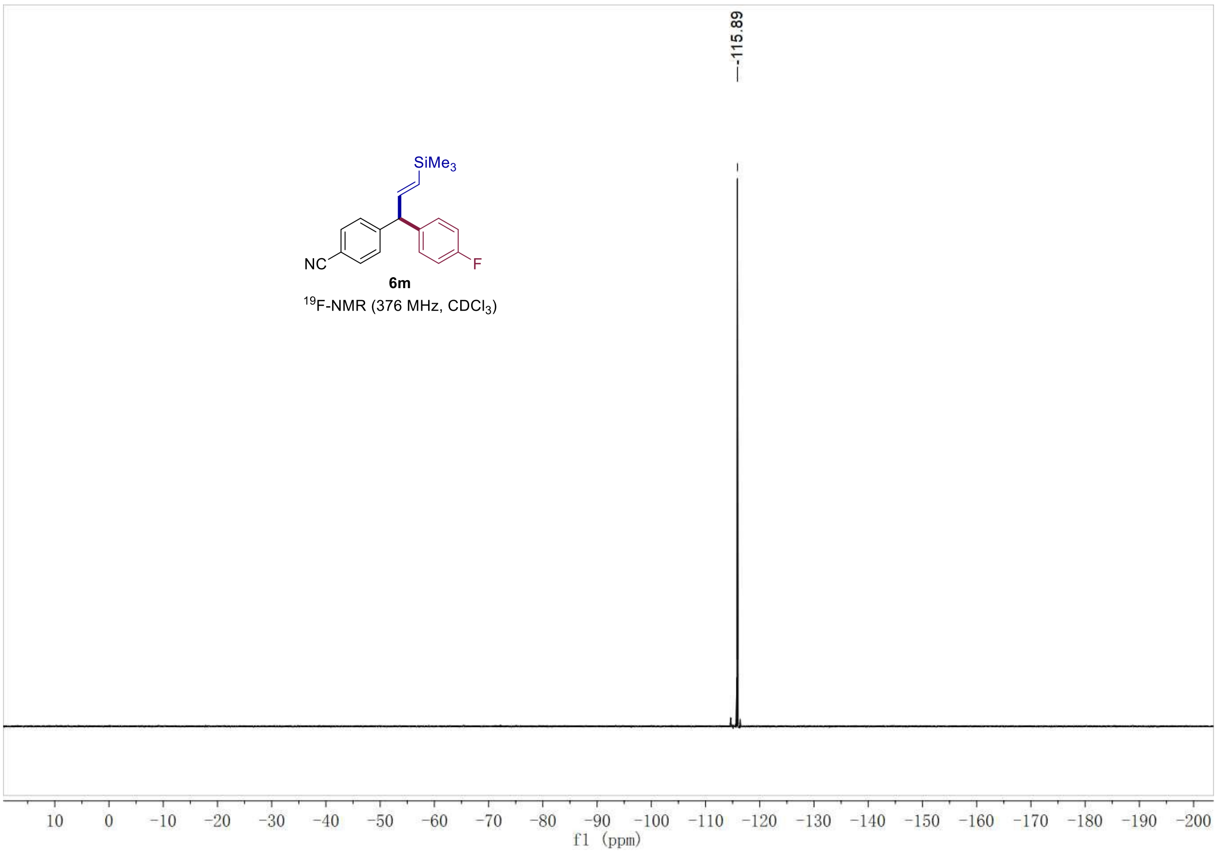
-115.89
SiMe3
NC
F
6m
19F-NMR (376 MHz, CDCl3)
10 0 −10 −20 −30 −40 −50 −60 −70 −80 −90 −100 −110 −120 −130 −140 −150 −160 −170 −180 −190 −200
f1 (ppm)

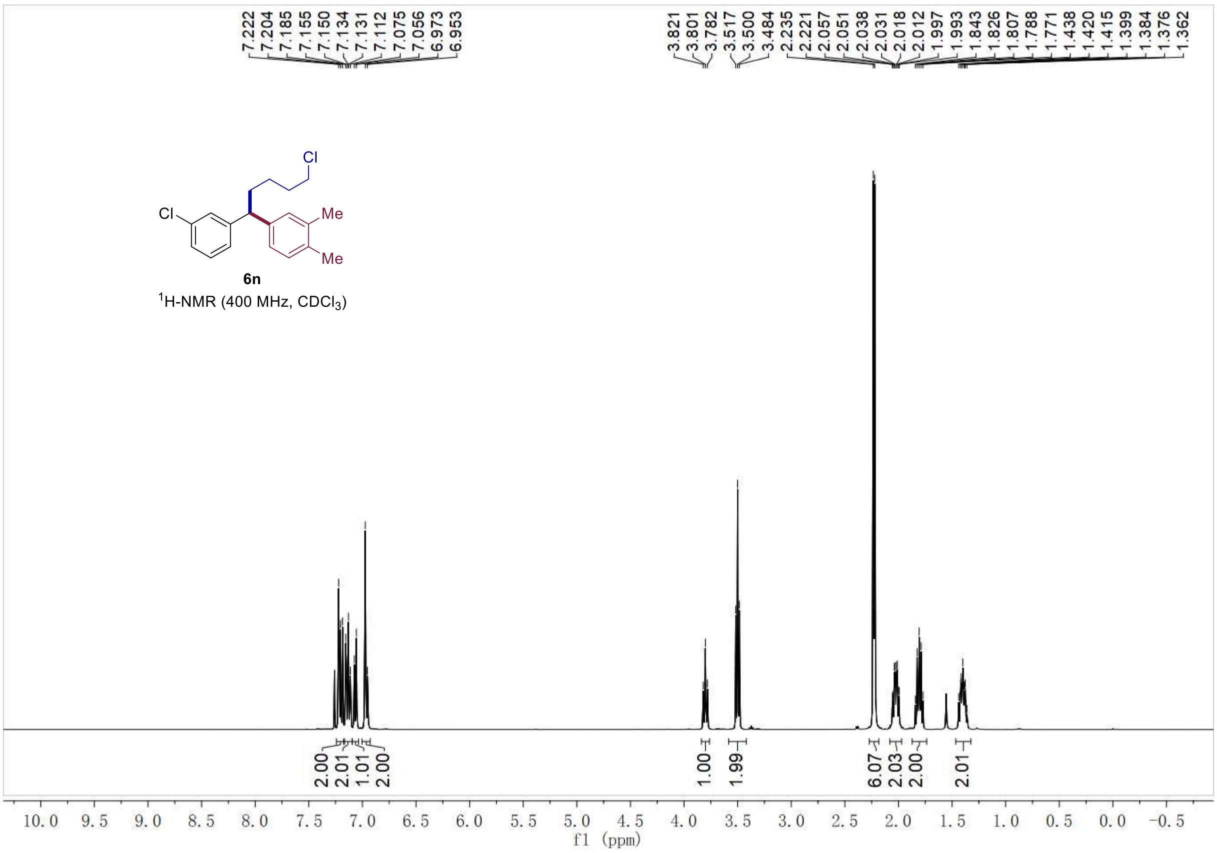
7.222 7.204 7.185 7.155 7.150 7.134 7.131 7.112 7.075 7.056 6.973 6.953
3.821 3.801 3.782 3.517 3.500 3.484 2.235 2.221 2.057 2.051 2.038 2.031 2.018 2.012 1.997 1.993 1.843 1.826 1.807 1.788 1.771 1.438 1.420 1.415 1.399 1.384 1.376 1.362
Cl
Cl
Me
Me
6n
1H-NMR (400 MHz, CDCl3)
2.00 2.01 1.01 2.00 1.00 1.99 6.07 2.03 2.00 2.01
10.0 9.5 9.0 8.5 8.0 7.5 7.0 6.5 6.0 5.5 5.0 4.5 4.0 3.5 3.0 2.5 2.0 1.5 1.0 0.5 0.0 −0.5
f1 (ppm)

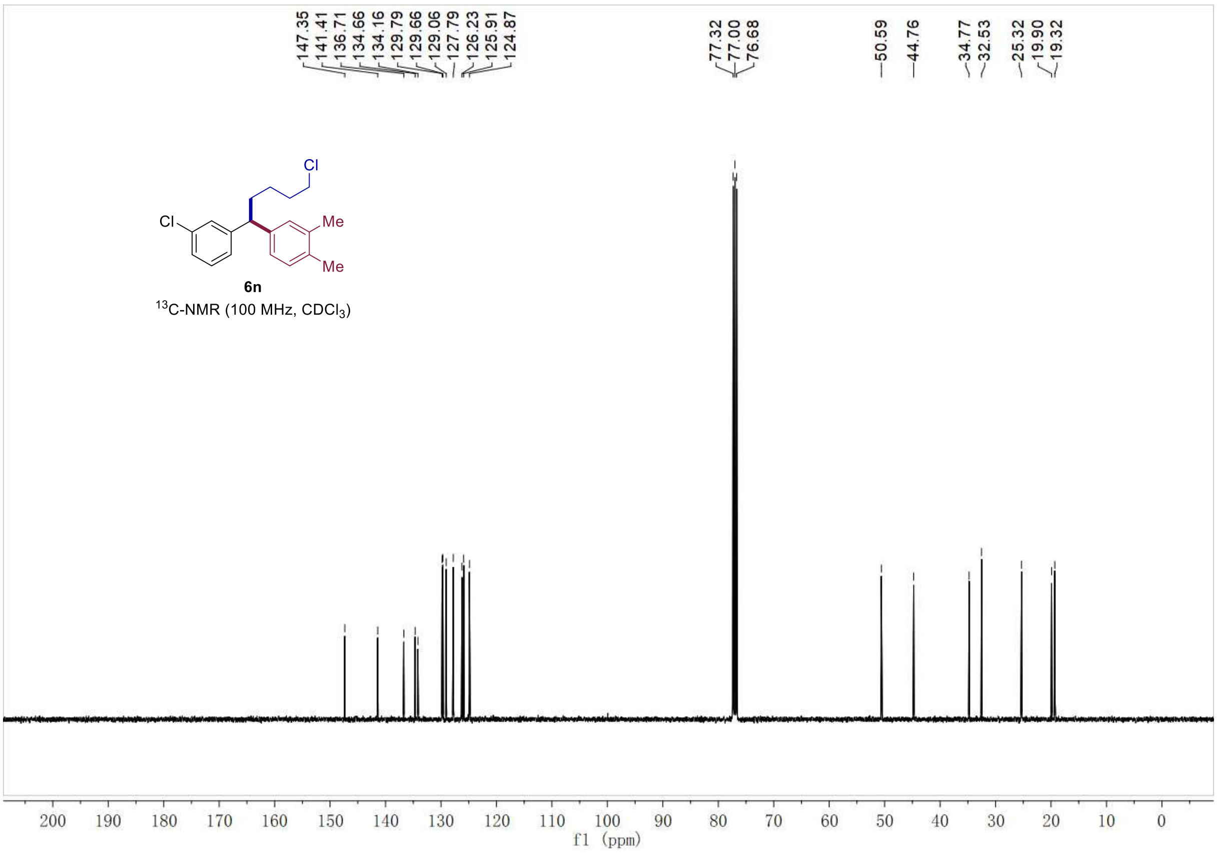
147.35
141.41
136.71
134.66
134.16
129.79
129.66
129.06
127.79
126.23
125.91
124.87
77.32
77.00
76.68
50.59
44.76
34.77
32.53
25.32
19.90
19.32
Cl
Cl
Me
Me
6n
^{13}C-NMR (100 MHz, CDCl$_3$)
f1 (ppm)

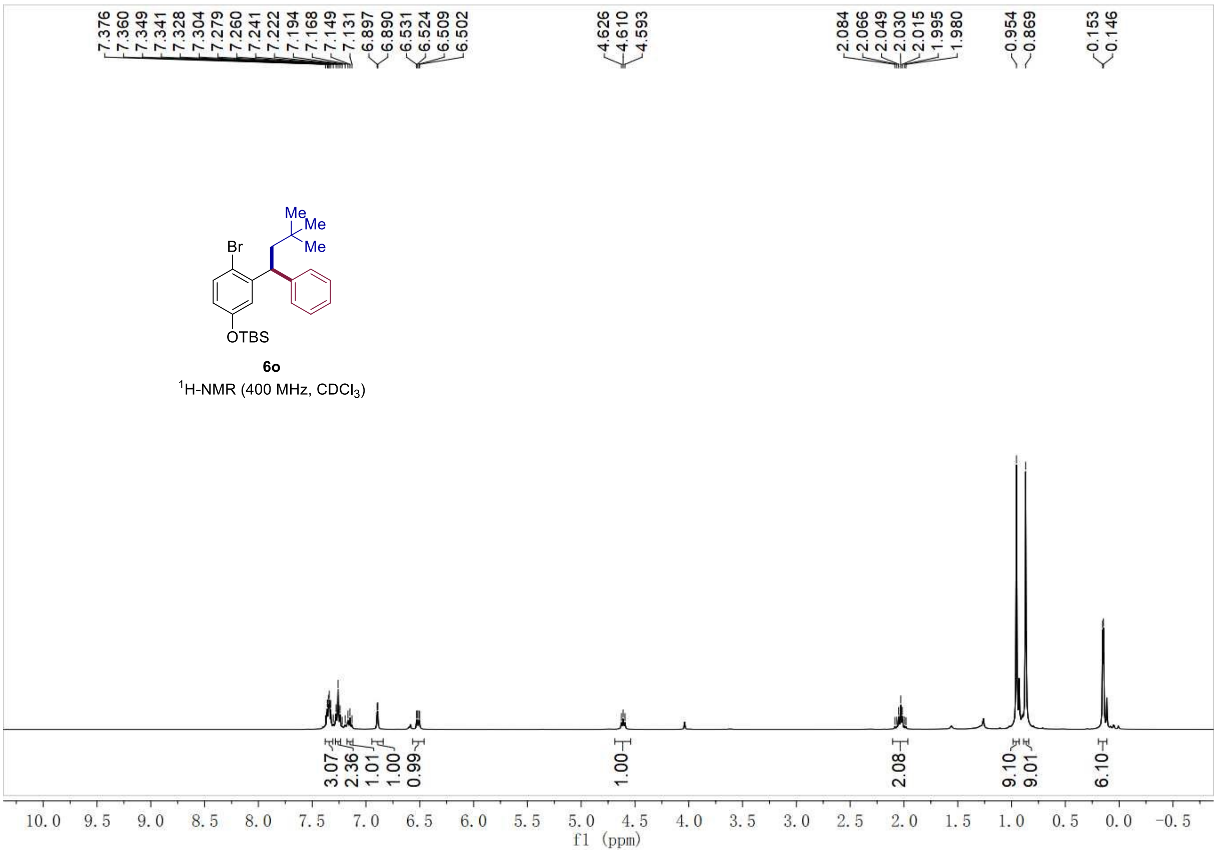
7.376
7.360
7.349
7.341
7.328
7.304
7.279
7.260
7.241
7.222
7.194
7.168
7.149
7.131
6.897
6.890
6.531
6.524
6.509
6.502
4.626
4.610
4.593
2.084
2.066
2.049
2.030
2.015
1.995
1.980
0.954
0.869
0.153
0.146
Br
Me
Me
Me
OTBS
6o
^{1}H-NMR (400 MHz, CDCl$_3$)
3.07
2.36
1.01
1.00
0.99
1.00
2.08
9.10
9.01
6.10
f1 (ppm)

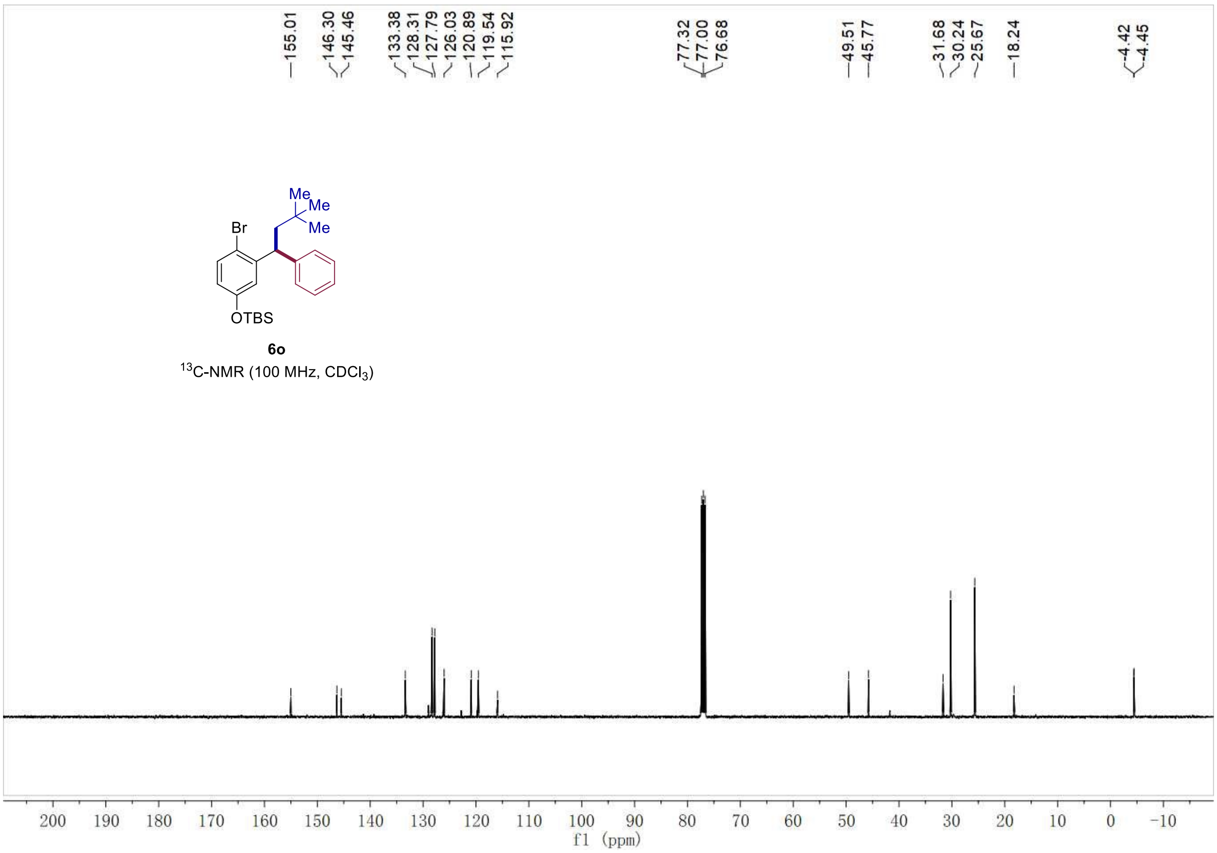

6o

^{13}C-NMR (100 MHz, $CDCl_3$)

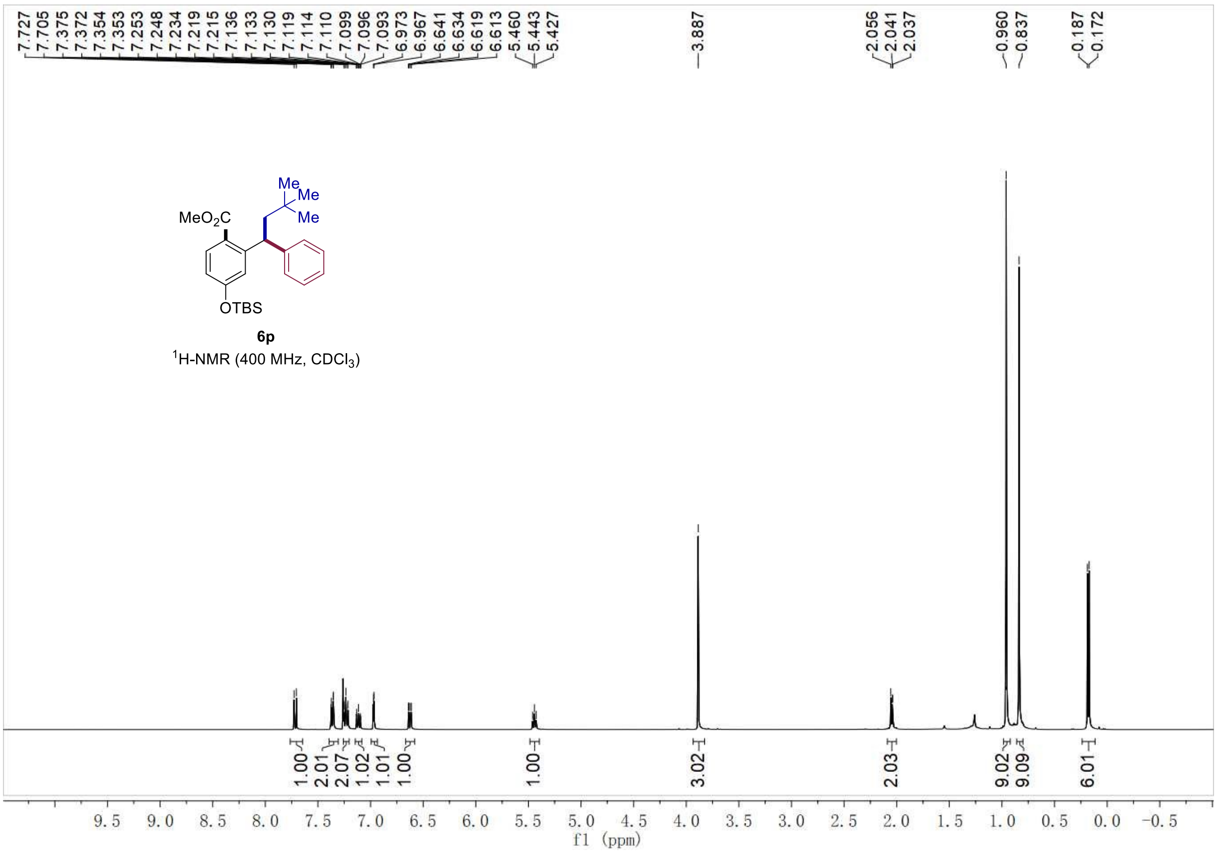

6p

^{1}H-NMR (400 MHz, $CDCl_3$)

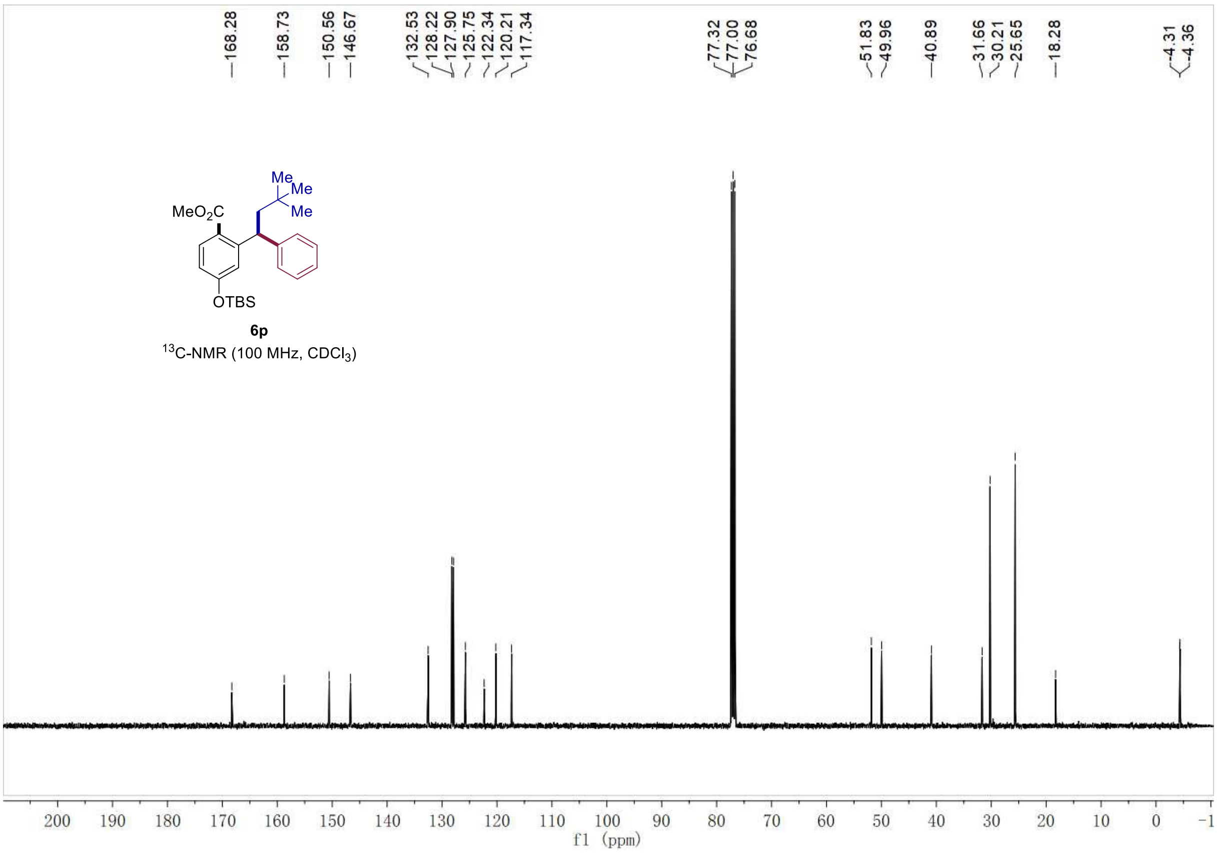
168.28
158.73
150.56
146.67
132.53
128.22
127.90
125.75
122.34
120.21
117.34
77.32
77.00
76.68
51.83
49.96
40.89
31.66
30.21
25.65
18.28
-4.31
-4.36
MeO2C
Me
Me
Me
OTBS
6p
13C-NMR (100 MHz, CDCl3)
f1 (ppm)

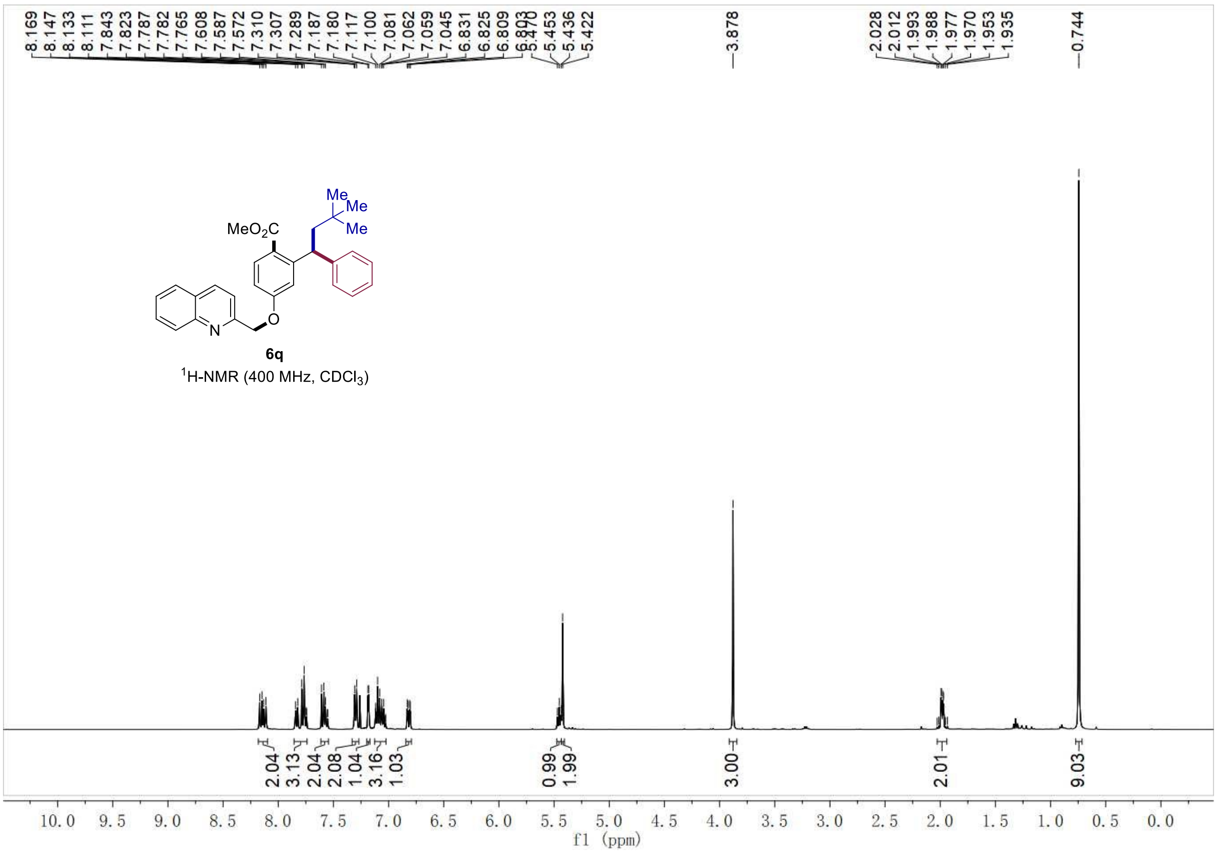
8.169
8.147
8.133
8.111
7.843
7.823
7.787
7.782
7.765
7.608
7.587
7.572
7.310
7.307
7.289
7.187
7.180
7.117
7.100
7.081
7.062
7.059
7.045
6.831
6.825
6.809
6.803
5.470
5.453
5.436
5.422
3.878
2.028
2.012
1.993
1.988
1.977
1.970
1.953
1.935
0.744
MeO2C
Me
Me
Me
N
O
6q
1H-NMR (400 MHz, CDCl3)
2.04
3.13
2.04
2.08
1.04
3.16
1.03
0.99
1.99
3.00
2.01
9.03
f1 (ppm)

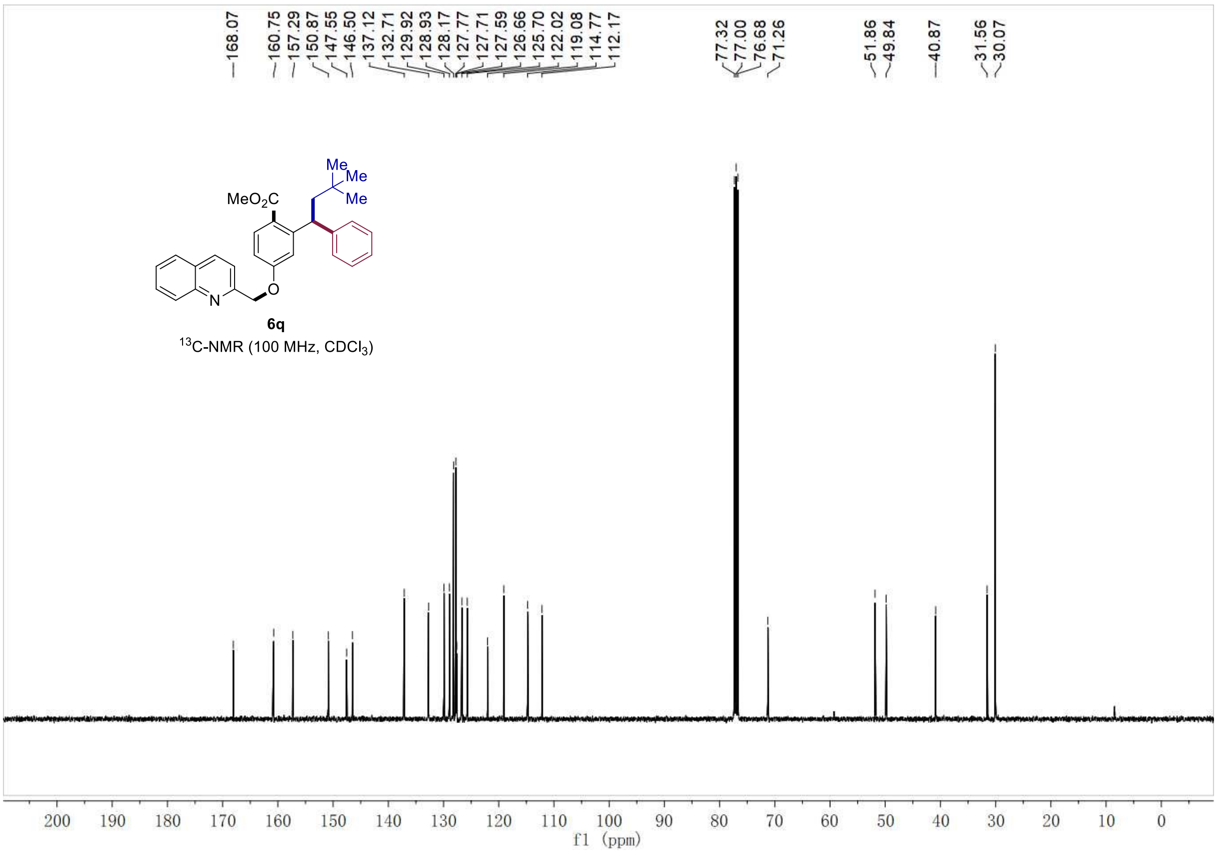

NMR Spectra of **8a-8e**

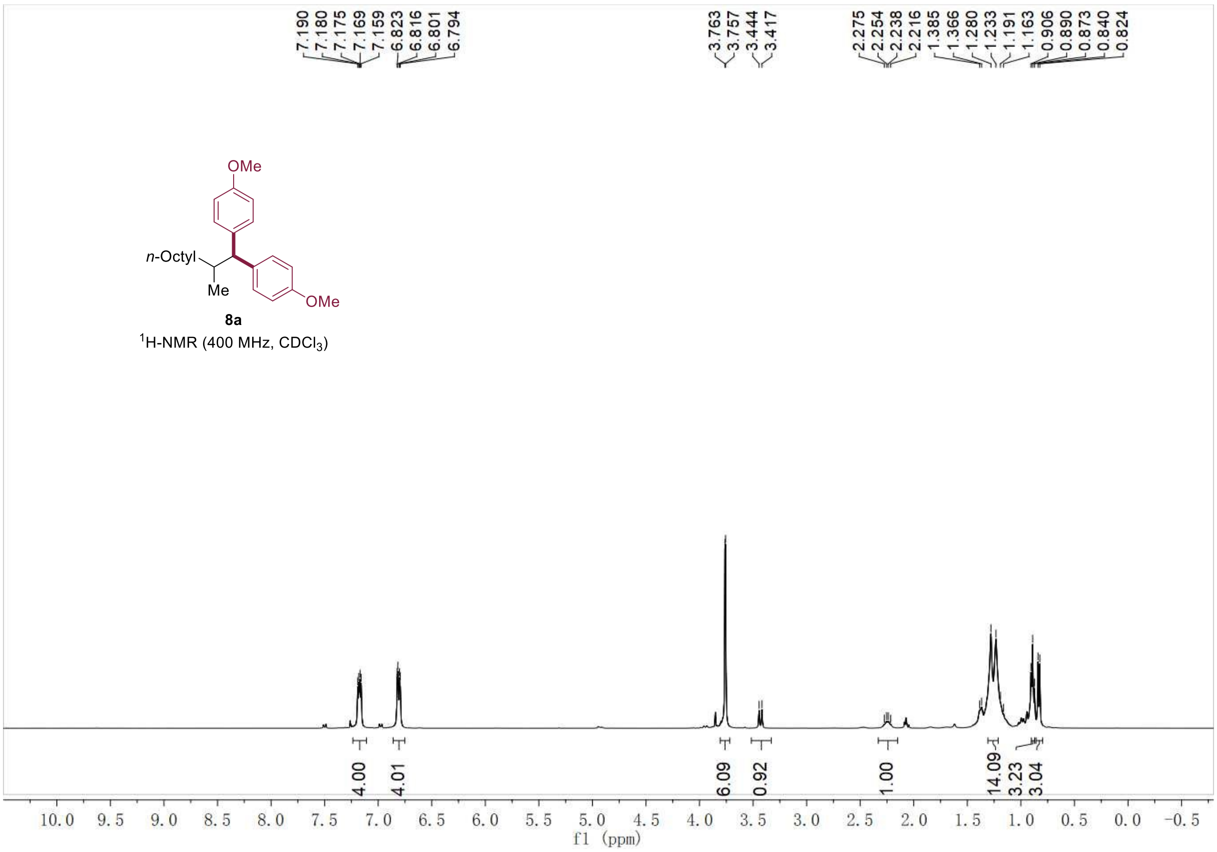

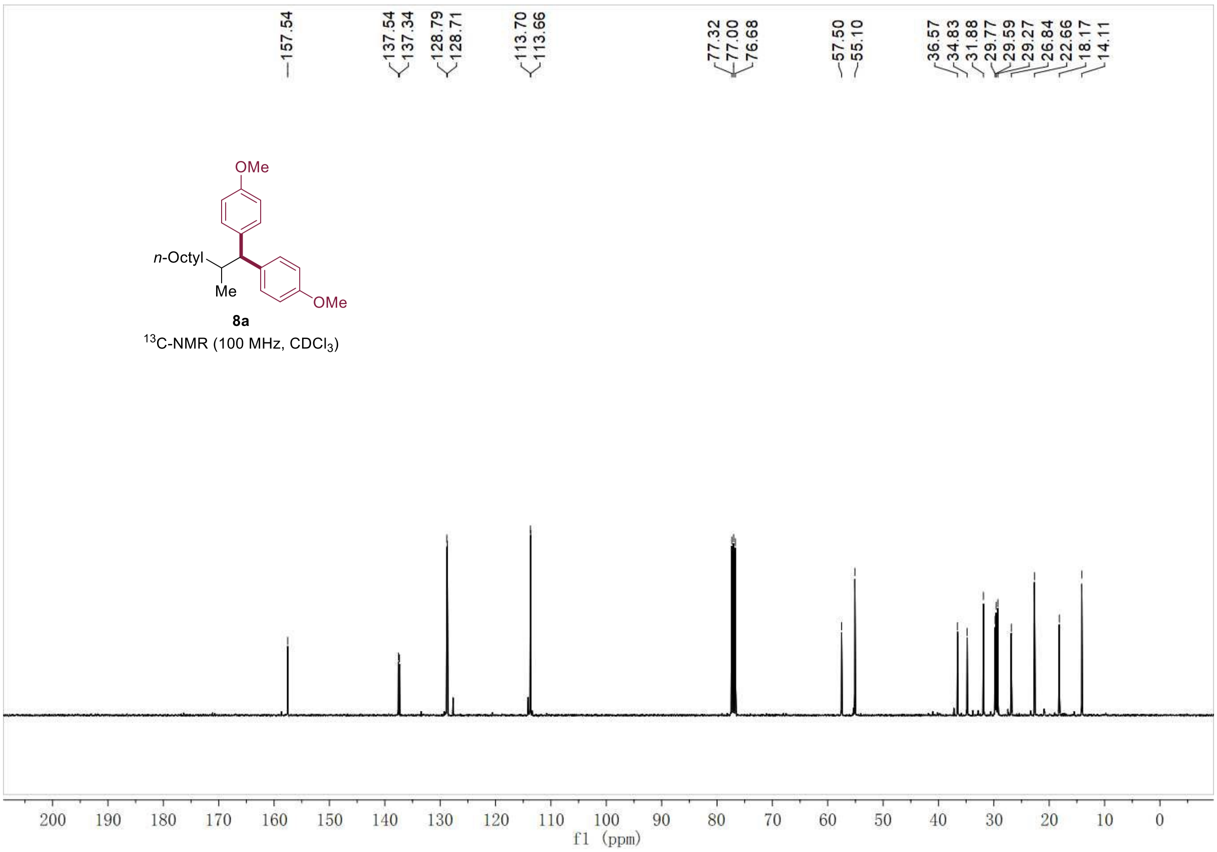
157.54
137.54
137.34
128.79
128.71
113.70
113.66
77.32
77.00
76.68
57.50
55.10
36.57
34.83
31.88
29.77
29.59
29.27
26.84
22.66
18.17
14.11
OMe
n-Octyl
Me
OMe
8a
13C-NMR (100 MHz, CDCl3)
200 190 180 170 160 150 140 130 120 110 100 90 80 70 60 50 40 30 20 10 0
f1 (ppm)

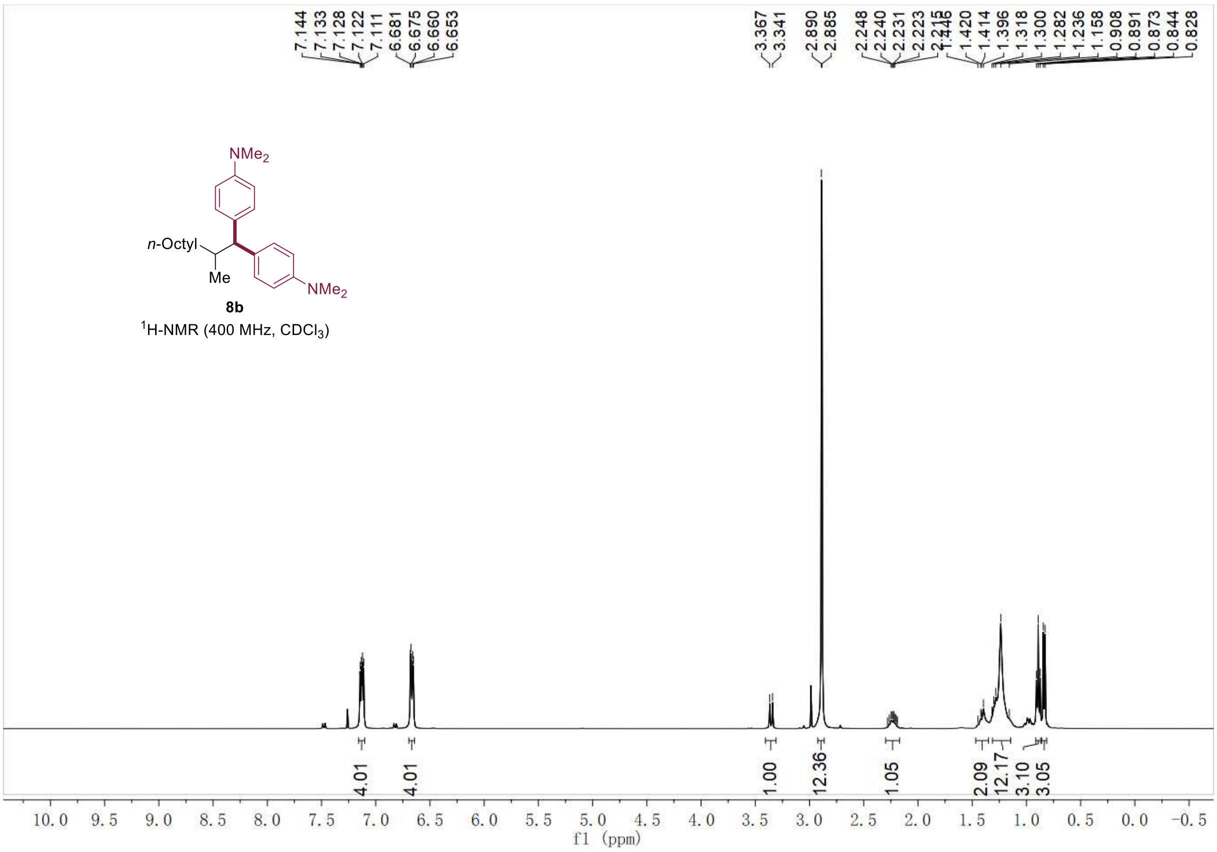
7.144
7.133
7.128
7.122
7.111
6.681
6.675
6.660
6.653
3.367
3.341
2.890
2.885
2.248
2.240
2.231
2.223
1.420
1.414
1.396
1.318
1.300
1.282
1.236
1.158
0.908
0.891
0.873
0.844
0.828
NMe2
n-Octyl
Me
NMe2
8b
1H-NMR (400 MHz, CDCl3)
4.01
4.01
1.00
12.36
1.05
2.09
12.17
3.10
3.05
10.0 9.5 9.0 8.5 8.0 7.5 7.0 6.5 6.0 5.5 5.0 4.5 4.0 3.5 3.0 2.5 2.0 1.5 1.0 0.5 0.0 -0.5
f1 (ppm)

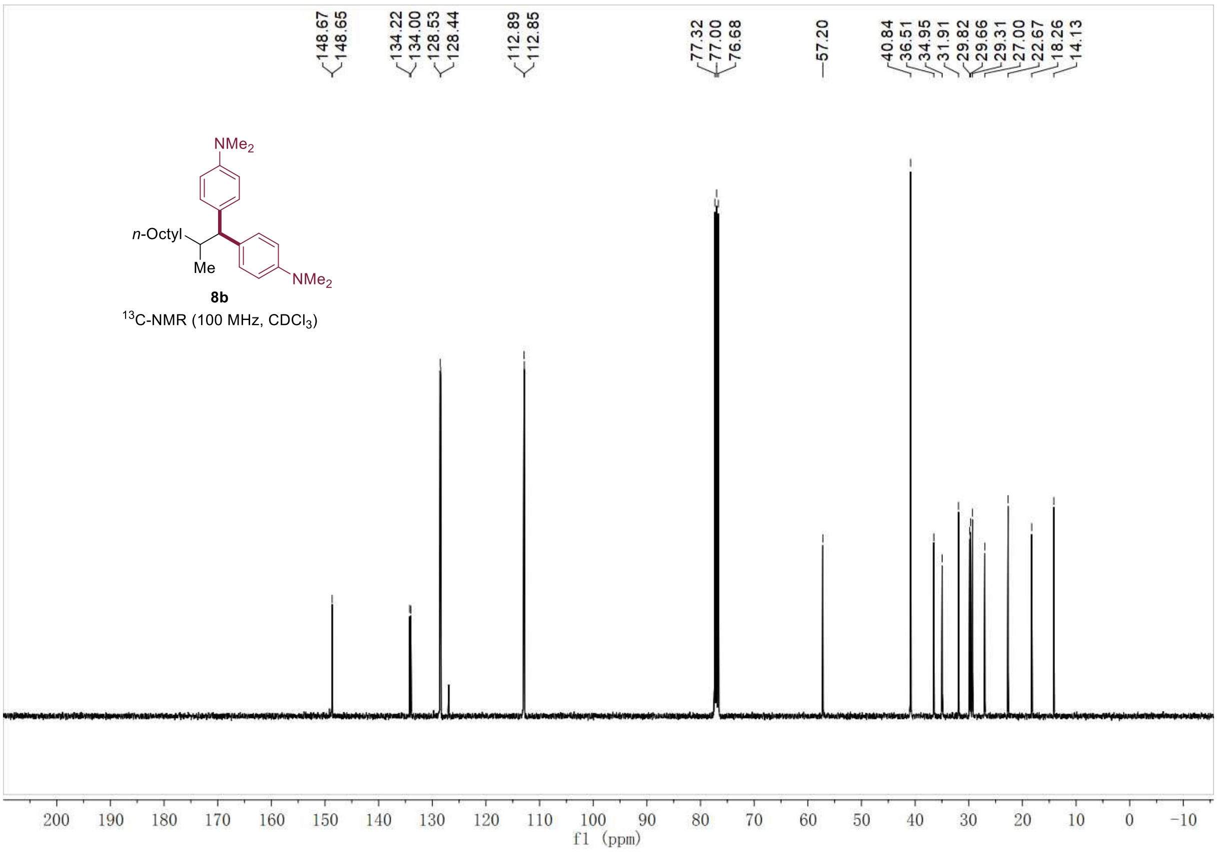

8b

^{13}C-NMR (100 MHz, $CDCl_3$)

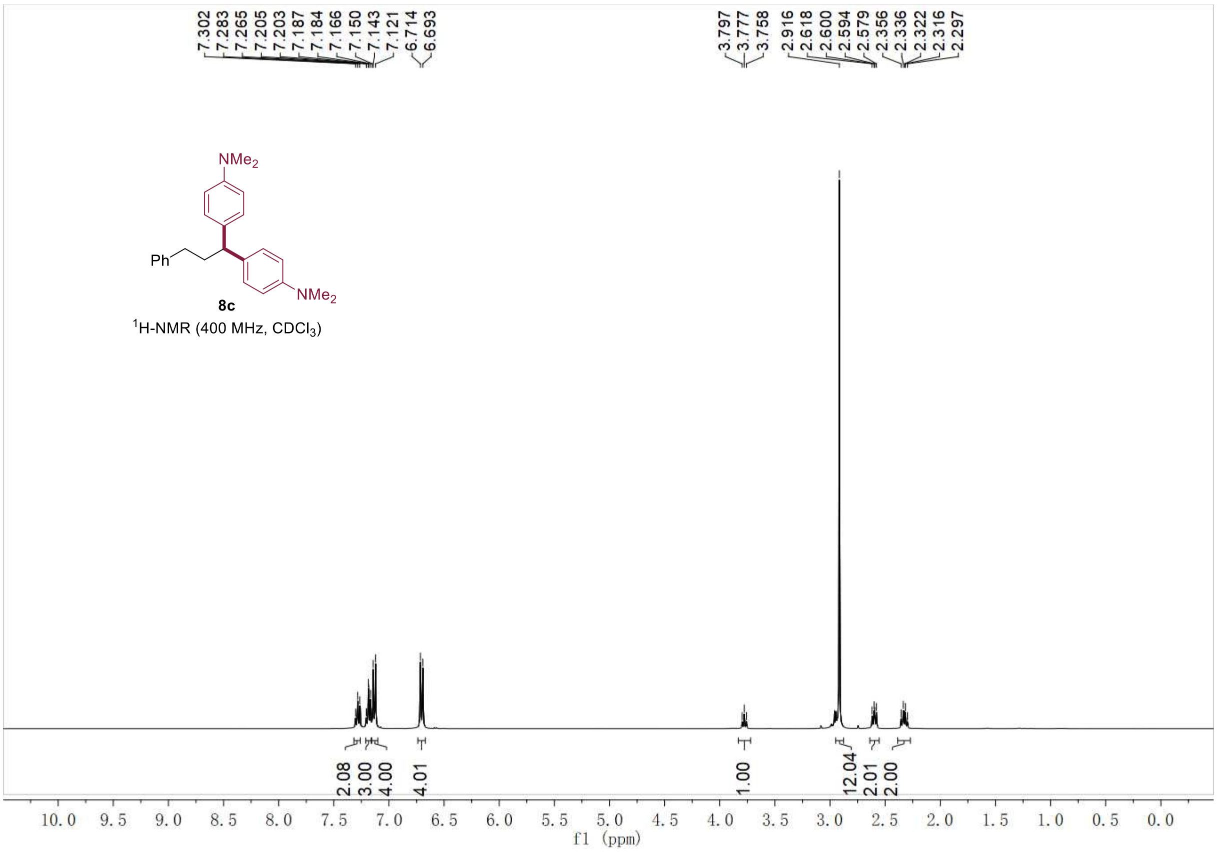

8c

^{1}H-NMR (400 MHz, $CDCl_3$)

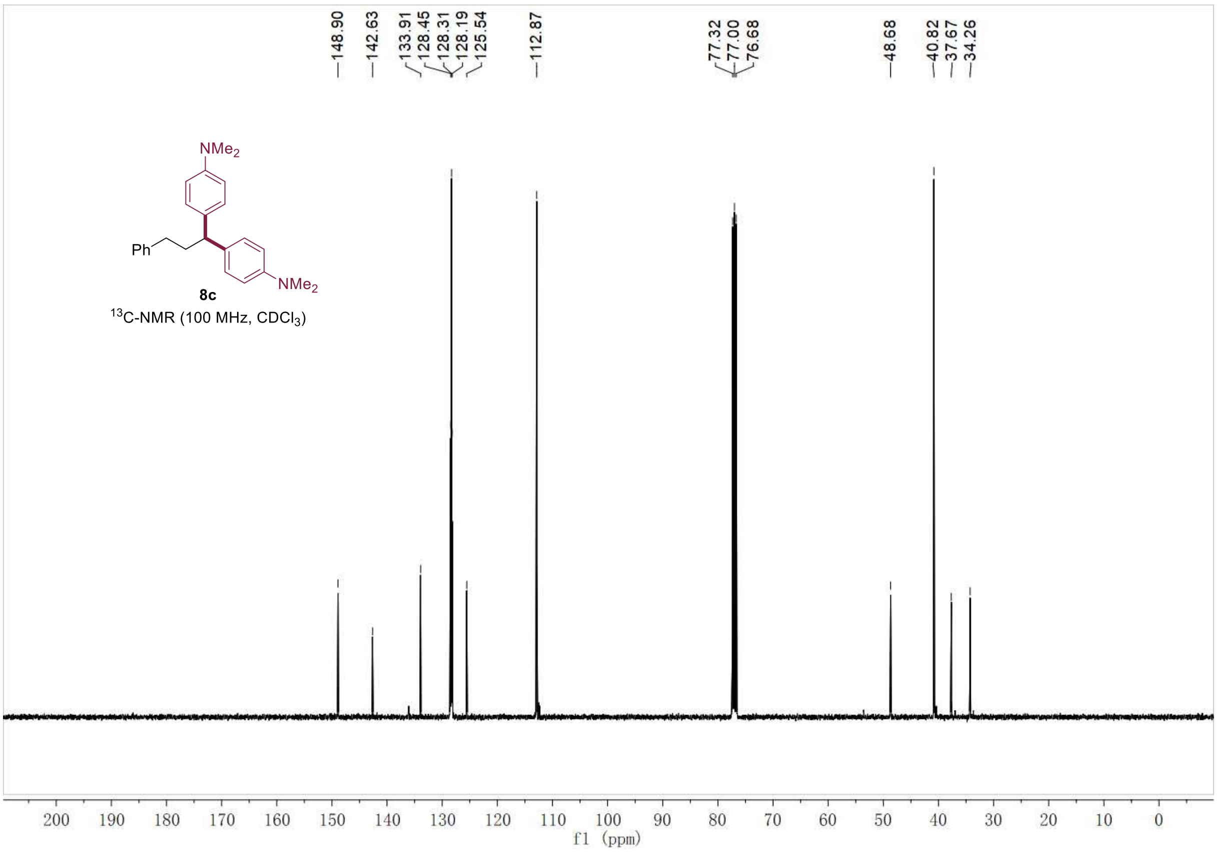
148.90
142.63
133.91
128.45
128.31
128.19
125.54
112.87
77.32
77.00
76.68
48.68
40.82
37.67
34.26
NMe2
Ph
NMe2
8c
13C-NMR (100 MHz, CDCl3)
f1 (ppm)

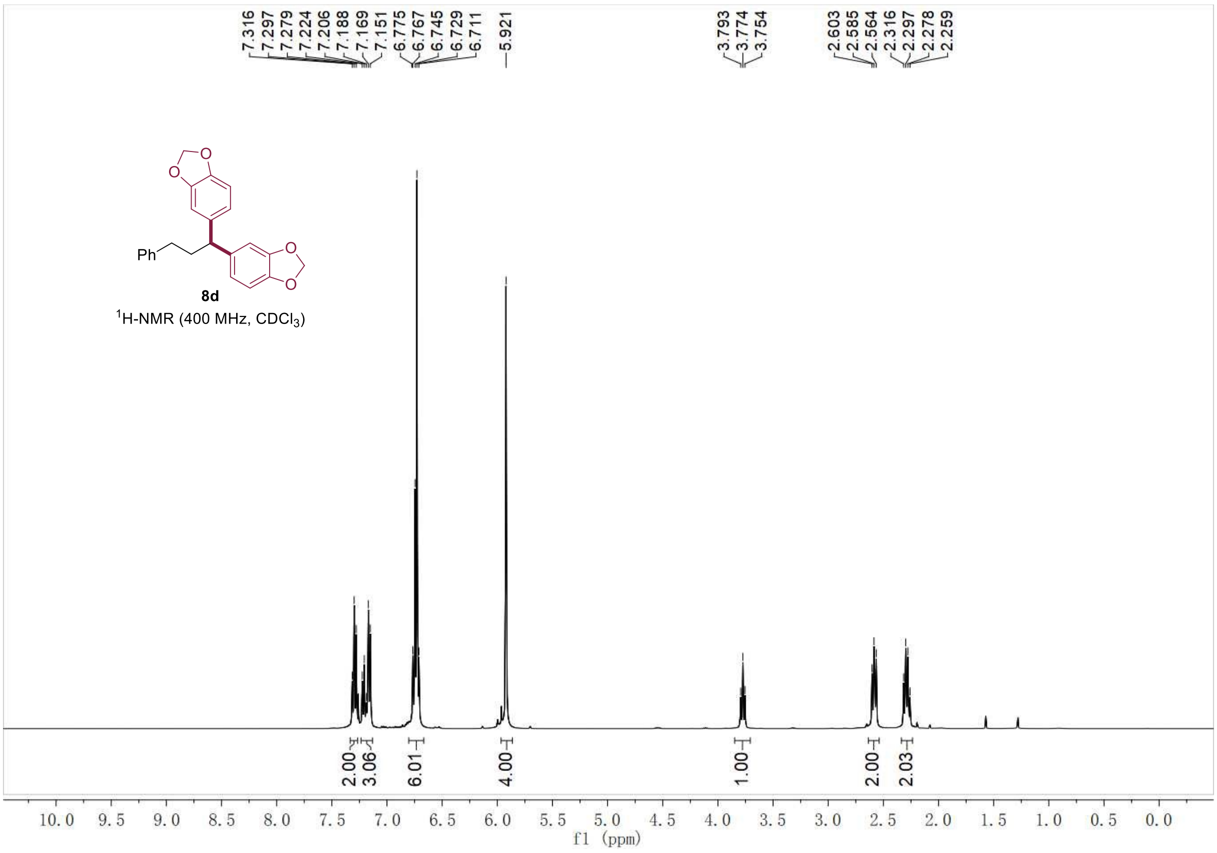
7.316
7.297
7.279
7.224
7.206
7.188
7.169
7.151
6.775
6.767
6.745
6.729
6.711
5.921
3.793
3.774
3.754
2.603
2.585
2.564
2.316
2.297
2.278
2.259
Ph
8d
1H-NMR (400 MHz, CDCl3)
2.00
3.06
6.01
4.00
1.00
2.00
2.03
f1 (ppm)

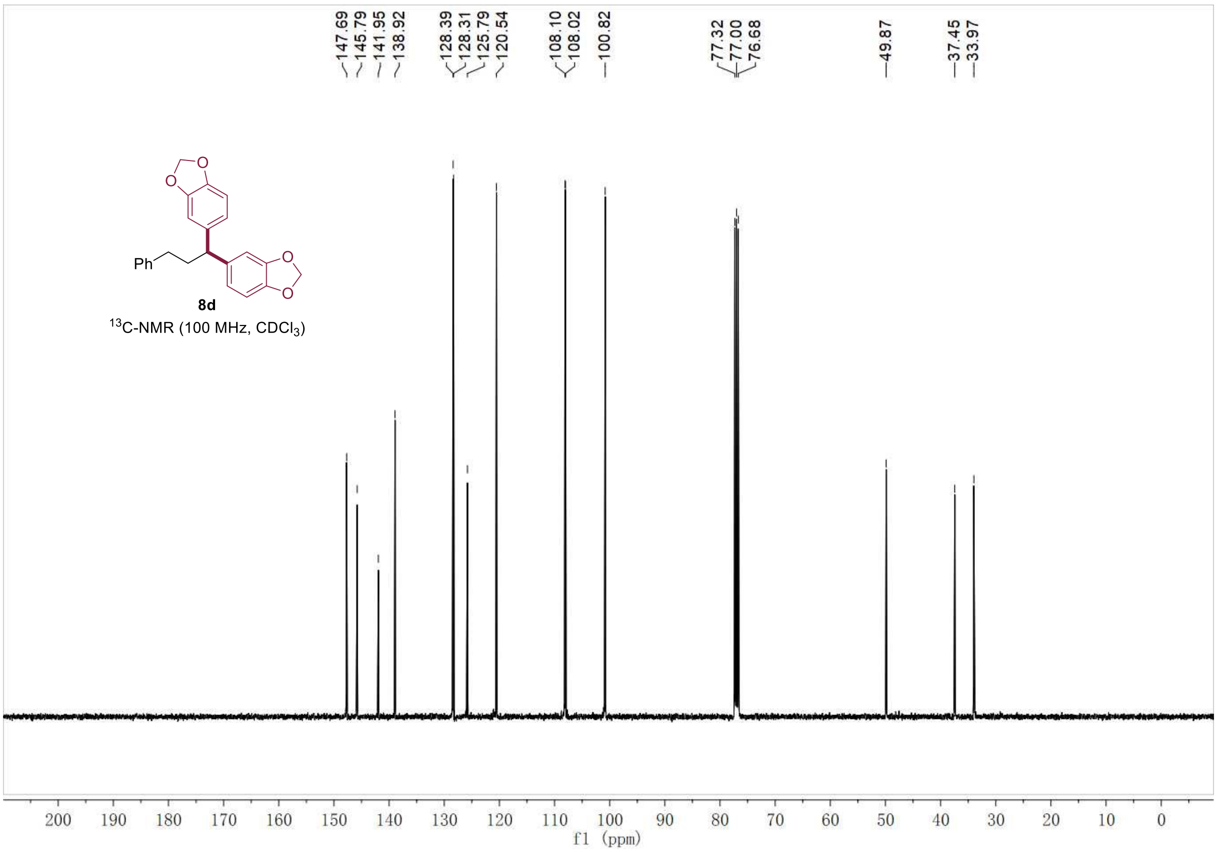
147.69
145.79
141.95
138.92
128.39
128.31
125.79
120.54
108.10
108.02
100.82
77.32
77.00
76.68
49.87
37.45
33.97
Ph
8d
13C-NMR (100 MHz, CDCl3)
f1 (ppm)

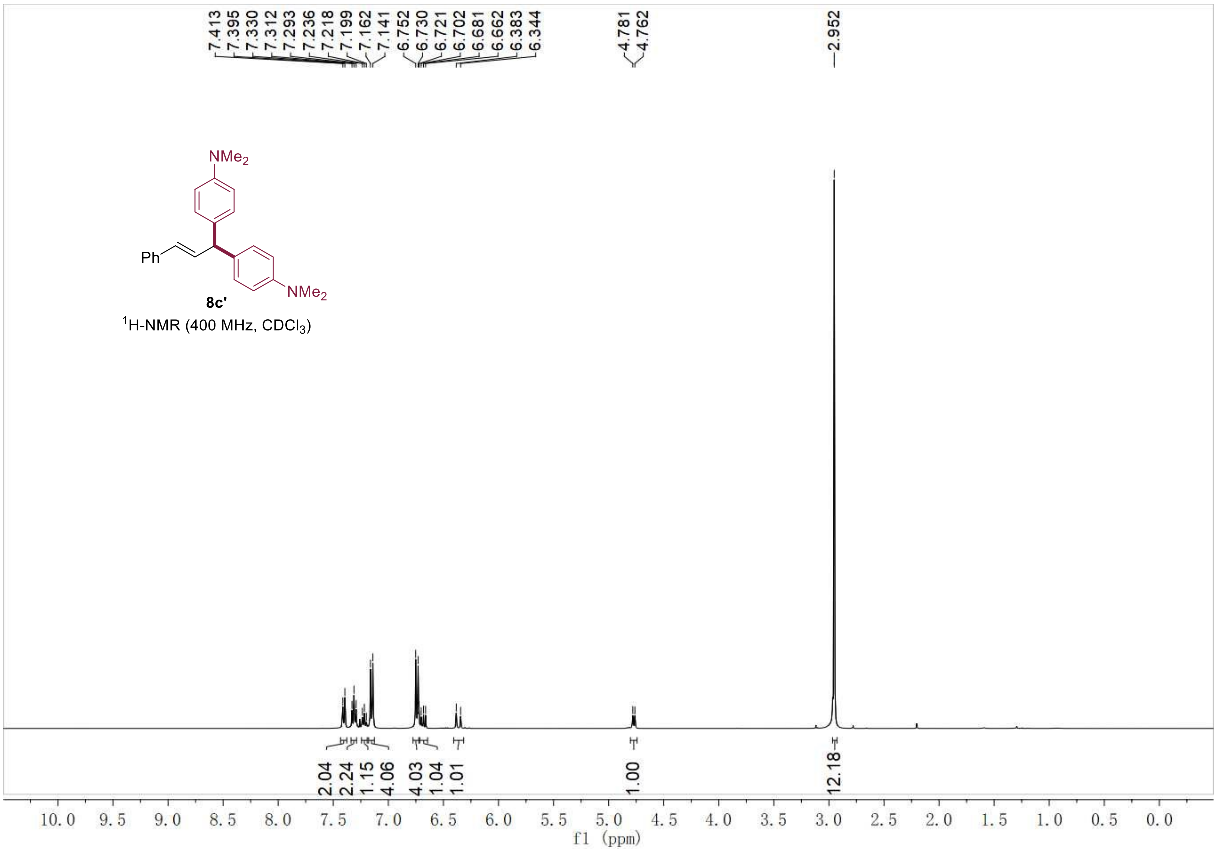
7.413
7.395
7.330
7.312
7.293
7.236
7.218
7.199
7.162
7.141
6.752
6.730
6.721
6.702
6.681
6.662
6.383
6.344
4.781
4.762
2.952
NMe2
Ph
NMe2
8c'
1H-NMR (400 MHz, CDCl3)
2.04
2.24
1.15
4.06
4.03
1.04
1.01
1.00
12.18
f1 (ppm)

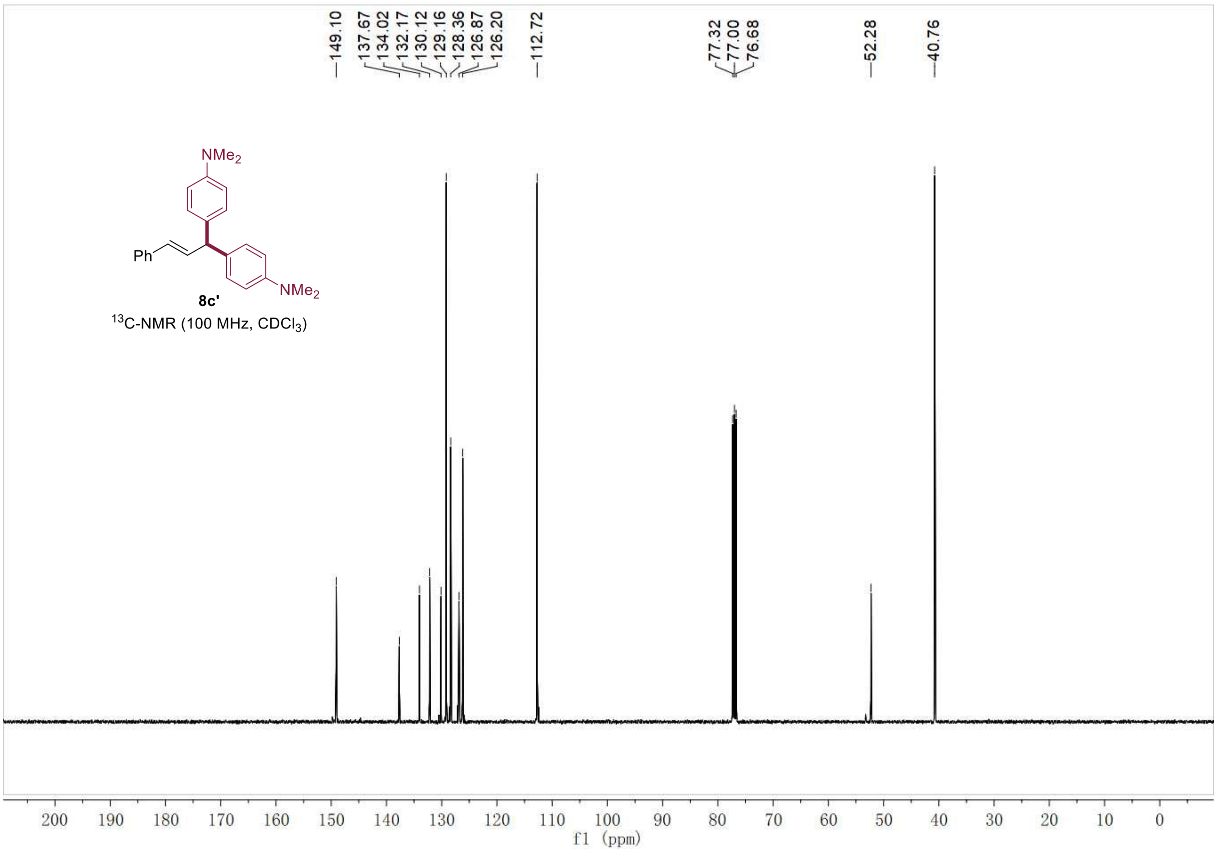
149.10
137.67
134.02
132.17
130.12
129.16
128.36
126.87
126.20
112.72
77.32
77.00
76.68
52.28
40.76
NMe2
Ph
NMe2
8c'
13C-NMR (100 MHz, CDCl3)
200 190 180 170 160 150 140 130 120 110 100 90 80 70 60 50 40 30 20 10 0
f1 (ppm)

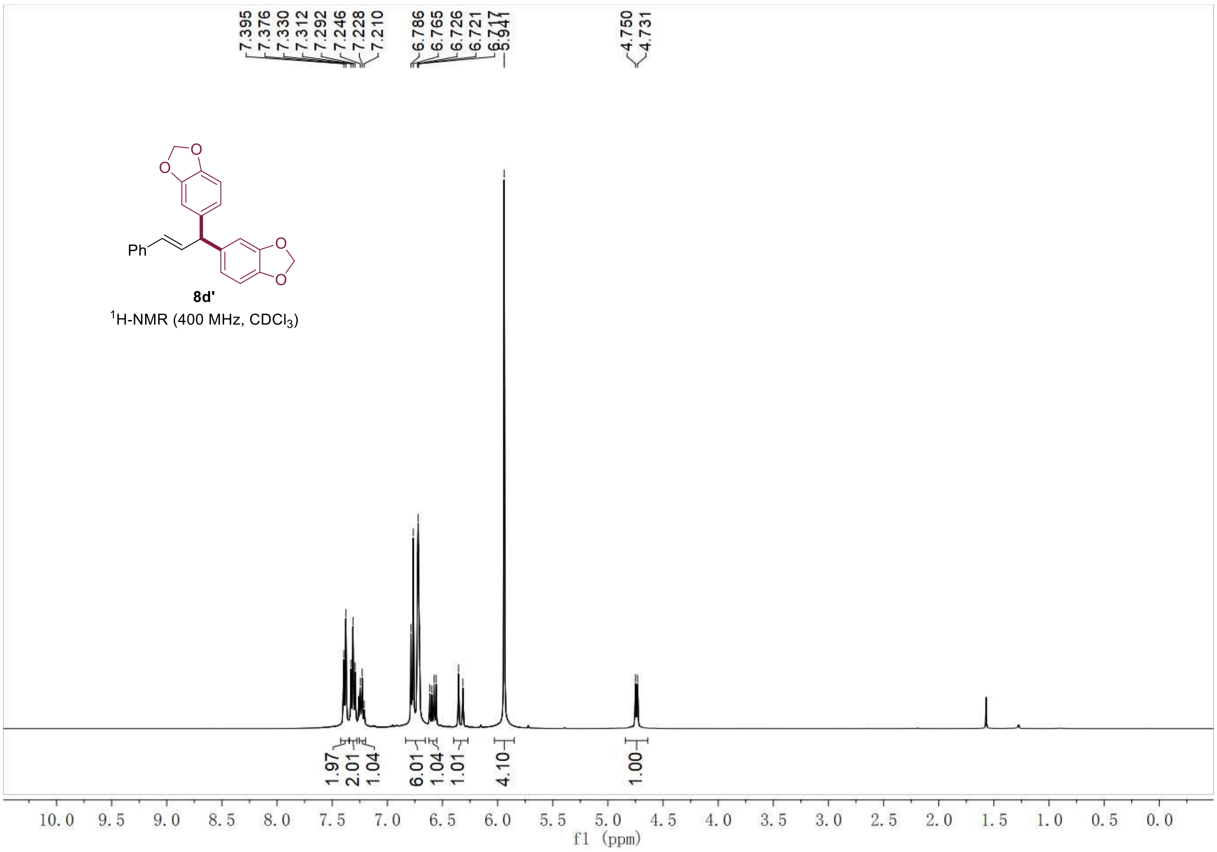
7.395
7.376
7.330
7.312
7.292
7.246
7.228
7.210
6.786
6.765
6.726
6.721
6.717
5.941
4.750
4.731
Ph
8d'
1H-NMR (400 MHz, CDCl3)
1.97
2.01
1.04
6.01
1.04
1.01
4.10
1.00
10.0 9.5 9.0 8.5 8.0 7.5 7.0 6.5 6.0 5.5 5.0 4.5 4.0 3.5 3.0 2.5 2.0 1.5 1.0 0.5 0.0
f1 (ppm)

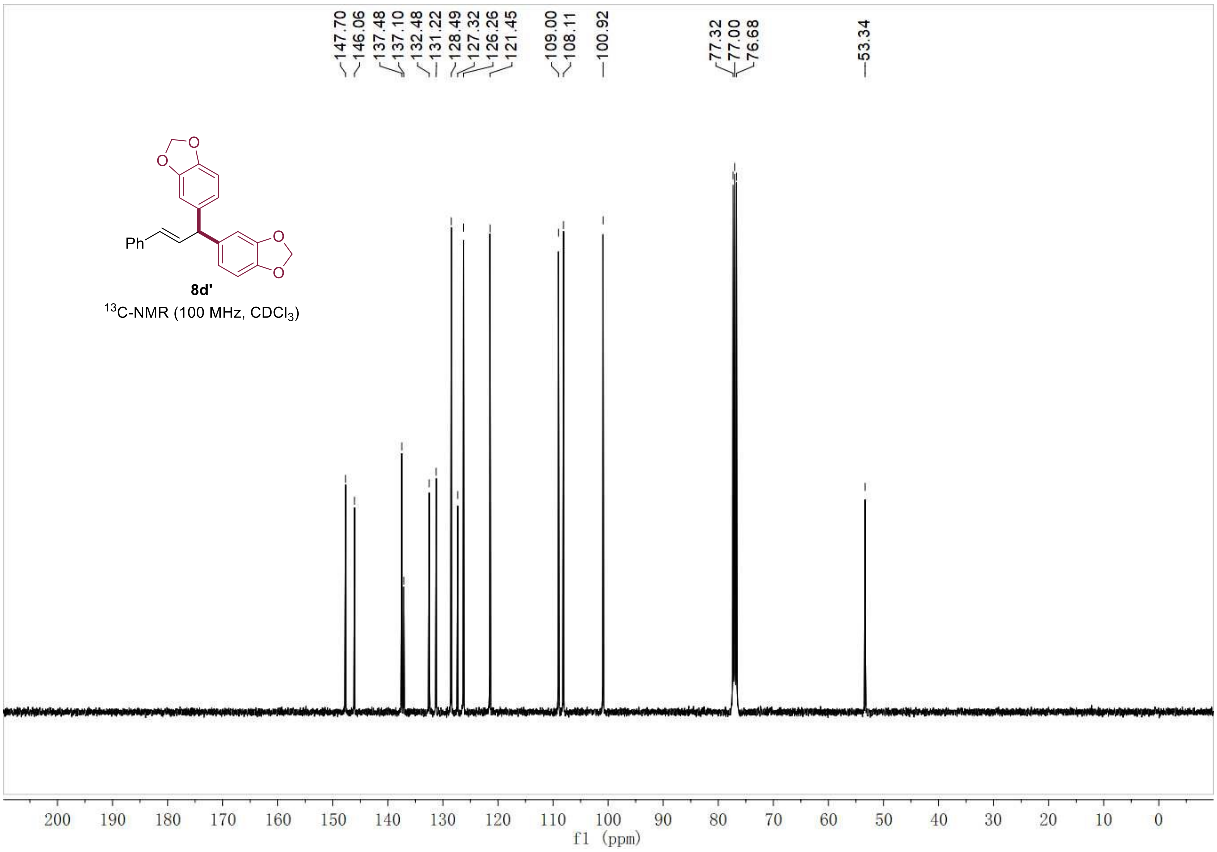
147.70
146.06
137.48
137.10
132.48
131.22
128.49
127.32
126.26
121.45
109.00
108.11
100.92
77.32
77.00
76.68
53.34
Ph
8d'
^{13}C-NMR (100 MHz, $CDCl_3$)
200 190 180 170 160 150 140 130 120 110 100 90 80 70 60 50 40 30 20 10 0
f1 (ppm)

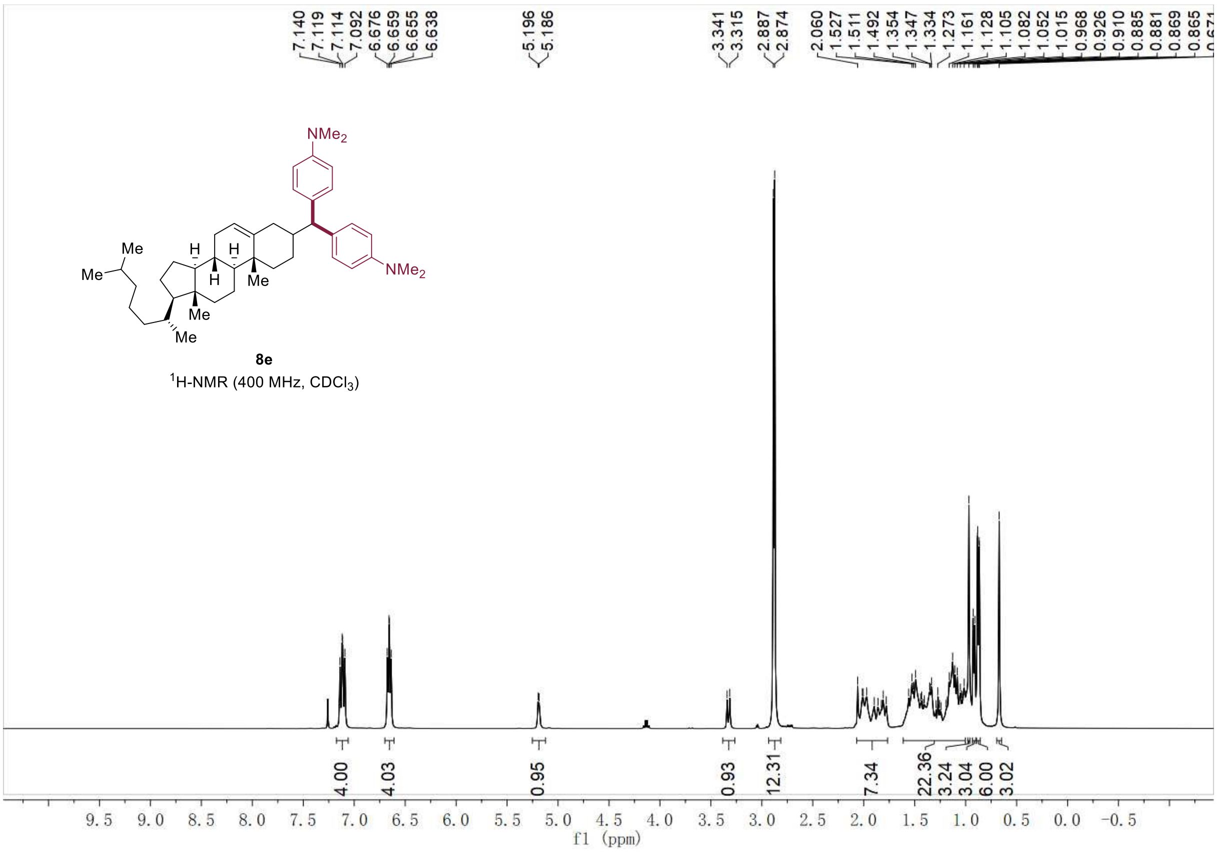
7.140
7.119
7.114
7.092
6.676
6.659
6.655
6.638
5.196
5.186
3.341
3.315
2.887
2.874
2.060
1.527
1.511
1.492
1.354
1.347
1.334
1.273
1.161
1.128
1.105
1.082
1.052
1.015
0.968
0.926
0.910
0.885
0.881
0.869
0.865
NMe_2
Me
H
8e
^{1}H-NMR (400 MHz, $CDCl_3$)
4.00
4.03
0.95
0.93
12.31
7.34
22.36
3.24
3.04
6.00
3.02
9.5 9.0 8.5 8.0 7.5 7.0 6.5 6.0 5.5 5.0 4.5 4.0 3.5 3.0 2.5 2.0 1.5 1.0 0.5 0.0 -0.5
f1 (ppm)

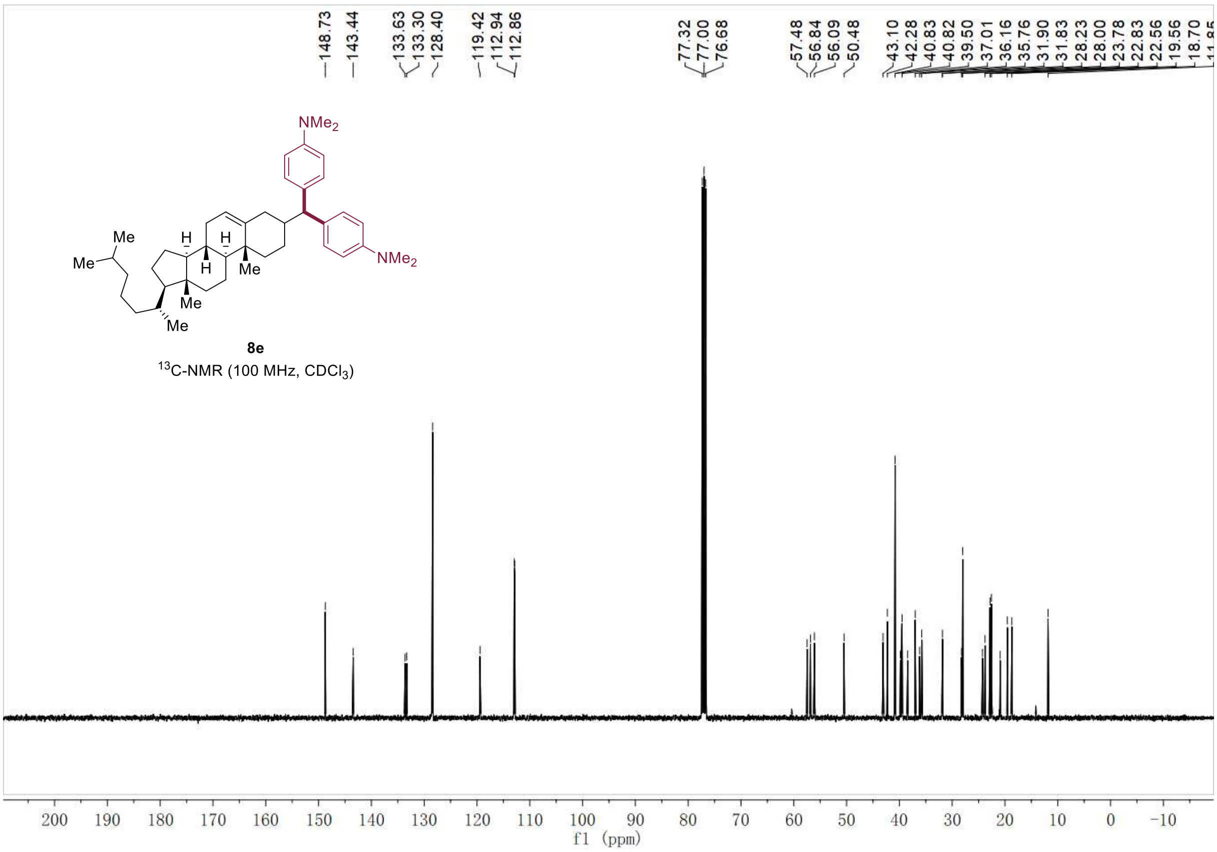

NMR Spectra of **10** and **11**

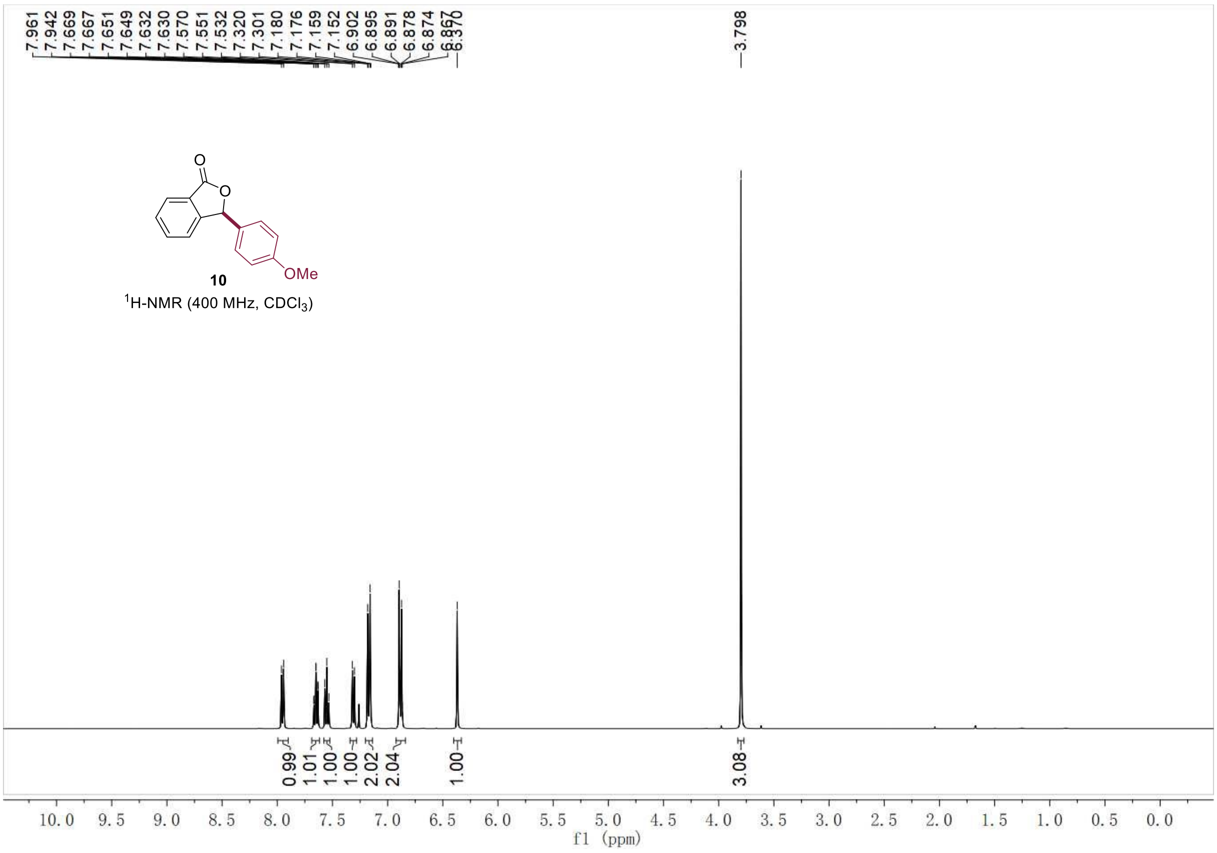

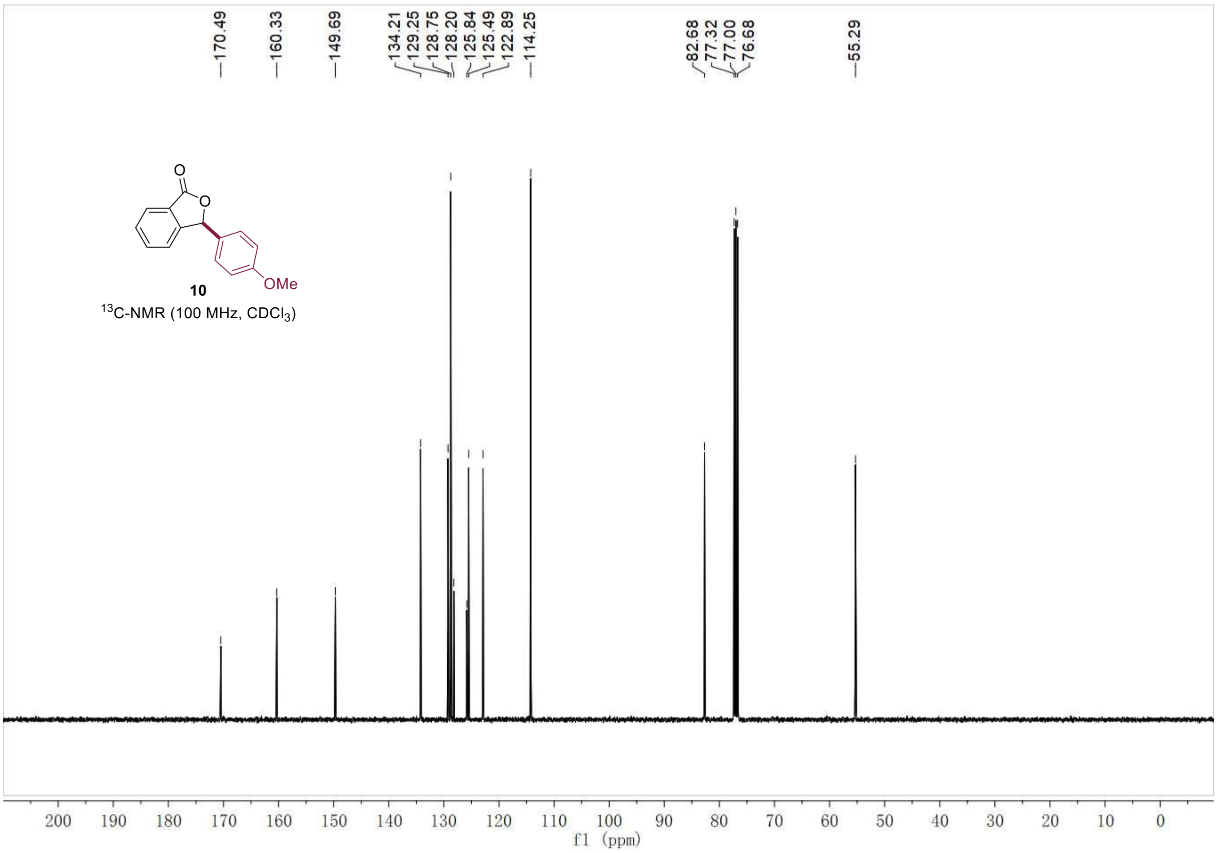
170.49
160.33
149.69
134.21
129.25
128.75
128.20
125.84
125.49
122.89
114.25
82.68
77.32
77.00
76.68
55.29
OMe
10
^{13}C-NMR (100 MHz, CDCl$_3$)
200 190 180 170 160 150 140 130 120 110 100 90 80 70 60 50 40 30 20 10 0
f1 (ppm)

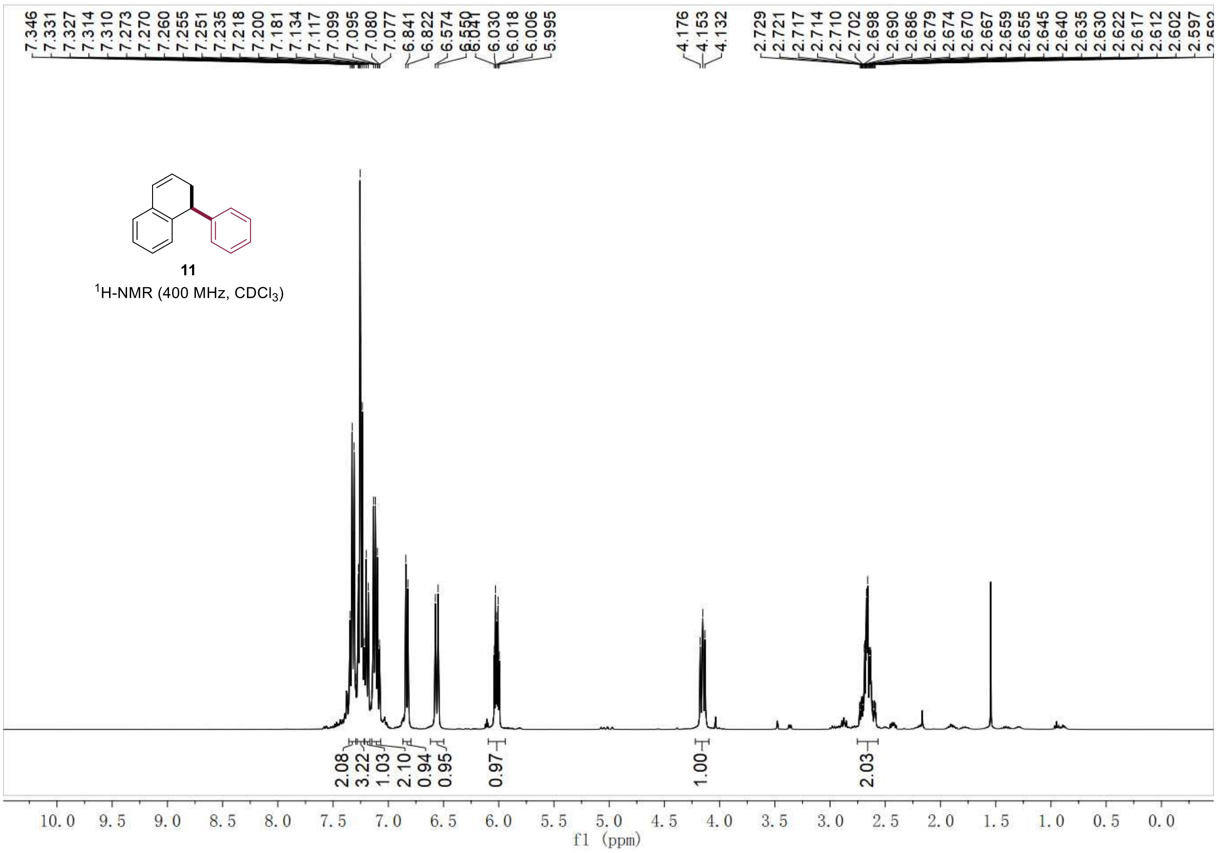
7.346
7.331
7.327
7.314
7.310
7.273
7.270
7.260
7.255
7.251
7.235
7.218
7.200
7.181
7.134
7.117
7.099
7.095
7.080
7.077
6.841
6.822
6.574
6.550
6.041
6.030
6.018
6.006
5.995
4.176
4.153
4.132
2.729
2.721
2.717
2.714
2.710
2.702
2.698
2.690
2.686
2.679
2.674
2.670
2.667
2.659
2.655
2.645
2.640
2.635
2.630
2.622
2.617
2.612
2.602
2.597
11
^{1}H-NMR (400 MHz, CDCl$_3$)
2.08
3.22
1.03
2.10
0.94
0.95
0.97
1.00
2.03
10.0 9.5 9.0 8.5 8.0 7.5 7.0 6.5 6.0 5.5 5.0 4.5 4.0 3.5 3.0 2.5 2.0 1.5 1.0 0.5 0.0
f1 (ppm)

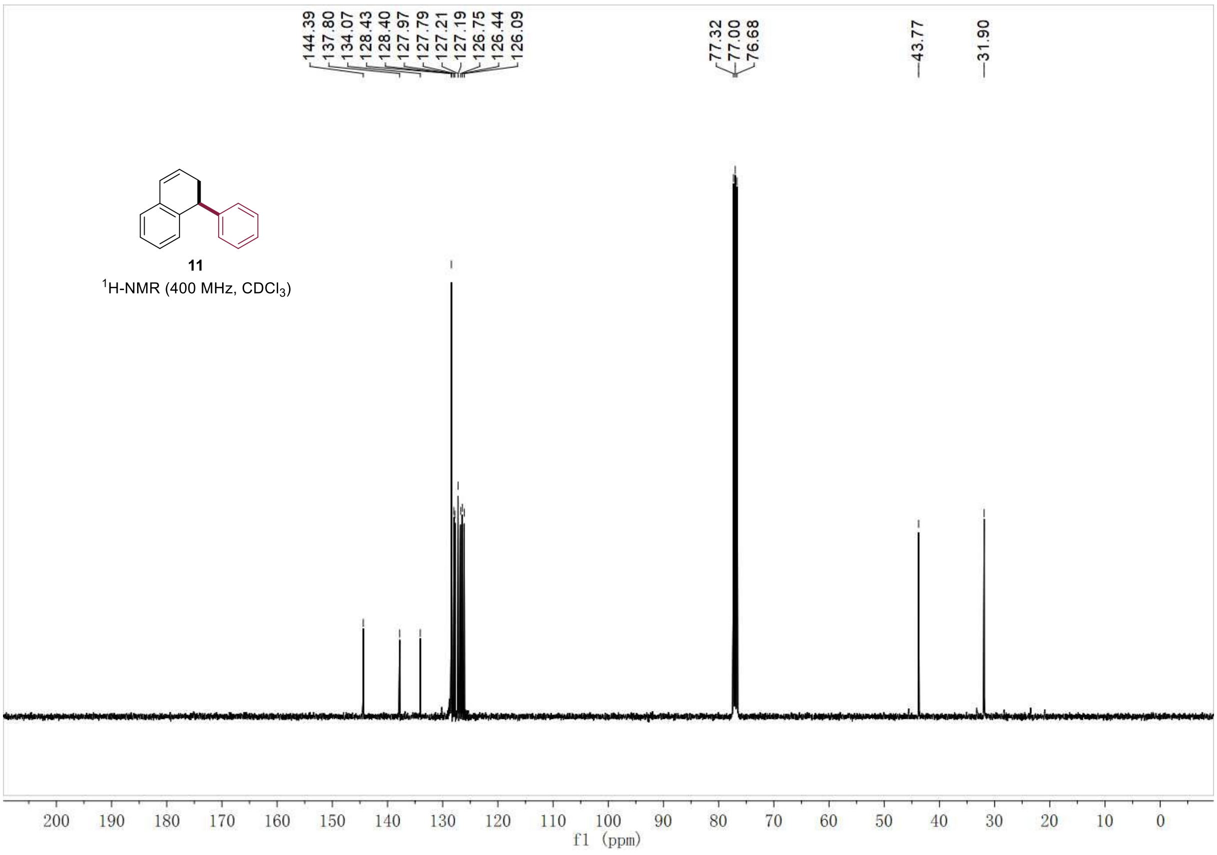
144.39
137.80
134.07
128.43
128.40
127.97
127.79
127.21
127.19
126.75
126.44
126.09
77.32
77.00
76.68
43.77
31.90
11
1H-NMR (400 MHz, CDCl3)
200 190 180 170 160 150 140 130 120 110 100 90 80 70 60 50 40 30 20 10 0
f1 (ppm)
